# Collision-Induced Dissociations of Linear Hexose and Disaccharides with Linear Hexose at the Reducing End

**DOI:** 10.1021/acs.jpca.4c05929

**Published:** 2024-11-18

**Authors:** Hock-Seng Nguan, Hsu-Chen Hsu, Wun-Long Li, Chia Yen Liew, Chi-Kung Ni

**Affiliations:** 1 Institute of Atomic and Molecular Sciences, Academia Sinica, P.O. Box 23-166, Taipei 10617, Taiwan; 2 Department of Chemistry, National Taiwan Normal University, Taipei 11677, Taiwan; 3 Department of Chemistry, National Tsing Hua University, Hsinchu 30013, Taiwan

## Abstract

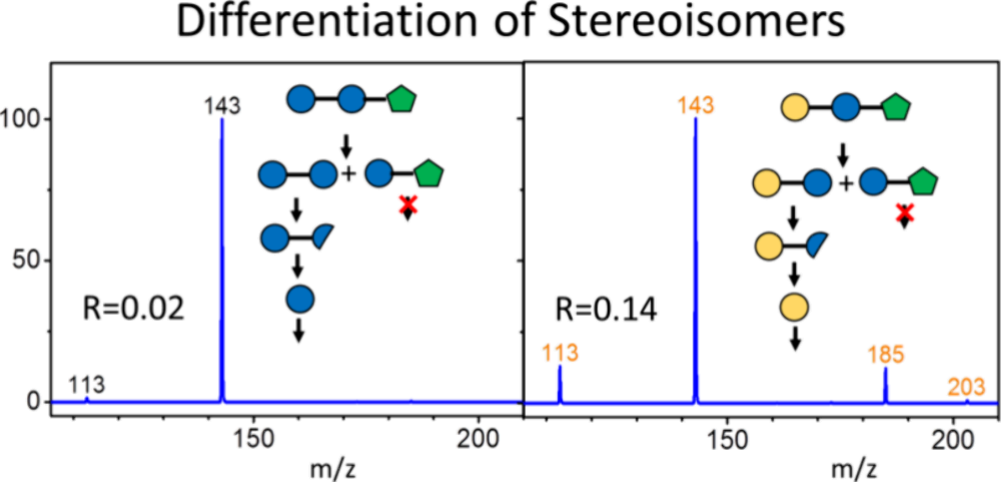

Characterization of carbohydrate structures using mass spectrometry is a challenging task. Understanding the dissociation mechanisms of carbohydrates in the gas phase is crucial for characterizing these structures through tandem mass spectrometry. In this study, we investigated the collision-induced dissociation (CID) of glucose, galactose, and mannose in their linear forms, as well as the linear forms of hexose at the reducing end of 1–6 linked disaccharides, using quantum chemistry calculations and tandem mass spectrometry. Our results suggest that the dehydration reaction in linear structures is unlikely to occur due to the significantly high reaction barrier compared to those of C=O migration and C–C bond cleavage. We demonstrate that the different intensities of the cross-ring fragments observed in the CID spectra can be explained by the different transition state energies of C=O migration and C2–C3, C3–C4, and C4–C5 bond cleavages, and the branching ratios of the cross-ring fragments are significantly different between glucose and galactose. The application of the cross-ring fragments to oligosaccharides reveals that the stereoisomers of glucose and galactose in oligosaccharides can be differentiated based on the relative intensities of the cross-ring fragments produced by the C2–C3 bond cleavage and C3–C4 bond cleavage, a differentiation that cannot be achieved by conventional tandem mass spectrometry.

## Introduction

Carbohydrates are the most abundant biomolecules among the four classes of biomolecules (nucleic acids, proteins, carbohydrates, and lipids) that are essential for life. They have many functions in biology, such as serving as an energy source, mediating cell–cell adhesion and signaling, and supporting the immune system.^1,2^ Understanding the biological functions of carbohydrates necessitates characterization of their structures. However, the number of carbohydrate isomers is vast due to the differences in the monosaccharide units and their sequences and linkages, making structural determination difficult.

Methods such as nuclear magnetic resonance spectroscopy^3,4^ and enzyme digestion^5−7^ are used traditionally for the carbohydrate structural determination. However, these methods often face challenges such as difficulties in obtaining a sufficient amount of samples and suitable enzymes for structural determination. Ion mobility spectrometry,^8−10^ infrared action spectra,^11−13^ and 2D UV-mass spectra^14,15^ have been shown to be able to differentiate carbohydrate isomers based on their ion mobility and spectral differences, but these methods usually require the carbohydrate standards to record the mobility drift times, the infrared spectra, or the UV spectra. Mass spectrometry, known for its high sensitivity, is widely used in carbohydrate structural determination.^16−22^ Single-stage mass spectrometry provides only compositional information, whereas tandem mass spectrometry is necessary for structural determination. In tandem mass spectrometry, carbohydrates are dissociated into fragments, and the carbohydrate structures are reconstructed from the structures of these fragments. Conventional tandem mass spectrometry typically determines the linkage positions of carbohydrates, but differentiating anomericities and stereoisomers remains challenging.

A new multistage tandem mass spectrometry method, based on the carbohydrate dissociation mechanisms in the gas phase, was developed for carbohydrate structural determination in our laboratory recently.^23−28^ In this method, precursor ions are dissociated into fragments through collision-induced dissociation (CID), and the structures of these fragments are then determined using mass spectra obtained by our specially designed CID sequences. Understanding the CID mechanisms is crucial in this method for deriving the structures of precursors and fragments.

Quantum chemistry calculations offer a method to understand how carbohydrates dissociate inside of the mass spectrometer. Since the 1990s, theoretical calculations have been carried out using semiempirical and Hartree–Fock methods to study the conformations and dissociation of disaccharides^29−32^ and oligosaccharides.^33,34^ Recently, more accurate methods, such as density functional theory (DFT), have been used to investigate monosaccharide and disaccharide CID.^35−46^ However, carbohydrates are floppy molecules, with the sugar ring adopting various puckering forms and the OH functional groups assuming different orientations. As a result, more than 200 stable conformers exist for a monosaccharide.^35,36,39−43^ When two monosaccharides are linked, the two dihedral angles of the glycosidic bonds change easily, resulting in over a million disaccharide conformers.^44−46^ However, some studies calculated only a handful of the most stable conformers, which may not accurately describe the dissociation mechanisms as the transition states (TS) with low dissociation barriers are not necessarily correlated to the most stable conformers. An efficient method to search for low-lying TSs among a large number of stable conformers is essential for understanding dissociation mechanisms.

We have developed an effective method for searching the low-lying TSs of monosaccharides and disaccharides attached to a sodium ion.^36,39−46^ Our previous computational studies focused on the monosaccharides and disaccharides in their ring forms.^36,39−46^ We have shown that the dehydration and ring-opening reactions at the C1 position are the two dissociation channels with low barrier heights in the ring form. After ring opening, carbohydrates adopt a linear form and subsequent C–C bond cleavage via the retro-aldol reaction occurs, resulting in cross-ring dissociation. The anomericity of the carbohydrates in their ring form can be determined by the branching ratios of the dehydration and cross-ring dissociation observed in the CID spectra. In our previous studies, the barrier for the ring-opening reaction was found to be higher than that for C–C bond cleavage; therefore, only the rate-determining step (i.e., the ring-opening reaction) of cross-ring dissociation was investigated in detail. In fact, different C–C bond cleavages lead to different cross-ring fragmentations. The CID spectra of previous studies showed that the relative intensities of these cross-ring fragments vary among different stereoisomers, but the mechanisms are not clear. Moreover, dehydration could possibly occur in a linear form in addition to the C–C bond cleavages. In this work, we focus on the reactions of carbohydrates after ring opening, i.e., reactions in linear forms. We investigate various C–C bond cleavages and dehydration reactions using quantum chemistry calculations and experimental measurements. We show that the branching ratios of different C–C bond cleavages can be explained by the differences between the barrier heights of C–C bond cleavage and the C=O migration. These distinct C–C bond cleavages can be used to differentiate the stereoisomers of glucose and galactose. The dehydration barriers in linear forms are very high, indicating that dehydration occurs only in the ring form, supporting our previous study. Applications for differentiating glucose and galactose in oligosaccharides are demonstrated.

## Experimental Method

The purity of the compounds was checked using high-performance liquid chromatography (HPLC). For HPLC-electrospray ionization (ESI)-MS experiments, 5 μL of the sample, dissolved in deionized water (DIW) at a concentration of 2 × 10^–4^ M, was injected into the HPLC system (Dionex Ultimate 3000, Thermo Fisher Scientific Inc., Waltham, MA, USA) with a Hypercarb column (100 × 2.1 mm, particle size: 3 μm, Thermo Fisher Scientific Inc.) for anomer separation. The eluents were then directly injected into the ESI source of a linear ion trap mass spectrometer (LTQ XL, Thermo Fisher Scientific Inc.). A NaCl solution (2 × 10^–4^ M NaCl dissolved in methanol:DIW = 50:50 solution (v/v%)) was added to the HPLC eluents before entering the ESI source. The mobile phase of the HPLC consisted of DI water (solution A) and HPLC-grade acetonitrile (solution B). The gradient of mobile phase was changed linearly from A = 100% and B = 0% at *t* = 0 min to *A* = 90% and *B* = 10% at *t* = 30 min, with a flow rate of 200 μL/min. The temperatures of the ESI source and transfer capillary were set to 280 and 350 °C, respectively. The voltages of the ion spray and capillary and tube lens were set to 4000 and 80 V, respectively. Helium gas was used as both the buffer gas and the collision gas in the ion trap. The linear ion trap settings were 1 u of isolation width, 30 ms of activation time, 30% of normalized collision energy, and 0.25 of the *Q* value in positive mode. For the direct injection of ESI emission to mass spectrometer, the analytes dissolved in the mixture of methanol/DIW (v/v% 50:50) solution in a concentration of 2 × 10^–4^ M with NaCl of 2 × 10^–4^ M were loaded in the emitter. The other experimental parameters were the same as those used for the HPLC-ESI-MS.

## Computational Method

**Figure 1:**
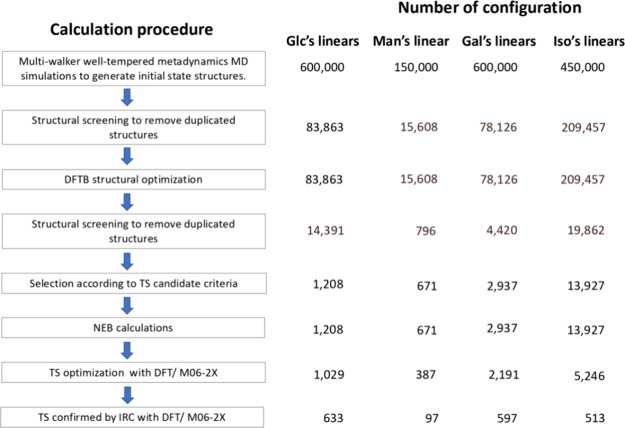
Calculation procedure and number of configurations involved in each stage of calculation.

Figure 1 illustrates the computational procedure and the number of structures involved in or obtained at each stage. The procedure begins with conformational search to identify as many low-energy conformers as possible. These conformers serve as initial reactants in the search for low-lying TSs for the reactions of interest. The conformational searches are conducted using molecular dynamics (MD) simulations to explore various conformers with Na^+^ complexes in a vacuum. For each complex, three multiwalkers, well-tempered metadynamics simulations are performed, with 10 walkers assigned to each MD simulation. Bias forces were introduced in the metadynamics MD simulations to facilitate sampling of broader conformational spaces, implemented through the use of collective variables. We used six collective variables: five dihedral angles formed between two neighboring O atoms and the coordination number of sodium ions to O atoms. The former five collective variables aided in sampling various linear structures with different dihedral angle combinations, while the latter collective variables explored different types of sodium ion binding to the O atoms. Three simulations with different rates of bias energy addition, 0.001, 0.002, and 0.01 hartree, yield a more diverse range of structures. The forces and the energies in the simulations were computed using tight-binding density functional theory (DFTB) method of GFN-xTB.^47^

The conformers resulting from the simulations were screened using the Ballester and Richards^48^ method. Conformers with similarity scores of >95% were grouped together, and only the conformer with the lowest energy in each group was selected for further calculations. These selected conformers were then geometrically optimized using the GFN-xTB method and were screened again using the same criteria. The resulting structures were analyzed to identify potential reactants leading to the low-lying TSs, based on the premise from our previous computational studies^36,46^ that the binding of a sodium ion to an O atom can weaken the O–H or O–C bonds of the associated O atom, thus reducing the TS energy. The TSs of C=O migration, C–C bond cleavage, and dehydration are the targets of our searches. The potential reactants leading to low-lying TSs that are prone to undergo C=O migration with a low reaction barrier are determined by the following two criteria. First, the distance between the O atom of the OH group next to the C=O group and the O atom of the C=O group (O2 and O1 for L1, O3 and O2 for L2, and O4 and O3 for L3) is less than 3 Å to promote H atom transfer. Second, the sodium ion binds to one of the aforementioned O atoms with a Na^+^–O distance of less than 2.5 Å.^36,39^ For C–C bond cleavage and dehydration, the first criterion changes to the distance between the O atom of the C=O group and the O atom of the OH group that is two C–C bonds away from the C=O group (e.g., O3 and O1 for L1, O4 and O2 for L2, and O5 and O3 for L3) being less than 3 Å for C–C bond cleavage, and the distance of any O–O of any two OH groups is less than 3 Å for dehydration. Meanwhile, the second criterion for selecting potential reactants remains the same.

After selecting the potential reactants, we performed climbing-image nudge-elastic band (NEB)^49^ calculations on the chosen candidates to obtain the reaction pathways as well as the TSs of the reactions. These calculations employed the GFN-xTB method, and the resulting TSs were used as initial guesses for more precise TS optimization via the DFT method. The DFT-optimized TSs were further verified through intrinsic reaction coordinate^50^ (IRC) calculations. All metadynamics simulations, GFN-xTB structure optimizations, and NEB calculations were carried out using CP2K software (version 8.1).^51^ The DFT calculations utilized Gaussian 16^52^ with the M06-2X density functional, and the basis set of 6-311+(d,p) was applied for the calculation of monosaccharides, while a slightly smaller basis set 6-31+(d,p) was applied to that of the disaccharides.

## Results and Discussion

**Figure 2:**
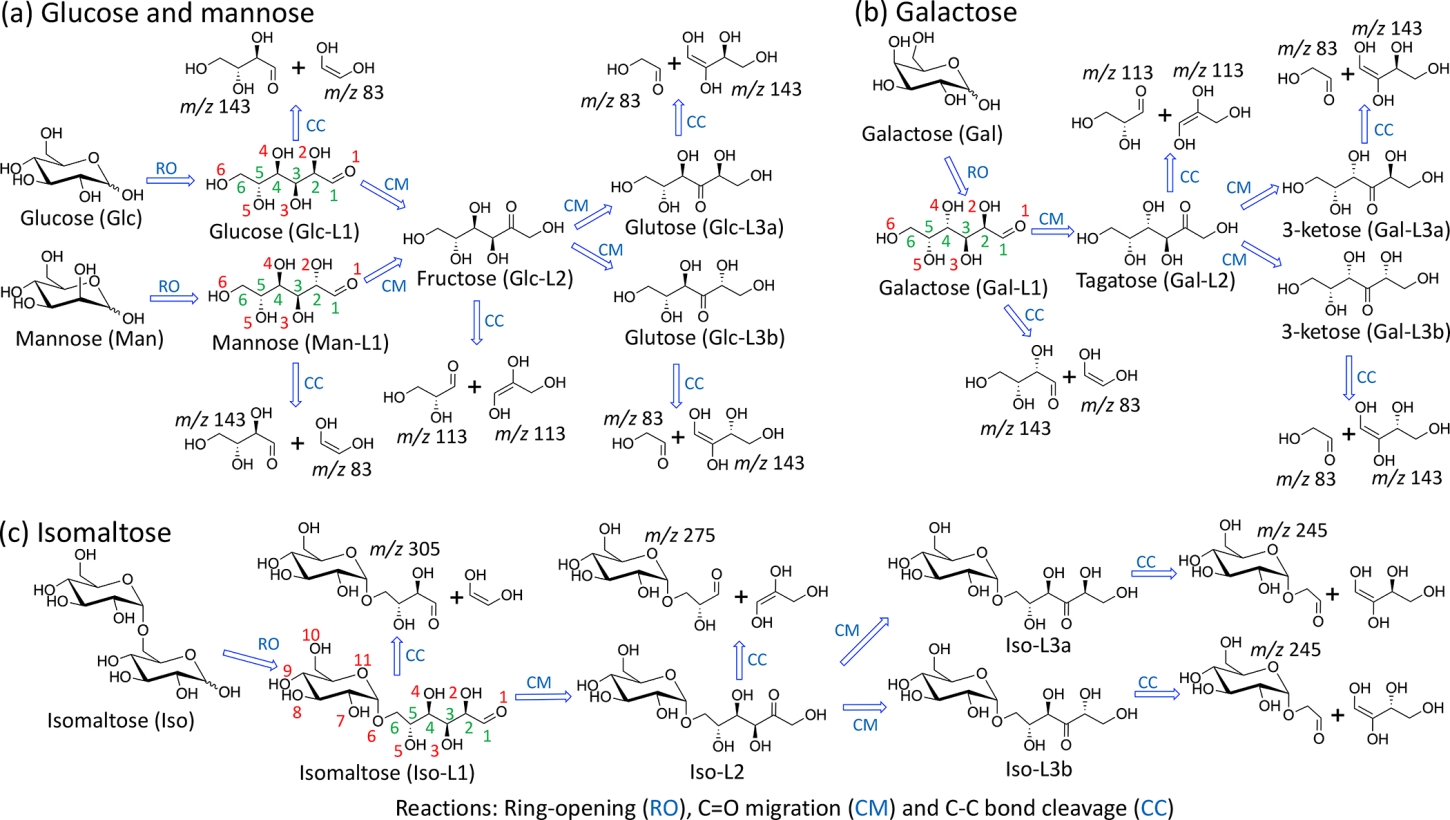
Structural changes from the ring form to various linear forms and fragmentation through various C–C bond cleavages. (a) Glucose and mannose, (b) galactose, and (c) isomaltose. The numberings of carbon and oxygen atoms are in green and red, respectively.

Most carbohydrates, including glucose, galactose, mannose, and isomaltose, studied in this work primarily exist in a ring form. During the CID process, some of them gain sufficient energy and change to a linear form through a ring-opening reaction. Carbohydrates in their linear form may undergo dissociation. Dehydration and C–C bond cleavage are the two major dissociation channels of carbohydrates observed in CID. In this work, we investigated the dehydration and C–C bond cleavage mechanisms of glucose, galactose, and mannose in their linear forms as well as the 1–6 linked disaccharides with the hexose at the reducing end in its linear form. The possible linear structures of these carbohydrates are illustrated in Figure 2, where L1 denotes the structure immediately after ring-opening reaction from the hexose in its ring forms. L2 is obtained from L1 by C=O migration from C1 to C2, while L3 is obtained from L2 by C=O migration from C2 to C3. There are two epimeric structures of L3, labeled as L3a and L3b.

### Monosaccharides

**Figure 3:**
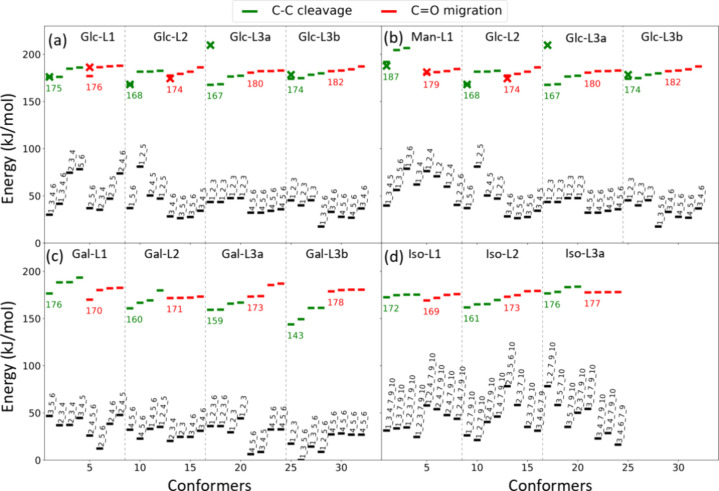
Calculated zero-point corrected energies of TSs and reactants using the DFT/M06-2X method: (a) glucose in linear form; (b) mannose in linear form; (c) galactose in linear form; (d) isomaltose with the glucose at the reducing end in linear form. Energies are relative to the energy of the global minimum structure of each monosaccharide in linear form. The green and red lines represent the TS energies of C–C bond cleavage and C=O migration, respectively. The crosses are the energies of the lowest TSs from our previous study^43^ reoptimized using the DFT/M06-2X method. The black lines right below each TS represent the energies of the reactant states leading to the TSs, where the numbers above the black line represent the numberings of the O atoms that bind to the sodium ion.

Figure 3 shows the relative zero-point corrected energy of the low-lying TSs and their corresponding reactant states for C–C bond cleavage and C=O migration, with the lowest energy of the most stable conformer of each monosaccharide in its linear form taken as the energy reference. Only the four lowest TSs are illustrated in Figure 3, and the full results are provided in the Supporting Information. For each monosaccharide in its linear form, it was found that the conformer with the C=O located at C3 is the most stable. The relevant lowest TS structures found in our previous study^41^ calculated using DFT/B3LYP and Møller–Plasset perturbation theory were reoptimized using the DFT/M06-2X method. These results are included in Figure 3 and are denoted by a cross sign. These TSs from previous work are generally similar to the lowest TSs identified in this study, except in the case of C–C bond cleavage of Glc-L3a where the current work found much lower reaction TSs.

**Figure 4:**
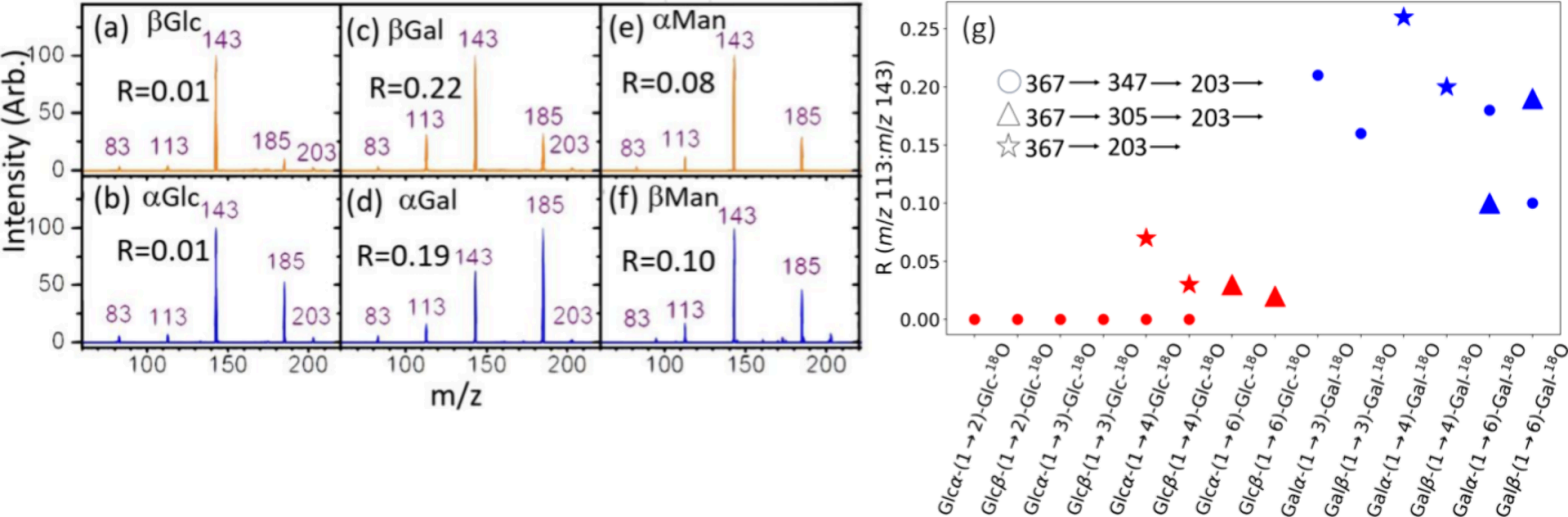
CID spectra of sodium ion adducts (*m*/*z* 203). (a) βGlucose, (b) αGlucose, (c) βGalactose, (d) αGalactose, (e) αMannose, and (f) βMannose. *R* represents the ratio of C3–C4 bond cleavage (fragment at *m*/*z* 113) to C2–C3 and C4–C5 bond cleavages (fragment at *m*/*z* 143). (g) *R* values of glucose (in red) and galactose (in blue) at the terminal nonreducing end produced from CID of ^18^O isotope labeled disaccharides, where ^18^O is labeled at the O1 atom of the sugar at the reducing end. Sequences of fragmentation are distinguished by different shapes (circle, triangle, and star).

The low-lying TSs for C–C bond cleavage and C=O migration of glucose and mannose in various linear structures are shown in Figure 3a and Figure 3b, respectively. For Glc-L1, the energies of the lowest TSs of C–C bond cleavage (which is C2–C3 bond cleavage, generating fragment *m*/*z* 143) and the C=O migration are similar (175 and 176 kJ/mol, respectively). In contrast, for Man-L1, the energy of the lowest TS of C–C bond cleavage (also C2–C3 bond cleavage, generating a fragment *m*/*z* 143) is 8 kJ/mol higher than that of C=O migration. This suggests that Man-L1 is more prone to undergo C=O migration to form Glc-L2 compared to Glc-L1. Both Glc-L1 and Man-L1 become Glc-L2 after the C=O migration from C1 to C2. For Glc-L2, the lowest TS for C–C bond cleavage (C3–C4 bond cleavage, generating fragment *m*/*z* 113) is lower than that for C=O migration by 6 kJ/mol, suggesting that Glc-L2 tends to undergo dissociation, rather than changing into different linear structures through C=O migration. If C=O migration occurs in Glc-L2, then Glc-L2 may revert to Glc-L1 or Man-L1, or it may become Glc-L3a or Glc-L3b. For both Glc-L3a and Glc-L3b, the lowest TS of C–C bond cleavage (C4–C5 bond cleavage) is lower than that of the lowest TS of C=O migration, indicating that Glc-L3a and Glc-L3b tend to dissociate into fragments. The C4–C5 bond cleavage of Glc-L3a and Glc-L3b also generates a fragment *m*/*z* 143. However, this makes only a minor contribution to fragment *m*/*z* 143, as most Glc-L2 undergoes C–C bond cleavage instead of converting into Glc-L3a and Glc-L3b. The differences in TS energies between C=O migration and C–C bond cleavage explain why the intensity ratio of fragment *m*/*z* 113 to fragment *m*/*z* 143 of mannose is higher than that for glucose in the CID spectra (Figure 4a,b,e,f).

Figure 3c shows the low-lying TSs for C–C bond cleavage and C=O migration of galactose in various linear structures. For Gal-L1, the energy of the lowest TS of C=O migration is 6 kJ/mol lower than that of C–C bond cleavage (which involves C2–C3 bond cleavage and generates a fragment at *m*/*z* 143). This suggests that, relative to Glc-L1, Gal-L1 has a lower tendency for C–C bond cleavage compared to C=O migration, similar to Man-L1. Meanwhile, Gal-L2 is found to have a significantly lower barrier of C–C bond cleavage (which involves C3–C4 bond cleavage and generates a fragment at *m*/*z* 113) compared with that of C=O migration, where the energy difference is 11 kJ/mol. This difference is nearly twice that of Glc-L2 (6 kJ/mol). For Gal-L3a and Gal-L3b, the lowest TS of C–C bond cleavage (which involves C4–C5 bond cleavage and generates a fragment at *m*/*z* 143) is significantly lower than that of C=O migration. However, most Gal-L2s do not convert to Gal-L3a or Gal-L3b, and the contributions of Gal-L3a and Gal-L3b to the fragment at *m*/*z* 143 are small. These TS energies suggest that the ratio of the fragment at *m*/*z* 113 to the fragment at *m*/*z* 143 for galactose must be larger than those for glucose and mannose. This is in agreement with the observation of the experimental results (Figure 4a–f).

The dehydration TSs for linear structures of glucose, mannose, and galactose were found to be approximately 80 kJ/mol higher than the lowest TSs for C–C bond cleavage or C=O migration. The significantly high barrier of dehydration in linear structures means that dehydration does not occur in linear forms; thus, the dehydrated product at *m*/*z* 185 observed in the CID spectra (Figure 4a–f) results entirely from dehydration in the ring form. Our previous computational studies^34,37^ suggested that the dehydration in ring form at C1 has a very low barrier, and most dehydration occurs at C1. This study further strengthens our previous conclusion.

**Figure 5:**
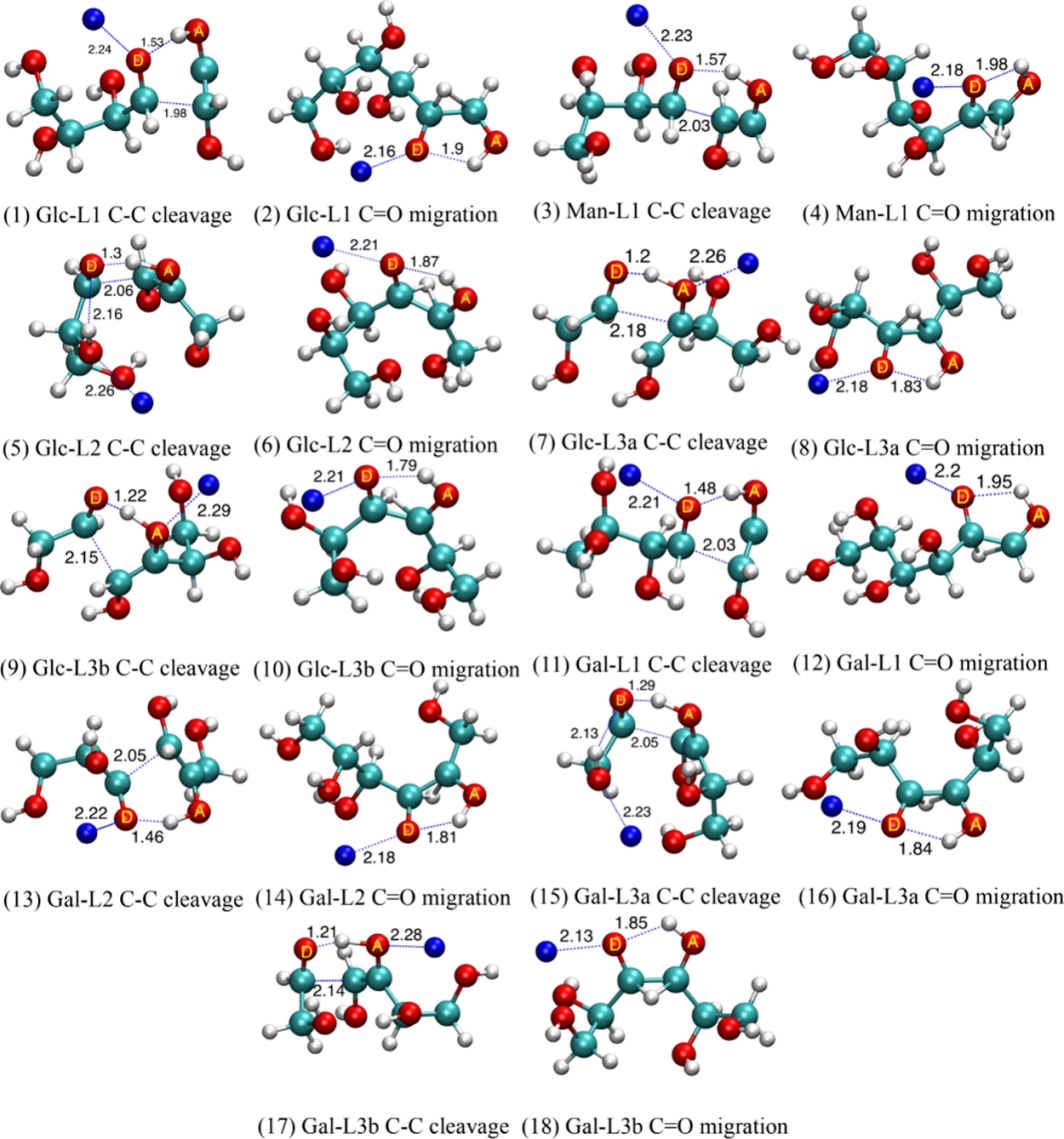
(1–18) Geometries of the lowest TSs of C–C bond cleavage and C=O migration in a series of linear structures. O atoms labeled as D and A are H atom donors and acceptors, respectively. Cyan, red, white, and blue spheres represent carbon, oxygen, hydrogen, and sodium atoms, respectively. The distances between atoms (in angstroms) indicating bond braking and sodium ion binding are labeled.

The structures of the lowest TS for each reaction in Figure 3 are illustrated in Figure 5. The structures of the lowest TSs indicate that the sodium ion mostly binds to the O atom that donates the H atom. In a few cases, such as C–C bond cleavage of Glc-L3a, Glc-L3b, and Gal-L3b [Figure 5 (7), (9), and (17), respectively], sodium ion binds to the acceptor. Although the starting configurations of the TS searches satisfy the criteria discussed in the Computational Method section, the optimization may end up with the low-lying TS in which the sodium ion does not bind to either the O atoms in which H atom transfer takes place, as in the case of C–C bond cleavage of Glc-L2 [Figure 5 (5)] and C–C bond cleavage of Gal-L3a [Figure 5 (15)]. The structures of the dehydration TSs are provided in the Supporting Information.

The differences in the intensity ratio of the fragment at *m*/*z* 113 to the fragment at *m*/*z* 143 observed in monosaccharides were also found in the monosaccharides produced from the CID of the disaccharides. Figure 4g shows the intensity ratios of the fragment at *m*/*z* 113 to the fragment at *m*/*z* 143 for glucose and galactose produced through various CID sequences: (1) 367 (sodium ion adduct of disaccharides) → 347 (fragment from dehydration at the reducing end) → 203 (monosaccharide at the nonreducing end) →, (2) 367 → 305 (fragment from cross-ring dissociation at the reducing end) → 203 (monosaccharide at the nonreducing end) →, and (3) 367 (sodium ion adduct of disaccharides with O1 at the reducing end labeled by ^18^O) → 203 (monosaccharide at the nonreducing end) →. It is clear that the ratios of galactose are higher than those of glucose, although the ratios vary with the CID sequences to generate the monosaccharide.

In summary, the transition state energies from calculations of linear glucose, mannose, and galactose predict the ratios of C3–C4 bond cleavage to C2–C3 bond cleavage increase from glucose to mannose and then to galactose. The trend was confirmed by the CID spectra of monosaccharides and monosaccharides produced from the dissociation of disaccharides. The large difference between the ratios of glucose and galactose is useful for the differentiation of these two stereoisomers.

### 1–6 Linked Disaccharides

**Figure 6:**
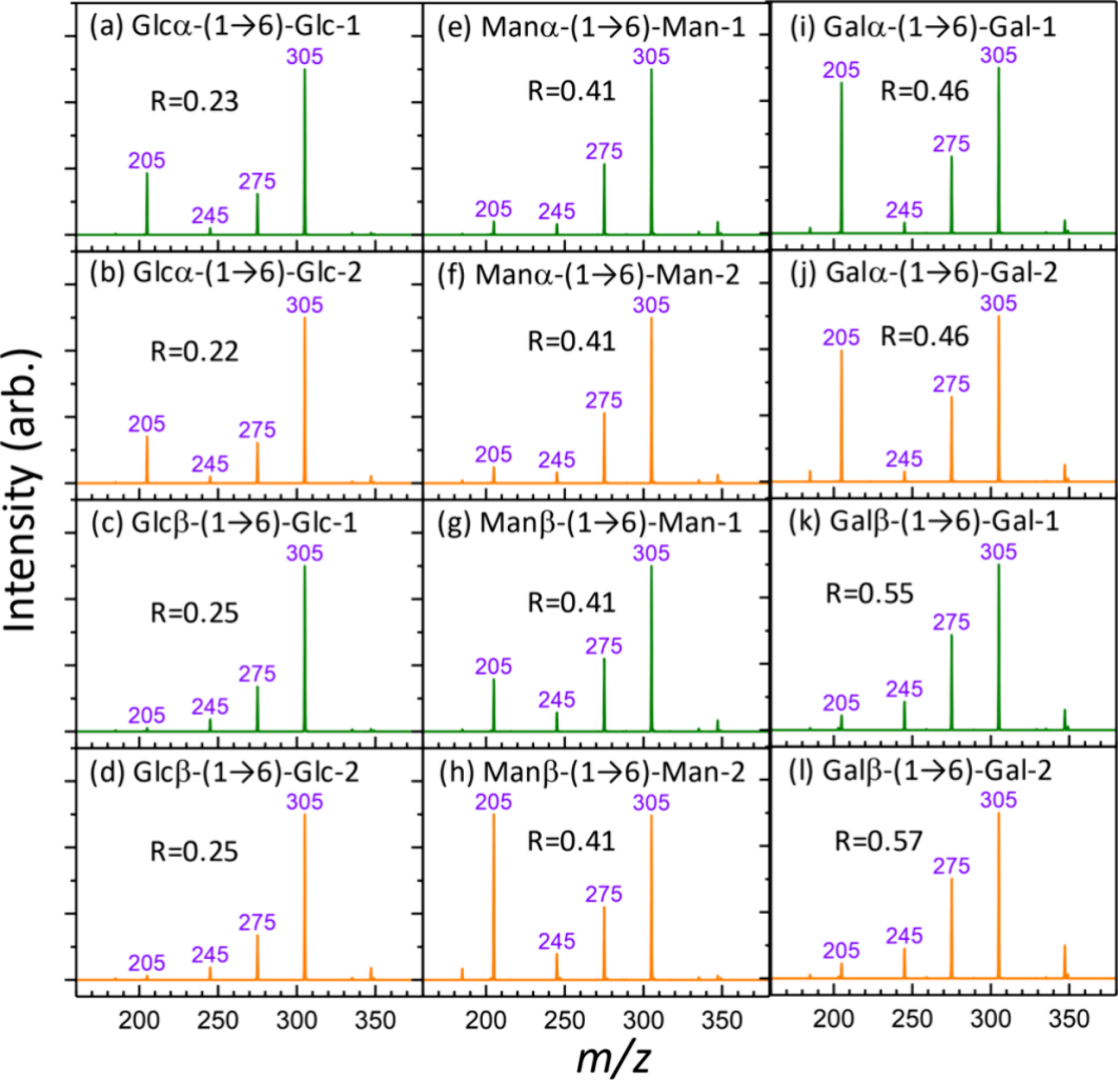
CID spectra of 1–6 linked disaccharide sodium ion adducts (*m*/*z* 365). (a–d) Glucose disaccharides, (e–h) mannose disaccharides, and (i–l) galactose disaccharides. Glcα-(1 → 6)-Glc-1 and Glcα-(1 → 6)-Glc-2 represent two anomers of Glcα-(1 → 6)-Glc (α or β of the glucose at the reducing end) separated by HPLC. Similar notations are used for the other disaccharides. *R* represents the intensity ratio of the fragment at *m*/*z* 275 to the fragment at *m*/*z* 305.

Figure 3d shows the TS energies for isomaltose with the glucose at the reducing end in its linear form. The structures of the TSs are provided in the Supporting Information. Unlike glucose in Glc-L1, where the lowest energy TS of C–C bond cleavage is just slightly lower than that of C=O migration, the lowest energy TS of C–C bond cleavage (which is C2–C3 bond cleavage) of Iso-L1 is higher than that of C=O migration. The C2–C3 bond cleavage of isomaltose generates a fragment at *m*/*z* 305 (loss of neutral *m* = 60), which is analogous to glucose Glc-L1 producing a fragment at *m*/*z* 143 (loss of neutral *m* = 60). The lowest TS for C–C bond cleavage of Iso-L2 (which involves C3–C4 bond cleavage) is 12 kJ/mol lower than that for C=O migration, which is significantly lower than that for the analogous reaction in Glc-L2 (6 kJ/mol). The C3–C4 bond cleavage of isomaltose generates a fragment at *m*/*z* 275 (loss of neutral *m* = 90), which is analogous to glucose Glc-L2, producing a fragment at *m*/*z* 113 (loss of neutral *m* = 90). These TS energies suggest that the presence of a monosaccharide at C6 enhances C=O migration from O1 to O2 and reduces the TS energy for C3–C4 bond cleavage. Consequently, the ratio of the fragment at *m*/*z* 275 to the fragment at *m*/*z* 305 in isomaltose must be larger than the ratio of the fragment at *m*/*z* 113 to the fragment at 143 in glucose. This is in agreement with the experimental observations (Figure 6). The ratio of C3–C4 bond cleavage to C2–C3 bond cleavage increases from glucose (0.01–0.07) to isomaltose (0.22–0.25). Only a small fraction of Iso-L2 converts to Iso-L3a and Iso-L3b, where the lowest TSs for C–C bond cleavage (C4–C5 bond) and C=O migration are similar to each other, suggesting an equal preference for the two reactions. Interestingly, the C2–C3 and C4–C5 bond cleavages of glucose produce ions with the same *m*/*z* value (*m*/*z* 143), which cannot be distinguished in the mass spectrum. In contrast, the C2–C3 and C4–C5 bond cleavages of isomaltose produce ions at *m*/*z* 305 and *m*/*z* 245, respectively. Using these distinct *m*/*z* values, the minor contribution of C4–C5 bond cleavage from Iso-L3 can be found in the CID spectra (Figure 6). The effect of a monosaccharide at the C6 position is also observed with different stereoisomers, such as mannose disaccharides (Figure 6e–h) and galactose disaccharides (Figure 6i–l), where the ratios of C3–C4 bond cleavage to C2–C3 bond cleavage increase from mannose (0.08–0.10) and galactose (0.09–0.26) to mannose disaccharides (0.41) and galactose disaccharides (0.46–0.57).

**Figure 7:**
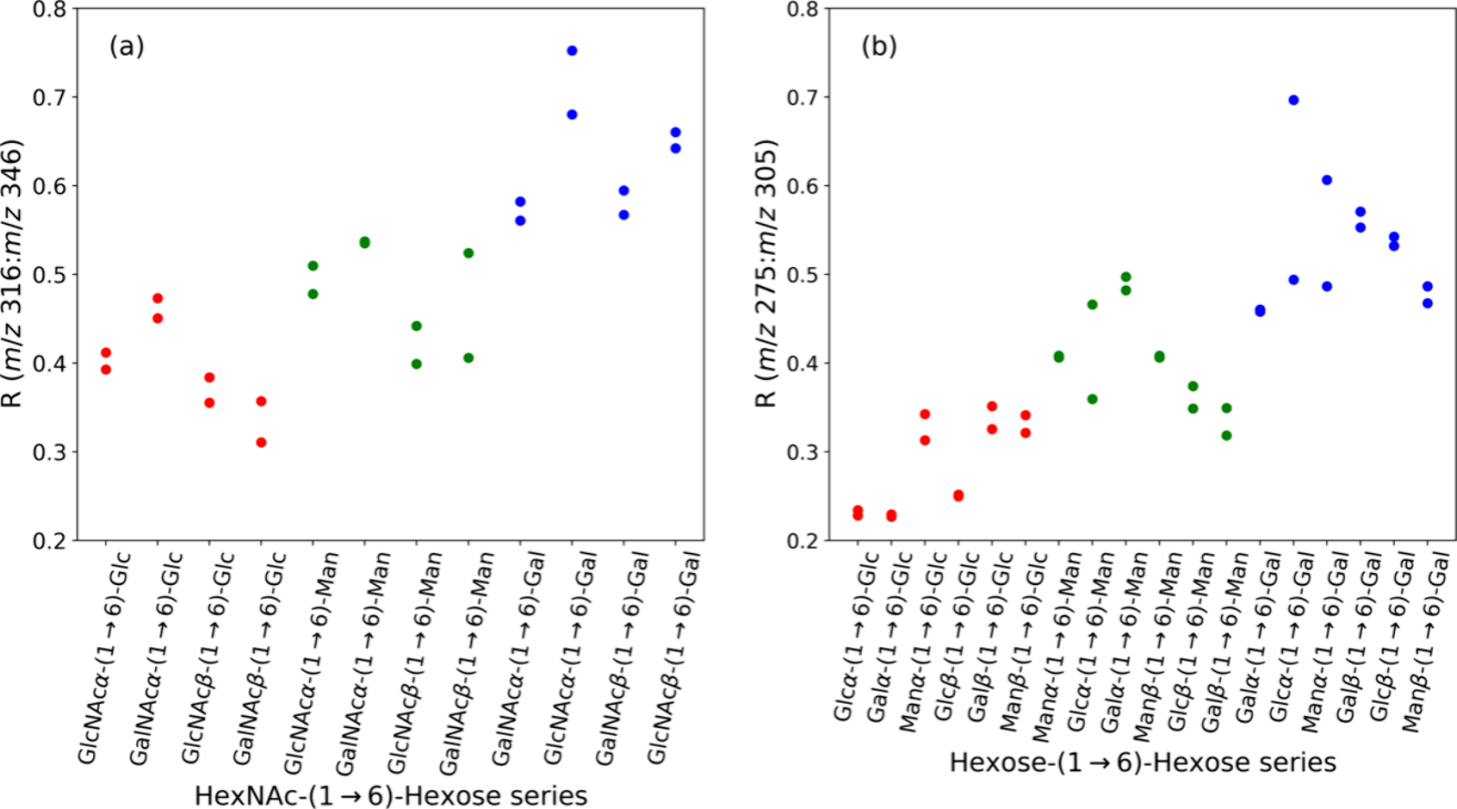
Ratios of C2–C3 bond cleavage to C3–C4 bond cleavage for various 1–6 linked disaccharides. (a) HexNAc-(1 → 6)-Hex through CID sequence: 406 (sodium ion adduct) →. (b) Hex-(1 → 6)-Hex through CID sequence: 365 (sodium ion adduct) →.

The increase in the ratios from monosaccharides to 1–6 linked disaccharides was also observed in different disaccharides with glucose, mannose, or galactose at the reducing end. Figure 7a and Figure 7b show such a comparison for disaccharides with the nonreducing end as *N*-acetylhexosamine (HexNAc) and Hex (hexose), respectively. The trend of increasing ratios from glucose to mannose and then to galactose was also observed in these disaccharides. Notably, the ratios for disaccharides with HexNAc at the nonreducing end span from 0.31 to 0.76, while the ratios for disaccharides with Hex at the nonreducing end span from 0.22 to 0.70. The distinctions between glucose and galactose are well separated, but the ratios of glucose and galactose relative to mannose are not clear-cut.

In summary, a similar trend that ratios of C3–C4 bond cleavage to C2–C3 bond cleavage increase from the glucose to mannose and then to galactose was found in the disaccharides with 1 → 6 linkage and can be used in the differentiation of glucose and galactose at the reducing end of disaccharides.

### Applications to Oligosaccharides

**Figure 8:**
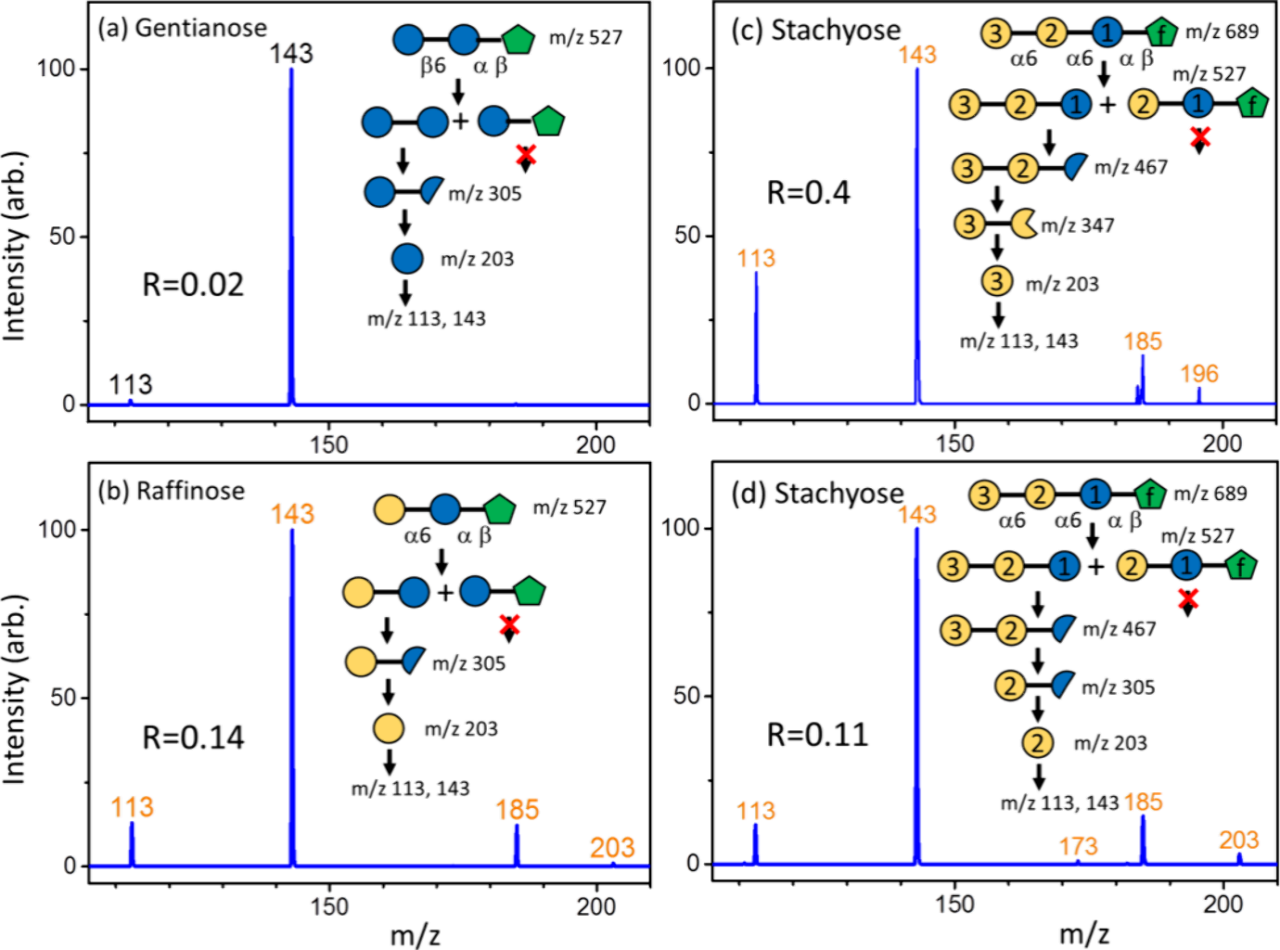
CID spectra of glucose and galactose produced from the CID of oligosaccharides and the structure changes during the CID process: (a) gentianose, (b) raffinose, and (c, d) stachyose. Blue and yellow circles represent glucose and galactose, respectively, while green pentagons represent fructose. Half circles and three-quarter circle represent cross-ring dissociation and dehydration of hexose, respectively. The *R* value represents the intensity ratio of the fragment at *m*/*z* 113 to the fragment at *m*/*z* 143.

The identification of stereoisomers in oligosaccharides using mass spectrometry is challenging. Here, we apply distinct ratios between glucose and galactose to identify the stereoisomers (glucose or galactose) in oligosaccharides. Figure 8 presents the CID spectra of glucose and galactose obtained from the sequential CID of oligosaccharides. Notably, the specially designed CID sequences, based on our new mass spectrometry method (logically derived sequence multistage tandem mass spectrometry),^21−26^ have been applied to obtain the glucose or galactose at specific positions of oligosaccharides. In brief, these CID sequences are designed based on the following dissociation mechanisms: (1) the dehydration and cross-ring dissociation in the CID of oligosaccharide sodium ion adducts mainly occur at the reducing end due to the low dissociation barriers, and (2) glycosidic bond cleavage occurs at any position. Details regarding how to design the CID sequences were reported in our previous reports.^21−26^ The results in Figure 8 demonstrate that glucose and galactose are correctly identified using the intensity ratio of the fragment at *m*/*z* 113 to the fragment at *m*/*z* 143, by comparison with the ratios in Figure 4.

**Figure 9:**
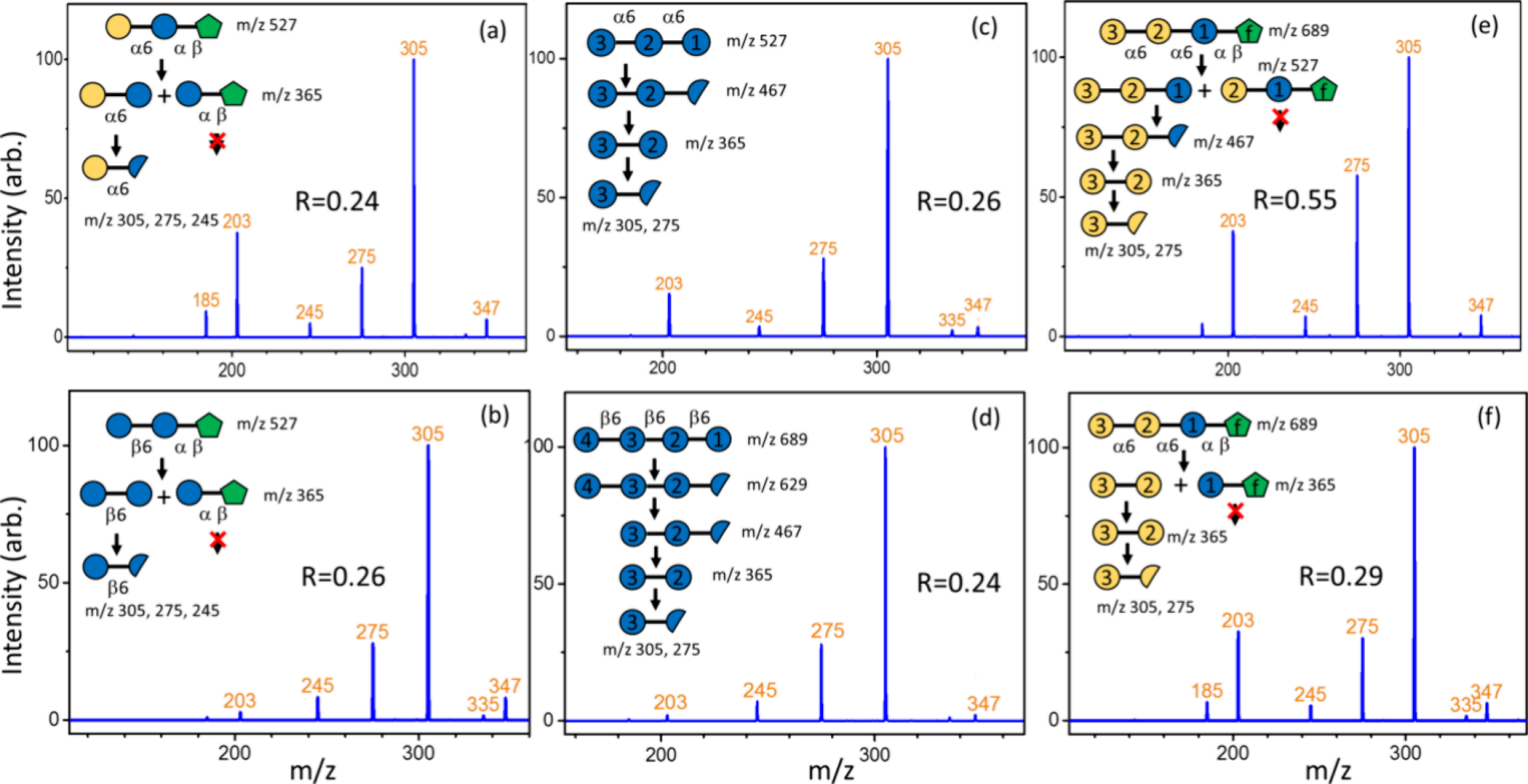
CID spectra of Hex-(1 → 6)-Hex disaccharides produced from CID of oligosaccharides and the structure changes during the CID process. (a) Raffinose, (b) gentianose, (c) isomaltriose, (d) Glc-β-(1-6)-Glc-β-(1-6)-Glc-β-(1-6)-Glc, and (e, f) stachyose. The *R* value represents the intensity ratio of the fragment at *m*/*z* 275 to the fragment at *m*/*z* 305 in the CID spectra. Comparison of panels (e, f) shows that the CID sequence of panel (e) generates the disaccharide consisting of sugars 2 and 3 for structural determination, while the CID sequence in panel (f) is interfered by the secondary dissociation (see details in the text).

We also applied the intensity ratios of the fragment at *m*/*z* 275 to the fragment at *m*/*z* 305, produced from the CID of Hex-(1 → 6)-Hex disaccharides, to differentiate glucose and galactose. Figure 9 presents the CID spectra of Hex-(1 → 6)-Hex produced from various oligosaccharides. Similar to the monosaccharides, these specific disaccharides are obtained through specially designed CID sequences. Comparing the intensity ratios of the fragment at *m*/*z* 275 to the fragment at *m*/*z* 305 in Figure 9 with the ratios in Figure 7 shows that most of the CID spectra correctly assign the glucose or galactose at the reducing end of Hex-(1 → 6)-Hex disaccharides, except for the CID spectrum in Figure 9f. Indeed, Figure 9e,f shows the CID spectra of the same disaccharides, i.e., Galα-(1 → 6)-Galα, although they are generated through different CID sequences. The low *R* value obtained from the CID spectrum in Figure 9f may result from the disaccharide Galα-(1 → 6)-Glcα produced through secondary dissociation during the CID sequence. In the first step of CID in Figure 9f, 689 → 365, only two disaccharides, Galα-(1 → 6)-Galα and Glcα-(1 ↔ 2)-Fruβ, are produced. However, it is possible that the disaccharide Galα-(1 → 6)-Glcα is produced through secondary dissociation, i.e., the precursor ion at *m*/*z* 689 is resonance-excited and undergoes CID to produce two trisaccharides: Galα-(1 → 6)-Galα-(1 → 6)-Glcα and Galα-(1 → 6)-Glcα-(1 ↔ 2)-Fruβ. These two trisaccharides have large enough energy left to undergo dissociation without resonance excitation, generating the disaccharide Galα-(1 → 6)-Glcα. Although the CID sequence in Figure 9e requires one more step compared with that in Figure 9f, it avoids the potential secondary dissociation and predicts the stereoisomer correctly. This example shows the importance of the CID sequence to obtain the desired disaccharides.

Compared to the method used in our previous reports for differentiating stereoisomers (glucose, galactose, and mannose) using the entire CID spectra of lithium ion adducts after dehydration,^23,25^ this method uses the CID spectra of sodium ion adducts, which is one CID step less than that of lithium ion adducts in multistage tandem mass spectrometry. However, this method is limited to the differentiation of glucose and galactose. Furthermore, if the ratio is near the boundary, then it is better to cross-check the stereoisomers using alternative CID sequences or by using lithium ion adducts.

## Conclusions

The transition state energies from calculations for linear glucose, mannose, and galactose suggest that the ratios of C3–C4 bond cleavage to C2–C3 bond cleavage increase progressively from glucose to mannose and then to galactose. The substantial difference between the ratios of glucose and galactose can thus be used to differentiate these two stereoisomers. The same trend was similarly observed in the calculations of the hexose at the reducing end of disaccharides with 1 → 6 linkage. These calculations were validated by experimental measurements. Although the calculations were conducted only for monosaccharides and disaccharides, they provide valuable insights into carbohydrate dissociation mechanisms in mass spectrometry (i.e., in the gas phase). The dissociation mechanisms are the foundation of our method, LODES/MS^*n*^, for determining oligosaccharide structures. We demonstrated the applications of these mechanisms and LODES/MS^*n*^ to determine the stereoisomers of monosaccharide compositions in trisaccharides and tetrasaccharides. Unlike other tandem mass spectrometry methods, which either provide only linkage position information or require oligosaccharide standards to record mass spectra as fingerprints, LODES/MS^*n*^ enables the determination of linkage positions, anomericities, and stereoisomers of oligosaccharides without using oligosaccharide standards,^27^ making LODES/MS^*n*^ particularly useful for discovering new oligosaccharides.^26,53^ The mechanisms studied in this work offer an alternative approach to LODES/MS^*n*^ for stereoisomer identification, enhancing the capability of LODES/MS^*n*^ for oligosaccharide structural determination.

## Supporting Information Available

Calculated energies of reactants and transition states of C–C bond cleavage, C=O migration, and dehydration of glucose, mannose, and galactose in linear forms and isomaltose with glucose at the reducing end in linear form; geometries of the lowest TSs for isomaltose with the glucose at the reducing end in linear forms; geometries of the global minimum and lowest dehydration TSs of linear glucose, mannose, and galactose; and files of *XYZ* coordinates for reactants and transition states (PDF)

## References

[ref1] Varki A.; Kornfeld S. Historical Background and Overview. In Essentials of Glycobiology 3 *rd ed.*, Varki A.; Cummings R. D.; Esko J. D.; Stanley P.; Hart G. W.; Aebi M.; Mohnen D.; Kinoshita T.; Packer N. H.; Prestegard J. H. Eds.; Cold Spring Harbor Laboratory Press: Cold Spring Harbor (NY), 2017; pp 1–18.

[ref2] Kamerling J. P. Basics Concepts and Nomenclature Recommendations in Carbohydrate Chemistry. In Comprehensive Glycoscience from Chemistry to Systems Biology, Kamerling J. P., Ed.; Elsevier Science Ltd.: 2007, Vol. 1, pp 1–37.

[ref3] Duus J. Ø.; Gotfredsen C. H.; Bock K. Carbohydrate Structural Determination by Nmr Spectroscopy: Modern Methods and Limitations. Chem. Rev. 2000, 100 (12), 4589–4614. 10.1021/cr990302n. 11749359

[ref4] Battistel M. D.; Azurmendi H. F.; Yu B.; Freedberg D. I. Nmr of Glycans: Shedding New Light on Old Problems. Prog. Nucl. Magn. Reson. Spectrosc. 2014, 79, 48–68. 10.1016/j.pnmrs.2014.01.001. 24815364

[ref5] Kamerling J. P.; Gerwig G. J. Strategies for the Structural Analysis of Carbohydrates. In Comprehensive Glycoscience, Kamerling J. P.; Boons J.; Lee Y. C.; Suzuki A.; Taniguchi N.; Voragen A. G. J., Eds.; Elsevier Science Ltd.: 2007, Vol. 2, pp 1–67.

[ref6] Jacob G. S.; Scudder P. [17] Glycosidases in Structural Analysis. In Methods in Enzymology; Academic Press: 1994, Vol. 230, pp 280–299. 8139502 10.1016/0076-6879(94)30019-4

[ref7] Prime S.; Dearnley J.; Ventom A. M.; Parekh R. B.; Edge C. J. Oligosaccharide Sequencing Based on Exo- and Endoglycosidase Digestion and Liquid Chromatographic Analysis of the Products. Journal of Chromatography A 1996, 720 (1), 263–274. 10.1016/0021-9673(95)00029-1. 8601195

[ref8] Pagel K.; Harvey D. J. Ion Mobility–Mass Spectrometry of Complex Carbohydrates: Collision Cross Sections of Sodiated N-Linked Glycans. Anal. Chem. 2013, 85 (10), 5138–5145. 10.1021/ac400403d. 23621517

[ref9] Both P.; Green A. P.; Gray C. J.; Šardzík R.; Voglmeir J.; Fontana C.; Austeri M.; Rejzek M.; Richardson D.; Field R. A.; et al. Discrimination of Epimeric Glycans and Glycopeptides Using Im-Ms and Its Potential for Carbohydrate Sequencing. Nat. Chem. 2014, 6 (1), 65–74. 10.1038/nchem.1817. 24345949

[ref10] Li L.; McKenna K. R.; Li Z.; Yadav M.; Krishnamurthy R.; Liotta C. L.; Fernández F. M. Rapid Resolution of Carbohydrate Isomers Via Multi-Site Derivatization Ion Mobility-Mass Spectrometry. Analyst 2018, 143 (4), 949–955. 10.1039/C7AN01796K. 29367994

[ref11] Gray C. J.; Schindler B.; Migas L. G.; Pičmanová M.; Allouche A. R.; Green A. P.; Mandal S.; Motawia M. S.; Sánchez-Pérez R.; Bjarnholt N.; et al. Bottom-up Elucidation of Glycosidic Bond Stereochemistry. Anal. Chem. 2017, 89 (8), 4540–4549. 10.1021/acs.analchem.6b04998. 28350444

[ref12] Bansal P.; Yatsyna V.; AbiKhodr A. H.; Warnke S.; Ben Faleh A.; Yalovenko N.; Wysocki V. H.; Rizzo T. R. Using Slim-Based Ims-Ims Together with Cryogenic Infrared Spectroscopy for Glycan Analysis. Anal. Chem. 2020, 92 (13), 9079–9085. 10.1021/acs.analchem.0c01265. 32456419 PMC7349563

[ref13] Greis K.; Kirschbaum C.; von Helden G.; Pagel K. Gas-Phase Infrared Spectroscopy of Glycans and Glycoconjugates. Curr. Opin. Struct. Biol. 2022, 72, 194–202. 10.1016/j.sbi.2021.11.006. 34952241

[ref14] Saparbaev E.; Kopysov V.; Aladinskaia V.; Ferrieres V.; Legentil L.; Boyarkin O. V. Identification and quantification of qny isoforms of carbohydrates by 2D UV-MS fingerprinting of cold Ions. Anal. Chem. 2020, 92, 14624–14632. 10.1021/acs.analchem.0c03122. 33138380

[ref15] Saparbaev E.; Kopysov V.; Yamaletdinov R.; Pereverzev A. Y.; Boyarkin O. V. Interplay of H-bonds with aromatics in isolated complexes identifies isomeric carbohydrates. Angew. Chem. 2019, 131, 7424–7428. 10.1002/ange.201902377. 30924999

[ref16] Haslam S. M.; Freedberg D. I.; Mulloy B.; Dell A.; Stanley P.; Prestegard J. H. Structural Analysis of Glycans. In Essentials of Glycobiology [Internet], 4 *th ed.*, Varki A.; Cummings R. D.; Esko J. D.; Stanley P.; Hart G. W.; Aebi M.; Mohnen D.; Kinoshita T.; Packer N. H.; Prestegard J. H.Eds.; Cold Spring Harbor Laboratory Press: Cold Spring Harbor (NY), 2022.

[ref17] Zaia J. Mass Spectrometry of Oligosaccharides. Mass Spectrom. Rev. 2004, 23 (3), 161–227. 10.1002/mas.10073. 14966796

[ref18] Wei J.; Papanastasiou D.; Kosmopoulou M.; Smyrnakis A.; Hong P.; Tursumamat N.; Klein J. A.; Xia C.; Tang Y.; Zaia J.; et al. De Novo Glycan Sequencing by Electronic Excitation Dissociation Ms2-Guided Ms3 Analysis on an Omnitrap-Orbitrap Hybrid Instrument. Chemical Science 2023, 14 (24), 6695–6704. 10.1039/D3SC00870C. 37350811 PMC10284134

[ref19] Harvey D. J. Fragmentation of Negative Ions from Carbohydrates: Part 1. Use of Nitrate and Other Anionic Adducts for the Production of Negative Ion Electrospray Spectra from N-Linked Carbohydrates. J. Am. Soc. Mass Spectrom. 2005, 16 (5), 622–630. 10.1016/j.jasms.2005.01.004. 15862764

[ref20] Dong X.; Zhou S.; Mechref Y. Lc-Ms/Ms Analysis of Permethylated Free Oligosaccharides and N-Glycans Derived from Human, Bovine, and Goat Milk Samples. ELECTROPHORESIS 2016, 37 (11), 1532–1548. 10.1002/elps.201500561. 26959529 PMC4963982

[ref21] Nwosu C. C.; Aldredge D. L.; Lee H.; Lerno L. A.; Zivkovic A. M.; German J. B.; Lebrilla C. B. Comparison of the Human and Bovine Milk N-Glycome Via High-Performance Microfluidic Chip Liquid Chromatography and Tandem Mass Spectrometry. J. Proteome Res. 2012, 11 (5), 2912–2924. 10.1021/pr300008u. 22439776 PMC3345083

[ref22] Ashline D.; Singh S.; Hanneman A.; Reinhold V. Congruent Strategies for Carbohydrate Sequencing. 1. Mining Structural Details by Msn. Anal. Chem. 2005, 77 (19), 6250–6262. 10.1021/ac050724z. 16194086 PMC1435741

[ref23] Hsu H. C.; Liew C. Y.; Huang S.-P.; Tsai S.-T.; Ni C.-K. Simple Approach for De Novo Structural Identification of Mannose Trisaccharides. J. Am. Soc. Mass Spectrom. 2018, 29 (3), 470–480. 10.1007/s13361-017-1850-5. 29235038

[ref24] Hsu H. C.; Liew C. Y.; Huang S.-P.; Tsai S.-T.; Ni C.-K. Simple Method for De Novo Structural Determination of Underivatised Glucose Oligosaccharides. Sci. Rep. 2018, 8 (1), 5562 10.1038/s41598-018-23903-4. 29615745 PMC5882935

[ref25] Tsai S.-T.; Liew C. Y.; Hsu C.; Huang S.-P.; Weng W.-C.; Kuo Y.-H.; Ni C.-K. Automatic Full Glycan Structural Determination through Logically Derived Sequence Tandem Mass Spectrometry. Chembiochem 2019, 20 (18), 2351–2359. 10.1002/cbic.201900228. 31016827

[ref26] Liew C. Y.; Luo H.-S.; Yang T.-Y. Y.; Hung A.-T.; Magoling B. J. A.; Lai C. P.-K.; Ni C.-K. Dentification of the High Mannose N-Glycan Isomers Undescribed by Conventional Multicellular Eukaryotic Biosynthetic Pathways. Anal. Chem. 2023, 95 (23), 8789–8797. 10.1021/acs.analchem.2c05599. 37235553 PMC10267891

[ref27] Liew C. Y.; Chan C.-K.; Huang S.-P.; Cheng Y.-T.; Tsai S.-T.; Hsu H. C.; Wang C.-C.; Ni C.-K. De Novo Structural Determination of Oligosaccharide Isomers in Glycosphingolipids Using Logically Derived Sequence Tandem Mass Spectrometry. Analyst 2021, 146 (23), 7345–7357. 10.1039/D1AN01448J. 34766961

[ref28] Ni C.-K.; Hsu H. C.; Liew C. Y.; Huang S.-P.; Tsai S.-T. 1.13 - Modern Mass Spectrometry Techniques for Oligosaccharide Structure Determination: Logically Derived Sequence Tandem Mass Spectrometry for Automatic Oligosaccharide Structural Determination. In Comprehensive Glycoscience *(*Second *Edition)*, Barchi J. J., Ed.; Elsevier: 2021, pp 309–339.

[ref29] Hofmeister G. E.; Zhou Z.; Leary J. A. Linkage Position Determination in Lithium-Cationized Disaccharides: Tandem Mass Spectrometry and Semiempirical Calculations. J. Am. Chem. Soc. 1991, 113 (16), 5964–5970. 10.1021/ja00016a007.

[ref30] Staempfli A.; Zhou Z.; Leary J. A. Gas-Phase Dissociation Mechanisms of Dilithiated Disaccharides: Tandem Mass Spectrometry and Semiempirical Calculations. J. Org. Chem. 1992, 57 (13), 3590–3594. 10.1021/jo00039a016.

[ref31] Mulroney B.; Barrie Peel J.; Traeger J. C. Theoretical Study of Deprotonated Glucopyranosyl Disaccharide Fragmentation. J. Mass Spectrom. 1999, 34 (8), 856–871. 10.1002/(SICI)1096-9888(199908)34:8<856::AID-JMS841>3.0.CO;2-8. 10423567

[ref32] Suzuki H.; Kameyama A.; Tachibana K.; Narimatsu H.; Fukui K. Computationally and Experimentally Derived General Rules for Fragmentation of Various Glycosyl Bonds in Sodium Adduct Oligosaccharides. Anal. Chem. 2009, 81 (3), 1108–1120. 10.1021/ac802230a. 19117495

[ref33] Fukui K.; Kameyama A.; Mukai Y.; Takahashi K.; Ikeda N.; Akiyama Y.; Narimatsu H. A Computational Study of Structure–Reactivity Relationships in Na-Adduct Oligosaccharides in Collision-Induced Dissociation Reactions. Carbohydr. Res. 2006, 341 (5), 624–633. 10.1016/j.carres.2006.01.013. 16442513

[ref34] Suzuki H.; Yamagaki T.; Tachibana K.; Fukui K. Fragmentation of Lewis-Type Trisaccharides in the Gas Phase: Experimental and Theoretical Studies. Int. J. Mass Spectrom. 2008, 278 (1), 1–9. 10.1016/j.ijms.2008.07.012.

[ref35] Satoh H.; Oda T.; Nakakoji K.; Uno T.; Tanaka H.; Iwata S.; Ohno K. Potential Energy Surface-Based Automatic Deduction of Conformational Transition Networks and Its Application on Quantum Mechanical Landscapes of D-Glucose Conformers. J. Chem. Theory Comput. 2016, 12 (11), 5293–5308. 10.1021/acs.jctc.6b00439. 27673598

[ref36] Chen J.-L.; Nguan H. S.; Hsu P.-J.; Tsai S.-T.; Liew C. Y.; Kuo J.-L.; Hu W.-P.; Ni C.-K. Collision-Induced Dissociation of Sodiated Glucose and Identification of Anomeric Configuration. Phys. Chem. Chem. Phys. 2017, 19 (23), 15454–15462. 10.1039/C7CP02393F. 28580968

[ref37] Bythell B. J.; Abutokaikah M. T.; Wagoner A. R.; Guan S.; Rabus J. M. Cationized Carbohydrate Gas-Phase Fragmentation Chemistry. J. Am. Soc. Mass Spectrom. 2017, 28 (4), 688–703. 10.1007/s13361-016-1530-x. 27896699

[ref38] Rabus J. M.; Abutokaikah M. T.; Ross R. T.; Bythell B. J. Sodium-Cationized Carbohydrate Gas-Phase Fragmentation Chemistry: Influence of Glycosidic Linkage Position. Phys. Chem. Chem. Phys. 2017, 19 (37), 25643–25652. 10.1039/C7CP04738J. 28905070

[ref39] Huynh H. T.; Phan H. T.; Hsu P.-J.; Chen J.-L.; Nguan H. S.; Tsai S.-T.; Roongcharoen T.; Liew C. Y.; Ni C.-K.; Kuo J.-L. Collision-Induced Dissociation of Sodiated Glucose, Galactose, and Mannose, and the Identification of Anomeric Configurations. Phys. Chem. Chem. Phys. 2018, 20 (29), 19614–19624. 10.1039/C8CP03753A. 30009293

[ref40] Chiu C.-c.; Tsai S.-T.; Hsu P.-J.; Huynh H. T.; Chen J.-L.; Phan H. T.; Huang S.-P.; Lin H.-Y.; Kuo J.-L.; Ni C.-K. Unexpected Dissociation Mechanism of Sodiated N-Acetylglucosamine and N-Acetylgalactosamine. J. Phys. Chem. A 2019, 123 (16), 3441–3453. 10.1021/acs.jpca.9b00934. 30945547

[ref41] Nguan H.-S.; Tsai S.-T.; Chen J.-L.; Hsu P.-J.; Kuo J.-L.; Ni C.-K. Collision-Induced Dissociation of Xylose and Its Applications in Linkage and Anomericity Identification. Phys. Chem. Chem. Phys. 2021, 23 (5), 3485–3495. 10.1039/D0CP05868H. 33511385

[ref42] Tsai S.-T.; Nguan H.-S.; Ni C.-K. Identification of Anomericity and Linkage of Arabinose and Ribose through Collision-Induced Dissociation. J. Phys. Chem. A 2021, 125 (28), 6109–6121. 10.1021/acs.jpca.1c03854. 34256570

[ref43] Huynh H. T.; Tsai S.-T.; Hsu P.-J.; Biswas A.; Phan H. T.; Kuo J.-L.; Ni C.-K.; Chiu C.-c. Collision-Induced Dissociation of Na+-Tagged Ketohexoses: Experimental and Computational Studies on Fructose. Phys. Chem. Chem. Phys. 2022, 24 (35), 20856–20866. 10.1039/D2CP02313J. 36043336

[ref44] Nguan H.-S.; Tsai S.-T.; Ni C.-K. Collision-Induced Dissociation of Cellobiose and Maltose. J. Phys. Chem. A 2022, 126 (9), 1486–1495. 10.1021/acs.jpca.1c10046. 35212541

[ref45] Nguan H.-S.; Ni C.-K. Collision-Induced Dissociation of Alpha-Isomaltose and Alpha-Maltose. J. Phys. Chem. A 2022, 126 (47), 8799–8808. 10.1021/acs.jpca.2c04278. 36394324

[ref46] Nguan H.-S.; Tsai S.-T.; Liew C. Y.; Reddy N. S.; Hung S.-C.; Ni C.-K. The Collision-Induced Dissociation Mechanism of Sodiated Hex–Hexnac Disaccharides. Phys. Chem. Chem. Phys. 2023, 25 (33), 22179–22194. 10.1039/D3CP02530F. 37565323

[ref47] Grimme S.; Bannwarth C.; Shushkov P. A Robust and Accurate Tight-Binding Quantum Chemical Method for Structures, Vibrational Frequencies, and Noncovalent Interactions of Large Molecular Systems Parametrized for All Spd-Block Elements (Z = 1–86). J. Chem. Theory Comput. 2017, 13 (5), 1989–2009. 10.1021/acs.jctc.7b00118. 28418654

[ref48] Ballester P. J.; Richards W. G. Ultrafast Shape Recognition to Search Compound Databases for Similar Molecular Shapes. J. Comput. Chem. 2007, 28 (10), 1711–1723. 10.1002/jcc.20681. 17342716

[ref49] Henkelman G.; Uberuaga B. P.; Jónsson H. A Climbing Image Nudged Elastic Band Method for Finding Saddle Points and Minimum Energy Paths. J. Chem. Phys. 2000, 113 (22), 9901–9904. 10.1063/1.1329672.

[ref50] Fukui K. The Path of Chemical Reactions - the Irc Approach. Acc. Chem. Res. 1981, 14 (12), 363–368. 10.1021/ar00072a001.

[ref51] Kühne T. D.; Iannuzzi M.; Del Ben M.; Rybkin V. V.; Seewald P.; Stein F.; Laino T.; Khaliullin R. Z.; Schütt O.; Schiffmann F.; et al. Cp2k: An Electronic Structure and Molecular Dynamics Software Package - Quickstep: Efficient and Accurate Electronic Structure Calculations. J. Chem. Phys. 2020, 152 (19), 194103 10.1063/5.0007045. 33687235

[ref52] Frisch M. J.; Trucks G. W.; Schlegel H. B.; Scuseria G. E.; Robb M. A.; Cheeseman J. R.; Scalmani G.; Barone V.; Petersson G. A.; Nakatsuji H. Gaussian 16, Revision A.03. Gaussian 16, rev. A.03, Gaussian, Inc.: Wallingford, CT, 2016.

[ref53] Weng W. C.; Liao H. E.; Huang S. P.; Tsai S. T.; Hsu H. C.; Liew C. Y.; Gannedi V.; Hung S. C.; Ni C. K. Unusual free oligosaccharides in human bovine and caprine milk. Sci. Rep. 2022, 12, 10790 10.1038/s41598-022-15140-7. 35750794 PMC9232581

