# Supplementary material for: Collision-Induced Dissociations of Linear Hexose and Disaccharides with Linear Hexose at the Reducing End
Source: J Phys Chem A. 2024 Nov 18;128(47):10213–23. doi: 10.1021/acs.jpca.4c05929 (PMC11613545; doi:10.1021/acs.jpca.4c05929)
Supplement: Supplementary file 1 — jp4c05929_si_001.pdf [file jp4c05929_si_001.pdf]

Supporting Information

Collision-Induced Dissociations of Linear Hexose and Disaccharides with Linear Hexose at the Reducing End

Hock-Seng Nguan[1*], Hsu-Chen Hsu[1], Wun-Long Li[1,2], Chia Yen Liew[1,3], and Chi-Kung Ni[1,4*]

1 Institute of Atomic and Molecular Sciences, Academia Sinica, P. O. Box 23-166, Taipei 10617, Taiwan.

2. Department of Chemistry, National Taiwan Normal University, Taipei 11677, Taiwan

3. Current address: Division of Biology and Biological Engineering, California Institute of Technology, CA, 91125, USA.

4 Department of Chemistry, National Tsing Hua University, Hsinchu 30013, Taiwan

Corresponding authors: E-mail: nguanhs@gmail.com, ckni@po.iams.sinica.edu.tw

Contents **Page**

Figure S1: Calculations results of Glc-L1 C-C bond cleavage reaction.	3
Figure S2: Calculations results of Glc-L1 C=O migration.	3
Figure S3: Calculations results of Glc-L2 C-C bond cleavage reaction.	4
Figure S4: Calculations results of Glc-L2 C=O migration.	4
Figure S5: Calculations results of Glc-L3a C-C bond cleavage reaction.	5
Figure S6: Calculations results of Glc-L3a C=O migration.	6
Figure S7: Calculations results of Glc-L3b C-C bond cleavage reaction.	7
Figure S8: Calculations results of Glc-L3b C=O migration.	7
Figure S9: Calculations results of Man-L1 C-C bond cleavage reaction.	8
Figure S10: Calculations results of Man-L1 C=O migration.	8
Figure S11: Calculations results of Gal-L1 C-C bond cleavage reaction.	9
Figure S12: Calculations results of Gal-L1 C=O migration.	9
Figure S13: Calculations results of Gal-L2 C-C bond cleavage reaction.	10
Figure S14: Calculations results of Gal-L2 C=O migration.	10
Figure S15: Calculations results of Gal-L3a C-C bond cleavage reaction.	11
Figure S16: Calculations results of Gal-L3a C=O migration.	11
Figure S17: Calculations results of Gal-L3b C-C bond cleavage reaction.	12
Figure S18: Calculations results of Gal-L3b C=O migration.	12
Figure S19: Calculations results of Iso-L1 C-C bond cleavage reaction.	13
Figure S20: Calculations results of Iso-L1 C=O migration.	14
Figure S21: Calculations results of Iso-L2 C-C bond cleavage reaction.	15
Figure S22: Calculations results of Iso-L2 C=O migration.	16
Figure S23: Calculations results of Iso-L3a C-C bond cleavage reaction.	16
Figure S24: Calculations results of Iso-L3a C=O migration.	17
Figure S25: Geometries of the lowest TSs for isomaltose linear forms.	18
Figure S26: Geometries of the global minimum.	18
Figure S27: Calculations results of glucose linear forms dehydration reaction.	19
Figure S28: Calculations results of galactose linear forms dehydration reaction.	19
Figure S29: Geometries of the lowest dehydration TSs.	20
XYZ coordinates for minimum and transition states	21-215

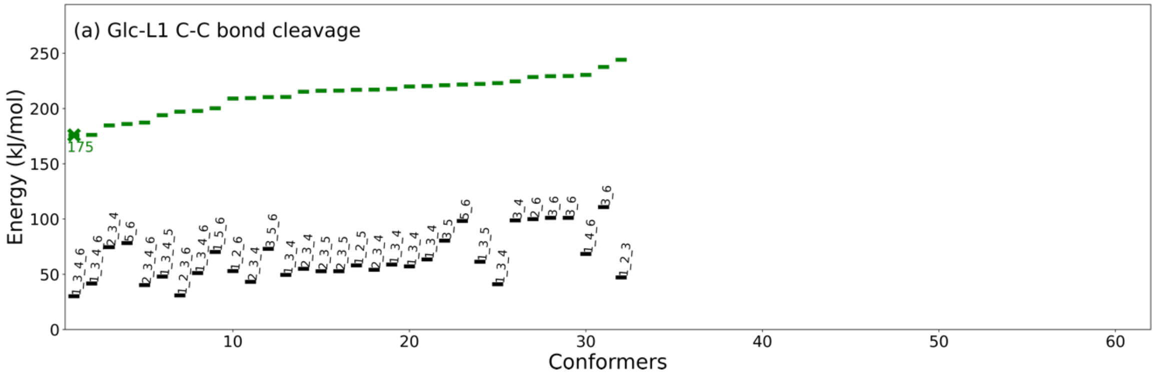

Figure S1. Calculated zero-point corrected energies of TSs and reactants of Glc-L1 for C-C bond cleavage reaction. The green lines represent the TS energies of C-C bond, the cross is the energy of the lowest TSs from our previous study[1] reoptimized using DFT/M06-2X method. The black lines right below each TS represent the energies of the reactant states leading to the TSs, where the numbers above the black line represent the numberings of the O atoms that bind to sodium ion.

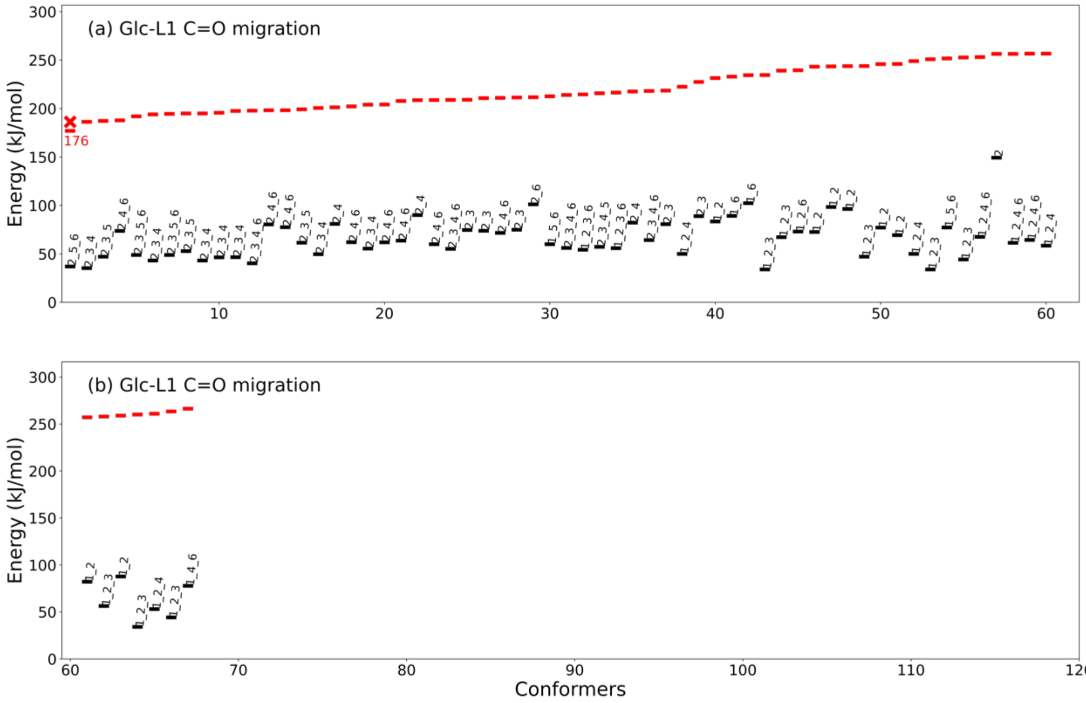

Figure S2. Calculated zero-point corrected energies of TSs and reactants of Glc-L1 for C=O migration. The red lines represent the TS energies of C-C bond, the cross is the energy of the lowest TSs from our previous study[1] reoptimized using DFT/M06-2X method. The black lines right below each TS represent the energies of the reactant states leading to the TSs, where the numbers above the black line represent the numberings of the O atoms that bind to sodium ion.

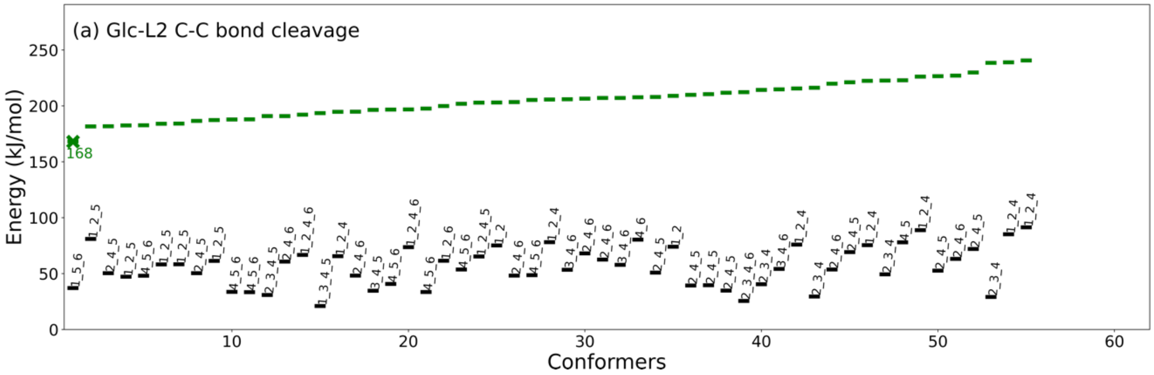

Figure S3. Calculated zero-point corrected energies of TSs and reactants of Glc-L2 for C-C bond cleavage reaction. The green lines represent the TS energies of C-C bond, the cross is the energy of the lowest TSs from our previous study[1] reoptimized using DFT/M06-2X method. The black lines right below each TS represent the energies of the reactant states leading to the TSs, where the numbers above the black line represent the numberings of the O atoms that bind to sodium ion.

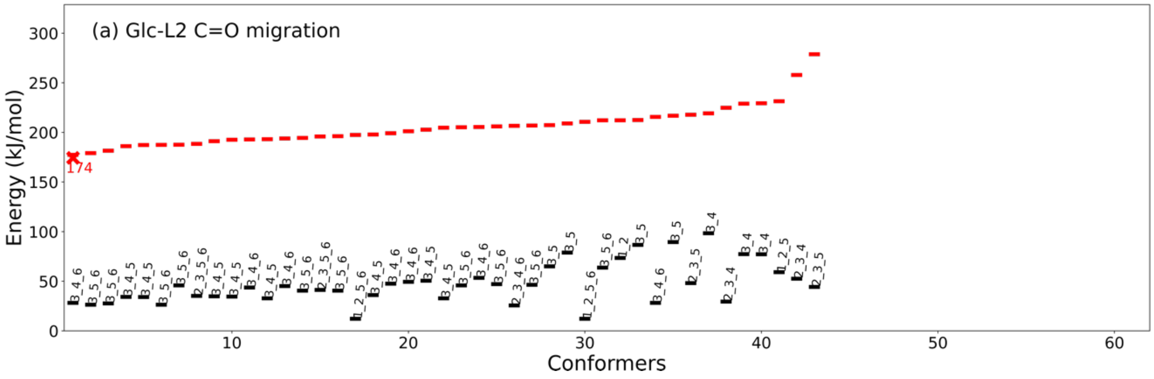

Figure S4. Calculated zero-point corrected energies of TSs and reactants of Glc-L2 for C=O migration. The red lines represent the TS energies of C-C bond, the cross is the energy of the lowest TSs from our previous study[1] reoptimized using DFT/M06-2X method. The black lines right below each TS represent the energies of the reactant states leading to the TSs, where the numbers above the black line represent the numberings of the O atoms that bind to sodium ion.

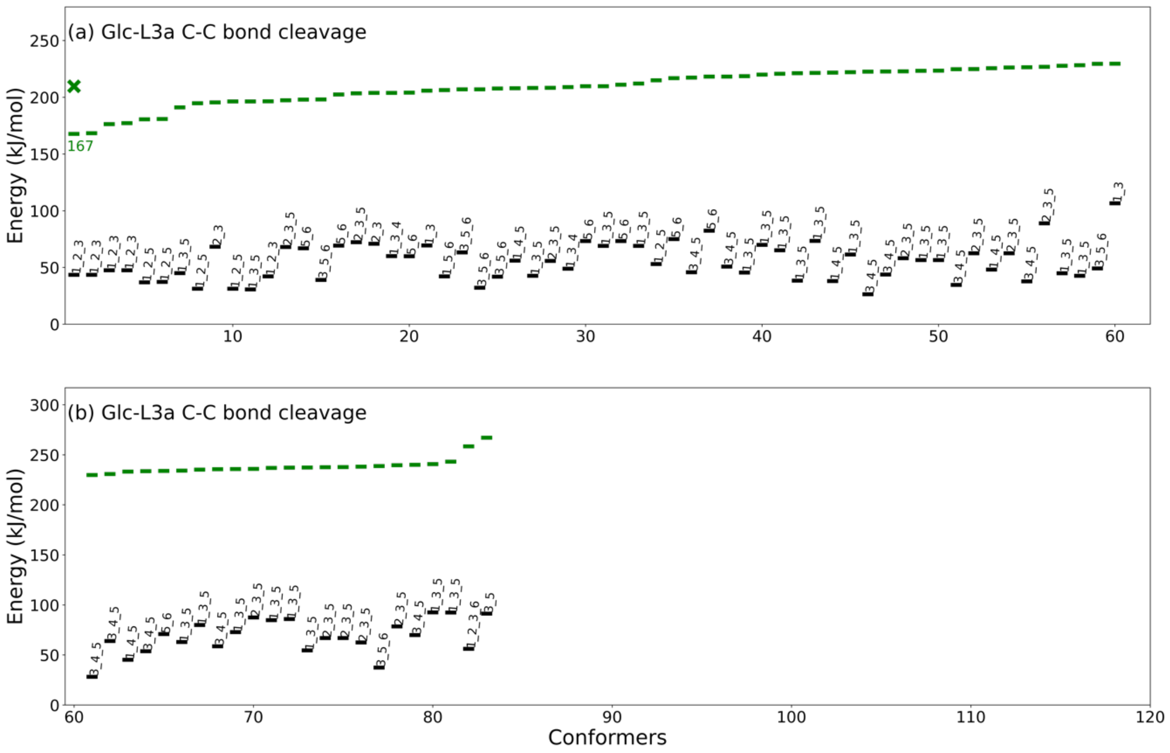

Figure S5. Calculated zero-point corrected energies of TSs and reactants of Glc-L3a for C-C bond cleavage reaction. The green lines represent the TS energies of C-C bond, the cross is the energy of the lowest TSs from our previous study[1] reoptimized using DFT/M06-2X method. The black lines right below each TS represent the energies of the reactant states leading to the TSs, where the numbers above the black line represent the numberings of the O atoms that bind to sodium ion.

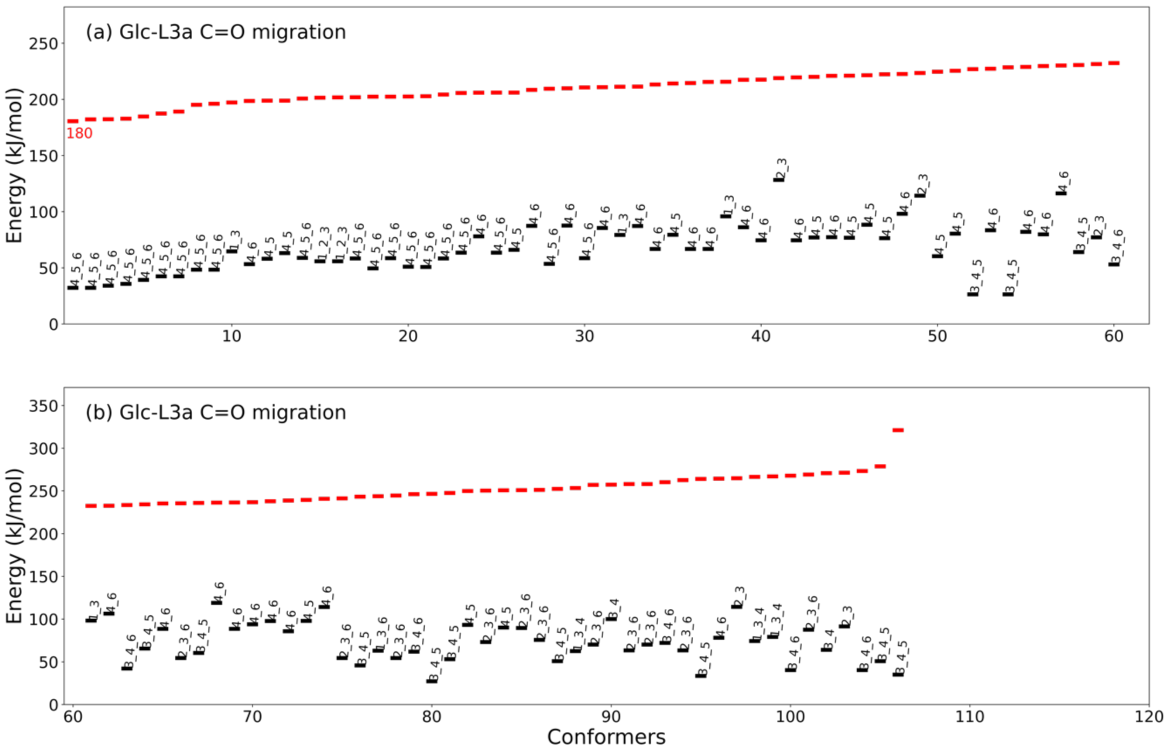

Figure S6. Calculated zero-point corrected energies of TSs and reactants of Glc-L3a for C=O migration. The red lines represent the TS energies of C-C bond. The black lines right below each TS represent the energies of the reactant states leading to the TSs, where the numbers above the black line represent the numberings of the O atoms that bind to sodium ion.

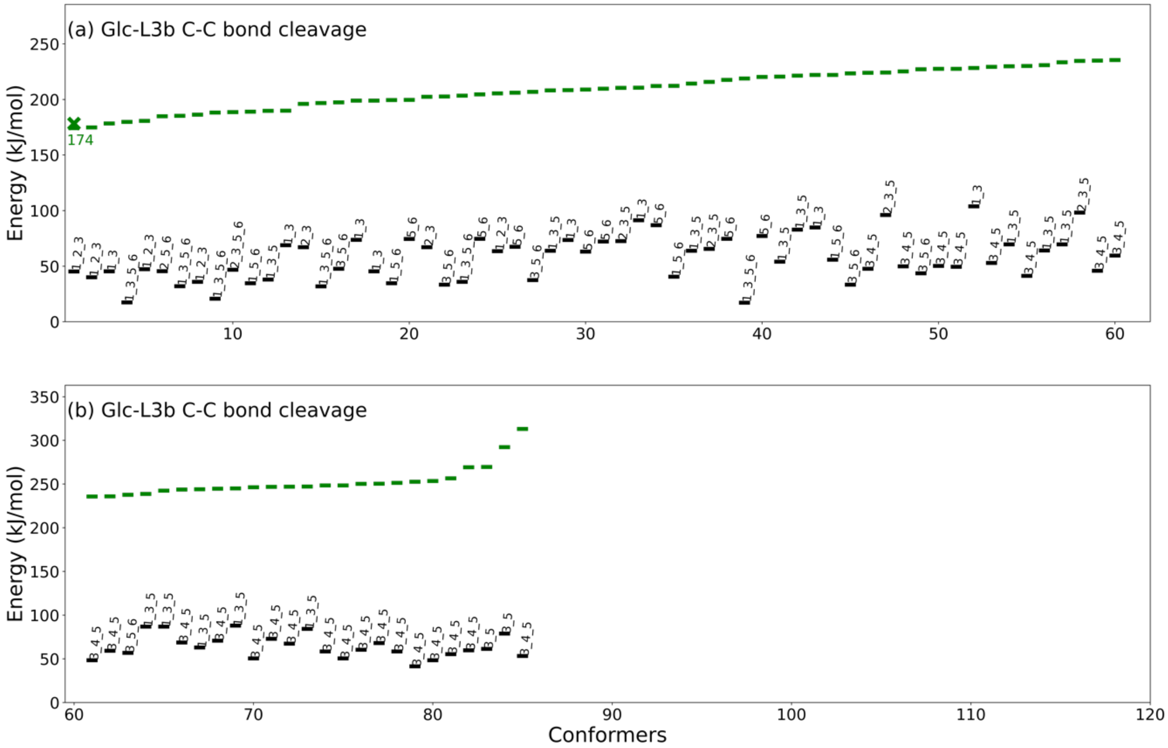

Figure S7. Calculated zero-point corrected energies of TSs and reactants of Glc-L3b for C-C bond cleavage reaction. The green lines represent the TS energies of C-C bond, the cross is the energy of the lowest TSs from our previous study[1] reoptimized using DFT/M06-2X method. The black lines right below each TS represent the energies of the reactant states leading to the TSs, where the numbers above the black line represent the numberings of the O atoms that bind to sodium ion.

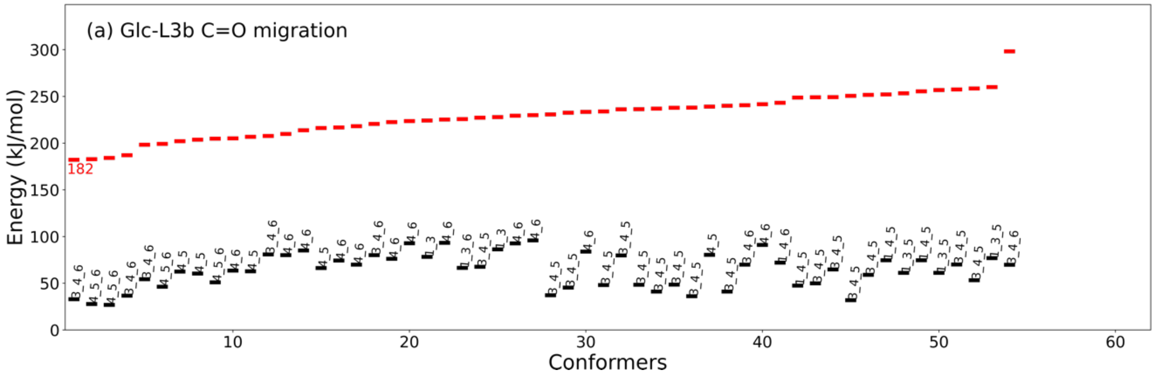

Figure S8. Calculated zero-point corrected energies of TSs and reactants of Glc-L3b for C=O migration. The red lines represent the TS energies of C-C bond. The black lines right below each TS represent the energies of the reactant states leading to the TSs, where the numbers above the black line represent the numberings of the O atoms that bind to sodium ion.

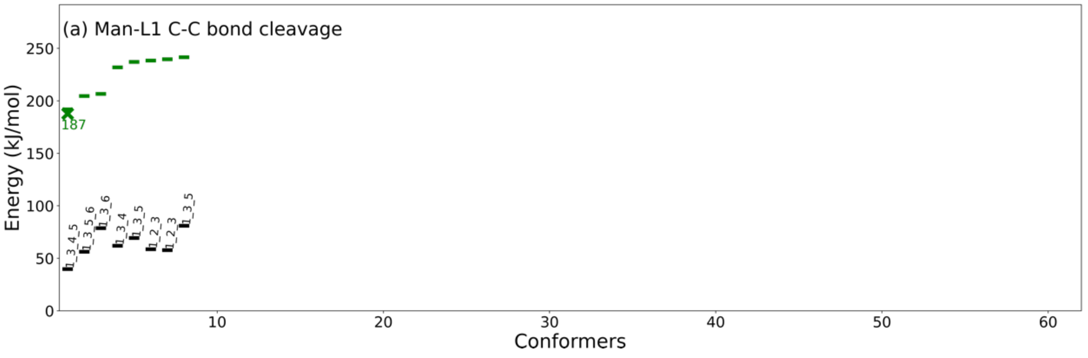

Figure S9. Calculated zero-point corrected energies of TSs and reactants of Man-L1 for C-C bond cleavage reaction. The green lines represent the TS energies of C-C bond, the cross is the energy of the lowest TSs from our previous study[1] reoptimized using DFT/M06-2X method. The black lines right below each TS represent the energies of the reactant states leading to the TSs, where the numbers above the black line represent the numberings of the O atoms that bind to sodium ion.

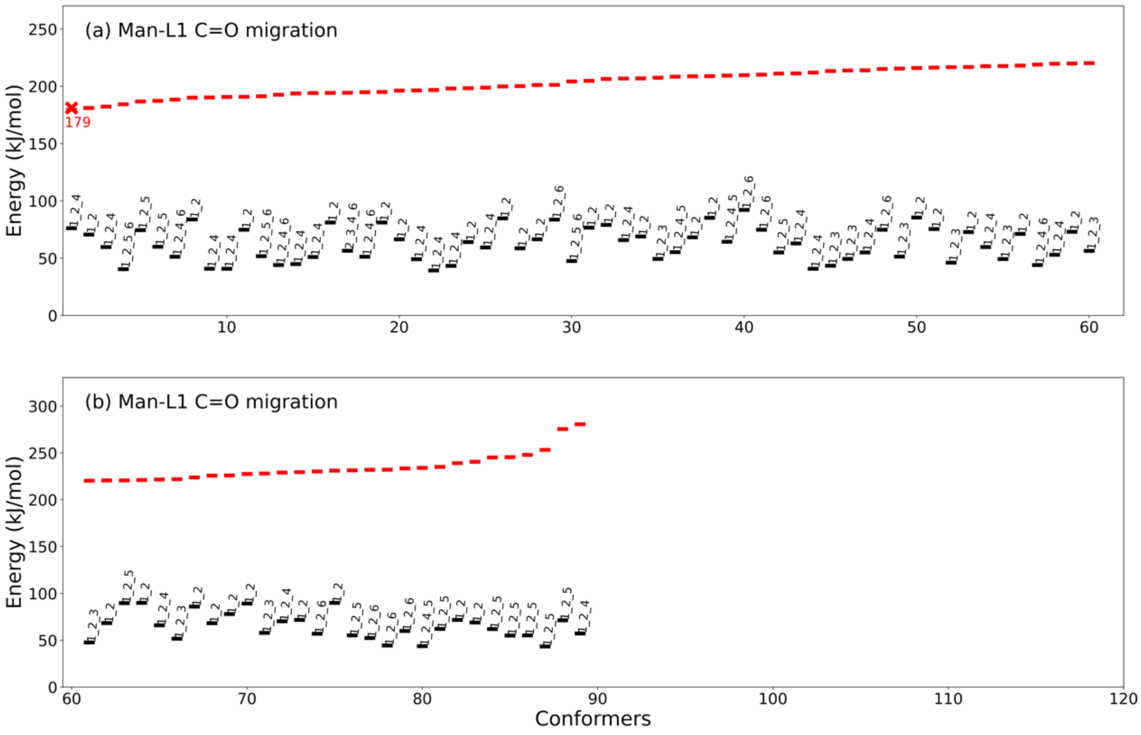

Figure S10. Calculated zero-point corrected energies of TSs and reactants of Man-L1 for C=O migration. The red lines represent the TS energies of C-C bond, the cross is the energy of the lowest TSs from our previous study[1] reoptimized using DFT/M06-2X method. The black lines right below each TS represent the energies of the reactant states leading to the TSs, where the numbers above the black line represent the numberings of the O atoms that bind to sodium ion.

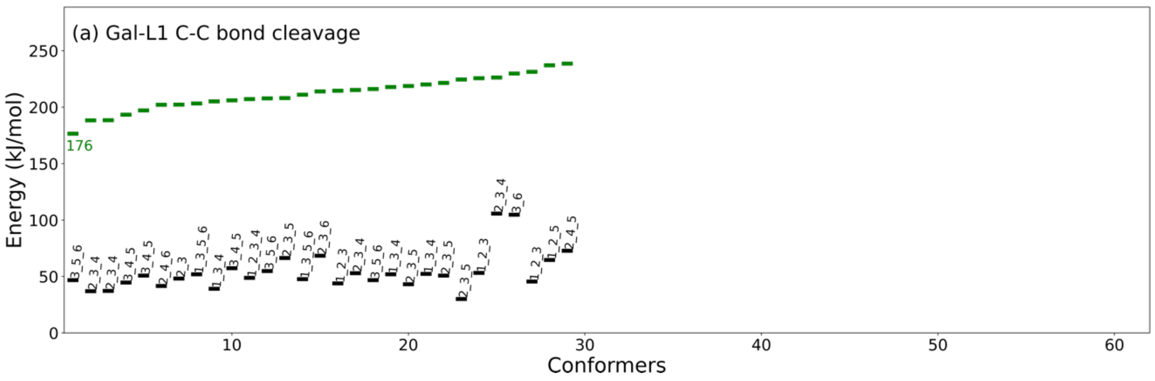

Figure S11. Calculated zero-point corrected energies of TSs and reactants of Gal-L1 for C-C bond cleavage reaction. The green lines represent the TS energies of C-C bond. The black lines right below each TS represent the energies of the reactant states leading to the TSs, where the numbers above the black line represent the numberings of the O atoms that bind to sodium ion.

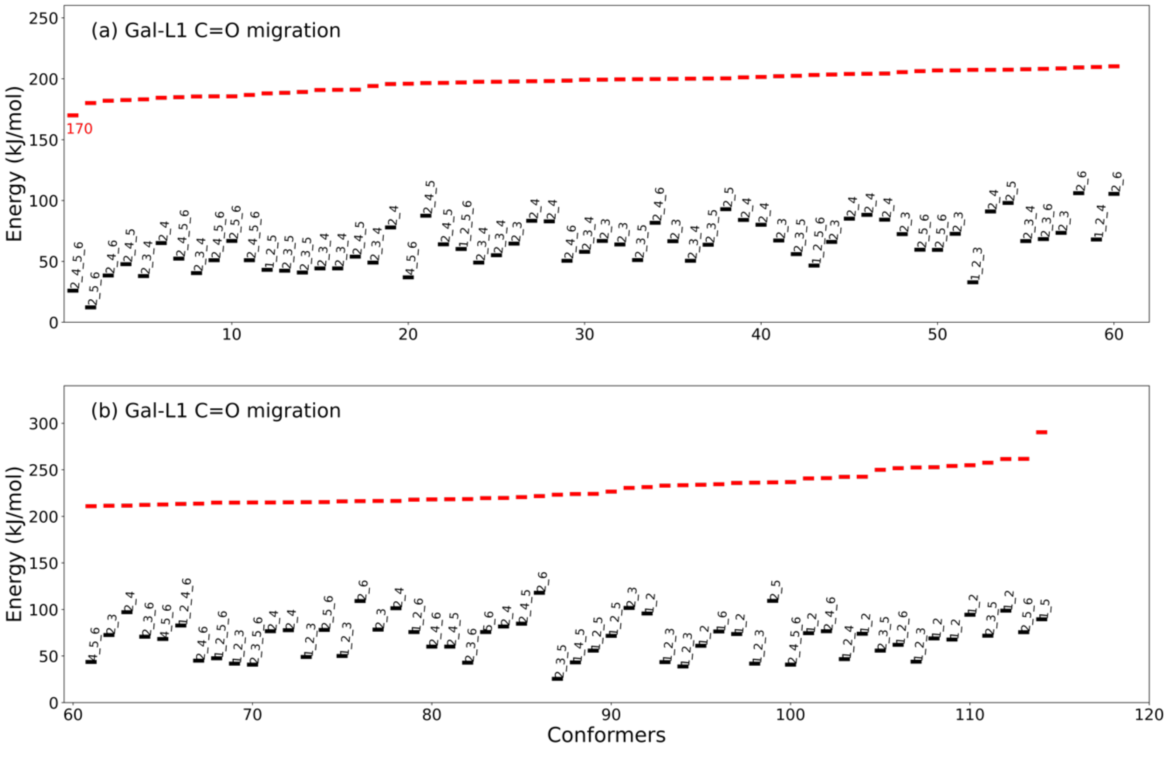

Figure S12. Calculated zero-point corrected energies of TSs and reactants of Gal-L1 for C=O migration. The red lines represent the TS energies of C-C bond. The black lines right below each TS represent the energies of the reactant states leading to the TSs, where the numbers above the black line represent the numberings of the O atoms that bind to sodium ion.

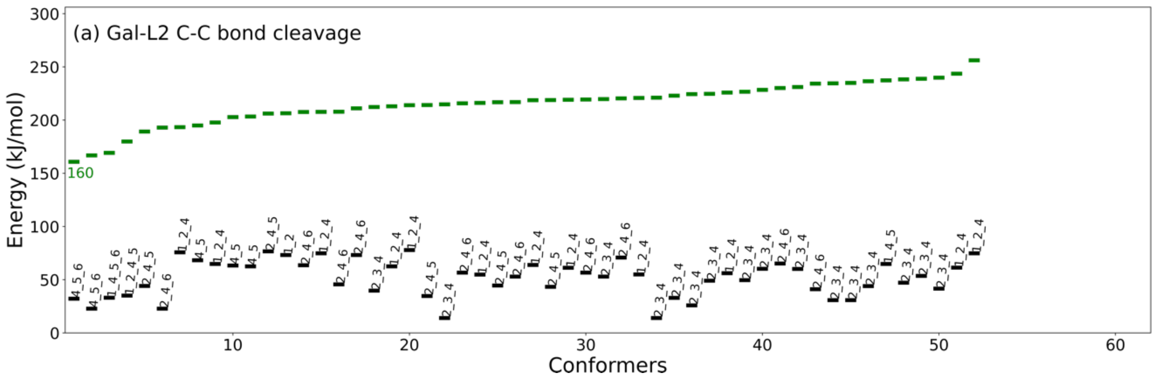

Figure S13. Calculated zero-point corrected energies of TSs and reactants of Gal-L2 for C-C bond cleavage reaction. The green lines represent the TS energies of C-C bond. The black lines right below each TS represent the energies of the reactant states leading to the TSs, where the numbers above the black line represent the numberings of the O atoms that bind to sodium ion.

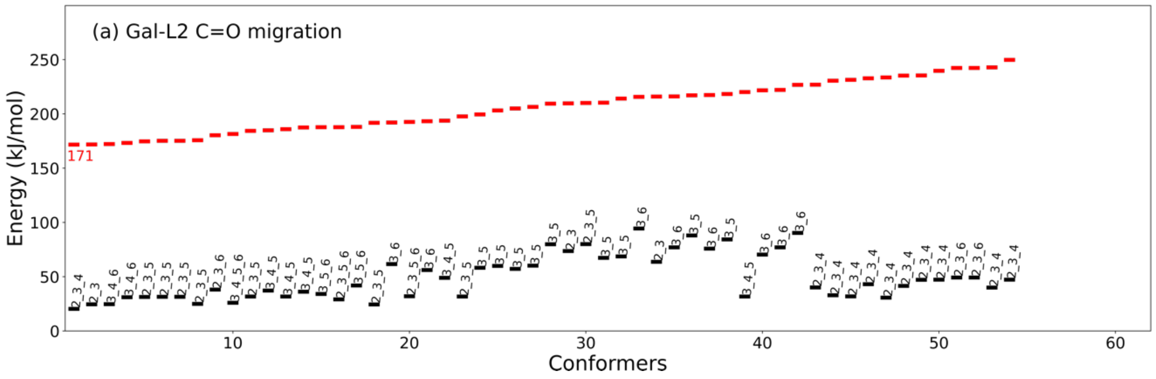

Figure S14. Calculated zero-point corrected energies of TSs and reactants of Gal-L2 for C=O migration. The red lines represent the TS energies of C-C bond. The black lines right below each TS represent the energies of the reactant states leading to the TSs, where the numbers above the black line represent the numberings of the O atoms that bind to sodium ion.

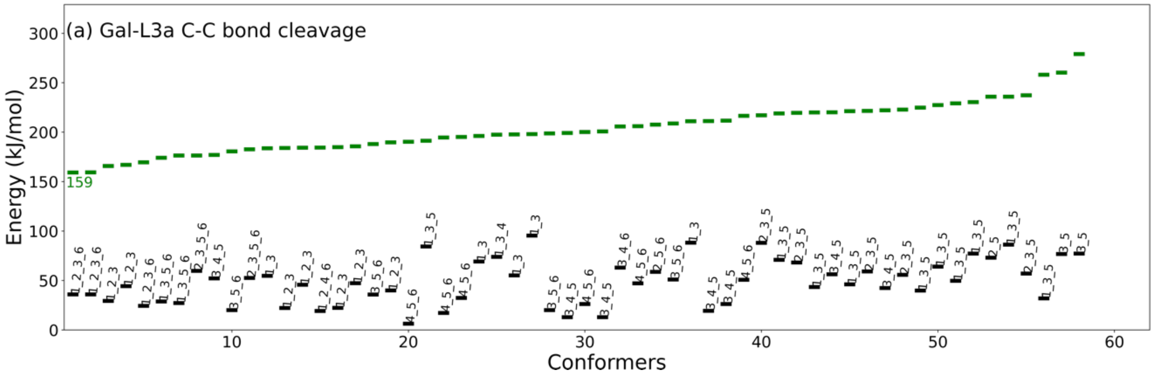

Figure S15. Calculated zero-point corrected energies of TSs and reactants of Gal-L3a for C-C bond cleavage reaction. The green lines represent the TS energies of C-C bond. The black lines right below each TS represent the energies of the reactant states leading to the TSs, where the numbers above the black line represent the numberings of the O atoms that bind to sodium ion.

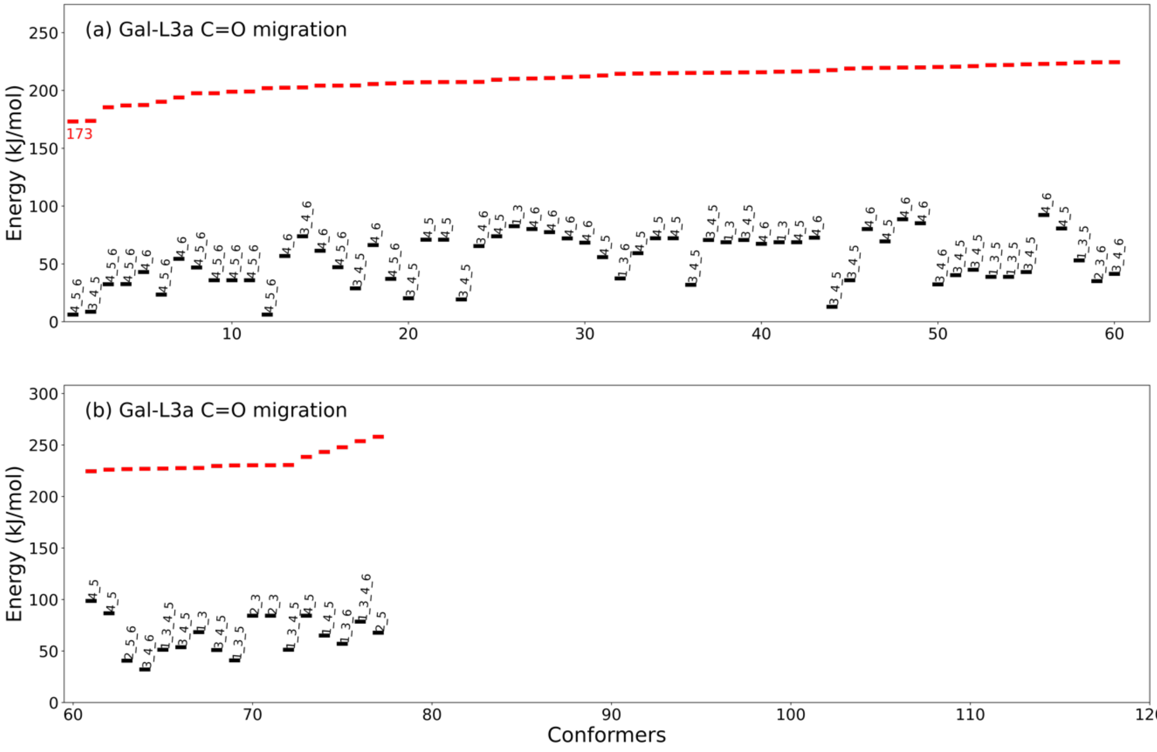

Figure S16. Calculated zero-point corrected energies of TSs and reactants of Gal-L3a for C=O migration. The red lines represent the TS energies of C-C bond. The black lines right below each TS represent the energies of the reactant states leading to the TSs, where the numbers above the black line represent the numberings of the O atoms that bind to sodium ion.

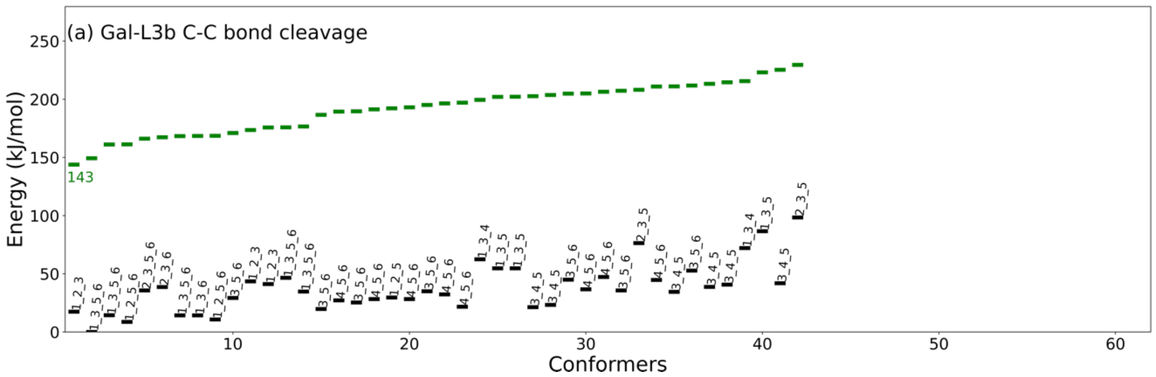

Figure S17. Calculated zero-point corrected energies of TSs and reactants of Gal-L3b for C-C bond cleavage reaction. The green lines represent the TS energies of C-C bond. The black lines right below each TS represent the energies of the reactant states leading to the TSs, where the numbers above the black line represent the numberings of the O atoms that bind to sodium ion.

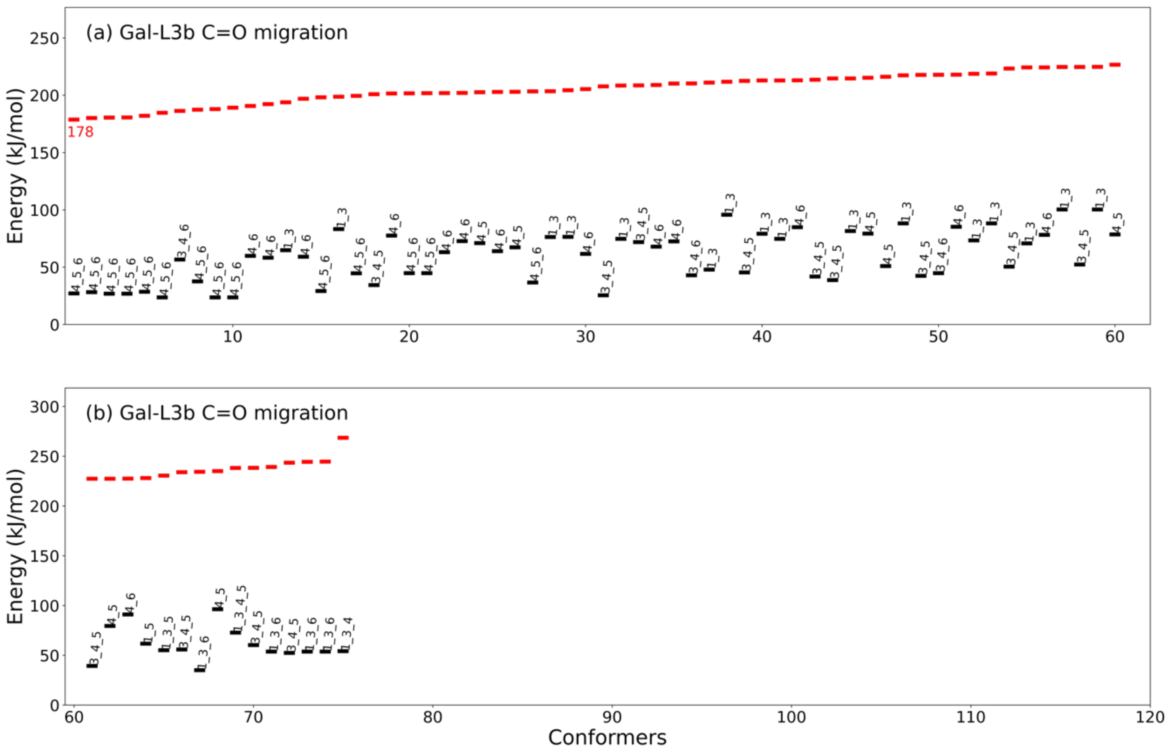

Figure S18. Calculated zero-point corrected energies of TSs and reactants of Gal-L3b for C=O migration. The red lines represent the TS energies of C-C bond. The black lines right below each TS represent the energies of the reactant states leading to the TSs, where the numbers above the black line represent the numberings of the O atoms that bind to sodium ion.

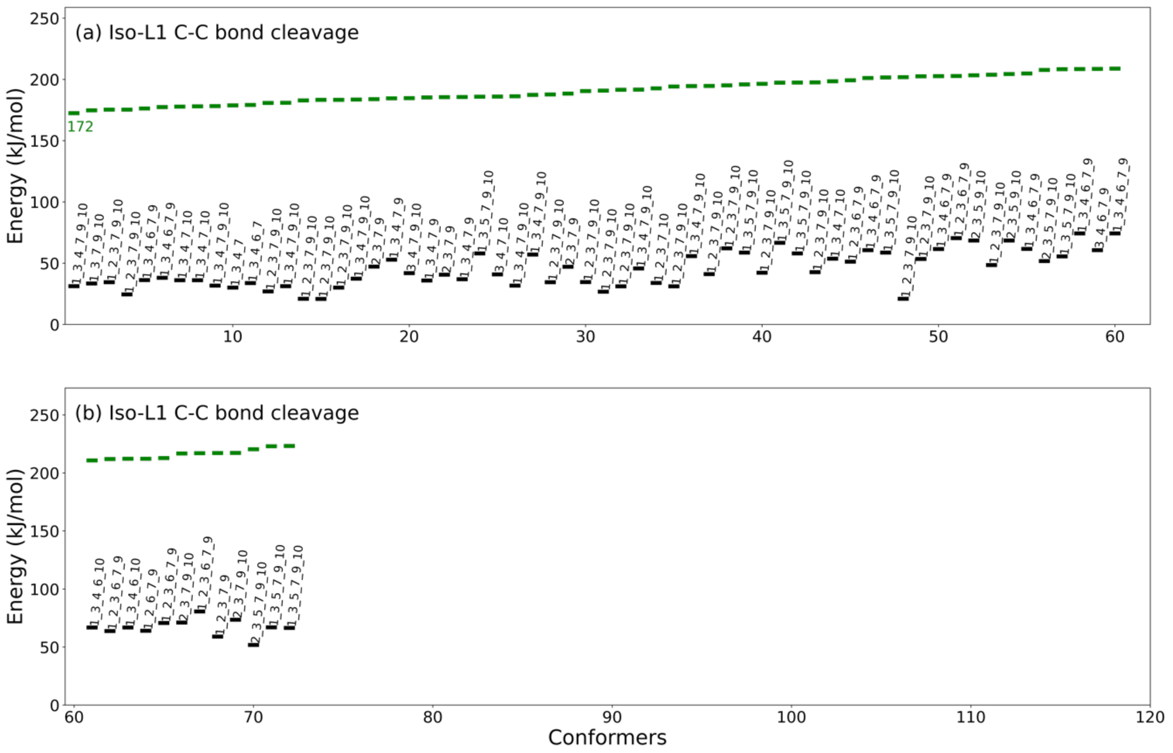

Figure S19. Calculated zero-point corrected energies of TSs and reactants of Iso-L1 for C-C bond cleavage reaction. The green lines represent the TS energies of C-C bond. The black lines right below each TS represent the energies of the reactant states leading to the TSs, where the numbers above the black line represent the numberings of the O atoms that bind to sodium ion.

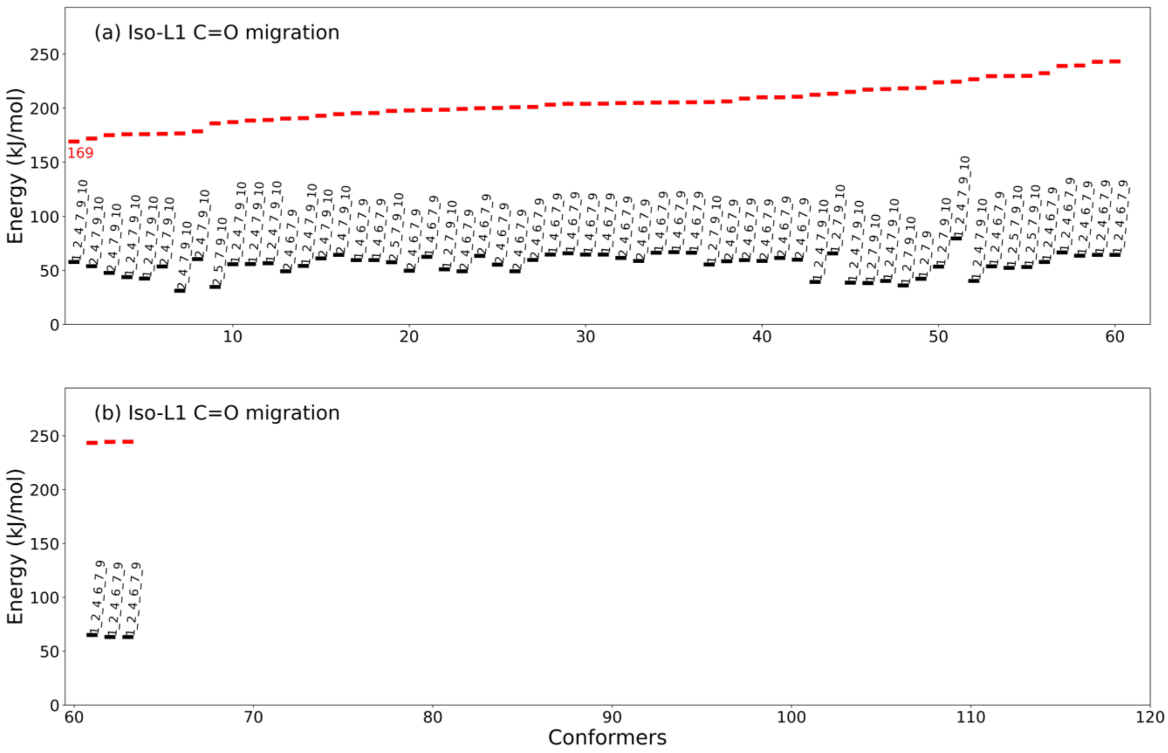

Figure S20. Calculated zero-point corrected energies of TSs and reactants of Iso-L1 for C=O migration. The red lines represent the TS energies of C-C bond. The black lines right below each TS represent the energies of the reactant states leading to the TSs, where the numbers above the black line represent the numberings of the O atoms that bind to sodium ion.

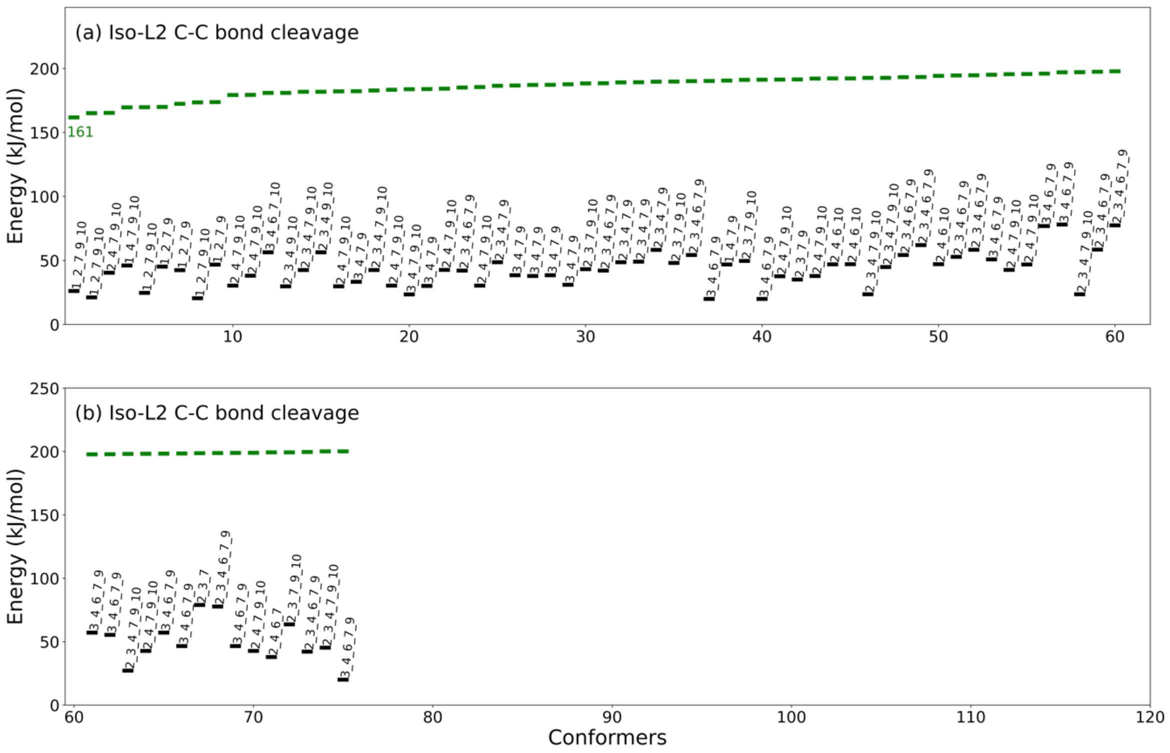

Figure S21. Calculated zero-point corrected energies of TSs and reactants of Iso-L2 for C-C bond cleavage reaction. The green lines represent the TS energies of C-C bond. The black lines right below each TS represent the energies of the reactant states leading to the TSs, where the numbers above the black line represent the numberings of the O atoms that bind to sodium ion.

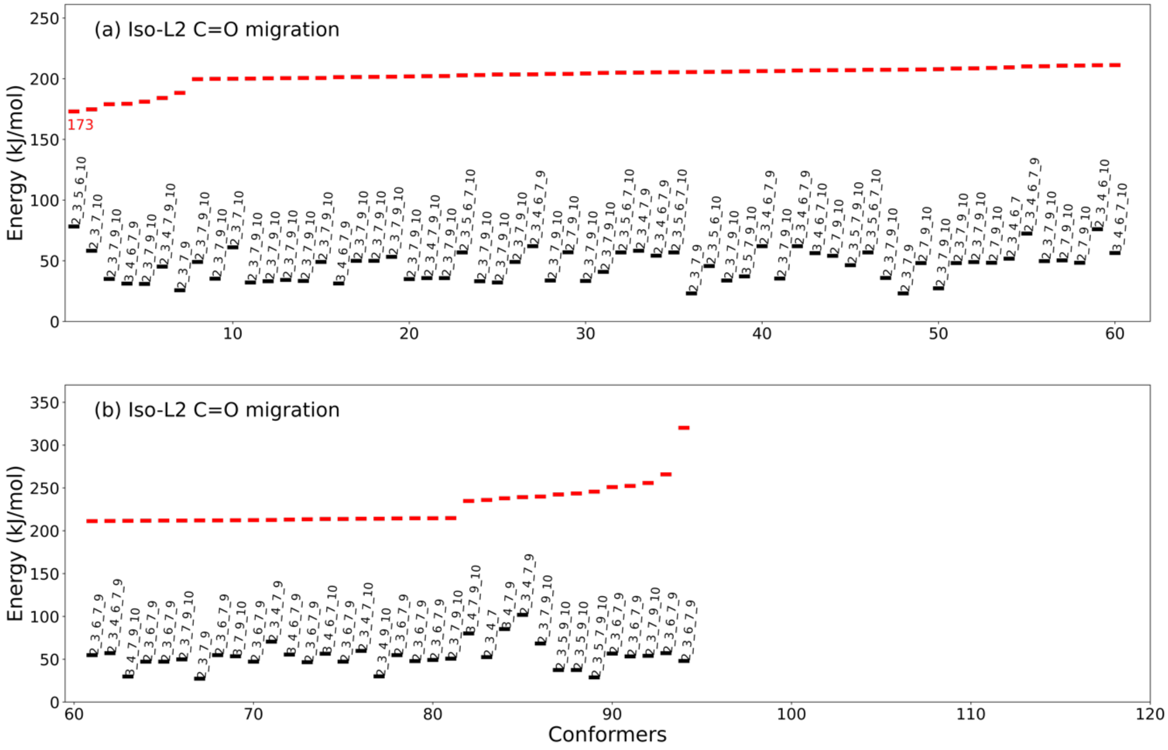

Figure S22. Calculated zero-point corrected energies of TSs and reactants of Iso-L2 for C=O migration. The red lines represent the TS energies of C-C bond. The black lines right below each TS represent the energies of the reactant states leading to the TSs, where the numbers above the black line represent the numberings of the O atoms that bind to sodium ion.

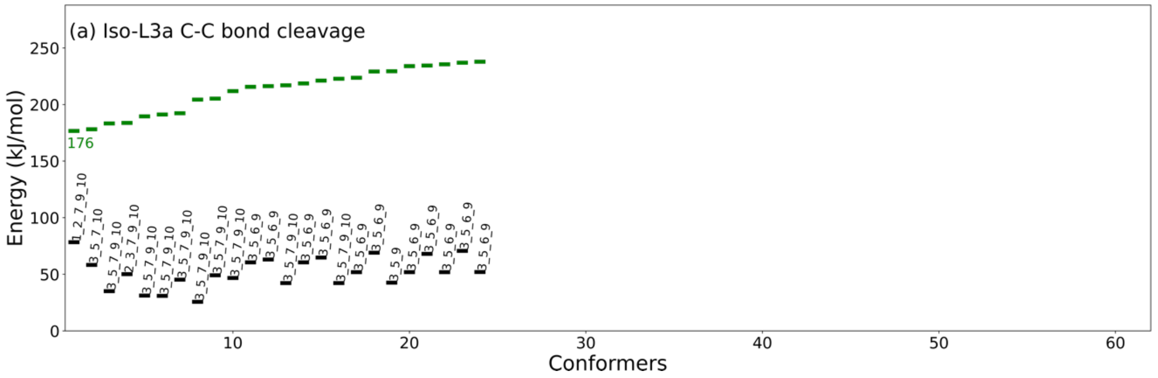

Figure S23. Calculated zero-point corrected energies of TSs and reactants of Iso-L3a for C-C bond cleavage reaction. The green lines represent the TS energies of C-C bond. The black lines right below each TS represent the energies of the reactant states leading to the TSs, where the numbers above the black line represent the numberings of the O atoms that bind to sodium ion.

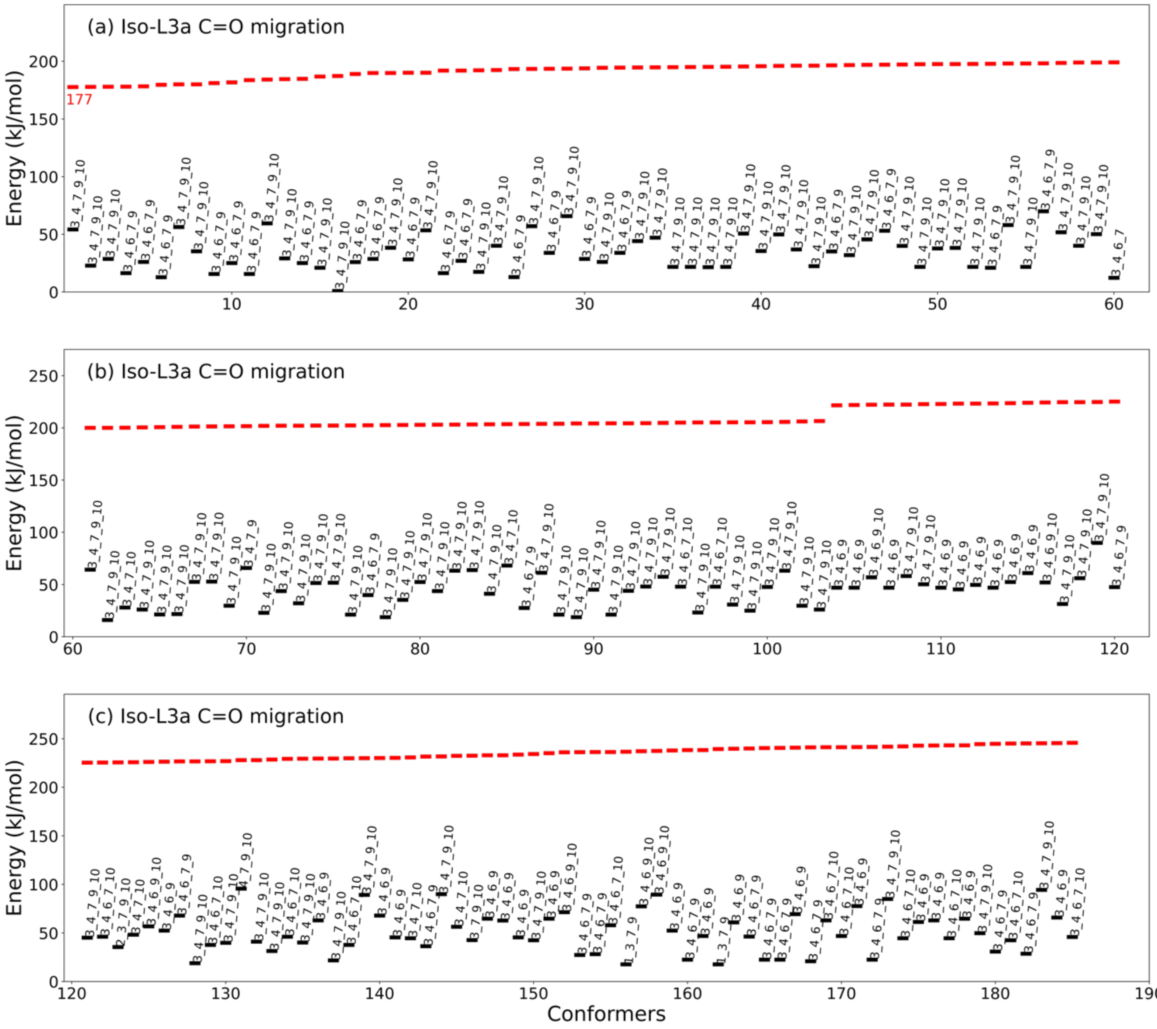

Figure S24. Calculated zero-point corrected energies of TSs and reactants of Iso-L3a for C=O migration. The red lines represent the TS energies of C-C bond. The black lines right below each TS represent the energies of the reactant states leading to the TSs, where the numbers above the black line represent the numberings of the O atoms that bind to sodium ion.

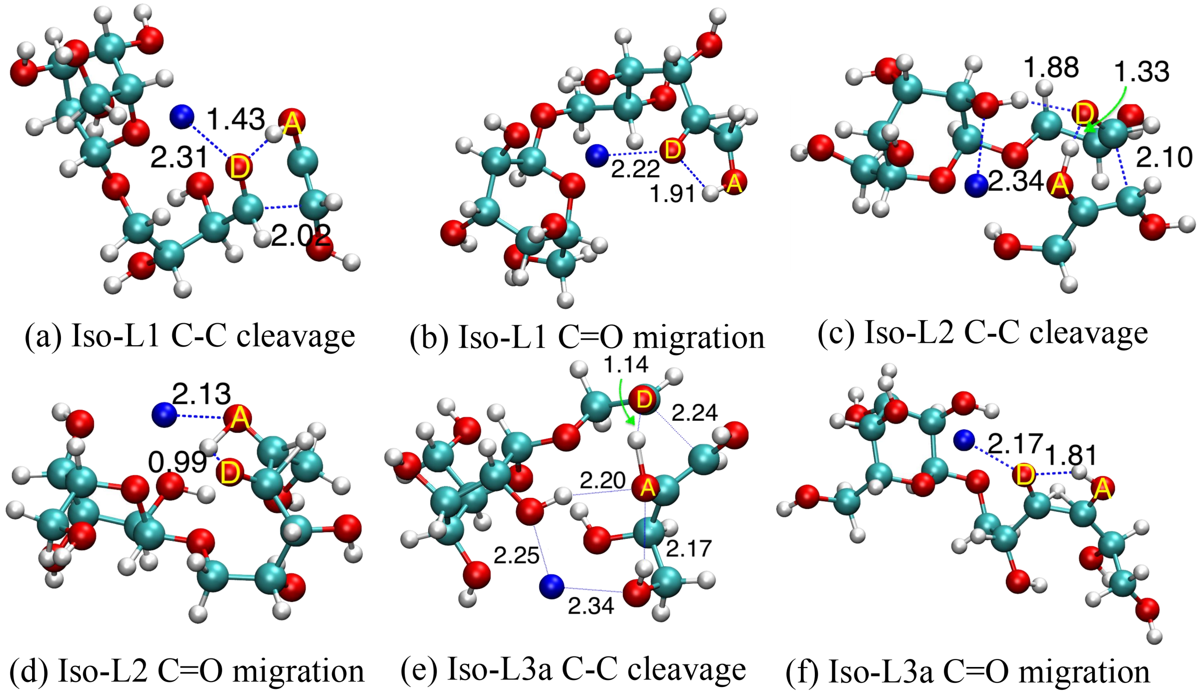

Figure S25. Geometries of the lowest TSs of C-C bond cleavage and C=O migration for the isomaltose linear forms.

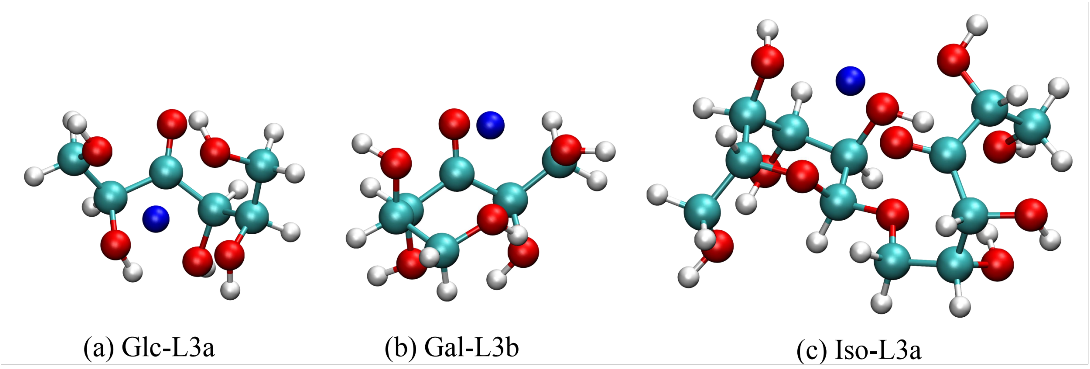

Figure S26. Geometries of the global minimum for linear forms of glucose and mannose, galactose and isomaltose, which form belongs to (a) Glc-L3a, (b) Gal-L3b and (a) Iso-L3a, respectively.

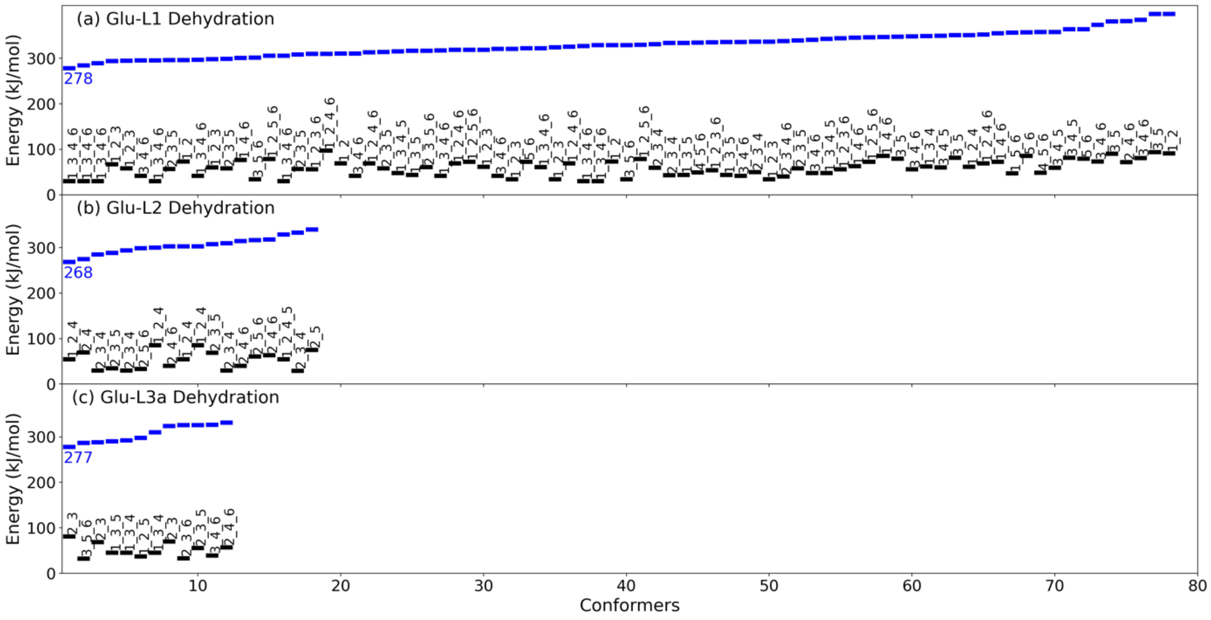

Figure S27. Calculated zero-point corrected energies of TSs and reactants of dehydration reactions for glucose in linear forms. The blue lines represent the TS energies of dehydration reactions. The black lines right below each TS represent the energies of the reactant states leading to the TSs, where the numbers above the black line represent the numberings of the O atoms that bind to sodium ion.

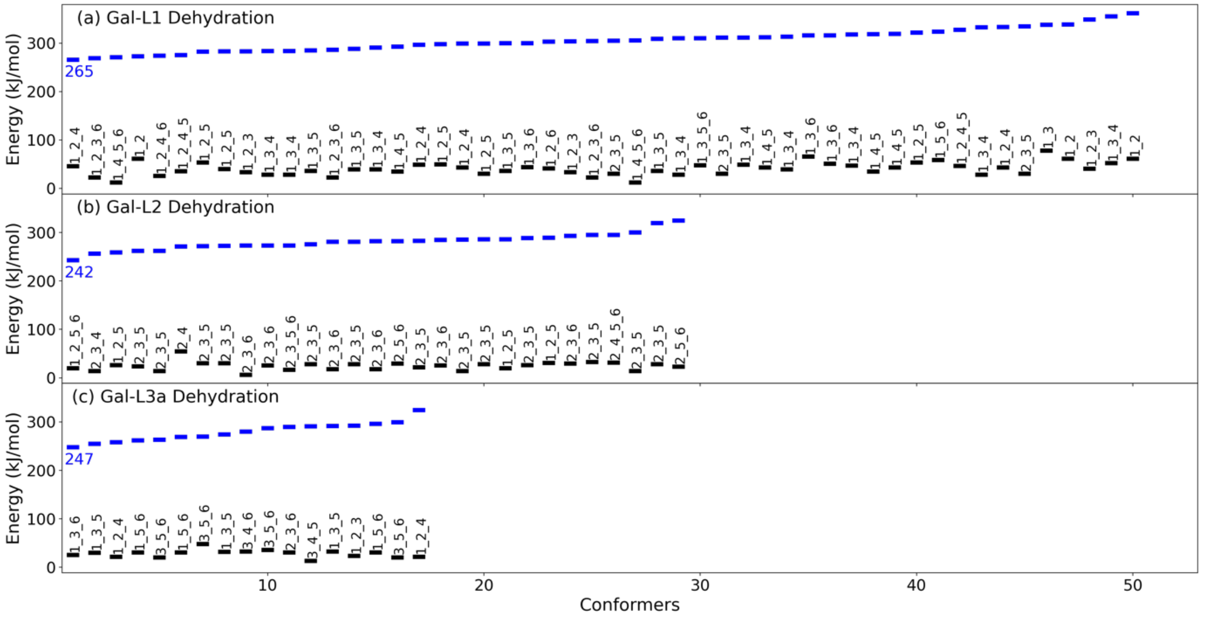

Figure S28. Calculated zero-point corrected energies of TSs and reactants of dehydration reactions for galactose in linear forms. The blue lines represent the TS energies of dehydration reactions. The black lines right below each TS represent the energies of the reactant states leading to the TSs, where the numbers above the black line represent the numberings of the O atoms that bind to sodium ion.

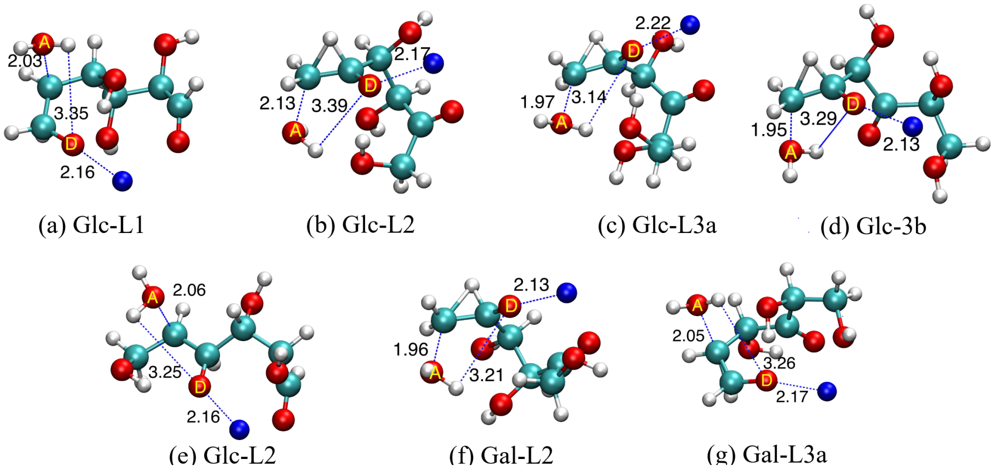

Figure S29. Geometries of the lowest TSs of dehydration for the series of linear structures.

Reference:

1. Huynh, H. T.; Tsai, S.-T.; Hsu, P.-J.; Biswas, A.; Phan, H. T.; Kuo, J.-L.; Ni, C.-K.; Chiu, C.- Collision-Induced Dissociation of Na+-Tagged Ketohexoses: Experimental and Computational Studies on Fructose. *Phys. Chem. Chem. Phys.* **2022**, *24* (35), 20856-20866

1. The global minimum glucose linear forms: Glu-L3a

25

C -2.58345 0.372459 0.272447
C -1.339479 1.173685 -0.050122
C -0.206353 0.871889 0.946168
O -0.434972 0.462322 2.053606
C 1.247879 1.13115 0.524268
C 2.016908 -0.132813 0.072606
C 1.753984 -1.371103 0.918448
O 0.458373 -1.885315 0.621034
O 1.675812 -0.473244 -1.265551
O 1.30831 2.07422 -0.540733
O -0.953772 0.851104 -1.377347
O -2.272355 -1.003737 0.031417
H 3.083693 0.108981 0.126037
H -3.394977 0.696964 -0.382344
H 1.741304 1.512758 1.422651
H 2.484197 -2.136533 0.654261
H 1.852789 -1.138912 1.980543
H 1.66416 2.914535 -0.23887
H -0.134802 -1.74898 1.366788
H 2.021155 0.211275 -1.848791
H -3.013751 -1.54882 0.310125
H -2.85457 0.528997 1.317804
H -0.272957 1.480156 -1.650245
H -1.581726 2.240165 0.038823
Na -0.471147 -1.417726 -1.398097

2. The global minimum galactose linesr forms: Gal-L3b

25

C	-2.599146	0.344766	-0.228663
C	-1.357574	1.219519	-0.234944
C	-0.220496	0.509003	-0.983913
O	-0.458063	-0.403297	-1.741976
C	1.22723	0.953211	-0.791699
C	2.055927	-0.103705	-0.005005
C	1.62968	-0.242981	1.44723
O	0.34021	-0.836126	1.505087
O	1.902113	-1.394477	-0.581243
O	1.271954	2.205686	-0.136643
O	-1.010478	1.515882	1.103164
O	-2.284946	-0.907843	0.394115
H	3.103435	0.215859	-0.035605
H	-2.93692	0.170664	-1.25101
H	1.637581	1.020129	-1.805236
H	2.332649	-0.904535	1.955307
H	1.640904	0.730805	1.93978
H	1.936426	2.778635	-0.528713
H	-0.313065	-0.151733	1.718387
H	2.385096	-1.456545	-1.411233
H	-3.001887	-1.157989	0.982052
H	-3.376886	0.863151	0.333042
H	-0.369085	2.238029	1.104167
H	-1.585895	2.137404	-0.793355
Na	-0.359308	-2.18194	-0.160513

3. The global minimum isomaltose linesr forms: Iso-L3b

46

O 0.321239 1.02582 0.531308
O -0.531745 -1.456411 1.036146
O -3.718963 0.001802 1.903922
O -2.601532 -2.067846 -0.902006
O -1.353357 0.494314 -0.900659
O -3.760762 2.057396 0.08095
C -1.048432 0.821463 0.46115
C -1.377154 -0.365912 1.364631
C -2.838538 -0.811469 1.180549
C -3.226814 -0.919733 -0.310058
C -2.733411 0.245921 -1.17751
C -3.593283 1.500412 -1.217405
H 0.37214 -1.271704 1.35284
H -3.852245 0.846016 1.441571
H -2.92488 -2.868032 -0.469284
H -4.361961 2.811371 0.047463
H -1.600473 1.724722 0.75331
H -1.250438 -0.067835 2.411866
H -2.915871 -1.814015 1.617713
H -4.31827 -1.003975 -0.382041
H -2.739313 -0.123811 -2.209674
H -3.100437 2.221957 -1.878714
H -4.56448 1.22865 -1.64912
O 2.115544 -0.885478 1.879872
O 1.883605 -2.434748 -0.53508
O 1.357742 -0.278159 -1.807519
O 4.378498 0.999599 -0.606711
O 2.725635 1.897043 1.320292
C 3.251431 -1.27971 1.110285
C 2.862032 -1.422562 -0.357463
C 2.318781 -0.178364 -1.072254
C 3.033058 1.161852 -0.970228
C 2.324748 2.1546 -0.004999
C 0.806745 2.18741 -0.134778
H 2.198745 1.153934 1.661439
H 2.28703 -1.061582 2.813869
H 2.264983 -3.298594 -0.338142
H 4.508459 1.413013 0.26061
H 4.057227 -0.549924 1.211695
H 3.789248 -1.663501 -0.901584
H 2.976112 1.582826 -1.982106
H 2.717333 3.145914 -0.255503
H 0.415923 3.08236 0.364555
H 0.501156 2.180155 -1.186808
H 3.615876 -2.255008 1.454578
Na -0.298006 -1.709369 -1.215834

4. Transition States of 1st Glu-L1 C-C cleavage

25

C -2.534556 -0.371417 -0.608413
C -2.512518 0.584601 0.396326
C -0.630536 0.233051 0.914068
O -0.490121 -1.028932 1.11529
C 0.192992 0.885615 -0.182905
C 1.616764 1.245584 0.293581
C 2.379226 0.143705 1.023254
O 2.639427 -0.964504 0.162184
O 2.341498 1.568756 -0.890804
O 0.244464 -0.02567 -1.276454
O -2.570152 1.907046 0.031256
O -2.339143 -1.62189 -0.33341
H 1.537596 2.1097 0.961465
H -2.572817 -0.090656 -1.659179
H -2.881939 0.307734 1.37985
H -0.768107 0.885398 1.786064
H -0.294961 1.813485 -0.497107
H -3.337239 2.339414 0.419985
H 3.323956 0.549548 1.394322
H 1.812114 -0.239391 1.871642
H 0.777489 0.395017 -1.963801
H -1.753419 -1.644748 0.507459
H 3.268665 -0.668137 -0.505326
H 2.728726 2.446156 -0.836866
Na 0.771264 -2.109892 -0.394406

5. Reactant of 1st Glu-L1 C-C cleavage

25

C -2.534556 -0.371417 -0.608413
C -2.512518 0.584601 0.396326
C -0.630536 0.233051 0.914068
O -0.490121 -1.028932 1.11529
C 0.192992 0.885615 -0.182905
C 1.616764 1.245584 0.293581
C 2.379226 0.143705 1.023254
O 2.639427 -0.964504 0.162184
O 2.341498 1.568756 -0.890804
O 0.244464 -0.02567 -1.276454
O -2.570152 1.907046 0.031256
O -2.339143 -1.62189 -0.33341
H 1.537596 2.1097 0.961465
H -2.572817 -0.090656 -1.659179
H -2.881939 0.307734 1.37985
H -0.768107 0.885398 1.786064
H -0.294961 1.813485 -0.497107
H -3.337239 2.339414 0.419985
H 3.323956 0.549548 1.394322
H 1.812114 -0.239391 1.871642
H 0.777489 0.395017 -1.963801
H -1.753419 -1.644748 0.507459
H 3.268665 -0.668137 -0.505326
H 2.728726 2.446156 -0.836866
Na 0.771264 -2.109892 -0.394406

6. Transition States of 2nd Glu-L1 C-C cleavage

25

C 2.534757 0.371582 -0.608651
C 2.512481 -0.584316 0.396284
C 0.630869 -0.233174 0.914269
O 0.490042 1.028755 1.115551
C -0.192683 -0.885838 -0.1827
C -1.616729 -1.245729 0.293576
C -2.378949 -0.143801 1.023258
O -2.640017 0.96383 0.161608
O -2.3437 -1.567554 -0.889865
O -0.244247 0.02555 -1.276093
O 2.570352 -1.906847 0.031283
O 2.33933 1.622111 -0.334
H -1.538335 -2.109904 0.961423
H 2.573282 0.090652 -1.659359
H 2.882266 -0.307376 1.379663
H 0.768158 -0.885486 1.786357
H 0.295505 -1.813605 -0.496946
H 3.338295 -2.338709 0.418897
H -3.323299 -0.549853 1.394885
H -1.811363 0.239939 1.871003
H -0.779682 -0.394046 -1.96224
H 1.754442 1.64539 0.50723
H -3.271547 0.667605 -0.5038
H -2.711458 -2.453809 -0.8457
Na -0.77171 2.11001 -0.394136

7. Reactant of 2nd Glu-L1 C-C cleavage

25

C   2.534757   0.371582   -0.608651
C   2.512481   -0.584316   0.396284
C   0.630869   -0.233174   0.914269
O   0.490042   1.028755   1.115551
C   -0.192683   -0.885838   -0.1827
C   -1.616729   -1.245729   0.293576
C   -2.378949   -0.143801   1.023258
O   -2.640017   0.96383   0.161608
O   -2.3437   -1.567554   -0.889865
O   -0.244247   0.02555   -1.276093
O   2.570352   -1.906847   0.031283
O   2.33933   1.622111   -0.334
H   -1.538335   -2.109904   0.961423
H   2.573282   0.090652   -1.659359
H   2.882266   -0.307376   1.379663
H   0.768158   -0.885486   1.786357
H   0.295505   -1.813605   -0.496946
H   3.338295   -2.338709   0.418897
H   -3.323299   -0.549853   1.394885
H   -1.811363   0.239939   1.871003
H   -0.779682   -0.394046   -1.96224
H   1.754442   1.64539   0.50723
H   -3.271547   0.667605   -0.5038
H   -2.711458   -2.453809   -0.8457
Na   -0.77171   2.11001   -0.394136

8. Transition States of 3rd Glu-L1 C-C cleavage

25

C -2.540314 -1.341107 -0.30611
C -2.095122 -0.803081 0.890124
C -0.336025 -0.508512 -0.16891
O -0.668648 0.324587 -1.106657
C 0.494573 0.088777 0.981319
C 1.996196 0.109418 0.626818
C 2.668477 -1.254813 0.55862
O 2.274412 -1.840969 -0.673639
O 2.197355 0.807839 -0.597471
O 0.151759 1.447969 1.189471
O -2.417316 0.48132 1.229368
O -2.86399 -0.539837 -1.263562
H 2.46951 0.697062 1.417543
H -2.474753 -2.414186 -0.488096
H -1.830508 -1.431038 1.731528
H -0.034815 -1.518883 -0.452542
H 0.377427 -0.490951 1.901852
H -3.044367 0.827553 0.577485
H 3.751615 -1.109891 0.588846
H 2.369963 -1.870052 1.413274
H -0.760671 1.479369 1.512012
H -1.89026 0.021547 -1.416877
H 2.841244 -2.587192 -0.887546
H 2.31174 0.13602 -1.286496
Na 0.490782 2.279025 -0.938384

9. Reactant of 3rd Glu-L1 C-C cleavage

25

C -2.540314 -1.341107 -0.30611
C -2.095122 -0.803081 0.890124
C -0.336025 -0.508512 -0.16891
O -0.668648 0.324587 -1.106657
C 0.494573 0.088777 0.981319
C 1.996196 0.109418 0.626818
C 2.668477 -1.254813 0.55862
O 2.274412 -1.840969 -0.673639
O 2.197355 0.807839 -0.597471
O 0.151759 1.447969 1.189471
O -2.417316 0.48132 1.229368
O -2.86399 -0.539837 -1.263562
H 2.46951 0.697062 1.417543
H -2.474753 -2.414186 -0.488096
H -1.830508 -1.431038 1.731528
H -0.034815 -1.518883 -0.452542
H 0.377427 -0.490951 1.901852
H -3.044367 0.827553 0.577485
H 3.751615 -1.109891 0.588846
H 2.369963 -1.870052 1.413274
H -0.760671 1.479369 1.512012
H -1.89026 0.021547 -1.416877
H 2.841244 -2.587192 -0.887546
H 2.31174 0.13602 -1.286496
Na 0.490782 2.279025 -0.938384

10. Transition States of 4th Glu-L1 C-C cleavage

25

C 2.797231 0.130675 0.26945
C 2.218281 1.208199 -0.381298
C 0.461375 0.31069 -0.708572
O 0.684528 -0.837161 -1.22727
C -0.233343 0.378234 0.648988
C -1.753558 0.219956 0.543884
C -2.447446 1.252377 -0.329243
O -3.809612 0.873761 -0.283712
O -2.035196 -1.085528 0.046572
O 0.296472 -0.702896 1.418427
O 1.934881 2.3365 0.353851
O 2.944926 -1.006679 -0.327531
H -2.169982 0.306731 1.556365
H 3.024946 0.168059 1.333485
H 2.392638 1.323239 -1.44716
H 0.191382 1.141786 -1.370691
H -0.009103 1.342374 1.114596
H 2.444983 3.090187 0.039975
H -2.286098 2.257398 0.075422
H -2.066914 1.203444 -1.355377
H 0.119815 -0.55634 2.353028
H 2.186876 -1.08536 -1.009448
H -4.341307 1.439159 -0.849828
H -2.977687 -1.102291 -0.170618
Na -0.262981 -2.468468 -0.033792

11. Reactant of 4th Glu-L1 C-C cleavage

25

C 2.797231 0.130675 0.26945
C 2.218281 1.208199 -0.381298
C 0.461375 0.31069 -0.708572
O 0.684528 -0.837161 -1.22727
C -0.233343 0.378234 0.648988
C -1.753558 0.219956 0.543884
C -2.447446 1.252377 -0.329243
O -3.809612 0.873761 -0.283712
O -2.035196 -1.085528 0.046572
O 0.296472 -0.702896 1.418427
O 1.934881 2.3365 0.353851
O 2.944926 -1.006679 -0.327531
H -2.169982 0.306731 1.556365
H 3.024946 0.168059 1.333485
H 2.392638 1.323239 -1.44716
H 0.191382 1.141786 -1.370691
H -0.009103 1.342374 1.114596
H 2.444983 3.090187 0.039975
H -2.286098 2.257398 0.075422
H -2.066914 1.203444 -1.355377
H 0.119815 -0.55634 2.353028
H 2.186876 -1.08536 -1.009448
H -4.341307 1.439159 -0.849828
H -2.977687 -1.102291 -0.170618
Na -0.262981 -2.468468 -0.033792

12. Transition States of 1st Glu-L1 C=O Migration

25

C -2.85358 0.298166 0.083878
C -1.432283 0.059342 0.050178
C -0.520683 1.060901 0.784535
O -0.023803 0.501836 1.981921
C 0.672735 1.599221 -0.023846
C 1.830337 0.679592 -0.451184
C 2.520294 -0.144465 0.632255
O 1.775164 -1.286666 1.017276
O 1.422273 -0.210115 -1.48205
O 0.099868 2.138897 -1.210756
O -1.111048 -1.178979 -0.154291
O -3.600913 -0.693109 -0.234962
H 2.592288 1.361117 -0.853913
H -3.333713 1.259557 0.242161
H -1.728425 0.703123 -0.963278
H -1.123403 1.951841 0.999523
H 1.108894 2.387842 0.598629
H -2.977006 -1.465354 -0.284943
H 3.463163 -0.513713 0.227508
H 2.743844 0.489419 1.494858
H 0.452965 3.012859 -1.399317
H -0.697828 0.499293 2.66856
H 1.070756 -0.983695 1.608481
H 1.014541 0.317487 -2.179353
Na 0.692788 -2.228294 -0.722811

13. Reactant of 1st Glu-L1 C=O Migration

25

C	-2.85358	0.298166	0.083878
C	-1.432283	0.059342	0.050178
C	-0.520683	1.060901	0.784535
O	-0.023803	0.501836	1.981921
C	0.672735	1.599221	-0.023846
C	1.830337	0.679592	-0.451184
C	2.520294	-0.144465	0.632255
O	1.775164	-1.286666	1.017276
O	1.422273	-0.210115	-1.48205
O	0.099868	2.138897	-1.210756
O	-1.111048	-1.178979	-0.154291
O	-3.600913	-0.693109	-0.234962
H	2.592288	1.361117	-0.853913
H	-3.333713	1.259557	0.242161
H	-1.728425	0.703123	-0.963278
H	-1.123403	1.951841	0.999523
H	1.108894	2.387842	0.598629
H	-2.977006	-1.465354	-0.284943
H	3.463163	-0.513713	0.227508
H	2.743844	0.489419	1.494858
H	0.452965	3.012859	-1.399317
H	-0.697828	0.499293	2.66856
H	1.070756	-0.983695	1.608481
H	1.014541	0.317487	-2.179353
Na	0.692788	-2.228294	-0.722811

14. Transition States of 2nd Glu-L1 C=O Migration

25

C 2.73998 -1.270738 -0.501821
C 1.743881 -0.235984 -0.386097
C 0.31008 -0.603997 0.003956
O 0.243667 -0.306997 1.387481
C -0.730416 0.193631 -0.800454
C -2.146341 -0.397329 -0.812733
C -2.807922 -0.554564 0.553492
O -2.109576 -1.555953 1.291614
O -2.874719 0.551331 -1.572551
O -0.77774 1.519542 -0.289698
O 2.210008 0.940357 -0.127266
O 3.96069 -0.880533 -0.492957
H -2.109118 -1.373414 -1.311172
H 2.544971 -2.319803 -0.70321
H 1.826518 -0.580933 -1.584867
H 0.140916 -1.672662 -0.176142
H -0.407389 0.219781 -1.848683
H 3.919759 0.082339 -0.252582
H -2.791753 0.401789 1.084957
H -3.849544 -0.848996 0.401174
H -1.461086 1.990015 -0.785346
H -0.526063 -0.784523 1.741541
H -2.705676 -2.010546 1.892498
H -3.722894 0.199066 -1.855944
Na 0.842467 1.979071 1.226067

15. Reactant of 2nd Glu-L1 C=O Migration

25

C 2.73998 -1.270738 -0.501821
C 1.743881 -0.235984 -0.386097
C 0.31008 -0.603997 0.003956
O 0.243667 -0.306997 1.387481
C -0.730416 0.193631 -0.800454
C -2.146341 -0.397329 -0.812733
C -2.807922 -0.554564 0.553492
O -2.109576 -1.555953 1.291614
O -2.874719 0.551331 -1.572551
O -0.77774 1.519542 -0.289698
O 2.210008 0.940357 -0.127266
O 3.96069 -0.880533 -0.492957
H -2.109118 -1.373414 -1.311172
H 2.544971 -2.319803 -0.70321
H 1.826518 -0.580933 -1.584867
H 0.140916 -1.672662 -0.176142
H -0.407389 0.219781 -1.848683
H 3.919759 0.082339 -0.252582
H -2.791753 0.401789 1.084957
H -3.849544 -0.848996 0.401174
H -1.461086 1.990015 -0.785346
H -0.526063 -0.784523 1.741541
H -2.705676 -2.010546 1.892498
H -3.722894 0.199066 -1.855944
Na 0.842467 1.979071 1.226067

16. Transition States of 3rd Glu-L1 C=O Migration

25

C	2.826022	-1.029236	0.004034
C	1.588521	-0.304856	-0.055834
C	0.581208	-0.377712	1.095529
O	0.607149	0.917305	1.681846
C	-0.815713	-0.8084	0.610242
C	-1.369844	-0.01996	-0.589901
C	-2.730301	-0.539318	-1.020192
O	-3.591337	-0.288038	0.082521
O	-1.494123	1.3661	-0.26448
O	-0.641214	-2.162832	0.233114
O	1.589273	0.727443	-0.844585
O	3.731519	-0.681053	-0.835707
H	-0.669809	-0.096105	-1.424457
H	3.011378	-1.910653	0.610559
H	1.42158	-1.395628	-0.623512
H	0.902698	-1.134719	1.818071
H	-1.514557	-0.712904	1.449606
H	3.389823	0.149476	-1.255448
H	-3.054315	0.012032	-1.907713
H	-2.676641	-1.603539	-1.264187
H	-1.410374	-2.686484	0.47247
H	0.205667	0.882791	2.555419
H	-4.506782	-0.449704	-0.159619
H	-2.357392	1.478017	0.157334
Na	0.46993	2.446628	-0.101224

17. Reactant of 3rd Glu-L1 C=O Migration

25

C 2.826022 -1.029236 0.004034
C 1.588521 -0.304856 -0.055834
C 0.581208 -0.377712 1.095529
O 0.607149 0.917305 1.681846
C -0.815713 -0.8084 0.610242
C -1.369844 -0.01996 -0.589901
C -2.730301 -0.539318 -1.020192
O -3.591337 -0.288038 0.082521
O -1.494123 1.3661 -0.26448
O -0.641214 -2.162832 0.233114
O 1.589273 0.727443 -0.844585
O 3.731519 -0.681053 -0.835707
H -0.669809 -0.096105 -1.424457
H 3.011378 -1.910653 0.610559
H 1.42158 -1.395628 -0.623512
H 0.902698 -1.134719 1.818071
H -1.514557 -0.712904 1.449606
H 3.389823 0.149476 -1.255448
H -3.054315 0.012032 -1.907713
H -2.676641 -1.603539 -1.264187
H -1.410374 -2.686484 0.47247
H 0.205667 0.882791 2.555419
H -4.506782 -0.449704 -0.159619
H -2.357392 1.478017 0.157334
Na 0.46993 2.446628 -0.101224

18. Transition States of 4th Glu-L1 C=O Migration

25

C	-2.443265	0.103965	0.700938
C	-1.570182	0.008845	-0.447818
C	-0.736006	1.226448	-0.859459
O	-1.280776	2.336349	-0.176305
C	0.770655	1.127329	-0.575037
C	1.221169	0.858083	0.865777
C	0.91901	-0.511209	1.472707
O	1.618863	-1.553861	0.800357
O	2.645406	0.974036	0.828265
O	1.355978	0.20466	-1.49031
O	-1.197863	-1.20263	-0.739415
O	-2.869951	-1.014188	1.160882
H	0.770614	1.629253	1.499814
H	-2.851255	1.02203	1.108451
H	-2.675158	0.307359	-0.875974
H	-0.825218	1.331333	-1.945186
H	1.1523	2.128907	-0.809507
H	-2.365416	-1.705759	0.653722
H	1.21485	-0.492126	2.525495
H	-0.137384	-0.765355	1.425673
H	2.303472	0.38754	-1.496005
H	-1.219134	3.131735	-0.712543
H	2.55997	-1.402612	0.946962
H	2.958929	1.634263	1.451278
Na	0.724718	-2.003842	-1.252966

19. Reactant of 4th Glu-L1 C=O Migration

25

C -2.443265 0.103965 0.700938
C -1.570182 0.008845 -0.447818
C -0.736006 1.226448 -0.859459
O -1.280776 2.336349 -0.176305
C 0.770655 1.127329 -0.575037
C 1.221169 0.858083 0.865777
C 0.91901 -0.511209 1.472707
O 1.618863 -1.553861 0.800357
O 2.645406 0.974036 0.828265
O 1.355978 0.20466 -1.49031
O -1.197863 -1.20263 -0.739415
O -2.869951 -1.014188 1.160882
H 0.770614 1.629253 1.499814
H -2.851255 1.02203 1.108451
H -2.675158 0.307359 -0.875974
H -0.825218 1.331333 -1.945186
H 1.1523 2.128907 -0.809507
H -2.365416 -1.705759 0.653722
H 1.21485 -0.492126 2.525495
H -0.137384 -0.765355 1.425673
H 2.303472 0.38754 -1.496005
H -1.219134 3.131735 -0.712543
H 2.55997 -1.402612 0.946962
H 2.958929 1.634263 1.451278
Na 0.724718 -2.003842 -1.252966

20. Transition States of 1st Glu-L2 C-C cleavage

25

C	-1.247893	-1.686779	0.609806
C	-1.610671	-0.287232	0.177743
C	-1.358482	0.866752	0.918288
O	-0.707462	0.754384	2.140271
C	-0.177214	1.817708	-0.470143
C	1.253312	1.287427	-0.542376
C	1.98226	0.935779	0.744236
O	1.59075	-0.33933	1.243701
O	1.303688	0.164926	-1.406216
O	-0.835978	1.714036	-1.560492
O	-2.110127	-0.186103	-1.002738
O	-0.542663	-2.37078	-0.415938
H	1.803014	2.12707	-0.996996
H	-2.183602	-2.207277	0.843633
H	-2.058052	1.688963	0.817912
H	-0.272487	2.731757	0.128373
H	3.05267	0.883745	0.53822
H	1.820222	1.71526	1.492149
H	-1.699637	0.744506	-1.437318
H	-1.238467	1.12487	2.852705
H	0.910892	-0.190017	1.914231
H	0.748502	0.379086	-2.170783
H	-1.112547	-2.416754	-1.193505
H	-0.630967	-1.691259	1.504004
Na	1.656993	-1.85354	-0.446059

21. Reactant of 1st Glu-L2 C-C cleavage

25

C -1.247893 -1.686779 0.609806
C -1.610671 -0.287232 0.177743
C -1.358482 0.866752 0.918288
O -0.707462 0.754384 2.140271
C -0.177214 1.817708 -0.470143
C 1.253312 1.287427 -0.542376
C 1.98226 0.935779 0.744236
O 1.59075 -0.33933 1.243701
O 1.303688 0.164926 -1.406216
O -0.835978 1.714036 -1.560492
O -2.110127 -0.186103 -1.002738
O -0.542663 -2.37078 -0.415938
H 1.803014 2.12707 -0.996996
H -2.183602 -2.207277 0.843633
H -2.058052 1.688963 0.817912
H -0.272487 2.731757 0.128373
H 3.05267 0.883745 0.53822
H 1.820222 1.71526 1.492149
H -1.699637 0.744506 -1.437318
H -1.238467 1.12487 2.852705
H 0.910892 -0.190017 1.914231
H 0.748502 0.379086 -2.170783
H -1.112547 -2.416754 -1.193505
H -0.630967 -1.691259 1.504004
Na 1.656993 -1.85354 -0.446059

22. Transition States of 2nd Glu-L2 C-C cleavage

25

C 0.955775 -1.581452 1.015749
C 0.839886 -1.197444 -0.435586
C -0.312757 -1.308981 -1.21339
O -1.468904 -1.902057 -0.834991
C -0.522903 0.740936 -1.361723
C -1.164455 1.367219 -0.122856
C -2.457256 0.752178 0.401618
O -2.102706 -0.333448 1.268369
O -0.209797 1.491099 0.914834
O 0.642617 1.21096 -1.661123
O 1.873988 -0.617143 -0.965495
O 1.616255 -0.527466 1.716409
H -1.408846 2.382077 -0.463588
H 1.551669 -2.500073 1.050525
H -0.148544 -1.374453 -2.284188
H -1.229499 0.551886 -2.177791
H -3.002949 1.5058 0.971432
H -3.079676 0.400656 -0.425573
H 1.451417 0.22282 -1.570134
H -1.708424 -1.648044 0.07005
H -2.815094 -0.49978 1.894176
H -0.41623 0.832792 1.593535
H 2.066429 -0.888347 2.485778
H -0.023188 -1.782744 1.453047
Na 1.99287 1.41701 0.38144

23. Reactant of 2nd Glu-L2 C-C cleavage

25

C 0.955775 -1.581452 1.015749
C 0.839886 -1.197444 -0.435586
C -0.312757 -1.308981 -1.21339
O -1.468904 -1.902057 -0.834991
C -0.522903 0.740936 -1.361723
C -1.164455 1.367219 -0.122856
C -2.457256 0.752178 0.401618
O -2.102706 -0.333448 1.268369
O -0.209797 1.491099 0.914834
O 0.642617 1.21096 -1.661123
O 1.873988 -0.617143 -0.965495
O 1.616255 -0.527466 1.716409
H -1.408846 2.382077 -0.463588
H 1.551669 -2.500073 1.050525
H -0.148544 -1.374453 -2.284188
H -1.229499 0.551886 -2.177791
H -3.002949 1.5058 0.971432
H -3.079676 0.400656 -0.425573
H 1.451417 0.22282 -1.570134
H -1.708424 -1.648044 0.07005
H -2.815094 -0.49978 1.894176
H -0.41623 0.832792 1.593535
H 2.066429 -0.888347 2.485778
H -0.023188 -1.782744 1.453047
Na 1.99287 1.41701 0.38144

24. Transition States of 3rd Glu-L2 C-C cleavage

25

C 0.958125 1.581624 -1.01518
C 0.841664 1.197082 0.43588
C -0.311379 1.308073 1.213447
O -1.466922 1.902483 0.835083
C -0.522772 -0.74047 1.361269
C -1.165709 -1.366251 0.122826
C -2.458562 -0.749731 -0.400073
O -2.103599 0.335199 -1.267654
O -0.212238 -1.489904 -0.916038
O 0.642611 -1.211884 1.659797
O 1.875296 0.616094 0.965494
O 1.618277 0.527383 -1.715575
H -1.410489 -2.381109 0.463211
H 1.554633 2.499895 -1.04961
H -0.146989 1.373925 2.2842
H -1.228813 -0.552055 2.177973
H -3.006398 -1.502569 -0.968842
H -3.079108 -0.39683 0.427892
H 1.452653 -0.223647 1.570033
H -1.706944 1.64863 -0.069876
H -2.815404 0.500677 -1.89434
H -0.420011 -0.831482 -1.594207
H 2.062885 0.886807 -2.48886
H -0.020672 1.783734 -1.452592
Na 1.989914 -1.420263 -0.382078

25. Reactant of 3rd Glu-L2 C-C cleavage

25

C 0.958125 1.581624 -1.01518
C 0.841664 1.197082 0.43588
C -0.311379 1.308073 1.213447
O -1.466922 1.902483 0.835083
C -0.522772 -0.74047 1.361269
C -1.165709 -1.366251 0.122826
C -2.458562 -0.749731 -0.400073
O -2.103599 0.335199 -1.267654
O -0.212238 -1.489904 -0.916038
O 0.642611 -1.211884 1.659797
O 1.875296 0.616094 0.965494
O 1.618277 0.527383 -1.715575
H -1.410489 -2.381109 0.463211
H 1.554633 2.499895 -1.04961
H -0.146989 1.373925 2.2842
H -1.228813 -0.552055 2.177973
H -3.006398 -1.502569 -0.968842
H -3.079108 -0.39683 0.427892
H 1.452653 -0.223647 1.570033
H -1.706944 1.64863 -0.069876
H -2.815404 0.500677 -1.89434
H -0.420011 -0.831482 -1.594207
H 2.062885 0.886807 -2.48886
H -0.020672 1.783734 -1.452592
Na 1.989914 -1.420263 -0.382078

26. Transition States of 4th Glu-L2 C-C cleavage

25

C 1.025773 1.555284 -0.964066
C 0.847496 1.154564 0.477714
C -0.338333 1.284272 1.192734
O -1.434131 1.899753 0.623133
C -0.639425 -0.781155 1.352969
C -1.206203 -1.366071 0.066311
C -2.471148 -0.730514 -0.524303
O -2.121976 0.279162 -1.45007
O -0.188683 -1.414807 -0.917589
O 0.507 -1.244279 1.713674
O 1.827573 0.530779 1.045977
O 1.808266 0.557082 -1.609332
H -1.44801 -2.397874 0.357878
H 1.548117 2.519045 -0.964814
H -0.258246 1.341565 2.273438
H -1.382618 -0.57788 2.13285
H -3.017349 -1.492553 -1.080433
H -3.119476 -0.354024 0.271133
H 1.341119 -0.302187 1.632005
H -1.762639 2.605477 1.189803
H -2.118262 1.123147 -0.984434
H -0.461781 -0.808064 -1.625147
H 2.220819 0.928317 -2.394385
H 0.05831 1.678308 -1.452723
Na 1.991515 -1.438462 -0.382697

27. Reactant of 4th Glu-L2 C-C cleavage

25

C 1.025773 1.555284 -0.964066
C 0.847496 1.154564 0.477714
C -0.338333 1.284272 1.192734
O -1.434131 1.899753 0.623133
C -0.639425 -0.781155 1.352969
C -1.206203 -1.366071 0.066311
C -2.471148 -0.730514 -0.524303
O -2.121976 0.279162 -1.45007
O -0.188683 -1.414807 -0.917589
O 0.507 -1.244279 1.713674
O 1.827573 0.530779 1.045977
O 1.808266 0.557082 -1.609332
H -1.44801 -2.397874 0.357878
H 1.548117 2.519045 -0.964814
H -0.258246 1.341565 2.273438
H -1.382618 -0.57788 2.13285
H -3.017349 -1.492553 -1.080433
H -3.119476 -0.354024 0.271133
H 1.341119 -0.302187 1.632005
H -1.762639 2.605477 1.189803
H -2.118262 1.123147 -0.984434
H -0.461781 -0.808064 -1.625147
H 2.220819 0.928317 -2.394385
H 0.05831 1.678308 -1.452723
Na 1.991515 -1.438462 -0.382697

28. Transition States of 1st Glu-L2 C=O Migration

25

C 3.106448 0.632834 0.142012
C 2.166396 -0.514267 -0.079262
C 0.725621 -0.528268 -0.062114
O 0.189489 -1.680582 -0.287468
C -0.114951 0.569731 0.607751
C -1.003205 1.332161 -0.389798
C -1.783434 0.463539 -1.373823
O -2.753849 -0.336556 -0.701485
O -1.943099 2.049361 0.406146
O -0.910246 -0.109876 1.572753
O 2.717734 -1.643344 -0.396339
O 2.411688 1.835964 -0.084421
H -0.352994 2.010245 -0.953022
H 3.956042 0.50009 -0.534221
H 1.190304 0.030391 -1.113753
H 0.528561 1.2938 1.107432
H -2.280264 1.108167 -2.103994
H -1.12674 -0.222839 -1.909498
H -1.451488 0.567476 1.99855
H 1.977558 -2.299711 -0.415756
H -3.405289 0.263704 -0.320739
H -1.949698 2.98305 0.181734
H 3.022685 2.576307 -0.027617
H 3.469571 0.550333 1.175073
Na -1.804841 -2.001376 0.524883

29. Reactant of 1st Glu-L2 C=O Migration

25

C 3.106448 0.632834 0.142012
C 2.166396 -0.514267 -0.079262
C 0.725621 -0.528268 -0.062114
O 0.189489 -1.680582 -0.287468
C -0.114951 0.569731 0.607751
C -1.003205 1.332161 -0.389798
C -1.783434 0.463539 -1.373823
O -2.753849 -0.336556 -0.701485
O -1.943099 2.049361 0.406146
O -0.910246 -0.109876 1.572753
O 2.717734 -1.643344 -0.396339
O 2.411688 1.835964 -0.084421
H -0.352994 2.010245 -0.953022
H 3.956042 0.50009 -0.534221
H 1.190304 0.030391 -1.113753
H 0.528561 1.2938 1.107432
H -2.280264 1.108167 -2.103994
H -1.12674 -0.222839 -1.909498
H -1.451488 0.567476 1.99855
H 1.977558 -2.299711 -0.415756
H -3.405289 0.263704 -0.320739
H -1.949698 2.98305 0.181734
H 3.022685 2.576307 -0.027617
H 3.469571 0.550333 1.175073
Na -1.804841 -2.001376 0.524883

30. Transition States of 2nd Glu-L2 C=O Migration

25

C 2.920737 -0.105282 0.81733
C 1.865658 0.803688 0.242595
C 0.484045 0.473412 -0.025896
O -0.154055 1.373163 -0.693454
C 0.099084 -1.014549 -0.003295
C -1.391612 -1.322273 0.177312
C -2.04604 -0.785805 1.434006
O -2.178904 0.634001 1.337502
O -2.138516 -0.867537 -0.943777
O 0.421165 -1.588491 -1.247951
O 2.212066 1.997982 -0.102906
O 3.029418 -1.178379 -0.103318
H -1.455881 -2.415269 0.231846
H 2.63669 -0.462004 1.811518
H 0.641946 0.764234 1.197981
H 0.640705 -1.514578 0.810434
H -3.031592 -1.246963 1.535463
H -1.436502 -1.057289 2.302028
H 1.381478 -1.670615 -1.310861
H 1.425391 2.348106 -0.60127
H -2.555902 0.971624 2.154951
H -1.727355 -1.253304 -1.729205
H 3.885352 -1.608272 -0.013051
H 3.845292 0.469495 0.894524
Na -2.306386 1.401252 -0.827767

31. Reactant of 2nd Glu-L2 C=O Migration

25

C 2.920737 -0.105282 0.81733
C 1.865658 0.803688 0.242595
C 0.484045 0.473412 -0.025896
O -0.154055 1.373163 -0.693454
C 0.099084 -1.014549 -0.003295
C -1.391612 -1.322273 0.177312
C -2.04604 -0.785805 1.434006
O -2.178904 0.634001 1.337502
O -2.138516 -0.867537 -0.943777
O 0.421165 -1.588491 -1.247951
O 2.212066 1.997982 -0.102906
O 3.029418 -1.178379 -0.103318
H -1.455881 -2.415269 0.231846
H 2.63669 -0.462004 1.811518
H 0.641946 0.764234 1.197981
H 0.640705 -1.514578 0.810434
H -3.031592 -1.246963 1.535463
H -1.436502 -1.057289 2.302028
H 1.381478 -1.670615 -1.310861
H 1.425391 2.348106 -0.60127
H -2.555902 0.971624 2.154951
H -1.727355 -1.253304 -1.729205
H 3.885352 -1.608272 -0.013051
H 3.845292 0.469495 0.894524
Na -2.306386 1.401252 -0.827767

32. Transition States of 3rd Glu-L2 C=O Migration

25

C	-2.969965	-0.469081	-0.143643
C	-1.931219	0.613857	-0.062391
C	-0.5356	0.510453	0.24543
O	0.117704	1.628759	0.274049
C	0.05483	-0.694251	0.981644
C	1.392639	-1.207655	0.413858
C	1.396781	-1.485877	-1.077407
O	1.412746	-0.234425	-1.779315
O	2.456488	-0.296704	0.686822
O	0.253337	-0.298721	2.320705
O	-2.35457	1.812459	-0.344915
O	-2.344951	-1.67831	-0.50626
H	1.596097	-2.155574	0.922232
H	-3.718554	-0.14828	-0.873662
H	-0.82956	0.105493	-0.935299
H	-0.65043	-1.523914	0.976253
H	2.287458	-2.062692	-1.33455
H	0.500535	-2.057897	-1.339801
H	0.453382	0.646171	2.324706
H	-1.612879	2.419662	-0.116609
H	1.418045	-0.406266	-2.72546
H	2.64311	-0.334888	1.631214
H	-3.005702	-2.37464	-0.566457
H	-3.446353	-0.528757	0.843404
Na	2.144912	1.577495	-0.559963

33. Reactant of 3rd Glu-L2 C=O Migration

25

C -2.969965 -0.469081 -0.143643
C -1.931219 0.613857 -0.062391
C -0.5356 0.510453 0.24543
O 0.117704 1.628759 0.274049
C 0.05483 -0.694251 0.981644
C 1.392639 -1.207655 0.413858
C 1.396781 -1.485877 -1.077407
O 1.412746 -0.234425 -1.779315
O 2.456488 -0.296704 0.686822
O 0.253337 -0.298721 2.320705
O -2.35457 1.812459 -0.344915
O -2.344951 -1.67831 -0.50626
H 1.596097 -2.155574 0.922232
H -3.718554 -0.14828 -0.873662
H -0.82956 0.105493 -0.935299
H -0.65043 -1.523914 0.976253
H 2.287458 -2.062692 -1.33455
H 0.500535 -2.057897 -1.339801
H 0.453382 0.646171 2.324706
H -1.612879 2.419662 -0.116609
H 1.418045 -0.406266 -2.72546
H 2.64311 -0.334888 1.631214
H -3.005702 -2.37464 -0.566457
H -3.446353 -0.528757 0.843404
Na 2.144912 1.577495 -0.559963

34. Transition States of 4th Glu-L2 C=O Migration

25

C 2.88152 0.01886 0.369191
C 1.781692 -0.8142 -0.214946
C 0.353612 -0.680991 -0.11607
O -0.312797 -1.588517 -0.765298
C -0.378241 0.099577 1.001119
C -1.346537 1.164986 0.471998
C -0.665089 2.362549 -0.177775
O -0.220474 1.928935 -1.459962
O -2.277939 0.586431 -0.436587
O -1.206941 -0.835929 1.692122
O 2.175802 -1.750822 -1.024525
O 2.336952 1.199221 0.909462
H -1.917073 1.512126 1.336599
H 3.607302 0.216016 -0.426126
H 0.836986 0.210138 -0.854145
H 0.327069 0.573649 1.682525
H -1.394845 3.169373 -0.280303
H 0.173718 2.699079 0.437705
H -0.691735 -1.323868 2.341266
H 1.345827 -2.239834 -1.263097
H 0.016614 2.682652 -2.00714
H -1.926953 0.740173 -1.326318
H 3.047554 1.751749 1.247564
H 3.372228 -0.600043 1.131328
Na -2.410479 -1.691857 -0.12206

35. Reactant of 4th Glu-L2 C=O Migration

25

C 2.88152 0.01886 0.369191
C 1.781692 -0.8142 -0.214946
C 0.353612 -0.680991 -0.11607
O -0.312797 -1.588517 -0.765298
C -0.378241 0.099577 1.001119
C -1.346537 1.164986 0.471998
C -0.665089 2.362549 -0.177775
O -0.220474 1.928935 -1.459962
O -2.277939 0.586431 -0.436587
O -1.206941 -0.835929 1.692122
O 2.175802 -1.750822 -1.024525
O 2.336952 1.199221 0.909462
H -1.917073 1.512126 1.336599
H 3.607302 0.216016 -0.426126
H 0.836986 0.210138 -0.854145
H 0.327069 0.573649 1.682525
H -1.394845 3.169373 -0.280303
H 0.173718 2.699079 0.437705
H -0.691735 -1.323868 2.341266
H 1.345827 -2.239834 -1.263097
H 0.016614 2.682652 -2.00714
H -1.926953 0.740173 -1.326318
H 3.047554 1.751749 1.247564
H 3.372228 -0.600043 1.131328
Na -2.410479 -1.691857 -0.12206

36. Transition States of 1st Glu-L3a C-C cleavage

25

C 2.183657 1.568777 0.223824
C 1.159165 0.617872 0.822333
C 0.24689 0.082552 -0.273668
O 0.5092 -1.092679 -0.779248
C -0.908694 0.746451 -0.604665
C -1.962697 -0.814449 0.485911
C -3.313321 -0.564489 -0.148825
O -3.624717 0.813989 -0.082613
O -1.413422 -1.940691 0.247793
O -1.150696 1.944915 -0.019113
O 1.887954 -0.449445 1.422921
O 2.947617 0.806974 -0.718233
H -1.797802 -0.30998 1.447315
H 2.831261 1.950257 1.016285
H -1.433159 0.497984 -1.522297
H -3.304547 -0.939409 -1.176575
H -4.027364 -1.163116 0.430747
H -2.106431 2.099659 -0.045499
H -4.568865 0.949192 -0.197959
H -0.389409 -1.744333 -0.346941
H 3.531287 1.38968 -1.211639
H 1.663702 2.393159 -0.269988
H 1.365233 -0.861855 2.117333
H 0.567996 1.16985 1.557827
Na 2.726419 -1.446898 -0.490921

37. Reactant of 1st Glu-L3a C-C cleavage

25

C 2.183657 1.568777 0.223824
C 1.159165 0.617872 0.822333
C 0.24689 0.082552 -0.273668
O 0.5092 -1.092679 -0.779248
C -0.908694 0.746451 -0.604665
C -1.962697 -0.814449 0.485911
C -3.313321 -0.564489 -0.148825
O -3.624717 0.813989 -0.082613
O -1.413422 -1.940691 0.247793
O -1.150696 1.944915 -0.019113
O 1.887954 -0.449445 1.422921
O 2.947617 0.806974 -0.718233
H -1.797802 -0.30998 1.447315
H 2.831261 1.950257 1.016285
H -1.433159 0.497984 -1.522297
H -3.304547 -0.939409 -1.176575
H -4.027364 -1.163116 0.430747
H -2.106431 2.099659 -0.045499
H -4.568865 0.949192 -0.197959
H -0.389409 -1.744333 -0.346941
H 3.531287 1.38968 -1.211639
H 1.663702 2.393159 -0.269988
H 1.365233 -0.861855 2.117333
H 0.567996 1.16985 1.557827
Na 2.726419 -1.446898 -0.490921

38. Transition States of 2nd Glu-L3a C-C cleavage

25

C 2.173754 1.587411 0.156559
C 1.145913 0.658153 0.793937
C 0.253423 0.070783 -0.285731
O 0.538156 -1.111946 -0.757648
C -0.89758 0.724001 -0.650281
C -1.962184 -0.846229 0.46079
C -3.331291 -0.543191 -0.106062
O -3.614125 0.836479 0.027724
O -1.449186 -1.9654 0.138958
O -1.142985 1.94168 -0.103141
O 1.806852 -0.431724 1.436034
O 2.967455 0.774811 -0.713997
H -1.736057 -0.387222 1.431907
H 2.803692 2.038378 0.928944
H -1.416126 0.444684 -1.562574
H -3.371901 -0.876566 -1.146953
H -4.033015 -1.151792 0.478392
H -2.102771 2.07331 -0.089732
H -4.560124 0.990927 -0.036931
H -0.383526 -1.766961 -0.34898
H 3.510782 1.332575 -1.277167
H 1.655356 2.373291 -0.398751
H 2.053159 -0.186949 2.332274
H 0.531907 1.228343 1.495272
Na 2.718825 -1.488072 -0.385856

39. Reactant of 2nd Glu-L3a C-C cleavage

25

C 2.173754 1.587411 0.156559
C 1.145913 0.658153 0.793937
C 0.253423 0.070783 -0.285731
O 0.538156 -1.111946 -0.757648
C -0.89758 0.724001 -0.650281
C -1.962184 -0.846229 0.46079
C -3.331291 -0.543191 -0.106062
O -3.614125 0.836479 0.027724
O -1.449186 -1.9654 0.138958
O -1.142985 1.94168 -0.103141
O 1.806852 -0.431724 1.436034
O 2.967455 0.774811 -0.713997
H -1.736057 -0.387222 1.431907
H 2.803692 2.038378 0.928944
H -1.416126 0.444684 -1.562574
H -3.371901 -0.876566 -1.146953
H -4.033015 -1.151792 0.478392
H -2.102771 2.07331 -0.089732
H -4.560124 0.990927 -0.036931
H -0.383526 -1.766961 -0.34898
H 3.510782 1.332575 -1.277167
H 1.655356 2.373291 -0.398751
H 2.053159 -0.186949 2.332274
H 0.531907 1.228343 1.495272
Na 2.718825 -1.488072 -0.385856

40. Transition States of 3rd Glu-L3a C-C cleavage

25

C 2.325314 1.466587 0.048919
C 1.23466 0.679192 0.768103
C 0.239041 0.130442 -0.240069
O 0.418318 -1.081728 -0.701779
C -0.867153 0.861827 -0.583991
C -1.89927 -0.638076 0.687712
C -3.237559 0.04916 0.576019
O -3.728431 0.081971 -0.732378
O -1.71579 -1.715004 0.032357
O -1.023473 2.107856 -0.058359
O 1.803731 -0.441745 1.444728
O 3.009086 0.541449 -0.799643
H -1.360446 -0.482015 1.632543
H 3.020839 1.894956 0.777224
H -1.497367 0.568111 -1.415507
H -3.906938 -0.49485 1.259387
H -3.169608 1.073536 0.945024
H -1.791384 2.537486 -0.448249
H -3.846325 -0.821234 -1.04661
H -0.617702 -1.652162 -0.382437
H 3.584626 1.016491 -1.405182
H 1.86604 2.270965 -0.53154
H 2.174832 -0.16158 2.286613
H 0.71665 1.34439 1.464161
Na 2.540824 -1.666479 -0.377726

41. Reactant of 3rd Glu-L3a C-C cleavage

25

C 2.325314 1.466587 0.048919
C 1.23466 0.679192 0.768103
C 0.239041 0.130442 -0.240069
O 0.418318 -1.081728 -0.701779
C -0.867153 0.861827 -0.583991
C -1.89927 -0.638076 0.687712
C -3.237559 0.04916 0.576019
O -3.728431 0.081971 -0.732378
O -1.71579 -1.715004 0.032357
O -1.023473 2.107856 -0.058359
O 1.803731 -0.441745 1.444728
O 3.009086 0.541449 -0.799643
H -1.360446 -0.482015 1.632543
H 3.020839 1.894956 0.777224
H -1.497367 0.568111 -1.415507
H -3.906938 -0.49485 1.259387
H -3.169608 1.073536 0.945024
H -1.791384 2.537486 -0.448249
H -3.846325 -0.821234 -1.04661
H -0.617702 -1.652162 -0.382437
H 3.584626 1.016491 -1.405182
H 1.86604 2.270965 -0.53154
H 2.174832 -0.16158 2.286613
H 0.71665 1.34439 1.464161
Na 2.540824 -1.666479 -0.377726

42. Transition States of 4th Glu-L3a C-C cleavage

25

C 2.325757 1.455509 0.041507
C 1.284498 0.645872 0.800089
C 0.243816 0.108163 -0.176911
O 0.380587 -1.117771 -0.614997
C -0.852273 0.859267 -0.514103
C -1.971635 -0.560648 0.720527
C -3.305157 0.103989 0.467314
O -3.706689 0.018119 -0.868022
O -1.752269 -1.687357 0.167506
O -0.964906 2.109852 0.018461
O 1.978121 -0.430207 1.425839
O 2.946269 0.557328 -0.884056
H -1.509898 -0.329153 1.691382
H 3.064802 1.845013 0.745213
H -1.484374 0.591472 -1.354155
H -4.013551 -0.390994 1.148397
H -3.270322 1.156351 0.752297
H -1.670923 2.597464 -0.416388
H -3.789571 -0.910915 -1.110271
H -0.646863 -1.643208 -0.27882
H 3.53362 1.0447 -1.468137
H 1.832348 2.277642 -0.482231
H 1.537257 -0.692189 2.238735
H 0.80932 1.301006 1.534695
Na 2.564474 -1.647256 -0.453555

43. Reactant of 4th Glu-L3a C-C cleavage

25

C 2.325757 1.455509 0.041507
C 1.284498 0.645872 0.800089
C 0.243816 0.108163 -0.176911
O 0.380587 -1.117771 -0.614997
C -0.852273 0.859267 -0.514103
C -1.971635 -0.560648 0.720527
C -3.305157 0.103989 0.467314
O -3.706689 0.018119 -0.868022
O -1.752269 -1.687357 0.167506
O -0.964906 2.109852 0.018461
O 1.978121 -0.430207 1.425839
O 2.946269 0.557328 -0.884056
H -1.509898 -0.329153 1.691382
H 3.064802 1.845013 0.745213
H -1.484374 0.591472 -1.354155
H -4.013551 -0.390994 1.148397
H -3.270322 1.156351 0.752297
H -1.670923 2.597464 -0.416388
H -3.789571 -0.910915 -1.110271
H -0.646863 -1.643208 -0.27882
H 3.53362 1.0447 -1.468137
H 1.832348 2.277642 -0.482231
H 1.537257 -0.692189 2.238735
H 0.80932 1.301006 1.534695
Na 2.564474 -1.647256 -0.453555

44. Transition States of 1st Glu-L3a C=O Migration

25

C -3.374848 0.114908 -0.224498
C -2.025631 -0.39362 -0.72364
C -0.93696 0.611404 -0.441294
O -1.149225 1.815809 -0.876222
C 0.299733 0.470053 0.290608
C 0.975313 -0.890394 0.588238
C 1.536586 -1.516184 -0.676549
O 2.493302 -0.609078 -1.228919
O 2.084546 -0.660915 1.448772
O 1.033796 1.537336 0.295092
O -1.714802 -1.643103 -0.169703
O -3.263651 0.0745 1.187363
H 0.276419 -1.597599 1.02831
H -4.154924 -0.56241 -0.583555
H -0.695226 0.547801 1.071739
H 2.018711 -2.46033 -0.411324
H 0.721364 -1.705378 -1.379329
H -0.306241 2.303683 -0.673462
H 2.854309 -0.977902 -2.039645
H 1.786036 -0.575899 2.359336
H -4.090971 0.334036 1.601572
H -3.558975 1.125252 -0.596697
H -2.213854 -1.728428 0.654073
H -2.060126 -0.487708 -1.815217
Na 3.155145 1.026499 0.241449

45. Reactant of 1st Glu-L3a C=O Migration

25

C -3.374848 0.114908 -0.224498
C -2.025631 -0.39362 -0.72364
C -0.93696 0.611404 -0.441294
O -1.149225 1.815809 -0.876222
C 0.299733 0.470053 0.290608
C 0.975313 -0.890394 0.588238
C 1.536586 -1.516184 -0.676549
O 2.493302 -0.609078 -1.228919
O 2.084546 -0.660915 1.448772
O 1.033796 1.537336 0.295092
O -1.714802 -1.643103 -0.169703
O -3.263651 0.0745 1.187363
H 0.276419 -1.597599 1.02831
H -4.154924 -0.56241 -0.583555
H -0.695226 0.547801 1.071739
H 2.018711 -2.46033 -0.411324
H 0.721364 -1.705378 -1.379329
H -0.306241 2.303683 -0.673462
H 2.854309 -0.977902 -2.039645
H 1.786036 -0.575899 2.359336
H -4.090971 0.334036 1.601572
H -3.558975 1.125252 -0.596697
H -2.213854 -1.728428 0.654073
H -2.060126 -0.487708 -1.815217
Na 3.155145 1.026499 0.241449

46. Transition States of 2nd Glu-L3a C=O Migration

25

C -3.405392 0.120879 -0.189553
C -2.07407 -0.409221 -0.713928
C -0.968156 0.588147 -0.466225
O -1.192633 1.794626 -0.889783
C 0.298461 0.448966 0.213305
C 0.973894 -0.90192 0.487858
C 1.635123 -1.460472 -0.767609
O 2.675646 -0.565431 -1.156141
O 1.971655 -0.630519 1.47209
O 1.029079 1.517279 0.186765
O -1.7722 -1.659505 -0.157529
O -3.263408 0.086873 1.219558
H 0.251399 -1.629828 0.847597
H -4.201465 -0.54871 -0.527491
H -0.664138 0.522788 1.035182
H 2.052241 -2.44642 -0.539665
H 0.886949 -1.567253 -1.55869
H -0.337894 2.277137 -0.721582
H 3.073981 -0.869842 -1.975899
H 2.108424 -1.404259 2.024824
H -4.076137 0.363607 1.650966
H -3.584795 1.131219 -0.563593
H -2.245504 -1.722359 0.683564
H -2.133594 -0.511991 -1.803571
Na 3.138752 1.067368 0.424145

47. Reactant of 2nd Glu-L3a C=O Migration

25

C -3.405392 0.120879 -0.189553
C -2.07407 -0.409221 -0.713928
C -0.968156 0.588147 -0.466225
O -1.192633 1.794626 -0.889783
C 0.298461 0.448966 0.213305
C 0.973894 -0.90192 0.487858
C 1.635123 -1.460472 -0.767609
O 2.675646 -0.565431 -1.156141
O 1.971655 -0.630519 1.47209
O 1.029079 1.517279 0.186765
O -1.7722 -1.659505 -0.157529
O -3.263408 0.086873 1.219558
H 0.251399 -1.629828 0.847597
H -4.201465 -0.54871 -0.527491
H -0.664138 0.522788 1.035182
H 2.052241 -2.44642 -0.539665
H 0.886949 -1.567253 -1.55869
H -0.337894 2.277137 -0.721582
H 3.073981 -0.869842 -1.975899
H 2.108424 -1.404259 2.024824
H -4.076137 0.363607 1.650966
H -3.584795 1.131219 -0.563593
H -2.245504 -1.722359 0.683564
H -2.133594 -0.511991 -1.803571
Na 3.138752 1.067368 0.424145

48. Transition States of 3rd Glu-L3a C=O Migration

25

C	3.290787	0.466565	-0.20013
C	2.009213	-0.134394	0.377598
C	0.831846	0.767635	0.189998
O	0.993963	2.000418	0.559758
C	-0.46513	0.501275	-0.392824
C	-1.050241	-0.919616	-0.569541
C	-1.440012	-1.521873	0.769272
O	-2.427596	-0.676359	1.362862
O	-2.24756	-0.816914	-1.330998
O	-1.275356	1.508386	-0.34682
O	1.728166	-1.378169	-0.205782
O	4.246855	-0.554642	-0.017583
H	-0.331266	-1.58377	-1.043244
H	3.548811	1.383388	0.335555
H	0.423437	0.615749	-1.290379
H	-1.850712	-2.519169	0.593522
H	-0.553588	-1.597318	1.404763
H	0.095737	2.412725	0.44202
H	-2.692047	-1.038916	2.212757
H	-2.045738	-0.819456	-2.271282
H	5.101514	-0.289495	-0.367056
H	3.139091	0.689188	-1.26365
H	2.567454	-1.856127	-0.261608
H	2.139345	-0.230838	1.466746
Na	-3.340781	0.837702	-0.106898

49. Reactant of 3rd Glu-L3a C=O Migration

25

C 3.290787 0.466565 -0.20013
C 2.009213 -0.134394 0.377598
C 0.831846 0.767635 0.189998
O 0.993963 2.000418 0.559758
C -0.46513 0.501275 -0.392824
C -1.050241 -0.919616 -0.569541
C -1.440012 -1.521873 0.769272
O -2.427596 -0.676359 1.362862
O -2.24756 -0.816914 -1.330998
O -1.275356 1.508386 -0.34682
O 1.728166 -1.378169 -0.205782
O 4.246855 -0.554642 -0.017583
H -0.331266 -1.58377 -1.043244
H 3.548811 1.383388 0.335555
H 0.423437 0.615749 -1.290379
H -1.850712 -2.519169 0.593522
H -0.553588 -1.597318 1.404763
H 0.095737 2.412725 0.44202
H -2.692047 -1.038916 2.212757
H -2.045738 -0.819456 -2.271282
H 5.101514 -0.289495 -0.367056
H 3.139091 0.689188 -1.26365
H 2.567454 -1.856127 -0.261608
H 2.139345 -0.230838 1.466746
Na -3.340781 0.837702 -0.106898

50. Transition States of 4th Glu-L3a C=O Migration

25

C 3.327614 -0.067161 0.055315
C 2.111486 0.3997 -0.745955
C 0.985466 -0.581024 -0.585606
O 1.199047 -1.754131 -1.103082
C -0.2469 -0.492581 0.158802
C -0.897497 0.836036 0.601548
C -1.560178 1.554807 -0.56165
O -2.589997 0.709642 -1.080054
O -1.93302 0.535648 1.532125
O -0.987839 -1.555106 0.076185
O 1.642068 1.66661 -0.35625
O 2.921182 -0.163622 1.401377
H -0.157316 1.505139 1.035576
H 4.129688 0.669379 -0.074313
H 0.740963 -0.641616 0.921919
H -1.991443 2.487469 -0.189016
H -0.80978 1.773865 -1.32442
H 0.352912 -2.250679 -0.935764
H -3.010299 1.139982 -1.82926
H -1.56224 0.418224 2.4118
H 3.640128 -0.4992 1.943508
H 3.6632 -1.028405 -0.346095
H 2.386258 2.25804 -0.206748
H 2.374149 0.377451 -1.811695
Na -3.098882 -1.056423 0.282489

51. Reactant of 4th Glu-L3a C=O Migration

25

C 3.327614 -0.067161 0.055315
C 2.111486 0.3997 -0.745955
C 0.985466 -0.581024 -0.585606
O 1.199047 -1.754131 -1.103082
C -0.2469 -0.492581 0.158802
C -0.897497 0.836036 0.601548
C -1.560178 1.554807 -0.56165
O -2.589997 0.709642 -1.080054
O -1.93302 0.535648 1.532125
O -0.987839 -1.555106 0.076185
O 1.642068 1.66661 -0.35625
O 2.921182 -0.163622 1.401377
H -0.157316 1.505139 1.035576
H 4.129688 0.669379 -0.074313
H 0.740963 -0.641616 0.921919
H -1.991443 2.487469 -0.189016
H -0.80978 1.773865 -1.32442
H 0.352912 -2.250679 -0.935764
H -3.010299 1.139982 -1.82926
H -1.56224 0.418224 2.4118
H 3.640128 -0.4992 1.943508
H 3.6632 -1.028405 -0.346095
H 2.386258 2.25804 -0.206748
H 2.374149 0.377451 -1.811695
Na -3.098882 -1.056423 0.282489

52. Transition States of 1st Glu-L3b C-C cleavage

25

C -1.335896 -0.924166 1.425372
C -1.355754 -1.24042 -0.05992
C -0.328333 -0.408188 -0.819058
O -0.704085 0.7448 -1.304078
C 0.994853 -0.785007 -0.838362
C 1.533639 0.895705 0.394871
C 3.017303 0.867167 0.097609
O 3.518248 -0.441131 0.294341
O 0.880972 1.906624 -0.023076
O 1.316642 -1.951774 -0.218773
O -2.665561 -0.924358 -0.529193
O -1.609779 0.476351 1.547184
H 1.232056 0.393307 1.323403
H -0.353683 -1.172306 1.835198
H 1.652557 -0.401223 -1.611883
H 3.188468 1.229653 -0.92034
H 3.474435 1.577439 0.798243
H 2.267536 -1.93855 -0.038075
H 4.475566 -0.423024 0.374243
H 0.024204 1.494205 -0.789277
H -1.534114 0.749276 2.46589
H -2.108032 -1.510759 1.928649
H -2.881132 -1.461725 -1.297447
H -1.128548 -2.300951 -0.189754
Na -2.668697 1.350001 -0.292107

53. Reactant of 1st Glu-L3b C-C cleavage

25

C -1.335896 -0.924166 1.425372
C -1.355754 -1.24042 -0.05992
C -0.328333 -0.408188 -0.819058
O -0.704085 0.7448 -1.304078
C 0.994853 -0.785007 -0.838362
C 1.533639 0.895705 0.394871
C 3.017303 0.867167 0.097609
O 3.518248 -0.441131 0.294341
O 0.880972 1.906624 -0.023076
O 1.316642 -1.951774 -0.218773
O -2.665561 -0.924358 -0.529193
O -1.609779 0.476351 1.547184
H 1.232056 0.393307 1.323403
H -0.353683 -1.172306 1.835198
H 1.652557 -0.401223 -1.611883
H 3.188468 1.229653 -0.92034
H 3.474435 1.577439 0.798243
H 2.267536 -1.93855 -0.038075
H 4.475566 -0.423024 0.374243
H 0.024204 1.494205 -0.789277
H -1.534114 0.749276 2.46589
H -2.108032 -1.510759 1.928649
H -2.881132 -1.461725 -1.297447
H -1.128548 -2.300951 -0.189754
Na -2.668697 1.350001 -0.292107

54. Transition States of 2nd Glu-L3b C-C cleavage

25

C 1.433186 0.422455 1.563075
C 1.281025 1.211783 0.260032
C 0.263085 0.558229 -0.656205
O 0.635585 -0.524699 -1.289149
C -1.068412 0.914169 -0.647593
C -1.55845 -0.839745 0.41949
C -2.980375 -0.970468 -0.079326
O -3.628692 0.279438 0.052711
O -0.73752 -1.784585 0.127221
O -1.447785 1.980356 0.097915
O 2.559876 1.17443 -0.38033
O 1.804315 -0.928643 1.295398
H -1.461036 -0.323026 1.381497
H 0.512075 0.473677 2.147296
H -1.692051 0.617499 -1.485146
H -2.97553 -1.330142 -1.113291
H -3.450413 -1.73538 0.551344
H -2.412517 1.966521 0.168131
H -4.58218 0.168941 0.009363
H -0.00633 -1.339292 -0.773142
H 1.009076 -1.480832 1.283115
H 2.23814 0.873712 2.144209
H 2.699788 1.972954 -0.897638
H 0.979978 2.235135 0.492428
Na 2.857674 -1.040789 -0.754104

55. Reactant of 2nd Glu-L3b C-C cleavage

25

C 1.433186 0.422455 1.563075
C 1.281025 1.211783 0.260032
C 0.263085 0.558229 -0.656205
O 0.635585 -0.524699 -1.289149
C -1.068412 0.914169 -0.647593
C -1.55845 -0.839745 0.41949
C -2.980375 -0.970468 -0.079326
O -3.628692 0.279438 0.052711
O -0.73752 -1.784585 0.127221
O -1.447785 1.980356 0.097915
O 2.559876 1.17443 -0.38033
O 1.804315 -0.928643 1.295398
H -1.461036 -0.323026 1.381497
H 0.512075 0.473677 2.147296
H -1.692051 0.617499 -1.485146
H -2.97553 -1.330142 -1.113291
H -3.450413 -1.73538 0.551344
H -2.412517 1.966521 0.168131
H -4.58218 0.168941 0.009363
H -0.00633 -1.339292 -0.773142
H 1.009076 -1.480832 1.283115
H 2.23814 0.873712 2.144209
H 2.699788 1.972954 -0.897638
H 0.979978 2.235135 0.492428
Na 2.857674 -1.040789 -0.754104

56. Transition States of 3rd Glu-L3b C-C cleavage

25

C 1.554965 1.376046 1.019335
C 1.635843 1.06117 -0.466278
C 0.37631 0.41139 -1.041732
O 0.542573 -0.728404 -1.646317
C -0.91959 0.843342 -0.814236
C -1.320077 -0.763522 0.41491
C -2.812073 -0.843422 0.172818
O -3.367581 0.45079 0.314769
O -0.621244 -1.793155 0.058956
O -1.135175 1.998585 -0.133176
O 2.729674 0.168535 -0.613105
O 1.347809 0.129144 1.698611
H -1.02321 -0.217978 1.314583
H 0.734804 2.064848 1.216536
H -1.669152 0.539942 -1.539726
H -3.002338 -1.268443 -0.818107
H -3.208365 -1.533311 0.927797
H -2.077703 2.035334 0.084052
H -4.32428 0.397858 0.386857
H -0.106872 -1.435994 -1.07942
H 1.358565 0.277561 2.648835
H 2.508033 1.814298 1.32207
H 2.959214 0.083064 -1.545009
H 1.811878 2.004115 -0.997446
Na 1.725338 -1.734115 0.448379

57. Reactant of 3rd Glu-L3b C-C cleavage

25

C 1.554965 1.376046 1.019335
C 1.635843 1.06117 -0.466278
C 0.37631 0.41139 -1.041732
O 0.542573 -0.728404 -1.646317
C -0.91959 0.843342 -0.814236
C -1.320077 -0.763522 0.41491
C -2.812073 -0.843422 0.172818
O -3.367581 0.45079 0.314769
O -0.621244 -1.793155 0.058956
O -1.135175 1.998585 -0.133176
O 2.729674 0.168535 -0.613105
O 1.347809 0.129144 1.698611
H -1.02321 -0.217978 1.314583
H 0.734804 2.064848 1.216536
H -1.669152 0.539942 -1.539726
H -3.002338 -1.268443 -0.818107
H -3.208365 -1.533311 0.927797
H -2.077703 2.035334 0.084052
H -4.32428 0.397858 0.386857
H -0.106872 -1.435994 -1.07942
H 1.358565 0.277561 2.648835
H 2.508033 1.814298 1.32207
H 2.959214 0.083064 -1.545009
H 1.811878 2.004115 -0.997446
Na 1.725338 -1.734115 0.448379

58. Transition States of 4th Glu-L3b C-C cleavage

25

C 2.098152 -1.370168 -0.362395
C 0.831676 -1.59508 0.451942
C -0.434983 -1.158857 -0.26742
O -0.370742 -0.840238 -1.52092
C -1.665791 -1.067866 0.367721
C -1.549092 1.017965 0.245189
C -0.759595 1.621514 1.384575
O 0.618123 1.694701 1.084926
O -1.168502 1.284057 -0.969937
O -2.826295 -1.199452 -0.326625
O 0.867085 -0.968858 1.725907
O 2.371679 0.026398 -0.512436
H -2.626942 0.94637 0.41473
H 1.978744 -1.836609 -1.342669
H -1.762754 -1.275408 1.426311
H -1.129708 2.644725 1.515505
H -0.943724 1.079263 2.313953
H -2.642053 -1.358212 -1.261738
H 1.041433 0.897499 1.438528
H -0.871675 0.210918 -1.510959
H 3.324889 0.149971 -0.496044
H 2.928642 -1.850189 0.160787
H 1.30913 -1.531518 2.369789
H 0.738985 -2.682577 0.575266
Na 1.054732 1.813441 -1.124405

59. Reactant of 4th Glu-L3b C-C cleavage

25

C 2.098152 -1.370168 -0.362395
C 0.831676 -1.59508 0.451942
C -0.434983 -1.158857 -0.26742
O -0.370742 -0.840238 -1.52092
C -1.665791 -1.067866 0.367721
C -1.549092 1.017965 0.245189
C -0.759595 1.621514 1.384575
O 0.618123 1.694701 1.084926
O -1.168502 1.284057 -0.969937
O -2.826295 -1.199452 -0.326625
O 0.867085 -0.968858 1.725907
O 2.371679 0.026398 -0.512436
H -2.626942 0.94637 0.41473
H 1.978744 -1.836609 -1.342669
H -1.762754 -1.275408 1.426311
H -1.129708 2.644725 1.515505
H -0.943724 1.079263 2.313953
H -2.642053 -1.358212 -1.261738
H 1.041433 0.897499 1.438528
H -0.871675 0.210918 -1.510959
H 3.324889 0.149971 -0.496044
H 2.928642 -1.850189 0.160787
H 1.30913 -1.531518 2.369789
H 0.738985 -2.682577 0.575266
Na 1.054732 1.813441 -1.124405

60. Transition States of 1st Glu-L3b C=O Migration

25

C -2.814906 -0.245699 -0.634732
C -2.163137 0.257548 0.655146
C -0.929948 1.019962 0.265667
O -1.127439 2.079679 -0.452924
C 0.459168 0.776988 0.574175
C 1.031791 -0.579389 1.073292
C 0.792894 -1.667008 0.033364
O 1.101909 -1.169915 -1.269946
O 2.444192 -0.440208 1.168736
O 1.2621 1.594669 -0.033429
O -1.766921 -0.771618 1.526639
O -1.825693 -0.92209 -1.398506
H 0.600031 -0.879715 2.026993
H -3.21605 0.610474 -1.184266
H -0.134564 1.298329 1.560002
H 1.464361 -2.500021 0.246116
H -0.240173 -2.004242 0.07614
H -0.20385 2.380636 -0.686579
H 0.261468 -0.964179 -1.699313
H 2.674133 0.13337 1.907119
H -2.245285 -1.34715 -2.152718
H -3.633303 -0.919806 -0.364619
H -2.523928 -1.095581 2.023419
H -2.859816 0.955513 1.134238
Na 2.826965 0.363075 -1.001142

61. Reactant of 1st Glu-L3b C=O Migration

25

C	-2.814906	-0.245699	-0.634732
C	-2.163137	0.257548	0.655146
C	-0.929948	1.019962	0.265667
O	-1.127439	2.079679	-0.452924
C	0.459168	0.776988	0.574175
C	1.031791	-0.579389	1.073292
C	0.792894	-1.667008	0.033364
O	1.101909	-1.169915	-1.269946
O	2.444192	-0.440208	1.168736
O	1.2621	1.594669	-0.033429
O	-1.766921	-0.771618	1.526639
O	-1.825693	-0.92209	-1.398506
H	0.600031	-0.879715	2.026993
H	-3.21605	0.610474	-1.184266
H	-0.134564	1.298329	1.560002
H	1.464361	-2.500021	0.246116
H	-0.240173	-2.004242	0.07614
H	-0.20385	2.380636	-0.686579
H	0.261468	-0.964179	-1.699313
H	2.674133	0.13337	1.907119
H	-2.245285	-1.34715	-2.152718
H	-3.633303	-0.919806	-0.364619
H	-2.523928	-1.095581	2.023419
H	-2.859816	0.955513	1.134238
Na	2.826965	0.363075	-1.001142

62. Transition States of 2nd Glu-L3b C=O Migration

25

C -2.997259 -0.227354 -0.584744
C -2.244877 0.234243 0.661537
C -1.016988 0.977453 0.211729
O -1.239155 2.047544 -0.483349
C 0.384512 0.737869 0.472391
C 0.960285 -0.615549 0.950003
C 0.921837 -1.653071 -0.157114
O 1.710087 -1.161817 -1.247003
O 2.331581 -0.421208 1.2818
O 1.171668 1.574919 -0.125558
O -1.821167 -0.823882 1.48431
O -2.086793 -0.960974 -1.376716
H 0.400562 -1.003628 1.797919
H -3.375133 0.654071 -1.11154
H -0.185831 1.238136 1.48609
H 1.353841 -2.581118 0.226437
H -0.113511 -1.810205 -0.465244
H -0.328808 2.350696 -0.75454
H 1.583301 -1.727487 -2.012559
H 2.410828 -0.06321 2.170902
H -2.517429 -1.237865 -2.189932
H -3.846666 -0.842572 -0.266173
H -2.58552 -1.275642 1.854341
H -2.884042 0.941154 1.205212
Na 3.046689 0.599951 -0.68469

63. Reactant of 2nd Glu-L3b C=O Migration

25

C -2.997259 -0.227354 -0.584744
C -2.244877 0.234243 0.661537
C -1.016988 0.977453 0.211729
O -1.239155 2.047544 -0.483349
C 0.384512 0.737869 0.472391
C 0.960285 -0.615549 0.950003
C 0.921837 -1.653071 -0.157114
O 1.710087 -1.161817 -1.247003
O 2.331581 -0.421208 1.2818
O 1.171668 1.574919 -0.125558
O -1.821167 -0.823882 1.48431
O -2.086793 -0.960974 -1.376716
H 0.400562 -1.003628 1.797919
H -3.375133 0.654071 -1.11154
H -0.185831 1.238136 1.48609
H 1.353841 -2.581118 0.226437
H -0.113511 -1.810205 -0.465244
H -0.328808 2.350696 -0.75454
H 1.583301 -1.727487 -2.012559
H 2.410828 -0.06321 2.170902
H -2.517429 -1.237865 -2.189932
H -3.846666 -0.842572 -0.266173
H -2.58552 -1.275642 1.854341
H -2.884042 0.941154 1.205212
Na 3.046689 0.599951 -0.68469

64. Transition States of 3rd Glu-L3b C=O Migration

25

C -3.02704 -0.225104 -0.584304
C -2.271863 0.222069 0.666111
C -1.041713 0.962128 0.215883
O -1.267273 2.048664 -0.452597
C 0.362994 0.708673 0.437857
C 0.926638 -0.647632 0.881321
C 0.975103 -1.63662 -0.275946
O 1.887217 -1.127303 -1.25256
O 2.252367 -0.36679 1.336086
O 1.141346 1.549811 -0.165098
O -1.861115 -0.847073 1.480755
O -2.119263 -0.955325 -1.382967
H 0.318987 -1.0695 1.678336
H -3.401663 0.661991 -1.103698
H -0.176807 1.191856 1.475204
H 1.334837 -2.600679 0.098487
H -0.028793 -1.752759 -0.691647
H -0.358642 2.346153 -0.73844
H 1.807864 -1.63812 -2.061799
H 2.508243 -1.003631 2.008284
H -2.552127 -1.226999 -2.196762
H -3.87827 -0.840334 -0.270864
H -2.631821 -1.284901 1.85419
H -2.904547 0.93078 1.214765
Na 3.104708 0.688108 -0.529139

65. Reactant of 3rd Glu-L3b C=O Migration

25

C -3.02704 -0.225104 -0.584304
C -2.271863 0.222069 0.666111
C -1.041713 0.962128 0.215883
O -1.267273 2.048664 -0.452597
C 0.362994 0.708673 0.437857
C 0.926638 -0.647632 0.881321
C 0.975103 -1.63662 -0.275946
O 1.887217 -1.127303 -1.25256
O 2.252367 -0.36679 1.336086
O 1.141346 1.549811 -0.165098
O -1.861115 -0.847073 1.480755
O -2.119263 -0.955325 -1.382967
H 0.318987 -1.0695 1.678336
H -3.401663 0.661991 -1.103698
H -0.176807 1.191856 1.475204
H 1.334837 -2.600679 0.098487
H -0.028793 -1.752759 -0.691647
H -0.358642 2.346153 -0.73844
H 1.807864 -1.63812 -2.061799
H 2.508243 -1.003631 2.008284
H -2.552127 -1.226999 -2.196762
H -3.87827 -0.840334 -0.270864
H -2.631821 -1.284901 1.85419
H -2.904547 0.93078 1.214765
Na 3.104708 0.688108 -0.529139

66. Transition States of 4th Glu-L3b C=O Migration

25

C 2.927173 0.120562 0.772686
C 2.103164 0.225879 -0.515302
C 0.836483 0.977906 -0.24356
O 0.985938 2.173905 0.236274
C -0.545781 0.621642 -0.48616
C -1.040167 -0.821843 -0.726438
C -1.077721 -1.619777 0.566294
O -1.995227 -0.982781 1.457351
O -2.381088 -0.757174 -1.200151
O -1.392464 1.512653 -0.082358
O 1.775977 -1.046025 -1.007602
O 3.995171 -0.723645 0.407316
H -0.393729 -1.345083 -1.427395
H 2.318893 -0.324562 1.570228
H 0.041255 0.948715 -1.558852
H -1.416925 -2.632619 0.335143
H -0.073446 -1.657786 0.996185
H 0.056976 2.48538 0.413939
H -2.059309 -1.492403 2.269491
H -2.392623 -0.651772 -2.155944
H 4.612811 -0.830408 1.135175
H 3.262116 1.114819 1.078356
H 2.593492 -1.562182 -1.017769
H 2.694768 0.793486 -1.245283
Na -3.306331 0.610249 0.446815

67. Reactant of 4th Glu-L3b C=O Migration

25

C   2.927173   0.120562   0.772686
C   2.103164   0.225879   -0.515302
C   0.836483   0.977906   -0.24356
O   0.985938   2.173905   0.236274
C   -0.545781   0.621642   -0.48616
C   -1.040167   -0.821843   -0.726438
C   -1.077721   -1.619777   0.566294
O   -1.995227   -0.982781   1.457351
O   -2.381088   -0.757174   -1.200151
O   -1.392464   1.512653   -0.082358
O   1.775977   -1.046025   -1.007602
O   3.995171   -0.723645   0.407316
H   -0.393729   -1.345083   -1.427395
H   2.318893   -0.324562   1.570228
H   0.041255   0.948715   -1.558852
H   -1.416925   -2.632619   0.335143
H   -0.073446   -1.657786   0.996185
H   0.056976   2.48538   0.413939
H   -2.059309   -1.492403   2.269491
H   -2.392623   -0.651772   -2.155944
H   4.612811   -0.830408   1.135175
H   3.262116   1.114819   1.078356
H   2.593492   -1.562182   -1.017769
H   2.694768   0.793486   -1.245283
Na   -3.306331   0.610249   0.446815

68. Transition States of 1st Man-L1 C-C cleavage

25

C	-2.880587	-0.395299	-0.630283
C	-2.208742	-0.383011	0.577876
C	-0.409668	-0.143794	-0.324497
O	-0.474762	0.991585	-0.910793
C	0.444444	-0.244916	0.936044
C	1.939628	-0.401549	0.583123
C	2.295178	-1.706362	-0.12296
O	1.912729	-1.540463	-1.478921
O	2.370878	0.706061	-0.198477
O	0.280512	0.985335	1.647053
O	-2.15888	-1.557953	1.301143
O	-2.866207	0.601326	-1.456266
H	2.488135	-0.361778	1.529434
H	-3.354203	-1.311736	-0.982815
H	-2.059617	0.547925	1.117351
H	-0.38495	-1.055834	-0.929892
H	0.120158	-1.096628	1.539111
H	-2.797405	-1.542483	2.021956
H	3.373246	-1.868949	-0.041509
H	1.771306	-2.545663	0.345993
H	0.48835	0.856635	2.577086
H	-1.9829	1.091586	-1.329566
H	2.287634	-2.237644	-2.023921
H	2.399198	0.395541	-1.116946
Na	0.914124	2.481044	-0.004729

69. Reactant of 1st Man-L1 C-C cleavage

25

C -2.880587 -0.395299 -0.630283
C -2.208742 -0.383011 0.577876
C -0.409668 -0.143794 -0.324497
O -0.474762 0.991585 -0.910793
C 0.444444 -0.244916 0.936044
C 1.939628 -0.401549 0.583123
C 2.295178 -1.706362 -0.12296
O 1.912729 -1.540463 -1.478921
O 2.370878 0.706061 -0.198477
O 0.280512 0.985335 1.647053
O -2.15888 -1.557953 1.301143
O -2.866207 0.601326 -1.456266
H 2.488135 -0.361778 1.529434
H -3.354203 -1.311736 -0.982815
H -2.059617 0.547925 1.117351
H -0.38495 -1.055834 -0.929892
H 0.120158 -1.096628 1.539111
H -2.797405 -1.542483 2.021956
H 3.373246 -1.868949 -0.041509
H 1.771306 -2.545663 0.345993
H 0.48835 0.856635 2.577086
H -1.9829 1.091586 -1.329566
H 2.287634 -2.237644 -2.023921
H 2.399198 0.395541 -1.116946
Na 0.914124 2.481044 -0.004729

70. Transition States of 2nd Man-L1 C-C cleavage

25

C -1.694138 -1.655743 0.08343
C -1.8911 -0.401215 -0.445902
C -0.757525 0.393142 1.18764
O 0.034866 -0.470952 1.712623
C -0.120105 1.594274 0.48835
C 1.164467 1.375804 -0.337754
C 2.417985 0.864259 0.375854
O 2.436905 -0.545827 0.546567
O 0.938547 0.507198 -1.456322
O -1.100652 2.205759 -0.339831
O -3.12421 0.141854 -0.409894
O -0.483991 -2.147734 0.168068
H 1.419899 2.372781 -0.714926
H -2.503307 -2.16238 0.612187
H -1.181073 -0.013891 -1.163181
H -1.701786 0.62351 1.689352
H 0.153025 2.267507 1.314079
H -3.06915 1.056799 -0.70748
H 3.276958 1.105117 -0.250848
H 2.543902 1.374974 1.334219
H -1.104708 3.15926 -0.21669
H -0.087872 -1.500746 1.057777
H 1.963112 -0.730644 1.367418
H 0.577044 1.020034 -2.186238
Na 1.398614 -1.737985 -1.09228

71. Reactant of 2nd Man-L1 C-C cleavage

25

C -1.694138 -1.655743 0.08343
C -1.8911 -0.401215 -0.445902
C -0.757525 0.393142 1.18764
O 0.034866 -0.470952 1.712623
C -0.120105 1.594274 0.48835
C 1.164467 1.375804 -0.337754
C 2.417985 0.864259 0.375854
O 2.436905 -0.545827 0.546567
O 0.938547 0.507198 -1.456322
O -1.100652 2.205759 -0.339831
O -3.12421 0.141854 -0.409894
O -0.483991 -2.147734 0.168068
H 1.419899 2.372781 -0.714926
H -2.503307 -2.16238 0.612187
H -1.181073 -0.013891 -1.163181
H -1.701786 0.62351 1.689352
H 0.153025 2.267507 1.314079
H -3.06915 1.056799 -0.70748
H 3.276958 1.105117 -0.250848
H 2.543902 1.374974 1.334219
H -1.104708 3.15926 -0.21669
H -0.087872 -1.500746 1.057777
H 1.963112 -0.730644 1.367418
H 0.577044 1.020034 -2.186238
Na 1.398614 -1.737985 -1.09228

72. Transition States of 3rd Man-L1 C-C cleavage

25

C -2.366576 -0.381127 0.94738
C -2.39146 -1.166162 -0.200094
C -0.596854 -0.275965 -0.566395
O -0.836631 1.004152 -0.633829
C 0.5004 -0.790656 0.364945
C 1.850071 -1.110855 -0.315918
C 2.750423 0.107985 -0.526677
O 2.051905 1.241631 -1.031416
O 2.599933 -1.931716 0.560965
O 0.668131 0.160658 1.40994
O -3.08431 -0.810769 -1.296543
O -2.649543 0.87066 0.84738
H 1.650029 -1.618582 -1.266793
H -1.964409 -0.777579 1.880107
H -2.153199 -2.222579 -0.157982
H -0.658407 -0.823127 -1.512203
H 0.165876 -1.745766 0.786578
H -3.514536 0.044023 -1.154165
H 3.163613 0.389009 0.442959
H 3.58828 -0.161688 -1.170926
H 1.197219 -0.242144 2.108775
H -1.866517 1.190394 0.071978
H 2.050941 1.245656 -1.992411
H 2.526903 -2.859663 0.323439
Na 0.667482 2.273254 0.415384

73. Reactant of 3rd Man-L1 C-C cleavage

25

C	-2.366576	-0.381127	0.94738
C	-2.39146	-1.166162	-0.200094
C	-0.596854	-0.275965	-0.566395
O	-0.836631	1.004152	-0.633829
C	0.5004	-0.790656	0.364945
C	1.850071	-1.110855	-0.315918
C	2.750423	0.107985	-0.526677
O	2.051905	1.241631	-1.031416
O	2.599933	-1.931716	0.560965
O	0.668131	0.160658	1.40994
O	-3.08431	-0.810769	-1.296543
O	-2.649543	0.87066	0.84738
H	1.650029	-1.618582	-1.266793
H	-1.964409	-0.777579	1.880107
H	-2.153199	-2.222579	-0.157982
H	-0.658407	-0.823127	-1.512203
H	0.165876	-1.745766	0.786578
H	-3.514536	0.044023	-1.154165
H	3.163613	0.389009	0.442959
H	3.58828	-0.161688	-1.170926
H	1.197219	-0.242144	2.108775
H	-1.866517	1.190394	0.071978
H	2.050941	1.245656	-1.992411
H	2.526903	-2.859663	0.323439
Na	0.667482	2.273254	0.415384

74. Transition States of 4th Man-L1 C-C cleavage

25

C -2.962635 0.845411 0.064701
C -1.855876 0.912826 -0.765471
C -0.696848 0.045667 0.639889
O -1.198472 -1.13085 0.828772
C 0.621408 0.145169 -0.107486
C 1.777864 0.221358 0.910422
C 3.098 -0.161127 0.240599
O 3.164207 0.309657 -1.094343
O 1.756013 1.572896 1.326766
O 0.737857 -0.984759 -0.967387
O -1.323141 2.094063 -1.188843
O -3.45125 -0.274466 0.490191
H 1.596459 -0.453988 1.758088
H -3.394703 1.755154 0.490444
H -1.649494 0.101482 -1.454375
H -0.761451 0.776992 1.45373
H 0.663563 1.070184 -0.686065
H -1.684006 2.847168 -0.708226
H 3.934481 0.209919 0.840535
H 3.187562 -1.247469 0.184615
H 1.465748 -0.781589 -1.575297
H -2.636363 -0.904239 0.648995
H 3.372079 1.24901 -1.104182
H 2.261072 1.681595 2.137788
Na -0.338921 -2.822944 -0.276656

75. Reactant of 4th Man-L1 C-C cleavage

25

C -2.962635 0.845411 0.064701
C -1.855876 0.912826 -0.765471
C -0.696848 0.045667 0.639889
O -1.198472 -1.13085 0.828772
C 0.621408 0.145169 -0.107486
C 1.777864 0.221358 0.910422
C 3.098 -0.161127 0.240599
O 3.164207 0.309657 -1.094343
O 1.756013 1.572896 1.326766
O 0.737857 -0.984759 -0.967387
O -1.323141 2.094063 -1.188843
O -3.45125 -0.274466 0.490191
H 1.596459 -0.453988 1.758088
H -3.394703 1.755154 0.490444
H -1.649494 0.101482 -1.454375
H -0.761451 0.776992 1.45373
H 0.663563 1.070184 -0.686065
H -1.684006 2.847168 -0.708226
H 3.934481 0.209919 0.840535
H 3.187562 -1.247469 0.184615
H 1.465748 -0.781589 -1.575297
H -2.636363 -0.904239 0.648995
H 3.372079 1.24901 -1.104182
H 2.261072 1.681595 2.137788
Na -0.338921 -2.822944 -0.276656

76. Transition States of 1st Man-L1 C=O Migration

25

C 2.366085 -1.267747 -0.020077
C 1.696739 -0.020467 0.207257
C 0.618687 0.053088 1.284319
O 0.45586 1.433679 1.572781
C -0.673274 -0.636531 0.800128
C -1.194975 -0.179262 -0.573637
C -2.477858 -0.901536 -0.948328
O -3.438853 -0.475524 0.008298
O -1.445236 1.226551 -0.580793
O -0.326405 -2.009273 0.724631
O 1.681448 0.810944 -0.78523
O 2.997729 -1.389849 -1.133941
H -0.43155 -0.377756 -1.328218
H 2.445686 -2.07168 0.70347
H 2.771667 -0.028061 0.842628
H 0.94566 -0.49137 2.175961
H -1.452273 -0.461199 1.551496
H 2.756917 -0.593413 -1.667266
H -2.769605 -0.603978 -1.959715
H -2.330023 -1.984547 -0.925847
H -1.041018 -2.559994 1.054694
H -0.002676 1.539056 2.41143
H -4.318838 -0.767105 -0.243481
H -2.346606 1.349978 -0.251653
Na 0.396717 2.544779 -0.482495

77. Reactant of 1st Man-L1 C=O Migration

25

C	2.366085	-1.267747	-0.020077
C	1.696739	-0.020467	0.207257
C	0.618687	0.053088	1.284319
O	0.45586	1.433679	1.572781
C	-0.673274	-0.636531	0.800128
C	-1.194975	-0.179262	-0.573637
C	-2.477858	-0.901536	-0.948328
O	-3.438853	-0.475524	0.008298
O	-1.445236	1.226551	-0.580793
O	-0.326405	-2.009273	0.724631
O	1.681448	0.810944	-0.78523
O	2.997729	-1.389849	-1.133941
H	-0.43155	-0.377756	-1.328218
H	2.445686	-2.07168	0.70347
H	2.771667	-0.028061	0.842628
H	0.94566	-0.49137	2.175961
H	-1.452273	-0.461199	1.551496
H	2.756917	-0.593413	-1.667266
H	-2.769605	-0.603978	-1.959715
H	-2.330023	-1.984547	-0.925847
H	-1.041018	-2.559994	1.054694
H	-0.002676	1.539056	2.41143
H	-4.318838	-0.767105	-0.243481
H	-2.346606	1.349978	-0.251653
Na	0.396717	2.544779	-0.482495

78. Transition States of 2nd Man-L1 C=O Migration

25

C   -2.494877   -0.897792   -0.004574
C   -1.32286   -0.067147   0.106343
C   -0.30621   -0.458274   1.197952
O   -0.228041   -1.851285   1.334705
C   1.086762   0.201661   1.113837
C   1.934036   0.071917   -0.160285
C   2.295952   -1.343491   -0.575343
O   1.108057   -1.954335   -1.08972
O   1.339549   0.770263   -1.24838
O   0.97646   1.612983   1.327189
O   -1.475448   1.143214   -0.332195
O   -3.486956   -0.391692   -0.644281
H   2.868031   0.594308   0.064078
H   -2.566438   -1.925993   0.332275
H   -1.130047   -0.918868   -0.784934
H   -0.738478   -0.089937   2.134563
H   1.66851   -0.251965   1.924609
H   -3.239358   0.557762   -0.789054
H   3.058279   -1.294332   -1.355443
H   2.685871   -1.90674   0.277431
H   0.852876   1.79854   2.262681
H   0.166292   -2.218945   0.530008
H   1.332566   -2.703249   -1.651073
H   0.828536   0.137014   -1.768613
Na   0.107961   2.59527   -0.547513

79. Reactant of 2nd Man-L1 C=O Migration

25

C -2.494877 -0.897792 -0.004574
C -1.32286 -0.067147 0.106343
C -0.30621 -0.458274 1.197952
O -0.228041 -1.851285 1.334705
C 1.086762 0.201661 1.113837
C 1.934036 0.071917 -0.160285
C 2.295952 -1.343491 -0.575343
O 1.108057 -1.954335 -1.08972
O 1.339549 0.770263 -1.24838
O 0.97646 1.612983 1.327189
O -1.475448 1.143214 -0.332195
O -3.486956 -0.391692 -0.644281
H 2.868031 0.594308 0.064078
H -2.566438 -1.925993 0.332275
H -1.130047 -0.918868 -0.784934
H -0.738478 -0.089937 2.134563
H 1.66851 -0.251965 1.924609
H -3.239358 0.557762 -0.789054
H 3.058279 -1.294332 -1.355443
H 2.685871 -1.90674 0.277431
H 0.852876 1.79854 2.262681
H 0.166292 -2.218945 0.530008
H 1.332566 -2.703249 -1.651073
H 0.828536 0.137014 -1.768613
Na 0.107961 2.59527 -0.547513

80. Transition States of 3rd Man-L1 C=O Migration

25

C 2.534569 -1.372235 0.579424
C 1.774848 -0.155744 0.435312
C 0.277659 -0.134628 0.733561
O -0.000248 1.192817 1.137252
C -0.528956 -0.527277 -0.519092
C -1.962259 -1.00469 -0.251057
C -2.870534 0.002584 0.448629
O -2.396041 0.204756 1.778336
O -2.459169 -1.251262 -1.554764
O -0.559503 0.586027 -1.401775
O 2.283029 0.69726 -0.400225
O 3.662089 -1.389488 -0.024482
H -1.915163 -1.924168 0.344901
H 2.298118 -2.203256 1.237661
H 2.28755 -0.115794 1.553102
H 0.046029 -0.843541 1.537394
H -0.018496 -1.366268 -1.009121
H 3.659564 -0.560644 -0.579662
H -2.877186 0.944228 -0.108578
H -3.887054 -0.39946 0.462136
H -1.103728 0.331985 -2.158891
H -0.859019 1.17604 1.59219
H -3.124708 0.406447 2.371066
H -3.281314 -1.748683 -1.529312
Na 0.834745 2.284925 -0.777116

81. Reactant of 3rd Man-L1 C=O Migration

25

C 2.534569 -1.372235 0.579424
C 1.774848 -0.155744 0.435312
C 0.277659 -0.134628 0.733561
O -0.000248 1.192817 1.137252
C -0.528956 -0.527277 -0.519092
C -1.962259 -1.00469 -0.251057
C -2.870534 0.002584 0.448629
O -2.396041 0.204756 1.778336
O -2.459169 -1.251262 -1.554764
O -0.559503 0.586027 -1.401775
O 2.283029 0.69726 -0.400225
O 3.662089 -1.389488 -0.024482
H -1.915163 -1.924168 0.344901
H 2.298118 -2.203256 1.237661
H 2.28755 -0.115794 1.553102
H 0.046029 -0.843541 1.537394
H -0.018496 -1.366268 -1.009121
H 3.659564 -0.560644 -0.579662
H -2.877186 0.944228 -0.108578
H -3.887054 -0.39946 0.462136
H -1.103728 0.331985 -2.158891
H -0.859019 1.17604 1.59219
H -3.124708 0.406447 2.371066
H -3.281314 -1.748683 -1.529312
Na 0.834745 2.284925 -0.777116

82. Transition States of 4th Man-L1 C=O Migration

25

C 3.056937 -0.063655 -0.000293
C 1.624908 -0.134958 -0.121205
C 0.794285 1.041168 0.40074
O 1.527537 2.196109 0.06497
C -0.611652 1.045589 -0.259371
C -1.723728 0.520607 0.661803
C -2.94547 0.105065 -0.140448
O -2.580493 -1.071406 -0.874994
O -1.251209 -0.594164 1.421857
O -1.028263 2.362138 -0.552456
O 1.135433 -1.334866 -0.224474
O 3.69547 -1.165885 -0.153354
H -1.990968 1.348284 1.325712
H 3.625714 0.854757 0.094941
H 2.055931 0.320576 -1.182029
H 0.701848 0.935189 1.48696
H -0.574532 0.444151 -1.176051
H 2.996636 -1.86708 -0.180875
H -3.782546 -0.119622 0.526335
H -3.223804 0.917204 -0.816159
H -0.550553 2.688281 -1.321272
H 1.109806 2.967986 0.460443
H -3.238263 -1.243821 -1.553964
H -1.650668 -0.58667 2.295803
Na -0.78528 -2.261594 -0.059963

83. Reactant of 4th Man-L1 C=O Migration

25

C 3.056937 -0.063655 -0.000293
C 1.624908 -0.134958 -0.121205
C 0.794285 1.041168 0.40074
O 1.527537 2.196109 0.06497
C -0.611652 1.045589 -0.259371
C -1.723728 0.520607 0.661803
C -2.94547 0.105065 -0.140448
O -2.580493 -1.071406 -0.874994
O -1.251209 -0.594164 1.421857
O -1.028263 2.362138 -0.552456
O 1.135433 -1.334866 -0.224474
O 3.69547 -1.165885 -0.153354
H -1.990968 1.348284 1.325712
H 3.625714 0.854757 0.094941
H 2.055931 0.320576 -1.182029
H 0.701848 0.935189 1.48696
H -0.574532 0.444151 -1.176051
H 2.996636 -1.86708 -0.180875
H -3.782546 -0.119622 0.526335
H -3.223804 0.917204 -0.816159
H -0.550553 2.688281 -1.321272
H 1.109806 2.967986 0.460443
H -3.238263 -1.243821 -1.553964
H -1.650668 -0.58667 2.295803
Na -0.78528 -2.261594 -0.059963

84. Transition States of 1st Gal-L1 C-C cleavage

25

C 2.690262 -0.711094 0.498385
C 2.446967 0.03744 -0.641928
C 0.428419 -0.077258 -0.502985
O 0.16754 -1.334156 -0.464351
C 0.019824 0.804091 0.680535
C -1.509703 0.833801 0.9065
C -2.316049 1.280468 -0.29686
O -2.323703 0.217814 -1.262309
O -2.015877 -0.434608 1.320208
O 0.361602 2.14693 0.478852
O 2.814212 1.370625 -0.60868
O 2.253505 -1.916188 0.633464
H -1.66785 1.582811 1.688512
H 3.139091 -0.243265 1.376191
H 2.409241 -0.452804 -1.608829
H 0.362462 0.446379 -1.46521
H 0.462677 0.390195 1.598267
H 3.500853 1.568368 -1.254073
H -3.339387 1.49758 0.017432
H -1.855577 2.179374 -0.712386
H 1.294525 2.227218 0.240395
H 1.383878 -1.98433 0.073666
H -2.726952 0.534404 -2.075467
H -1.855827 -0.56278 2.259439
Na -1.97488 -1.871925 -0.434299

85. Reactant of 1st Gal-L1 C-C cleavage

25

C 2.690262 -0.711094 0.498385
C 2.446967 0.03744 -0.641928
C 0.428419 -0.077258 -0.502985
O 0.16754 -1.334156 -0.464351
C 0.019824 0.804091 0.680535
C -1.509703 0.833801 0.9065
C -2.316049 1.280468 -0.29686
O -2.323703 0.217814 -1.262309
O -2.015877 -0.434608 1.320208
O 0.361602 2.14693 0.478852
O 2.814212 1.370625 -0.60868
O 2.253505 -1.916188 0.633464
H -1.66785 1.582811 1.688512
H 3.139091 -0.243265 1.376191
H 2.409241 -0.452804 -1.608829
H 0.362462 0.446379 -1.46521
H 0.462677 0.390195 1.598267
H 3.500853 1.568368 -1.254073
H -3.339387 1.49758 0.017432
H -1.855577 2.179374 -0.712386
H 1.294525 2.227218 0.240395
H 1.383878 -1.98433 0.073666
H -2.726952 0.534404 -2.075467
H -1.855827 -0.56278 2.259439
Na -1.97488 -1.871925 -0.434299

86. Transition States of 2nd Gal-L1 C-C cleavage

25

C	-2.492263	1.412612	0.0728
C	-1.276999	1.179737	-0.557355
C	-0.699507	0.174602	1.141297
O	-1.535187	-0.807032	1.180534
C	0.754102	-0.199382	0.817052
C	1.640083	0.922379	0.23687
C	3.106525	0.671554	0.547752
O	3.415583	-0.577565	-0.069844
O	1.456418	1.025059	-1.168757
O	0.776841	-1.352594	-0.008993
O	-1.150769	0.133593	-1.446073
O	-3.333455	0.468977	0.298217
H	1.347113	1.877335	0.685149
H	-2.661737	2.386195	0.537048
H	-0.619805	2.02355	-0.731429
H	-0.757266	0.93717	1.926427
H	1.153625	-0.462136	1.808209
H	-0.237828	0.203283	-1.771956
H	3.714865	1.475629	0.124371
H	3.264163	0.62934	1.629043
H	1.706739	-1.604178	-0.113553
H	-2.69394	-0.305757	0.752068
H	4.353554	-0.775831	0.004957
H	2.170125	0.540012	-1.601262
Na	-1.2697	-2.092465	-0.643658

87. Reactant of 2nd Gal-L1 C-C cleavage

25

C -2.492263 1.412612 0.0728
C -1.276999 1.179737 -0.557355
C -0.699507 0.174602 1.141297
O -1.535187 -0.807032 1.180534
C 0.754102 -0.199382 0.817052
C 1.640083 0.922379 0.23687
C 3.106525 0.671554 0.547752
O 3.415583 -0.577565 -0.069844
O 1.456418 1.025059 -1.168757
O 0.776841 -1.352594 -0.008993
O -1.150769 0.133593 -1.446073
O -3.333455 0.468977 0.298217
H 1.347113 1.877335 0.685149
H -2.661737 2.386195 0.537048
H -0.619805 2.02355 -0.731429
H -0.757266 0.93717 1.926427
H 1.153625 -0.462136 1.808209
H -0.237828 0.203283 -1.771956
H 3.714865 1.475629 0.124371
H 3.264163 0.62934 1.629043
H 1.706739 -1.604178 -0.113553
H -2.69394 -0.305757 0.752068
H 4.353554 -0.775831 0.004957
H 2.170125 0.540012 -1.601262
Na -1.2697 -2.092465 -0.643658

88. Transition States of 3rd Gal-L1 C-C cleavage

25

C -2.513634 1.376638 0.147099
C -1.306505 1.199015 -0.513771
C -0.674944 0.146159 1.145996
O -1.495638 -0.847951 1.175441
C 0.772819 -0.209774 0.77371
C 1.664143 0.929716 0.247898
C 3.13112 0.622341 0.513592
O 3.397076 -0.667095 -0.015755
O 1.407027 1.07392 -1.141911
O 0.775888 -1.31996 -0.10271
O -1.171851 0.190938 -1.441928
O -3.330056 0.404405 0.344762
H 1.407173 1.853941 0.78381
H -2.693123 2.324689 0.658238
H -0.664789 2.059624 -0.654547
H -0.723021 0.884539 1.954199
H 1.190243 -0.516413 1.74543
H -0.259838 0.264441 -1.770257
H 3.76179 1.382012 0.036794
H 3.312859 0.653365 1.592946
H 1.707056 -1.522076 -0.287456
H -2.664725 -0.362557 0.783408
H 4.315681 -0.913219 0.123463
H 2.143135 1.51104 -1.580444
Na -1.266364 -2.061636 -0.710538

89. Reactant of 3rd Gal-L1 C-C cleavage

25

C -2.513634 1.376638 0.147099
C -1.306505 1.199015 -0.513771
C -0.674944 0.146159 1.145996
O -1.495638 -0.847951 1.175441
C 0.772819 -0.209774 0.77371
C 1.664143 0.929716 0.247898
C 3.13112 0.622341 0.513592
O 3.397076 -0.667095 -0.015755
O 1.407027 1.07392 -1.141911
O 0.775888 -1.31996 -0.10271
O -1.171851 0.190938 -1.441928
O -3.330056 0.404405 0.344762
H 1.407173 1.853941 0.78381
H -2.693123 2.324689 0.658238
H -0.664789 2.059624 -0.654547
H -0.723021 0.884539 1.954199
H 1.190243 -0.516413 1.74543
H -0.259838 0.264441 -1.770257
H 3.76179 1.382012 0.036794
H 3.312859 0.653365 1.592946
H 1.707056 -1.522076 -0.287456
H -2.664725 -0.362557 0.783408
H 4.315681 -0.913219 0.123463
H 2.143135 1.51104 -1.580444
Na -1.266364 -2.061636 -0.710538

90. Transition States of 4th Gal-L1 C-C cleavage

25

C -2.328036 -0.415337 0.888601
C -2.266109 -0.925939 -0.392683
C -0.841139 0.398052 -1.119389
O -1.060061 1.544475 -0.615675
C 0.518483 -0.285328 -0.870224
C 0.837024 -0.717722 0.564533
C 2.125761 -1.525636 0.644014
O 3.172455 -0.808005 0.008251
O 0.892254 0.471785 1.357148
O 1.438973 0.71795 -1.302778
O -1.75284 -2.193709 -0.555795
O -2.598248 0.828365 1.123154
H 0.038196 -1.376094 0.915626
H -2.033562 -1.022466 1.745163
H -2.954405 -0.549469 -1.141367
H -1.220523 0.20355 -2.128275
H 0.577484 -1.169562 -1.511109
H -2.388676 -2.770207 -0.991851
H 2.368536 -1.715422 1.696301
H 1.9545 -2.489738 0.152827
H 2.328169 0.334725 -1.242298
H -2.208909 1.382056 0.355597
H 3.994703 -1.303543 0.055855
H 1.092768 0.247076 2.270788
Na 0.857778 2.415787 0.129017

91. Reactant of 4th Gal-L1 C-C cleavage

25

C	-2.328036	-0.415337	0.888601
C	-2.266109	-0.925939	-0.392683
C	-0.841139	0.398052	-1.119389
O	-1.060061	1.544475	-0.615675
C	0.518483	-0.285328	-0.870224
C	0.837024	-0.717722	0.564533
C	2.125761	-1.525636	0.644014
O	3.172455	-0.808005	0.008251
O	0.892254	0.471785	1.357148
O	1.438973	0.71795	-1.302778
O	-1.75284	-2.193709	-0.555795
O	-2.598248	0.828365	1.123154
H	0.038196	-1.376094	0.915626
H	-2.033562	-1.022466	1.745163
H	-2.954405	-0.549469	-1.141367
H	-1.220523	0.20355	-2.128275
H	0.577484	-1.169562	-1.511109
H	-2.388676	-2.770207	-0.991851
H	2.368536	-1.715422	1.696301
H	1.9545	-2.489738	0.152827
H	2.328169	0.334725	-1.242298
H	-2.208909	1.382056	0.355597
H	3.994703	-1.303543	0.055855
H	1.092768	0.247076	2.270788
Na	0.857778	2.415787	0.129017

92. Transition States of 1st Gal-L1 C=O Migration

25

C 2.951162 1.072022 0.17014
C 1.680695 0.395709 0.189662
C 0.621353 0.733413 -0.858432
O 0.606278 -0.378917 -1.738968
C -0.78323 0.993769 -0.283959
C -1.266809 -0.014433 0.773844
C -2.585531 0.404492 1.406799
O -3.554443 0.384567 0.360987
O -1.408274 -1.320766 0.216755
O -1.599959 1.004849 -1.434133
O 1.679731 -0.757378 0.775334
O 3.892214 0.543547 0.862139
H -0.514067 -0.091634 1.561523
H 3.146003 2.033094 -0.296612
H 1.632384 1.383525 0.96288
H 0.914518 1.636055 -1.408585
H -0.760003 1.984866 0.193012
H 3.5408 -0.334228 1.158075
H -2.849351 -0.310524 2.190386
H -2.496133 1.403849 1.843657
H -2.528535 1.073905 -1.173535
H -0.134863 -0.227157 -2.343355
H -4.442187 0.492291 0.7134
H -2.256878 -1.344778 -0.245146
Na 0.55619 -2.27421 -0.352812

93. Reactant of 1st Gal-L1 C=O Migration

25

C	2.951162	1.072022	0.17014
C	1.680695	0.395709	0.189662
C	0.621353	0.733413	-0.858432
O	0.606278	-0.378917	-1.738968
C	-0.78323	0.993769	-0.283959
C	-1.266809	-0.014433	0.773844
C	-2.585531	0.404492	1.406799
O	-3.554443	0.384567	0.360987
O	-1.408274	-1.320766	0.216755
O	-1.599959	1.004849	-1.434133
O	1.679731	-0.757378	0.775334
O	3.892214	0.543547	0.862139
H	-0.514067	-0.091634	1.561523
H	3.146003	2.033094	-0.296612
H	1.632384	1.383525	0.96288
H	0.914518	1.636055	-1.408585
H	-0.760003	1.984866	0.193012
H	3.5408	-0.334228	1.158075
H	-2.849351	-0.310524	2.190386
H	-2.496133	1.403849	1.843657
H	-2.528535	1.073905	-1.173535
H	-0.134863	-0.227157	-2.343355
H	-4.442187	0.492291	0.7134
H	-2.256878	-1.344778	-0.245146
Na	0.55619	-2.27421	-0.352812

94. Transition States of 2nd Gal-L1 C=O Migration

25

C	2.765074	-0.2094	-0.369361
C	1.639299	-0.005644	0.491258
C	0.880204	1.324566	0.40702
O	1.711279	2.22453	-0.287509
C	-0.46407	1.107324	-0.320537
C	-1.642337	0.811657	0.629861
C	-2.948656	0.559336	-0.110323
O	-2.823314	-0.663051	-0.848948
O	-1.457235	-0.318118	1.461648
O	-0.253479	0.089017	-1.297042
O	1.059078	-1.073241	0.952872
O	3.214418	-1.413764	-0.465246
H	-1.784746	1.714584	1.239363
H	3.313818	0.583148	-0.86385
H	2.679564	0.321828	1.092085
H	0.681164	1.677119	1.426029
H	-0.691695	2.054806	-0.821529
H	2.579436	-1.981978	0.028852
H	-3.748177	0.470584	0.628133
H	-3.171481	1.393032	-0.78382
H	-0.873757	0.207863	-2.023533
H	1.583408	3.123792	0.02579
H	-3.695184	-0.943938	-1.140806
H	-0.544409	-0.373691	1.790011
Na	-0.846454	-1.867016	-0.099131

95. Reactant of 2nd Gal-L1 C=O Migration

25

C 2.765074 -0.2094 -0.369361
C 1.639299 -0.005644 0.491258
C 0.880204 1.324566 0.40702
O 1.711279 2.22453 -0.287509
C -0.46407 1.107324 -0.320537
C -1.642337 0.811657 0.629861
C -2.948656 0.559336 -0.110323
O -2.823314 -0.663051 -0.848948
O -1.457235 -0.318118 1.461648
O -0.253479 0.089017 -1.297042
O 1.059078 -1.073241 0.952872
O 3.214418 -1.413764 -0.465246
H -1.784746 1.714584 1.239363
H 3.313818 0.583148 -0.86385
H 2.679564 0.321828 1.092085
H 0.681164 1.677119 1.426029
H -0.691695 2.054806 -0.821529
H 2.579436 -1.981978 0.028852
H -3.748177 0.470584 0.628133
H -3.171481 1.393032 -0.78382
H -0.873757 0.207863 -2.023533
H 1.583408 3.123792 0.02579
H -3.695184 -0.943938 -1.140806
H -0.544409 -0.373691 1.790011
Na -0.846454 -1.867016 -0.099131

96. Transition States of 3rd Gal-L1 C=O Migration

25

C 2.847935 -0.728633 -0.148004
C 1.49026 -0.243011 -0.202871
C 0.402224 -1.273723 0.098945
O 0.12392 -1.16011 1.481501
C -0.886017 -1.259918 -0.741326
C -1.846153 -0.053174 -0.707226
C -2.309217 0.410007 0.672348
O -1.324371 1.164791 1.355026
O -1.299908 1.07792 -1.388032
O -1.575637 -2.387986 -0.22576
O 1.369445 1.026507 -0.029156
O 3.770832 0.158105 -0.072644
H -2.748086 -0.389604 -1.234455
H 3.151905 -1.765777 -0.251106
H 1.866581 -0.584297 -1.355579
H 0.835278 -2.262593 -0.103609
H -0.588686 -1.423045 -1.786846
H 3.292667 1.011029 0.087756
H -3.168513 1.068914 0.540393
H -2.628933 -0.455289 1.258378
H -2.248449 -2.700668 -0.837163
H -0.408296 -1.923527 1.740402
H -0.680994 0.539224 1.724989
H -1.465865 1.011919 -2.331928
Na -0.174277 2.520964 -0.02272

97. Reactant of 3rd Gal-L1 C=O Migration

25

```
C   2.847935   -0.728633   -0.148004
C   1.49026   -0.243011   -0.202871
C   0.402224   -1.273723   0.098945
O   0.12392   -1.16011   1.481501
C   -0.886017   -1.259918   -0.741326
C   -1.846153   -0.053174   -0.707226
C   -2.309217   0.410007   0.672348
O   -1.324371   1.164791   1.355026
O   -1.299908   1.07792   -1.388032
O   -1.575637   -2.387986   -0.22576
O   1.369445   1.026507   -0.029156
O   3.770832   0.158105   -0.072644
H   -2.748086   -0.389604   -1.234455
H   3.151905   -1.765777   -0.251106
H   1.866581   -0.584297   -1.355579
H   0.835278   -2.262593   -0.103609
H   -0.588686   -1.423045   -1.786846
H   3.292667   1.011029   0.087756
H   -3.168513   1.068914   0.540393
H   -2.628933   -0.455289   1.258378
H   -2.248449   -2.700668   -0.837163
H   -0.408296   -1.923527   1.740402
H   -0.680994   0.539224   1.724989
H   -1.465865   1.011919   -2.331928
Na   -0.174277   2.520964   -0.02272
```

98. Transition States of 4th Gal-L1 C=O Migration

25

C 2.702547 0.474797 -0.70987
C 1.832114 0.116387 0.374696
C 0.625682 1.007927 0.685056
O 0.914879 2.378791 0.518706
C -0.559504 0.700297 -0.262504
C -1.865248 0.576473 0.528061
C -3.101654 0.618323 -0.356171
O -2.937128 -0.313661 -1.415118
O -1.760362 -0.653491 1.255919
O -0.307851 -0.491751 -0.99466
O 1.821891 -1.131965 0.71684
O 3.498169 -0.440769 -1.1343
H -1.923095 1.420363 1.226025
H 2.81017 1.476236 -1.113613
H 2.781022 0.763713 0.841765
H 0.344 0.778373 1.717946
H -0.631534 1.552566 -0.945956
H 3.214567 -1.268777 -0.673464
H -3.989108 0.378168 0.239981
H -3.211325 1.636609 -0.744876
H -1.054022 -0.600614 -1.604321
H 1.242657 2.758072 1.339183
H -3.709134 -0.308222 -1.987603
H -2.469678 -0.710034 1.903157
Na -0.094993 -2.147173 0.606097

99. Reactant of 4th Gal-L1 C=O Migration

25

C 2.702547 0.474797 -0.70987
C 1.832114 0.116387 0.374696
C 0.625682 1.007927 0.685056
O 0.914879 2.378791 0.518706
C -0.559504 0.700297 -0.262504
C -1.865248 0.576473 0.528061
C -3.101654 0.618323 -0.356171
O -2.937128 -0.313661 -1.415118
O -1.760362 -0.653491 1.255919
O -0.307851 -0.491751 -0.99466
O 1.821891 -1.131965 0.71684
O 3.498169 -0.440769 -1.1343
H -1.923095 1.420363 1.226025
H 2.81017 1.476236 -1.113613
H 2.781022 0.763713 0.841765
H 0.344 0.778373 1.717946
H -0.631534 1.552566 -0.945956
H 3.214567 -1.268777 -0.673464
H -3.989108 0.378168 0.239981
H -3.211325 1.636609 -0.744876
H -1.054022 -0.600614 -1.604321
H 1.242657 2.758072 1.339183
H -3.709134 -0.308222 -1.987603
H -2.469678 -0.710034 1.903157
Na -0.094993 -2.147173 0.606097

100. Transition States of 1st Gal-L2 C-C cleavage

25

C 3.17324 -0.460036 -0.675827
C 2.000481 -0.710638 0.226367
C 1.262726 0.248771 0.905493
O 1.552932 1.571697 0.98442
C -0.25911 0.003018 -0.447102
C -1.191362 1.028119 0.20123
C -2.282315 1.443575 -0.783922
O -3.094389 0.300952 -1.051534
O -1.782061 0.400673 1.338245
O -0.616527 -1.22814 -0.42731
O 1.597554 -1.953639 0.243574
O 3.513569 0.911283 -0.663753
H -0.618634 1.91473 0.48308
H 2.900744 -0.804867 -1.681369
H 0.628374 -0.102787 1.712808
H 0.239165 0.400323 -1.343331
H -2.887584 2.237659 -0.335743
H -1.824869 1.821926 -1.702522
H 0.579064 -1.92906 0.022365
H 2.345828 1.763242 0.458815
H -3.714035 0.499627 -1.758676
H -2.017229 1.060051 1.997249
H 4.363536 1.05725 -1.086972
H 3.99179 -1.093927 -0.316067
Na -2.688793 -1.469421 0.327617

101. Reactant of 1st Gal-L2 C-C cleavage

25

C 3.17324 -0.460036 -0.675827
C 2.000481 -0.710638 0.226367
C 1.262726 0.248771 0.905493
O 1.552932 1.571697 0.98442
C -0.25911 0.003018 -0.447102
C -1.191362 1.028119 0.20123
C -2.282315 1.443575 -0.783922
O -3.094389 0.300952 -1.051534
O -1.782061 0.400673 1.338245
O -0.616527 -1.22814 -0.42731
O 1.597554 -1.953639 0.243574
O 3.513569 0.911283 -0.663753
H -0.618634 1.91473 0.48308
H 2.900744 -0.804867 -1.681369
H 0.628374 -0.102787 1.712808
H 0.239165 0.400323 -1.343331
H -2.887584 2.237659 -0.335743
H -1.824869 1.821926 -1.702522
H 0.579064 -1.92906 0.022365
H 2.345828 1.763242 0.458815
H -3.714035 0.499627 -1.758676
H -2.017229 1.060051 1.997249
H 4.363536 1.05725 -1.086972
H 3.99179 -1.093927 -0.316067
Na -2.688793 -1.469421 0.327617

102. Transition States of 2nd Gal-L2 C-C cleavage

25

C 2.055334 1.457527 0.56269
C 1.906479 -0.027921 0.36312
C 1.800825 -0.642738 -0.877365
O 2.080904 -1.960143 -1.033681
C -0.224577 -0.806421 -0.543989
C -0.951898 0.486433 -0.90141
C -2.381694 0.190414 -1.343053
O -3.081943 -0.351191 -0.222412
O -1.007836 1.344102 0.230255
O -0.390067 -1.295836 0.646094
O 1.77239 -0.765092 1.419824
O 1.501561 2.160663 -0.53942
H -0.439473 0.97645 -1.732645
H 3.123242 1.670157 0.678685
H 1.876516 -0.065272 -1.790402
H -0.169434 -1.528591 -1.365561
H -2.849873 1.123986 -1.664964
H -2.374147 -0.520926 -2.173682
H 0.753651 -1.208906 1.253461
H 2.307135 -2.353624 -0.180099
H -3.962335 -0.62807 -0.49009
H -0.349265 2.040413 0.101249
H 2.034358 2.932459 -0.750228
H 1.556508 1.728783 1.497894
Na -1.975797 -0.106422 1.731921

103. Reactant of 2nd Gal-L2 C-C cleavage

25

C 2.055334 1.457527 0.56269
C 1.906479 -0.027921 0.36312
C 1.800825 -0.642738 -0.877365
O 2.080904 -1.960143 -1.033681
C -0.224577 -0.806421 -0.543989
C -0.951898 0.486433 -0.90141
C -2.381694 0.190414 -1.343053
O -3.081943 -0.351191 -0.222412
O -1.007836 1.344102 0.230255
O -0.390067 -1.295836 0.646094
O 1.77239 -0.765092 1.419824
O 1.501561 2.160663 -0.53942
H -0.439473 0.97645 -1.732645
H 3.123242 1.670157 0.678685
H 1.876516 -0.065272 -1.790402
H -0.169434 -1.528591 -1.365561
H -2.849873 1.123986 -1.664964
H -2.374147 -0.520926 -2.173682
H 0.753651 -1.208906 1.253461
H 2.307135 -2.353624 -0.180099
H -3.962335 -0.62807 -0.49009
H -0.349265 2.040413 0.101249
H 2.034358 2.932459 -0.750228
H 1.556508 1.728783 1.497894
Na -1.975797 -0.106422 1.731921

104. Transition States of 3rd Gal-L2 C-C cleavage

25

C 1.642189 -1.401982 0.935297
C 1.879276 -0.164961 0.107308
C 1.753045 1.131788 0.590089
O 2.301474 2.189177 -0.052156
C -0.168359 1.14122 -0.322883
C -1.18382 0.961917 0.794614
C -2.606249 1.157226 0.257367
O -2.934453 0.009946 -0.518979
O -1.045 -0.346625 1.332478
O -0.203502 0.338886 -1.3381
O 2.029555 -0.334868 -1.165295
O 0.808616 -2.315398 0.223762
H -0.989371 1.72339 1.5561
H 1.161721 -1.165766 1.880797
H 1.56739 1.318148 1.642739
H 0.062493 2.191752 -0.530016
H -3.300607 1.251837 1.099274
H -2.654354 2.071776 -0.340813
H 0.978443 -0.015417 -1.542531
H 2.704509 1.890699 -0.879896
H -3.729792 0.181648 -1.030763
H -1.620441 -0.448313 2.098803
H 1.368893 -2.828354 -0.367738
H 2.613446 -1.86249 1.143138
Na -1.246575 -1.599009 -0.613956

105. Reactant of 3rd Gal-L2 C-C cleavage

25

C 1.642189 -1.401982 0.935297
C 1.879276 -0.164961 0.107308
C 1.753045 1.131788 0.590089
O 2.301474 2.189177 -0.052156
C -0.168359 1.14122 -0.322883
C -1.18382 0.961917 0.794614
C -2.606249 1.157226 0.257367
O -2.934453 0.009946 -0.518979
O -1.045 -0.346625 1.332478
O -0.203502 0.338886 -1.3381
O 2.029555 -0.334868 -1.165295
O 0.808616 -2.315398 0.223762
H -0.989371 1.72339 1.5561
H 1.161721 -1.165766 1.880797
H 1.56739 1.318148 1.642739
H 0.062493 2.191752 -0.530016
H -3.300607 1.251837 1.099274
H -2.654354 2.071776 -0.340813
H 0.978443 -0.015417 -1.542531
H 2.704509 1.890699 -0.879896
H -3.729792 0.181648 -1.030763
H -1.620441 -0.448313 2.098803
H 1.368893 -2.828354 -0.367738
H 2.613446 -1.86249 1.143138
Na -1.246575 -1.599009 -0.613956

106. Transition States of 4th Gal-L2 C-C cleavage

25

C -1.595684 -1.038339 -1.321704
C -1.707894 0.163634 -0.41453
C -1.32411 1.437297 -0.809279
O -1.795013 2.548878 -0.19924
C 0.404516 1.153754 0.353875
C 1.451349 0.384821 -0.415279
C 2.850412 0.726783 0.09617
O 3.692197 -0.183146 -0.583835
O 1.216512 -1.002989 -0.243214
O 0.021575 0.687528 1.500872
O -2.085903 -0.003818 0.81547
O -1.375267 -2.211862 -0.554996
H 1.404692 0.641472 -1.480332
H -0.78233 -0.895865 -2.038312
H -0.939258 1.621335 -1.806574
H 0.474585 2.24141 0.256373
H 3.091648 1.76709 -0.142135
H 2.891123 0.579415 1.181239
H -1.192261 0.456832 1.397186
H -2.41628 2.313087 0.502661
H 4.610516 -0.056706 -0.330888
H 2.001951 -1.457693 -0.580998
H -2.025989 -2.880213 -0.782186
H -2.533016 -1.11559 -1.881478
Na -0.222702 -1.714454 1.353587

107. Reactant of 4th Gal-L2 C-C cleavage

25

C -1.595684 -1.038339 -1.321704
C -1.707894 0.163634 -0.41453
C -1.32411 1.437297 -0.809279
O -1.795013 2.548878 -0.19924
C 0.404516 1.153754 0.353875
C 1.451349 0.384821 -0.415279
C 2.850412 0.726783 0.09617
O 3.692197 -0.183146 -0.583835
O 1.216512 -1.002989 -0.243214
O 0.021575 0.687528 1.500872
O -2.085903 -0.003818 0.81547
O -1.375267 -2.211862 -0.554996
H 1.404692 0.641472 -1.480332
H -0.78233 -0.895865 -2.038312
H -0.939258 1.621335 -1.806574
H 0.474585 2.24141 0.256373
H 3.091648 1.76709 -0.142135
H 2.891123 0.579415 1.181239
H -1.192261 0.456832 1.397186
H -2.41628 2.313087 0.502661
H 4.610516 -0.056706 -0.330888
H 2.001951 -1.457693 -0.580998
H -2.025989 -2.880213 -0.782186
H -2.533016 -1.11559 -1.881478
Na -0.222702 -1.714454 1.353587

108. Transition States of 1st Gal-L2 C=O Migration

25

C	-2.77908	-1.273215	0.065618
C	-2.196568	0.106174	0.086643
C	-0.902024	0.617862	-0.308412
O	-0.747424	1.879757	-0.07131
C	0.330662	-0.260436	-0.526358
C	1.03591	-0.570009	0.807785
C	2.032348	-1.707811	0.640572
O	2.859379	-1.414575	-0.474491
O	1.668059	0.634845	1.238041
O	1.180856	0.491611	-1.378474
O	-3.010357	1.029221	0.495833
O	-1.812307	-2.200102	-0.356701
H	0.282777	-0.884607	1.539981
H	-3.66516	-1.260903	-0.577674
H	-1.630222	0.383777	-1.317655
H	0.046342	-1.202676	-0.992285
H	2.628872	-1.81432	1.554074
H	1.471058	-2.635192	0.481209
H	1.961502	-0.059762	-1.539037
H	-2.44973	1.851789	0.531841
H	3.506099	-2.112633	-0.607384
H	2.086287	0.493313	2.092341
H	-2.232794	-3.048762	-0.521687
H	-3.115741	-1.461408	1.092958
Na	1.352507	2.446358	-0.177724

109. Reactant of 1st Gal-L2 C=O Migration

25

C -2.77908 -1.273215 0.065618
C -2.196568 0.106174 0.086643
C -0.902024 0.617862 -0.308412
O -0.747424 1.879757 -0.07131
C 0.330662 -0.260436 -0.526358
C 1.03591 -0.570009 0.807785
C 2.032348 -1.707811 0.640572
O 2.859379 -1.414575 -0.474491
O 1.668059 0.634845 1.238041
O 1.180856 0.491611 -1.378474
O -3.010357 1.029221 0.495833
O -1.812307 -2.200102 -0.356701
H 0.282777 -0.884607 1.539981
H -3.66516 -1.260903 -0.577674
H -1.630222 0.383777 -1.317655
H 0.046342 -1.202676 -0.992285
H 2.628872 -1.81432 1.554074
H 1.471058 -2.635192 0.481209
H 1.961502 -0.059762 -1.539037
H -2.44973 1.851789 0.531841
H 3.506099 -2.112633 -0.607384
H 2.086287 0.493313 2.092341
H -2.232794 -3.048762 -0.521687
H -3.115741 -1.461408 1.092958
Na 1.352507 2.446358 -0.177724

110. Transition States of 2nd Gal-L2 C=O Migration

25

C 2.887062 0.055125 -0.117549
C 1.652105 0.886791 -0.300099
C 0.339187 0.793351 0.283797
O -0.518352 1.651616 -0.157574
C -0.171714 -0.47146 0.965481
C -0.510592 -1.540218 -0.097372
C -1.396613 -1.074766 -1.25013
O -2.664113 -0.600548 -0.782225
O 0.678451 -1.953252 -0.730688
O -1.314293 -0.075691 1.7182
O 1.802113 1.883541 -1.123626
O 2.723628 -0.870708 0.933135
H -1.006397 -2.37322 0.419872
H 3.7241 0.73673 0.056804
H 1.205736 1.300472 1.082732
H 0.591033 -0.881072 1.628071
H -0.894093 -0.274791 -1.798376
H -1.527723 -1.922606 -1.92396
H -1.500441 -0.737912 2.389486
H 0.89974 2.271479 -1.233872
H -3.36736 -1.060428 -1.245424
H 1.307225 -2.275497 -0.074533
H 3.581723 -1.08017 1.312469
H 3.04581 -0.447635 -1.078857
Na -2.592312 1.324731 0.42755

111. Reactant of 2nd Gal-L2 C=O Migration

25

C 2.887062 0.055125 -0.117549
C 1.652105 0.886791 -0.300099
C 0.339187 0.793351 0.283797
O -0.518352 1.651616 -0.157574
C -0.171714 -0.47146 0.965481
C -0.510592 -1.540218 -0.097372
C -1.396613 -1.074766 -1.25013
O -2.664113 -0.600548 -0.782225
O 0.678451 -1.953252 -0.730688
O -1.314293 -0.075691 1.7182
O 1.802113 1.883541 -1.123626
O 2.723628 -0.870708 0.933135
H -1.006397 -2.37322 0.419872
H 3.7241 0.73673 0.056804
H 1.205736 1.300472 1.082732
H 0.591033 -0.881072 1.628071
H -0.894093 -0.274791 -1.798376
H -1.527723 -1.922606 -1.92396
H -1.500441 -0.737912 2.389486
H 0.89974 2.271479 -1.233872
H -3.36736 -1.060428 -1.245424
H 1.307225 -2.275497 -0.074533
H 3.581723 -1.08017 1.312469
H 3.04581 -0.447635 -1.078857
Na -2.592312 1.324731 0.42755

112. Transition States of 3rd Gal-L2 C=O Migration

25

C 2.882859 0.040206 -0.120064
C 1.655252 0.882611 -0.299738
C 0.345 0.794565 0.292127
O -0.508084 1.663564 -0.136448
C -0.179437 -0.477227 0.965119
C -0.508271 -1.537952 -0.108923
C -1.4039 -1.064444 -1.252599
O -2.672512 -0.586349 -0.804932
O 0.683344 -1.928889 -0.751378
O -1.353236 -0.145226 1.700838
O 1.81001 1.880008 -1.121508
O 2.715251 -0.869507 0.944535
H -0.988119 -2.377591 0.410354
H 3.72861 0.714515 0.040213
H 1.223807 1.290816 1.087513
H 0.575401 -0.902459 1.625392
H -0.91896 -0.24271 -1.780947
H -1.526233 -1.89117 -1.954382
H -1.175651 -0.204275 2.642786
H 0.91141 2.276942 -1.226739
H -3.300063 -1.312974 -0.776689
H 1.294506 -2.307475 -0.109458
H 3.57481 -1.093929 1.31182
H 3.027981 -0.476513 -1.075939
Na -2.597699 1.326496 0.391063

113. Reactant of 3rd Gal-L2 C=O Migration

25

C 2.882859 0.040206 -0.120064
C 1.655252 0.882611 -0.299738
C 0.345 0.794565 0.292127
O -0.508084 1.663564 -0.136448
C -0.179437 -0.477227 0.965119
C -0.508271 -1.537952 -0.108923
C -1.4039 -1.064444 -1.252599
O -2.672512 -0.586349 -0.804932
O 0.683344 -1.928889 -0.751378
O -1.353236 -0.145226 1.700838
O 1.81001 1.880008 -1.121508
O 2.715251 -0.869507 0.944535
H -0.988119 -2.377591 0.410354
H 3.72861 0.714515 0.040213
H 1.223807 1.290816 1.087513
H 0.575401 -0.902459 1.625392
H -0.91896 -0.24271 -1.780947
H -1.526233 -1.89117 -1.954382
H -1.175651 -0.204275 2.642786
H 0.91141 2.276942 -1.226739
H -3.300063 -1.312974 -0.776689
H 1.294506 -2.307475 -0.109458
H 3.57481 -1.093929 1.31182
H 3.027981 -0.476513 -1.075939
Na -2.597699 1.326496 0.391063

114. Transition States of 4th Gal-L2 C=O Migration

25

C	-2.900728	-0.219157	0.066248
C	-1.774015	0.749454	0.24095
C	-0.446125	0.753367	-0.305026
O	0.316409	1.702627	0.121016
C	0.173325	-0.492264	-0.958675
C	0.567206	-1.56192	0.099949
C	2.02681	-1.502477	0.530467
O	2.363207	-0.230852	1.068502
O	-0.278418	-1.358114	1.224729
O	1.339656	-0.08195	-1.671968
O	-2.041287	1.753938	1.023064
O	-2.603863	-1.122756	-0.967886
H	0.382794	-2.547905	-0.340018
H	-3.81256	0.354741	-0.126335
H	-1.33475	1.158091	-1.148757
H	-0.545396	-0.938801	-1.639643
H	2.20833	-2.286291	1.273384
H	2.686365	-1.678075	-0.317604
H	1.186785	-0.154544	-2.617275
H	-1.182165	2.227691	1.13966
H	1.743147	-0.037049	1.782831
H	-0.329632	-2.167732	1.74158
H	-3.380884	-1.654251	-1.161045
H	-3.001859	-0.718291	1.037512
Na	2.430487	1.525205	-0.367948

115. Reactant of 4th Gal-L2 C=O Migration

25

C -2.900728 -0.219157 0.066248
C -1.774015 0.749454 0.24095
C -0.446125 0.753367 -0.305026
O 0.316409 1.702627 0.121016
C 0.173325 -0.492264 -0.958675
C 0.567206 -1.56192 0.099949
C 2.02681 -1.502477 0.530467
O 2.363207 -0.230852 1.068502
O -0.278418 -1.358114 1.224729
O 1.339656 -0.08195 -1.671968
O -2.041287 1.753938 1.023064
O -2.603863 -1.122756 -0.967886
H 0.382794 -2.547905 -0.340018
H -3.81256 0.354741 -0.126335
H -1.33475 1.158091 -1.148757
H -0.545396 -0.938801 -1.639643
H 2.20833 -2.286291 1.273384
H 2.686365 -1.678075 -0.317604
H 1.186785 -0.154544 -2.617275
H -1.182165 2.227691 1.13966
H 1.743147 -0.037049 1.782831
H -0.329632 -2.167732 1.74158
H -3.380884 -1.654251 -1.161045
H -3.001859 -0.718291 1.037512
Na 2.430487 1.525205 -0.367948

116. Transition States of 1st Gal-L3a C-C cleavage

25

C 2.109404 1.008989 -0.453952
C 1.274177 1.014886 0.810544
C -0.234317 1.185023 0.680062
O -0.864968 0.995863 1.788806
C -0.991543 1.442639 -0.461715
C -2.178667 -0.229373 -0.390511
C -1.37837 -1.341062 -1.038933
O -0.562843 -1.966154 -0.063625
O -2.490296 -0.407629 0.836211
O -0.392927 1.487464 -1.70948
O 1.55626 -0.19525 1.509518
O 1.796201 -0.112471 -1.27495
H -2.898046 0.260234 -1.055682
H 3.157865 0.918216 -0.165472
H -1.87692 2.052531 -0.322145
H -2.095427 -2.059982 -1.453977
H -0.752439 -0.976207 -1.852473
H -0.73649 2.221421 -2.228277
H -1.111073 -2.028211 0.73428
H -1.769008 0.389143 1.546071
H 1.133315 0.180347 -1.912542
H 1.982197 1.943026 -1.000591
H 1.084287 -0.173095 2.351695
H 1.598223 1.870413 1.419527
Na 1.667999 -1.954459 0.034069

117. Reactant of 1st Gal-L3a C-C cleavage

25

C 2.109404 1.008989 -0.453952
C 1.274177 1.014886 0.810544
C -0.234317 1.185023 0.680062
O -0.864968 0.995863 1.788806
C -0.991543 1.442639 -0.461715
C -2.178667 -0.229373 -0.390511
C -1.37837 -1.341062 -1.038933
O -0.562843 -1.966154 -0.063625
O -2.490296 -0.407629 0.836211
O -0.392927 1.487464 -1.70948
O 1.55626 -0.19525 1.509518
O 1.796201 -0.112471 -1.27495
H -2.898046 0.260234 -1.055682
H 3.157865 0.918216 -0.165472
H -1.87692 2.052531 -0.322145
H -2.095427 -2.059982 -1.453977
H -0.752439 -0.976207 -1.852473
H -0.73649 2.221421 -2.228277
H -1.111073 -2.028211 0.73428
H -1.769008 0.389143 1.546071
H 1.133315 0.180347 -1.912542
H 1.982197 1.943026 -1.000591
H 1.084287 -0.173095 2.351695
H 1.598223 1.870413 1.419527
Na 1.667999 -1.954459 0.034069

118. Transition States of 2nd Gal-L3a C-C cleavage

25

C -2.111461 1.00522 0.458865
C -1.276527 1.016759 -0.805786
C 0.231762 1.188516 -0.675142
O 0.861589 1.006617 -1.785663
C 0.989507 1.441483 0.467229
C 2.181848 -0.228184 0.385873
C 1.385641 -1.342147 1.03524
O 0.56673 -1.965932 0.062086
O 2.48745 -0.402559 -0.84283
O 0.391119 1.478804 1.715279
O -1.556513 -0.191569 -1.508999
O -1.798451 -0.12052 1.273969
H 2.904164 0.260555 1.048477
H -3.160126 0.916615 0.170551
H 1.872973 2.054642 0.329995
H 2.105216 -2.061132 1.445794
H 0.762752 -0.979568 1.852191
H 0.740616 2.20459 2.241492
H 1.109557 -2.016761 -0.740473
H 1.765972 0.399261 -1.547581
H -1.133489 0.16834 1.910974
H -1.983096 1.936361 1.010161
H -1.090998 -0.162444 -2.354479
H -1.601829 1.873755 -1.411953
Na -1.664701 -1.95662 -0.042141

119. Reactant of 2nd Gal-L3a C-C cleavage

25

C -2.111461 1.00522 0.458865
C -1.276527 1.016759 -0.805786
C 0.231762 1.188516 -0.675142
O 0.861589 1.006617 -1.785663
C 0.989507 1.441483 0.467229
C 2.181848 -0.228184 0.385873
C 1.385641 -1.342147 1.03524
O 0.56673 -1.965932 0.062086
O 2.48745 -0.402559 -0.84283
O 0.391119 1.478804 1.715279
O -1.556513 -0.191569 -1.508999
O -1.798451 -0.12052 1.273969
H 2.904164 0.260555 1.048477
H -3.160126 0.916615 0.170551
H 1.872973 2.054642 0.329995
H 2.105216 -2.061132 1.445794
H 0.762752 -0.979568 1.852191
H 0.740616 2.20459 2.241492
H 1.109557 -2.016761 -0.740473
H 1.765972 0.399261 -1.547581
H -1.133489 0.16834 1.910974
H -1.983096 1.936361 1.010161
H -1.090998 -0.162444 -2.354479
H -1.601829 1.873755 -1.411953
Na -1.664701 -1.95662 -0.042141

120. Transition States of 3rd Gal-L3a C-C cleavage

25

C -1.001943 -0.186083 1.63508
C -1.242328 0.981194 0.685264
C -0.287103 0.975792 -0.500807
O -0.592842 0.244243 -1.529998
C 0.933654 1.623639 -0.461246
C 2.117329 -0.129609 -0.740601
C 2.406059 -0.681394 0.642004
O 1.579138 -1.778775 0.966248
O 1.477049 -0.85442 -1.57718
O 1.30078 2.210137 0.722029
O -2.572529 0.816344 0.188802
O -1.112838 -1.407492 0.916231
H 2.89752 0.537275 -1.121765
H -1.765572 -0.161449 2.414576
H 1.359438 1.994582 -1.385743
H 3.462198 -0.977212 0.659094
H 2.275585 0.090058 1.403989
H 1.768807 3.037523 0.574384
H 1.851477 -2.534051 0.433413
H 0.389531 -0.371821 -1.755047
H -0.214076 -1.777795 0.880792
H -0.01671 -0.098498 2.099099
H -2.950671 1.666886 -0.0527
H -1.134549 1.920511 1.232364
Na -2.373368 -1.151594 -0.948151

121. Reactant of 3rd Gal-L3a C-C cleavage

25

C -1.001943 -0.186083 1.63508
C -1.242328 0.981194 0.685264
C -0.287103 0.975792 -0.500807
O -0.592842 0.244243 -1.529998
C 0.933654 1.623639 -0.461246
C 2.117329 -0.129609 -0.740601
C 2.406059 -0.681394 0.642004
O 1.579138 -1.778775 0.966248
O 1.477049 -0.85442 -1.57718
O 1.30078 2.210137 0.722029
O -2.572529 0.816344 0.188802
O -1.112838 -1.407492 0.916231
H 2.89752 0.537275 -1.121765
H -1.765572 -0.161449 2.414576
H 1.359438 1.994582 -1.385743
H 3.462198 -0.977212 0.659094
H 2.275585 0.090058 1.403989
H 1.768807 3.037523 0.574384
H 1.851477 -2.534051 0.433413
H 0.389531 -0.371821 -1.755047
H -0.214076 -1.777795 0.880792
H -0.01671 -0.098498 2.099099
H -2.950671 1.666886 -0.0527
H -1.134549 1.920511 1.232364
Na -2.373368 -1.151594 -0.948151

122. Transition States of 4th Gal-L3a C-C cleavage

25

C -0.920179 -0.143315 1.653475
C -1.270867 0.988439 0.693878
C -0.371395 0.976328 -0.535998
O -0.734721 0.241942 -1.541476
C 0.86789 1.588214 -0.544979
C 2.017628 -0.247003 -0.93632
C 2.629787 -0.594636 0.39177
O 1.761456 -1.425944 1.130569
O 1.203724 -1.049584 -1.502365
O 1.304141 2.174034 0.616157
O -2.616905 0.751115 0.278524
O -0.961537 -1.383206 0.955044
H 2.674975 0.361132 -1.565977
H -1.665416 -0.165283 2.450666
H 1.26962 1.940766 -1.48721
H 3.567039 -1.115864 0.155278
H 2.872736 0.324226 0.9361
H 1.713029 3.027511 0.44263
H 2.255075 -1.90486 1.801987
H 0.214087 -0.46879 -1.752434
H -0.043618 -1.684951 0.849184
H 0.069173 0.026227 2.079392
H -3.025812 1.568838 -0.020129
H -1.176666 1.945019 1.213219
Na -2.371881 -1.24809 -0.811391

123. Reactant of 4th Gal-L3a C-C cleavage

25

C -0.920179 -0.143315 1.653475
C -1.270867 0.988439 0.693878
C -0.371395 0.976328 -0.535998
O -0.734721 0.241942 -1.541476
C 0.86789 1.588214 -0.544979
C 2.017628 -0.247003 -0.93632
C 2.629787 -0.594636 0.39177
O 1.761456 -1.425944 1.130569
O 1.203724 -1.049584 -1.502365
O 1.304141 2.174034 0.616157
O -2.616905 0.751115 0.278524
O -0.961537 -1.383206 0.955044
H 2.674975 0.361132 -1.565977
H -1.665416 -0.165283 2.450666
H 1.26962 1.940766 -1.48721
H 3.567039 -1.115864 0.155278
H 2.872736 0.324226 0.9361
H 1.713029 3.027511 0.44263
H 2.255075 -1.90486 1.801987
H 0.214087 -0.46879 -1.752434
H -0.043618 -1.684951 0.849184
H 0.069173 0.026227 2.079392
H -3.025812 1.568838 -0.020129
H -1.176666 1.945019 1.213219
Na -2.371881 -1.24809 -0.811391

124. Transition States of 1st Gal-L3a C=O Migration

25

C -2.873411 -0.236748 0.822448
C -2.364515 -0.020612 -0.605743
C -1.160278 0.8803 -0.526011
O -1.411574 2.151719 -0.484719
C 0.236827 0.547448 -0.454939
C 0.704562 -0.866279 -0.068183
C 1.867809 -1.39337 -0.888954
O 3.028064 -0.598303 -0.649632
O 1.129958 -0.782155 1.287761
O 1.011817 1.562943 -0.255286
O -1.988577 -1.216714 -1.241694
O -1.81285 -0.77191 1.60166
H -0.11773 -1.572878 -0.171702
H -3.719417 -0.929352 0.780748
H -0.217761 0.443713 -1.645169
H 2.055758 -2.425351 -0.581414
H 1.596974 -1.374289 -1.948746
H -0.529113 2.568742 -0.291333
H 3.76206 -0.939153 -1.16712
H 0.326798 -0.757514 1.824867
H -2.178481 -1.176187 2.393677
H -3.217143 0.719769 1.228843
H -2.771446 -1.699059 -1.52359
H -3.134473 0.520281 -1.167077
Na 2.729391 0.944747 0.957416

125. Reactant of 1st Gal-L3a C=O Migration

25

C -2.873411 -0.236748 0.822448
C -2.364515 -0.020612 -0.605743
C -1.160278 0.8803 -0.526011
O -1.411574 2.151719 -0.484719
C 0.236827 0.547448 -0.454939
C 0.704562 -0.866279 -0.068183
C 1.867809 -1.39337 -0.888954
O 3.028064 -0.598303 -0.649632
O 1.129958 -0.782155 1.287761
O 1.011817 1.562943 -0.255286
O -1.988577 -1.216714 -1.241694
O -1.81285 -0.77191 1.60166
H -0.11773 -1.572878 -0.171702
H -3.719417 -0.929352 0.780748
H -0.217761 0.443713 -1.645169
H 2.055758 -2.425351 -0.581414
H 1.596974 -1.374289 -1.948746
H -0.529113 2.568742 -0.291333
H 3.76206 -0.939153 -1.16712
H 0.326798 -0.757514 1.824867
H -2.178481 -1.176187 2.393677
H -3.217143 0.719769 1.228843
H -2.771446 -1.699059 -1.52359
H -3.134473 0.520281 -1.167077
Na 2.729391 0.944747 0.957416

126. Transition States of 2nd Gal-L3a C=O Migration

25

C -2.764868 -0.265595 0.920719
C -2.387907 0.112278 -0.521779
C -1.150254 0.973203 -0.440861
O -1.36348 2.2409 -0.271975
C 0.244998 0.609392 -0.443868
C 0.696643 -0.840746 -0.220328
C 1.898033 -1.261203 -1.051923
O 3.056338 -0.543523 -0.633934
O 1.057045 -0.938032 1.151697
O 1.044334 1.584128 -0.160241
O -2.16208 -1.073959 -1.246889
O -1.792463 -1.137431 1.46853
H -0.116893 -1.523516 -0.465641
H -3.751645 -0.733311 0.909602
H -0.241656 0.632688 -1.62763
H 2.052103 -2.332426 -0.898273
H 1.687792 -1.073009 -2.109153
H -0.463217 2.617369 -0.072252
H 3.817986 -0.844528 -1.135999
H 0.231498 -1.005171 1.655618
H -2.00743 -2.03875 1.207878
H -2.806598 0.626352 1.547511
H -2.648245 -1.059679 -2.075805
H -3.186214 0.724491 -0.951266
Na 2.681373 0.817168 1.100559

127. Reactant of 2nd Gal-L3a C=O Migration

25

C -2.764868 -0.265595 0.920719
C -2.387907 0.112278 -0.521779
C -1.150254 0.973203 -0.440861
O -1.36348 2.2409 -0.271975
C 0.244998 0.609392 -0.443868
C 0.696643 -0.840746 -0.220328
C 1.898033 -1.261203 -1.051923
O 3.056338 -0.543523 -0.633934
O 1.057045 -0.938032 1.151697
O 1.044334 1.584128 -0.160241
O -2.16208 -1.073959 -1.246889
O -1.792463 -1.137431 1.46853
H -0.116893 -1.523516 -0.465641
H -3.751645 -0.733311 0.909602
H -0.241656 0.632688 -1.62763
H 2.052103 -2.332426 -0.898273
H 1.687792 -1.073009 -2.109153
H -0.463217 2.617369 -0.072252
H 3.817986 -0.844528 -1.135999
H 0.231498 -1.005171 1.655618
H -2.00743 -2.03875 1.207878
H -2.806598 0.626352 1.547511
H -2.648245 -1.059679 -2.075805
H -3.186214 0.724491 -0.951266
Na 2.681373 0.817168 1.100559

128. Transition States of 3rd Gal-L3a C=O Migration

25

C -3.094543 0.245406 0.603995
C -2.096844 0.029313 -0.537313
C -0.90443 0.917456 -0.35639
O -1.09172 2.178911 -0.589639
C 0.461467 0.560701 -0.054494
C 0.890264 -0.787937 0.60815
C 1.519382 -1.734317 -0.394623
O 2.605511 -1.028092 -1.006031
O 1.904293 -0.500543 1.561331
O 1.267814 1.569453 0.030437
O -1.649486 -1.301745 -0.57168
O -4.079825 -0.73451 0.367954
H 0.040133 -1.282716 1.074449
H -3.493811 1.262431 0.5564
H 0.165467 0.259101 -1.243145
H 1.889483 -2.617004 0.132014
H 0.772892 -2.024286 -1.13679
H -0.229406 2.611642 -0.358299
H 2.972796 -1.554972 -1.720907
H 1.517219 -0.083297 2.337239
H -4.784281 -0.676504 1.018524
H -2.596988 0.087939 1.569287
H -2.434039 -1.863699 -0.512208
H -2.59034 0.307519 -1.476833
Na 3.315128 0.792938 0.200486

129. Reactant of 3rd Gal-L3a C=O Migration

25

C	-3.094543	0.245406	0.603995
C	-2.096844	0.029313	-0.537313
C	-0.90443	0.917456	-0.35639
O	-1.09172	2.178911	-0.589639
C	0.461467	0.560701	-0.054494
C	0.890264	-0.787937	0.60815
C	1.519382	-1.734317	-0.394623
O	2.605511	-1.028092	-1.006031
O	1.904293	-0.500543	1.561331
O	1.267814	1.569453	0.030437
O	-1.649486	-1.301745	-0.57168
O	-4.079825	-0.73451	0.367954
H	0.040133	-1.282716	1.074449
H	-3.493811	1.262431	0.5564
H	0.165467	0.259101	-1.243145
H	1.889483	-2.617004	0.132014
H	0.772892	-2.024286	-1.13679
H	-0.229406	2.611642	-0.358299
H	2.972796	-1.554972	-1.720907
H	1.517219	-0.083297	2.337239
H	-4.784281	-0.676504	1.018524
H	-2.596988	0.087939	1.569287
H	-2.434039	-1.863699	-0.512208
H	-2.59034	0.307519	-1.476833
Na	3.315128	0.792938	0.200486

130. Transition States of 4th Gal-L3a C=O Migration

25

C -3.053195 0.214218 0.678118
C -2.131765 0.052911 -0.533634
C -0.917364 0.91773 -0.379218
O -1.09113 2.184734 -0.588102
C 0.455292 0.539679 -0.134248
C 0.864948 -0.820759 0.485686
C 1.607693 -1.702159 -0.505979
O 2.763713 -0.976893 -0.928327
O 1.739008 -0.473967 1.560154
O 1.277437 1.533031 -0.074047
O -1.710987 -1.280702 -0.674565
O -4.073421 -0.730288 0.443896
H -0.012152 -1.352806 0.848386
H -3.432018 1.239449 0.718482
H 0.100218 0.257525 -1.31626
H 1.904972 -2.634393 -0.015119
H 0.952278 -1.933979 -1.349961
H -0.214127 2.599164 -0.376746
H 3.192589 -1.440096 -1.652822
H 1.710132 -1.159327 2.233398
H -4.741663 -0.690579 1.132845
H -2.501014 -0.012386 1.598778
H -2.503244 -1.831977 -0.620537
H -2.675989 0.392659 -1.423324
Na 3.2754 0.846337 0.422406

131. Reactant of 4th Gal-L3a C=O Migration

25

C -3.053195 0.214218 0.678118
C -2.131765 0.052911 -0.533634
C -0.917364 0.91773 -0.379218
O -1.09113 2.184734 -0.588102
C 0.455292 0.539679 -0.134248
C 0.864948 -0.820759 0.485686
C 1.607693 -1.702159 -0.505979
O 2.763713 -0.976893 -0.928327
O 1.739008 -0.473967 1.560154
O 1.277437 1.533031 -0.074047
O -1.710987 -1.280702 -0.674565
O -4.073421 -0.730288 0.443896
H -0.012152 -1.352806 0.848386
H -3.432018 1.239449 0.718482
H 0.100218 0.257525 -1.31626
H 1.904972 -2.634393 -0.015119
H 0.952278 -1.933979 -1.349961
H -0.214127 2.599164 -0.376746
H 3.192589 -1.440096 -1.652822
H 1.710132 -1.159327 2.233398
H -4.741663 -0.690579 1.132845
H -2.501014 -0.012386 1.598778
H -2.503244 -1.831977 -0.620537
H -2.675989 0.392659 -1.423324
Na 3.2754 0.846337 0.422406

132. Transition States of 1st Gal-L3b C-C cleavage

25

C -2.123342 1.204172 0.679227
C -0.821781 0.433291 0.84055
C -0.077425 0.398968 -0.489648
O -0.229029 -0.641749 -1.262238
C 0.823537 1.38189 -0.830463
C 2.377225 -0.055679 -0.517012
C 2.450373 -0.237895 0.983545
O 1.580748 -1.265951 1.411643
O 2.078606 -1.062029 -1.243694
O 1.010462 2.412992 0.045519
O -1.152501 -0.877072 1.258531
O -2.904437 0.503314 -0.294634
H 3.055473 0.69869 -0.930319
H -1.906684 2.221447 0.344564
H 1.11938 1.510228 -1.865475
H 3.491019 -0.475378 1.238769
H 2.183662 0.682189 1.50701
H 1.497816 3.133201 -0.364146
H 1.868204 -2.087513 0.995573
H 0.874923 -1.04782 -1.390239
H -3.694508 1.008476 -0.504639
H -2.644253 1.226393 1.638917
H -0.320579 -1.273968 1.564293
H -0.215547 0.949749 1.590171
Na -2.195566 -1.622746 -0.649336

133. Reactant of 1st Gal-L3b C-C cleavage

25

C	-2.123342	1.204172	0.679227
C	-0.821781	0.433291	0.84055
C	-0.077425	0.398968	-0.489648
O	-0.229029	-0.641749	-1.262238
C	0.823537	1.38189	-0.830463
C	2.377225	-0.055679	-0.517012
C	2.450373	-0.237895	0.983545
O	1.580748	-1.265951	1.411643
O	2.078606	-1.062029	-1.243694
O	1.010462	2.412992	0.045519
O	-1.152501	-0.877072	1.258531
O	-2.904437	0.503314	-0.294634
H	3.055473	0.69869	-0.930319
H	-1.906684	2.221447	0.344564
H	1.11938	1.510228	-1.865475
H	3.491019	-0.475378	1.238769
H	2.183662	0.682189	1.50701
H	1.497816	3.133201	-0.364146
H	1.868204	-2.087513	0.995573
H	0.874923	-1.04782	-1.390239
H	-3.694508	1.008476	-0.504639
H	-2.644253	1.226393	1.638917
H	-0.320579	-1.273968	1.564293
H	-0.215547	0.949749	1.590171
Na	-2.195566	-1.622746	-0.649336

134. Transition States of 2nd Gal-L3b C-C cleavage

25

C   -2.449621   0.62752   -0.300572
C   -1.16138   1.416968   -0.121294
C   -0.01572   0.84093   -0.946398
O   -0.278251   -0.092286   -1.805418
C   1.312566   1.198125   -0.745122
C   1.95443   -0.666324   -0.170285
C   1.656716   -0.721421   1.313352
O   0.302787   -1.055631   1.540542
O   1.361468   -1.51575   -0.939407
O   1.585604   2.123166   0.240074
O   -0.859535   1.439318   1.266193
O   -2.332077   -0.672573   0.282872
H   2.988637   -0.399892   -0.414667
H   -2.670565   0.503039   -1.35959
H   1.975359   1.165509   -1.602719
H   2.281022   -1.516807   1.734243
H   1.933816   0.217056   1.798958
H   2.311666   2.699297   -0.017248
H   -0.194215   -0.233164   1.688557
H   0.540089   -0.814009   -1.706957
H   -2.738341   -0.655153   1.154337
H   -3.265156   1.176714   0.17097
H   -0.20462   2.127975   1.437251
H   -1.332532   2.438023   -0.487643
Na   -0.695189   -2.244848   -0.113857

135. Reactant of 2nd Gal-L3b C-C cleavage

25

C -2.449621 0.62752 -0.300572
C -1.16138 1.416968 -0.121294
C -0.01572 0.84093 -0.946398
O -0.278251 -0.092286 -1.805418
C 1.312566 1.198125 -0.745122
C 1.95443 -0.666324 -0.170285
C 1.656716 -0.721421 1.313352
O 0.302787 -1.055631 1.540542
O 1.361468 -1.51575 -0.939407
O 1.585604 2.123166 0.240074
O -0.859535 1.439318 1.266193
O -2.332077 -0.672573 0.282872
H 2.988637 -0.399892 -0.414667
H -2.670565 0.503039 -1.35959
H 1.975359 1.165509 -1.602719
H 2.281022 -1.516807 1.734243
H 1.933816 0.217056 1.798958
H 2.311666 2.699297 -0.017248
H -0.194215 -0.233164 1.688557
H 0.540089 -0.814009 -1.706957
H -2.738341 -0.655153 1.154337
H -3.265156 1.176714 0.17097
H -0.20462 2.127975 1.437251
H -1.332532 2.438023 -0.487643
Na -0.695189 -2.244848 -0.113857

136. Transition States of 3rd Gal-L3b C-C cleavage

25

C -2.477016 0.532173 -0.285319
C -1.215534 1.369286 -0.138806
C -0.041303 0.833386 -0.942435
O -0.279354 -0.108316 -1.800341
C 1.273321 1.253532 -0.758669
C 1.965735 -0.578838 -0.174305
C 1.690845 -0.643936 1.312233
O 0.353998 -1.040458 1.558795
O 1.404342 -1.468637 -0.930467
O 1.662735 2.204324 0.127948
O -0.794235 1.498115 1.221241
O -2.283698 -0.763562 0.285536
H 2.983225 -0.267285 -0.428596
H -2.726332 0.446634 -1.345128
H 1.915613 1.208036 -1.630678
H 2.352684 -1.410374 1.729058
H 1.937391 0.303388 1.796748
H 1.00985 2.284737 0.834657
H -0.180431 -0.258291 1.755925
H 0.568412 -0.820138 -1.6789
H -3.101851 -1.040741 0.707006
H -3.294867 1.045198 0.227457
H -1.407906 2.058767 1.710456
H -1.433387 2.365374 -0.549985
Na -0.573545 -2.28006 -0.082355

137. Reactant of 3rd Gal-L3b C-C cleavage

25

C -2.477016 0.532173 -0.285319
C -1.215534 1.369286 -0.138806
C -0.041303 0.833386 -0.942435
O -0.279354 -0.108316 -1.800341
C 1.273321 1.253532 -0.758669
C 1.965735 -0.578838 -0.174305
C 1.690845 -0.643936 1.312233
O 0.353998 -1.040458 1.558795
O 1.404342 -1.468637 -0.930467
O 1.662735 2.204324 0.127948
O -0.794235 1.498115 1.221241
O -2.283698 -0.763562 0.285536
H 2.983225 -0.267285 -0.428596
H -2.726332 0.446634 -1.345128
H 1.915613 1.208036 -1.630678
H 2.352684 -1.410374 1.729058
H 1.937391 0.303388 1.796748
H 1.00985 2.284737 0.834657
H -0.180431 -0.258291 1.755925
H 0.568412 -0.820138 -1.6789
H -3.101851 -1.040741 0.707006
H -3.294867 1.045198 0.227457
H -1.407906 2.058767 1.710456
H -1.433387 2.365374 -0.549985
Na -0.573545 -2.28006 -0.082355

138. Transition States of 4th Gal-L3b C-C cleavage

25

C 2.720293 0.245193 -0.199563
C 1.433613 0.91607 -0.641662
C 0.492742 1.183985 0.53341
O 0.806229 0.726878 1.693376
C -0.794379 1.664764 0.316716
C -1.794148 -0.087846 1.035841
C -2.564355 -0.522156 -0.182949
O -1.738082 -1.315483 -1.018669
O -0.898838 -0.860681 1.533141
O -1.158453 1.93836 -0.988709
O 0.79674 0.018047 -1.546931
O 2.40611 -1.087576 0.21541
H -2.399842 0.49612 1.736113
H 3.162988 0.810975 0.622066
H -1.27886 2.230236 1.104177
H -3.410832 -1.110836 0.19403
H -2.950081 0.348626 -0.717704
H -1.504943 2.832137 -1.076826
H -2.252434 -1.624811 -1.770008
H 0.037313 -0.076598 1.903394
H 3.076821 -1.393471 0.831244
H 3.411824 0.212195 -1.043996
H 0.035896 0.465033 -1.940296
H 1.671229 1.861205 -1.141515
Na 0.338971 -1.891566 -0.270216

139. Reactant of 4th Gal-L3b C-C cleavage

25

C 2.720293 0.245193 -0.199563
C 1.433613 0.91607 -0.641662
C 0.492742 1.183985 0.53341
O 0.806229 0.726878 1.693376
C -0.794379 1.664764 0.316716
C -1.794148 -0.087846 1.035841
C -2.564355 -0.522156 -0.182949
O -1.738082 -1.315483 -1.018669
O -0.898838 -0.860681 1.533141
O -1.158453 1.93836 -0.988709
O 0.79674 0.018047 -1.546931
O 2.40611 -1.087576 0.21541
H -2.399842 0.49612 1.736113
H 3.162988 0.810975 0.622066
H -1.27886 2.230236 1.104177
H -3.410832 -1.110836 0.19403
H -2.950081 0.348626 -0.717704
H -1.504943 2.832137 -1.076826
H -2.252434 -1.624811 -1.770008
H 0.037313 -0.076598 1.903394
H 3.076821 -1.393471 0.831244
H 3.411824 0.212195 -1.043996
H 0.035896 0.465033 -1.940296
H 1.671229 1.861205 -1.141515
Na 0.338971 -1.891566 -0.270216

140. Transition States of 1st Gal-L3b C=O Migration

25

C -3.372814 0.027371 -0.128127
C -2.159724 -0.088894 0.788041
C -1.051386 0.830169 0.330244
O -1.39117 2.035838 -0.007189
C 0.348509 0.537756 0.142028
C 1.083916 -0.668538 0.803508
C 1.471275 -1.730394 -0.207055
O 2.277451 -1.096039 -1.2034
O 2.307018 -0.186589 1.346135
O 1.054927 1.550923 -0.243985
O -1.699383 -1.41411 0.851977
O -2.946929 -0.559104 -1.346313
H 0.466352 -1.13023 1.571542
H -3.65799 1.074237 -0.251147
H -0.365988 0.041492 -0.782566
H 2.039791 -2.512672 0.30146
H 0.570047 -2.158328 -0.652878
H -0.531513 2.497551 -0.191604
H 2.491719 -1.726456 -1.896367
H 2.142641 0.268911 2.177393
H -3.661352 -0.562603 -1.988767
H -4.199099 -0.533965 0.317415
H -2.007267 -1.86262 0.052643
H -2.433891 0.23989 1.796934
Na 3.128416 0.933875 -0.545791

141. Reactant of 1st Gal-L3b C=O Migration

25

C	-3.372814	0.027371	-0.128127
C	-2.159724	-0.088894	0.788041
C	-1.051386	0.830169	0.330244
O	-1.39117	2.035838	-0.007189
C	0.348509	0.537756	0.142028
C	1.083916	-0.668538	0.803508
C	1.471275	-1.730394	-0.207055
O	2.277451	-1.096039	-1.2034
O	2.307018	-0.186589	1.346135
O	1.054927	1.550923	-0.243985
O	-1.699383	-1.41411	0.851977
O	-2.946929	-0.559104	-1.346313
H	0.466352	-1.13023	1.571542
H	-3.65799	1.074237	-0.251147
H	-0.365988	0.041492	-0.782566
H	2.039791	-2.512672	0.30146
H	0.570047	-2.158328	-0.652878
H	-0.531513	2.497551	-0.191604
H	2.491719	-1.726456	-1.896367
H	2.142641	0.268911	2.177393
H	-3.661352	-0.562603	-1.988767
H	-4.199099	-0.533965	0.317415
H	-2.007267	-1.86262	0.052643
H	-2.433891	0.23989	1.796934
Na	3.128416	0.933875	-0.545791

142. Transition States of 2nd Gal-L3b C=O Migration

25

C -3.25722 0.286395 -0.47858
C -2.079679 -0.001093 0.451116
C -0.913343 0.896512 0.176703
O -1.177827 2.135751 -0.100661
C 0.484522 0.550307 0.09314
C 1.09415 -0.737433 0.730209
C 1.494997 -1.757339 -0.316653
O 2.405332 -1.106799 -1.210066
O 2.294039 -0.364737 1.395115
O 1.266564 1.550766 -0.150791
O -1.646735 -1.331869 0.315496
O -4.183003 -0.730289 -0.160574
H 0.389915 -1.202951 1.417994
H -2.924047 0.215072 -1.521254
H -0.149683 0.14559 -0.92561
H 1.985004 -2.599391 0.177784
H 0.603587 -2.100444 -0.846038
H -0.290032 2.574766 -0.167576
H 2.628151 -1.699907 -1.932596
H 2.089079 0.061834 2.232791
H -4.974125 -0.653357 -0.699896
H -3.652171 1.287041 -0.287574
H -2.443344 -1.880327 0.28542
H -2.397794 0.202878 1.485049
Na 3.320722 0.81841 -0.351114

143. Reactant of 2nd Gal-L3b C=O Migration

25

C	-3.25722	0.286395	-0.47858
C	-2.079679	-0.001093	0.451116
C	-0.913343	0.896512	0.176703
O	-1.177827	2.135751	-0.100661
C	0.484522	0.550307	0.09314
C	1.09415	-0.737433	0.730209
C	1.494997	-1.757339	-0.316653
O	2.405332	-1.106799	-1.210066
O	2.294039	-0.364737	1.395115
O	1.266564	1.550766	-0.150791
O	-1.646735	-1.331869	0.315496
O	-4.183003	-0.730289	-0.160574
H	0.389915	-1.202951	1.417994
H	-2.924047	0.215072	-1.521254
H	-0.149683	0.14559	-0.92561
H	1.985004	-2.599391	0.177784
H	0.603587	-2.100444	-0.846038
H	-0.290032	2.574766	-0.167576
H	2.628151	-1.699907	-1.932596
H	2.089079	0.061834	2.232791
H	-4.974125	-0.653357	-0.699896
H	-3.652171	1.287041	-0.287574
H	-2.443344	-1.880327	0.28542
H	-2.397794	0.202878	1.485049
Na	3.320722	0.81841	-0.351114

144. Transition States of 3rd Gal-L3b C=O Migration

25

C 3.419816 0.067445 0.064445
C 2.173226 -0.119024 -0.793352
C 1.057133 0.783861 -0.315736
O 1.385314 2.002374 -0.014055
C -0.33277 0.479126 -0.071465
C -1.056856 -0.747448 -0.672795
C -1.58992 -1.697571 0.389903
O -2.523334 -0.975653 1.19173
O -2.157054 -0.191624 -1.395645
O -1.036139 1.487368 0.32238
O 1.7632 -1.462095 -0.794819
O 3.063453 -0.487031 1.319503
H -0.388013 -1.297117 -1.33156
H 3.678257 1.125525 0.13922
H 0.436096 0.011634 0.8348
H -2.086098 -2.541632 -0.099389
H -0.761298 -2.074732 0.996885
H 0.522572 2.452206 0.186662
H -2.805955 -1.51997 1.931403
H -2.339418 -0.722683 -2.175211
H 3.802383 -0.444168 1.931994
H 4.242769 -0.485923 -0.396869
H 2.09344 -1.862505 0.021285
H 2.390864 0.18333 -1.823736
Na -3.161172 1.053728 0.285978

145. Reactant of 3rd Gal-L3b C=O Migration

25

C 3.419816 0.067445 0.064445
C 2.173226 -0.119024 -0.793352
C 1.057133 0.783861 -0.315736
O 1.385314 2.002374 -0.014055
C -0.33277 0.479126 -0.071465
C -1.056856 -0.747448 -0.672795
C -1.58992 -1.697571 0.389903
O -2.523334 -0.975653 1.19173
O -2.157054 -0.191624 -1.395645
O -1.036139 1.487368 0.32238
O 1.7632 -1.462095 -0.794819
O 3.063453 -0.487031 1.319503
H -0.388013 -1.297117 -1.33156
H 3.678257 1.125525 0.13922
H 0.436096 0.011634 0.8348
H -2.086098 -2.541632 -0.099389
H -0.761298 -2.074732 0.996885
H 0.522572 2.452206 0.186662
H -2.805955 -1.51997 1.931403
H -2.339418 -0.722683 -2.175211
H 3.802383 -0.444168 1.931994
H 4.242769 -0.485923 -0.396869
H 2.09344 -1.862505 0.021285
H 2.390864 0.18333 -1.823736
Na -3.161172 1.053728 0.285978

146. Transition States of 4th Gal-L3b C=O Migration

25

C 3.419786 -0.067296 -0.064465
C 2.173174 0.11921 0.793293
C 1.057139 -0.783842 0.315887
O 1.38534 -2.002331 0.014247
C -0.332824 -0.479237 0.071773
C -1.056934 0.747246 0.673109
C -1.589773 1.697564 -0.389551
O -2.523125 0.975897 -1.191682
O -2.156987 0.191018 1.395888
O -1.036092 -1.487617 -0.321925
O 1.763011 1.462242 0.794502
O 3.063409 0.486907 -1.319635
H -0.388185 1.296859 1.332034
H 3.678333 -1.125366 -0.13903
H 0.435692 -0.011683 -0.834636
H -2.085911 2.541686 0.099709
H -0.761 2.074735 -0.996318
H 0.522596 -2.452261 -0.186261
H -2.805931 1.520585 -1.931016
H -2.343013 0.725551 2.172235
H 3.802306 0.443823 -1.93215
H 4.24268 0.486232 0.396763
H 2.093315 1.862547 -0.021627
H 2.390837 -0.182938 1.823735
Na -3.160871 -1.053688 -0.286807

147. Reactant of 4th Gal-L3b C=O Migration

25

C 3.419786 -0.067296 -0.064465
C 2.173174 0.11921 0.793293
C 1.057139 -0.783842 0.315887
O 1.38534 -2.002331 0.014247
C -0.332824 -0.479237 0.071773
C -1.056934 0.747246 0.673109
C -1.589773 1.697564 -0.389551
O -2.523125 0.975897 -1.191682
O -2.156987 0.191018 1.395888
O -1.036092 -1.487617 -0.321925
O 1.763011 1.462242 0.794502
O 3.063409 0.486907 -1.319635
H -0.388185 1.296859 1.332034
H 3.678333 -1.125366 -0.13903
H 0.435692 -0.011683 -0.834636
H -2.085911 2.541686 0.099709
H -0.761 2.074735 -0.996318
H 0.522596 -2.452261 -0.186261
H -2.805931 1.520585 -1.931016
H -2.343013 0.725551 2.172235
H 3.802306 0.443823 -1.93215
H 4.24268 0.486232 0.396763
H 2.093315 1.862547 -0.021627
H 2.390837 -0.182938 1.823735
Na -3.160871 -1.053688 -0.286807

148. Transition States of 1st Iso-L1 C-C cleavage

46

O 0.23214 1.679184 0.011973
O 1.213904 0.675719 -2.132049
O 4.385726 0.777383 -0.489477
O 1.912846 -1.853751 -1.204113
O 1.313516 -0.108552 0.819777
O 4.704708 -1.11493 1.62143
C 1.510958 1.173181 0.22858
C 2.166849 1.097015 -1.163381
C 3.34602 0.121933 -1.17953
C 2.980566 -1.208859 -0.507702
C 2.439466 -0.984123 0.900314
C 3.464513 -0.495752 1.932914
H 0.675891 1.442603 -2.365524
H 4.99336 0.132319 -0.096293
H 2.211103 -2.123502 -2.081477
H 5.293292 -1.087387 2.384157
H 2.094229 1.815588 0.903232
H 2.555274 2.090379 -1.413701
H 3.6154 -0.058485 -2.230661
H 3.857015 -1.863784 -0.460163
H 2.018789 -1.936926 1.234476
H 3.564421 0.593382 1.890753
H 3.118836 -0.779973 2.931874
O -2.646167 -2.549936 -0.660846
O -5.322473 -0.480908 0.441585
O -1.626467 -1.090225 0.930353
O -2.175993 0.571193 -1.123972
O -2.120543 3.116379 -0.256277
C -3.723256 -1.823034 -0.679072
C -4.28803 -1.381863 0.508688
C -2.647393 -0.313864 1.025663
C -2.721135 0.915566 0.141411
C -1.998996 2.132186 0.753618
C -0.548416 1.921533 1.175668
H -1.742678 3.956954 0.028624
H -6.10226 -0.80887 0.906007
H -2.089195 -2.206319 0.165441
H -2.161612 1.38738 -1.645774
H -4.207224 -2.00851 1.394202
H -3.096264 -0.162369 2.017448
H -3.772458 1.209922 0.02891
H -2.561189 2.420459 1.654934
H -0.177161 2.825817 1.680391
H -0.476323 1.074597 1.867344
H -4.070771 -1.448349 -1.64268
Na -0.217901 -0.76008 -0.876509

149. Reactant of 1st Iso-L1 C-C cleavage

46

O 0.23214 1.679184 0.011973
O 1.213904 0.675719 -2.132049
O 4.385726 0.777383 -0.489477
O 1.912846 -1.853751 -1.204113
O 1.313516 -0.108552 0.819777
O 4.704708 -1.11493 1.62143
C 1.510958 1.173181 0.22858
C 2.166849 1.097015 -1.163381
C 3.34602 0.121933 -1.17953
C 2.980566 -1.208859 -0.507702
C 2.439466 -0.984123 0.900314
C 3.464513 -0.495752 1.932914
H 0.675891 1.442603 -2.365524
H 4.99336 0.132319 -0.096293
H 2.211103 -2.123502 -2.081477
H 5.293292 -1.087387 2.384157
H 2.094229 1.815588 0.903232
H 2.555274 2.090379 -1.413701
H 3.6154 -0.058485 -2.230661
H 3.857015 -1.863784 -0.460163
H 2.018789 -1.936926 1.234476
H 3.564421 0.593382 1.890753
H 3.118836 -0.779973 2.931874
O -2.646167 -2.549936 -0.660846
O -5.322473 -0.480908 0.441585
O -1.626467 -1.090225 0.930353
O -2.175993 0.571193 -1.123972
O -2.120543 3.116379 -0.256277
C -3.723256 -1.823034 -0.679072
C -4.28803 -1.381863 0.508688
C -2.647393 -0.313864 1.025663
C -2.721135 0.915566 0.141411
C -1.998996 2.132186 0.753618
C -0.548416 1.921533 1.175668
H -1.742678 3.956954 0.028624
H -6.10226 -0.80887 0.906007
H -2.089195 -2.206319 0.165441
H -2.161612 1.38738 -1.645774
H -4.207224 -2.00851 1.394202
H -3.096264 -0.162369 2.017448
H -3.772458 1.209922 0.02891
H -2.561189 2.420459 1.654934
H -0.177161 2.825817 1.680391
H -0.476323 1.074597 1.867344
H -4.070771 -1.448349 -1.64268
Na -0.217901 -0.76008 -0.876509

150. Transition States of 2nd Iso-L1 C-C cleavage

46

O 0.212102 1.637028 0.003882
O 1.216227 0.663951 -2.138995
O 4.387635 0.79063 -0.497479
O 1.925621 -1.854889 -1.195123
O 1.325126 -0.119563 0.828593
O 4.72979 -1.088294 1.617447
C 1.500364 1.161448 0.225309
C 2.162421 1.09054 -1.164863
C 3.349944 0.125679 -1.181042
C 2.995296 -1.205698 -0.505143
C 2.46039 -0.982021 0.905344
C 3.485323 -0.477814 1.929973
H 0.69632 1.434925 -2.397849
H 4.999284 0.150979 -0.101674
H 2.218242 -2.116613 -2.076833
H 5.322365 -1.047054 2.376447
H 2.068275 1.820677 0.896582
H 2.542437 2.087794 -1.411478
H 3.61689 -0.055621 -2.232709
H 3.874607 -1.857165 -0.462078
H 2.052858 -1.938495 1.245423
H 3.574852 0.61192 1.879728
H 3.146413 -0.758335 2.93231
O -2.649013 -2.548441 -0.66431
O -5.33144 -0.48721 0.440166
O -1.63716 -1.09392 0.934163
O -2.190452 0.576636 -1.119457
O -2.013898 3.156575 -0.236934
C -3.72746 -1.825105 -0.683638
C -4.294277 -1.387107 0.504768
C -2.658187 -0.317775 1.028863
C -2.730502 0.914718 0.148245
C -1.999392 2.137012 0.751923
C -0.553146 1.918515 1.170107
H -2.784881 3.725308 -0.128848
H -6.119291 -0.832631 0.877835
H -2.093794 -2.202468 0.165049
H -2.09881 1.411245 -1.604491
H -4.214622 -2.016344 1.388565
H -3.109812 -0.169789 2.020098
H -3.786348 1.200777 0.040734
H -2.548069 2.446201 1.651829
H -0.177227 2.836483 1.638791
H -0.484214 1.084075 1.877058
H -4.0739 -1.448862 -1.647189
Na -0.214774 -0.758307 -0.862

151. Reactant of 2nd Iso-L1 C-C cleavage

46

O 0.212102 1.637028 0.003882
O 1.216227 0.663951 -2.138995
O 4.387635 0.79063 -0.497479
O 1.925621 -1.854889 -1.195123
O 1.325126 -0.119563 0.828593
O 4.72979 -1.088294 1.617447
C 1.500364 1.161448 0.225309
C 2.162421 1.09054 -1.164863
C 3.349944 0.125679 -1.181042
C 2.995296 -1.205698 -0.505143
C 2.46039 -0.982021 0.905344
C 3.485323 -0.477814 1.929973
H 0.69632 1.434925 -2.397849
H 4.999284 0.150979 -0.101674
H 2.218242 -2.116613 -2.076833
H 5.322365 -1.047054 2.376447
H 2.068275 1.820677 0.896582
H 2.542437 2.087794 -1.411478
H 3.61689 -0.055621 -2.232709
H 3.874607 -1.857165 -0.462078
H 2.052858 -1.938495 1.245423
H 3.574852 0.61192 1.879728
H 3.146413 -0.758335 2.93231
O -2.649013 -2.548441 -0.66431
O -5.33144 -0.48721 0.440166
O -1.63716 -1.09392 0.934163
O -2.190452 0.576636 -1.119457
O -2.013898 3.156575 -0.236934
C -3.72746 -1.825105 -0.683638
C -4.294277 -1.387107 0.504768
C -2.658187 -0.317775 1.028863
C -2.730502 0.914718 0.148245
C -1.999392 2.137012 0.751923
C -0.553146 1.918515 1.170107
H -2.784881 3.725308 -0.128848
H -6.119291 -0.832631 0.877835
H -2.093794 -2.202468 0.165049
H -2.09881 1.411245 -1.604491
H -4.214622 -2.016344 1.388565
H -3.109812 -0.169789 2.020098
H -3.786348 1.200777 0.040734
H -2.548069 2.446201 1.651829
H -0.177227 2.836483 1.638791
H -0.484214 1.084075 1.877058
H -4.0739 -1.448862 -1.647189
Na -0.214774 -0.758307 -0.862

152. Transition States of 3rd Iso-L1 C-C cleavage

46

O -0.202508 -1.178366 0.692567
O 1.14036 0.58829 2.106269
O 4.203011 -0.97288 0.996626
O 2.550766 2.19241 0.180756
O 1.090259 -0.028026 -0.753447
O 3.380184 -1.813126 -1.478848
C 1.115445 -1.002685 0.29592
C 1.883095 -0.471031 1.508769
C 3.271854 0.064991 1.121929
C 3.209186 0.958749 -0.131517
C 2.358362 0.382796 -1.270079
C 3.001172 -0.640967 -2.192446
H 0.414607 0.197541 2.610515
H 4.058749 -1.454765 0.164766
H 3.059428 2.673698 0.845415
H 3.835397 -2.428849 -2.065917
H 1.556613 -1.935823 -0.077382
H 2.032239 -1.28792 2.222893
H 3.612613 0.683889 1.960209
H 4.230829 1.146394 -0.484421
H 2.121125 1.231886 -1.922134
H 2.270199 -0.89371 -2.968341
H 3.874783 -0.174036 -2.663935
O -1.527421 2.510229 -0.984523
O -1.814995 1.03737 1.226976
O -1.67007 0.262892 -1.675424
O -4.590144 -1.028081 -0.084833
O -2.567358 -1.478206 1.597799
C -2.719129 2.364146 -0.459721
C -2.917281 1.453989 0.554019
C -2.879061 0.136013 -1.288907
C -3.325819 -1.195847 -0.702497
C -2.438656 -1.989033 0.279877
C -0.944047 -2.128604 -0.055648
H -3.50768 -1.51155 1.82945
H -1.948664 0.115783 1.550138
H -1.407469 1.426506 -1.518435
H -5.277662 -1.522896 -0.543943
H -3.898525 1.260409 0.979825
H -3.689724 0.612591 -1.856414
H -3.415629 -1.823794 -1.604303
H -2.861902 -3.00241 0.263048
H -0.609422 -3.132067 0.23175
H -0.78427 -1.980652 -1.129219
H -3.569711 2.870003 -0.918942
Na 0.206971 1.872239 0.413258

153. Reactant of 3rd Iso-L1 C-C cleavage

46

O -0.202508 -1.178366 0.692567
O 1.14036 0.58829 2.106269
O 4.203011 -0.97288 0.996626
O 2.550766 2.19241 0.180756
O 1.090259 -0.028026 -0.753447
O 3.380184 -1.813126 -1.478848
C 1.115445 -1.002685 0.29592
C 1.883095 -0.471031 1.508769
C 3.271854 0.064991 1.121929
C 3.209186 0.958749 -0.131517
C 2.358362 0.382796 -1.270079
C 3.001172 -0.640967 -2.192446
H 0.414607 0.197541 2.610515
H 4.058749 -1.454765 0.164766
H 3.059428 2.673698 0.845415
H 3.835397 -2.428849 -2.065917
H 1.556613 -1.935823 -0.077382
H 2.032239 -1.28792 2.222893
H 3.612613 0.683889 1.960209
H 4.230829 1.146394 -0.484421
H 2.121125 1.231886 -1.922134
H 2.270199 -0.89371 -2.968341
H 3.874783 -0.174036 -2.663935
O -1.527421 2.510229 -0.984523
O -1.814995 1.03737 1.226976
O -1.67007 0.262892 -1.675424
O -4.590144 -1.028081 -0.084833
O -2.567358 -1.478206 1.597799
C -2.719129 2.364146 -0.459721
C -2.917281 1.453989 0.554019
C -2.879061 0.136013 -1.288907
C -3.325819 -1.195847 -0.702497
C -2.438656 -1.989033 0.279877
C -0.944047 -2.128604 -0.055648
H -3.50768 -1.51155 1.82945
H -1.948664 0.115783 1.550138
H -1.407469 1.426506 -1.518435
H -5.277662 -1.522896 -0.543943
H -3.898525 1.260409 0.979825
H -3.689724 0.612591 -1.856414
H -3.415629 -1.823794 -1.604303
H -2.861902 -3.00241 0.263048
H -0.609422 -3.132067 0.23175
H -0.78427 -1.980652 -1.129219
H -3.569711 2.870003 -0.918942
Na 0.206971 1.872239 0.413258

154. Transition States of 4th Iso-L1 C-C cleavage

46

O -0.200155 -1.178361 0.692132
O 1.139946 0.592955 2.104512
O 4.205239 -0.96615 1.000192
O 2.546995 2.194099 0.176182
O 1.092831 -0.030758 -0.755276
O 3.386139 -1.813357 -1.473679
C 1.11818 -1.002203 0.297072
C 1.883927 -0.466889 1.509514
C 3.272237 0.070361 1.122546
C 3.208893 0.961501 -0.132714
C 2.360801 0.381388 -1.27115
C 3.006555 -0.643297 -2.1904
H 0.415308 0.202964 2.610919
H 4.06285 -1.449768 0.16901
H 3.052486 2.677067 0.842051
H 3.842703 -2.429938 -2.058797
H 1.560947 -1.93591 -0.073008
H 2.033631 -1.282162 2.225345
H 3.61119 0.691599 1.95983
H 4.230498 1.150805 -0.484862
H 2.122866 1.228655 -1.9253
H 2.277042 -0.898872 -2.966741
H 3.880122 -0.176104 -2.661705
O -1.5358 2.512822 -0.982684
O -1.816842 1.03491 1.226799
O -1.672731 0.266011 -1.675323
O -4.589179 -1.033684 -0.084231
O -2.564273 -1.48134 1.597405
C -2.726279 2.362925 -0.456301
C -2.920887 1.450485 0.556146
C -2.881149 0.135736 -1.28766
C -3.324651 -1.197796 -0.70236
C -2.435575 -1.990391 0.278813
C -0.941053 -2.127141 -0.058583
H -3.504392 -1.516798 1.829606
H -1.949075 0.113196 1.550348
H -1.413899 1.430342 -1.518434
H -5.276003 -1.528219 -0.544671
H -3.901125 1.254135 0.982983
H -3.693386 0.610754 -1.854231
H -3.413058 -1.825012 -1.604817
H -2.857278 -3.004378 0.261156
H -0.604954 -3.131046 0.225575
H -0.782087 -1.97569 -1.131772
H -3.578788 2.867487 -0.913367
Na 0.201913 1.867422 0.407558

155. Reactant of 4th Iso-L1 C-C cleavage

46

O -0.200155 -1.178361 0.692132
O 1.139946 0.592955 2.104512
O 4.205239 -0.96615 1.000192
O 2.546995 2.194099 0.176182
O 1.092831 -0.030758 -0.755276
O 3.386139 -1.813357 -1.473679
C 1.11818 -1.002203 0.297072
C 1.883927 -0.466889 1.509514
C 3.272237 0.070361 1.122546
C 3.208893 0.961501 -0.132714
C 2.360801 0.381388 -1.27115
C 3.006555 -0.643297 -2.1904
H 0.415308 0.202964 2.610919
H 4.06285 -1.449768 0.16901
H 3.052486 2.677067 0.842051
H 3.842703 -2.429938 -2.058797
H 1.560947 -1.93591 -0.073008
H 2.033631 -1.282162 2.225345
H 3.61119 0.691599 1.95983
H 4.230498 1.150805 -0.484862
H 2.122866 1.228655 -1.9253
H 2.277042 -0.898872 -2.966741
H 3.880122 -0.176104 -2.661705
O -1.5358 2.512822 -0.982684
O -1.816842 1.03491 1.226799
O -1.672731 0.266011 -1.675323
O -4.589179 -1.033684 -0.084231
O -2.564273 -1.48134 1.597405
C -2.726279 2.362925 -0.456301
C -2.920887 1.450485 0.556146
C -2.881149 0.135736 -1.28766
C -3.324651 -1.197796 -0.70236
C -2.435575 -1.990391 0.278813
C -0.941053 -2.127141 -0.058583
H -3.504392 -1.516798 1.829606
H -1.949075 0.113196 1.550348
H -1.413899 1.430342 -1.518434
H -5.276003 -1.528219 -0.544671
H -3.901125 1.254135 0.982983
H -3.693386 0.610754 -1.854231
H -3.413058 -1.825012 -1.604817
H -2.857278 -3.004378 0.261156
H -0.604954 -3.131046 0.225575
H -0.782087 -1.97569 -1.131772
H -3.578788 2.867487 -0.913367
Na 0.201913 1.867422 0.407558

156. Transition States of 1st Iso-L1 C=O Migration

46

O	-0.483633	-1.829478	0.98069
O	-1.492631	-1.692634	-1.459403
O	-4.560673	-0.495056	0.11911
O	-2.109034	1.09706	-2.09056
O	-0.963165	0.327259	0.396863
O	-3.408046	1.022443	2.09368
C	-1.524671	-0.935302	0.778671
C	-2.383179	-1.459992	-0.367274
C	-3.469589	-0.433852	-0.75354
C	-2.892963	0.993824	-0.895895
C	-1.90721	1.386221	0.212157
C	-2.487976	1.934507	1.507232
H	-1.852274	-2.378683	-2.033114
H	-4.356861	-0.026222	0.945788
H	-2.681658	1.042996	-2.865365
H	-3.816015	1.411945	2.876716
H	-2.113127	-0.803909	1.695629
H	-2.873493	-2.387431	-0.054167
H	-3.851807	-0.726727	-1.740014
H	-3.728226	1.704687	-0.922823
H	-1.308033	2.20915	-0.194987
H	-1.654642	2.12848	2.192455
H	-2.984016	2.884723	1.274028
O	2.936248	2.906982	0.245926
O	1.953271	1.22732	-1.419068
O	4.58882	-0.738358	0.092633
O	1.304695	-1.654529	-1.03055
O	2.666747	-0.333236	2.134229
C	3.524624	1.756018	0.198276
C	3.030054	0.846007	-0.799092
C	3.40584	-0.633517	-0.679631
C	2.2603	-1.416666	-0.013687
C	1.667608	-0.661544	1.18734
C	0.543061	-1.429954	1.894478
H	3.394335	-0.969444	2.101691
H	2.169899	2.818651	-0.381067
H	5.252168	-1.261269	-0.370593
H	0.536309	-2.11143	-0.647192
H	4.088477	1.378773	-1.202244
H	3.532059	-1.045284	-1.68618
H	2.692007	-2.370273	0.334324
H	1.256287	0.284048	0.824706
H	0.934962	-2.353447	2.331961
H	0.130191	-0.809755	2.698748
H	4.381129	1.579017	0.839028
Na	0.072376	0.129521	-1.830903

157. Reactant of 1st Iso-L1 C=O Migration

46

```
O   -0.483633   -1.829478   0.98069
O   -1.492631   -1.692634   -1.459403
O   -4.560673   -0.495056   0.11911
O   -2.109034   1.09706   -2.09056
O   -0.963165   0.327259   0.396863
O   -3.408046   1.022443   2.09368
C   -1.524671   -0.935302   0.778671
C   -2.383179   -1.459992   -0.367274
C   -3.469589   -0.433852   -0.75354
C   -2.892963   0.993824   -0.895895
C   -1.90721   1.386221   0.212157
C   -2.487976   1.934507   1.507232
H   -1.852274   -2.378683   -2.033114
H   -4.356861   -0.026222   0.945788
H   -2.681658   1.042996   -2.865365
H   -3.816015   1.411945   2.876716
H   -2.113127   -0.803909   1.695629
H   -2.873493   -2.387431   -0.054167
H   -3.851807   -0.726727   -1.740014
H   -3.728226   1.704687   -0.922823
H   -1.308033   2.20915   -0.194987
H   -1.654642   2.12848   2.192455
H   -2.984016   2.884723   1.274028
O   2.936248   2.906982   0.245926
O   1.953271   1.22732   -1.419068
O   4.58882   -0.738358   0.092633
O   1.304695   -1.654529   -1.03055
O   2.666747   -0.333236   2.134229
C   3.524624   1.756018   0.198276
C   3.030054   0.846007   -0.799092
C   3.40584   -0.633517   -0.679631
C   2.2603   -1.416666   -0.013687
C   1.667608   -0.661544   1.18734
C   0.543061   -1.429954   1.894478
H   3.394335   -0.969444   2.101691
H   2.169899   2.818651   -0.381067
H   5.252168   -1.261269   -0.370593
H   0.536309   -2.11143   -0.647192
H   4.088477   1.378773   -1.202244
H   3.532059   -1.045284   -1.68618
H   2.692007   -2.370273   0.334324
H   1.256287   0.284048   0.824706
H   0.934962   -2.353447   2.331961
H   0.130191   -0.809755   2.698748
H   4.381129   1.579017   0.839028
Na   0.072376   0.129521   -1.830903
```

158. Transition States of 2nd Iso-L1 C=O Migration

46

O   -0.506127   -2.002155   0.609681
O   -1.512859   -1.382271   -1.75573
O   -4.555857   -0.466169   0.04544
O   -2.095197   1.484005   -1.84375
O   -0.949833   0.233769   0.460174
O   -3.367679   0.638701   2.266349
C   -1.533134   -1.06811   0.587056
C   -2.397904   -1.351506   -0.636359
C   -3.470353   -0.255815   -0.811182
C   -2.871809   1.164015   -0.684327
C   -1.875483   1.325793   0.471506
C   -2.448375   1.638626   1.845825
H   -1.88167   -1.942092   -2.448532
H   -4.339745   -0.164757   0.943591
H   -2.672777   1.583552   -2.610448
H   -3.791831   0.896912   3.093924
H   -2.121202   -1.111059   1.513087
H   -2.900762   -2.314948   -0.50384
H   -3.864199   -0.350249   -1.831304
H   -3.696296   1.878321   -0.566734
H   -1.260495   2.197649   0.219079
H   -1.612285   1.70694   2.551659
H   -2.941488   2.61668   1.787097
O   2.99136   2.872973   0.658034
O   1.901014   1.472099   -1.096756
O   4.615845   -0.760324   -0.121801
O   1.28324   -1.412646   -1.319599
O   2.756659   -0.976848   1.967317
C   3.595785   1.75674   0.42753
C   3.02549   0.979432   -0.643575
C   3.396824   -0.508584   -0.771522
C   2.245737   -1.4053   -0.282633
C   1.661675   -0.926555   1.055423
C   0.522447   -1.814312   1.577983
H   2.500537   -0.643559   2.835758
H   2.188229   2.812781   0.053879
H   4.434092   -0.987937   0.802044
H   0.53192   -1.971086   -1.061012
H   4.035126   1.536493   -1.077161
H   3.531695   -0.73772   -1.832477
H   2.668938   -2.411376   -0.140362
H   1.301076   0.100925   0.93693
H   0.917226   -2.809834   1.80116
H   0.102814   -1.388052   2.500636
H   4.519055   1.541468   0.955522
Na   0.069282   0.467756   -1.790155

159. Reactant of 2nd Iso-L1 C=O Migration

46

O -0.506127 -2.002155 0.609681
O -1.512859 -1.382271 -1.75573
O -4.555857 -0.466169 0.04544
O -2.095197 1.484005 -1.84375
O -0.949833 0.233769 0.460174
O -3.367679 0.638701 2.266349
C -1.533134 -1.06811 0.587056
C -2.397904 -1.351506 -0.636359
C -3.470353 -0.255815 -0.811182
C -2.871809 1.164015 -0.684327
C -1.875483 1.325793 0.471506
C -2.448375 1.638626 1.845825
H -1.88167 -1.942092 -2.448532
H -4.339745 -0.164757 0.943591
H -2.672777 1.583552 -2.610448
H -3.791831 0.896912 3.093924
H -2.121202 -1.111059 1.513087
H -2.900762 -2.314948 -0.50384
H -3.864199 -0.350249 -1.831304
H -3.696296 1.878321 -0.566734
H -1.260495 2.197649 0.219079
H -1.612285 1.70694 2.551659
H -2.941488 2.61668 1.787097
O 2.99136 2.872973 0.658034
O 1.901014 1.472099 -1.096756
O 4.615845 -0.760324 -0.121801
O 1.28324 -1.412646 -1.319599
O 2.756659 -0.976848 1.967317
C 3.595785 1.75674 0.42753
C 3.02549 0.979432 -0.643575
C 3.396824 -0.508584 -0.771522
C 2.245737 -1.4053 -0.282633
C 1.661675 -0.926555 1.055423
C 0.522447 -1.814312 1.577983
H 2.500537 -0.643559 2.835758
H 2.188229 2.812781 0.053879
H 4.434092 -0.987937 0.802044
H 0.53192 -1.971086 -1.061012
H 4.035126 1.536493 -1.077161
H 3.531695 -0.73772 -1.832477
H 2.668938 -2.411376 -0.140362
H 1.301076 0.100925 0.93693
H 0.917226 -2.809834 1.80116
H 0.102814 -1.388052 2.500636
H 4.519055 1.541468 0.955522
Na 0.069282 0.467756 -1.790155

160. Transition States of 3rd Iso-L1 C=O Migration

46

O -0.347078 -1.845887 1.161617
O -1.406646 -1.936789 -1.257577
O -4.304939 -0.741727 0.536689
O -2.21227 0.634191 -2.126217
O -0.973387 0.231327 0.443267
O -3.609422 2.661251 1.038606
C -1.442333 -1.030987 0.918266
C -2.286281 -1.692074 -0.167632
C -3.433477 -0.754115 -0.574276
C -2.937648 0.669006 -0.888747
C -1.998549 1.199221 0.196842
C -2.686918 1.68008 1.475222
H -1.835407 -2.512489 -1.901848
H -5.146388 -0.329125 0.307095
H -2.507106 1.355165 -2.693839
H -3.992742 3.118109 1.795289
H -2.031724 -0.885588 1.833682
H -2.705434 -2.626181 0.221838
H -3.920384 -1.160686 -1.471796
H -3.800558 1.338046 -0.968284
H -1.472403 2.063001 -0.224382
H -3.202536 0.867261 1.994887
H -1.917767 2.103549 2.133405
O 2.775725 3.011831 0.016438
O 1.863151 1.159013 -1.509137
O 4.637287 -0.534663 0.081416
O 1.395978 -1.724572 -0.89811
O 2.728942 -0.0857 2.127424
C 3.427844 1.894813 0.034278
C 2.970156 0.888532 -0.884835
C 3.435648 -0.554349 -0.668526
C 2.351331 -1.352477 0.078269
C 1.734214 -0.544294 1.231425
C 0.671362 -1.32064 2.019516
H 3.488306 -0.684472 2.134587
H 2.000375 2.839596 -0.5797
H 5.321397 -1.050178 -0.359331
H 0.673021 -2.215669 -0.472117
H 3.988836 1.452859 -1.346121
H 3.566543 -1.030106 -1.645845
H 2.846916 -2.248888 0.487438
H 1.259652 0.345107 0.80864
H 1.124611 -2.18515 2.514578
H 0.238175 -0.666123 2.784901
H 4.304645 1.811858 0.666501
Na 0.046287 -0.074761 -1.777612

161. Reactant of 3rd Iso-L1 C=O Migration

46

O -0.347078 -1.845887 1.161617
O -1.406646 -1.936789 -1.257577
O -4.304939 -0.741727 0.536689
O -2.21227 0.634191 -2.126217
O -0.973387 0.231327 0.443267
O -3.609422 2.661251 1.038606
C -1.442333 -1.030987 0.918266
C -2.286281 -1.692074 -0.167632
C -3.433477 -0.754115 -0.574276
C -2.937648 0.669006 -0.888747
C -1.998549 1.199221 0.196842
C -2.686918 1.68008 1.475222
H -1.835407 -2.512489 -1.901848
H -5.146388 -0.329125 0.307095
H -2.507106 1.355165 -2.693839
H -3.992742 3.118109 1.795289
H -2.031724 -0.885588 1.833682
H -2.705434 -2.626181 0.221838
H -3.920384 -1.160686 -1.471796
H -3.800558 1.338046 -0.968284
H -1.472403 2.063001 -0.224382
H -3.202536 0.867261 1.994887
H -1.917767 2.103549 2.133405
O 2.775725 3.011831 0.016438
O 1.863151 1.159013 -1.509137
O 4.637287 -0.534663 0.081416
O 1.395978 -1.724572 -0.89811
O 2.728942 -0.0857 2.127424
C 3.427844 1.894813 0.034278
C 2.970156 0.888532 -0.884835
C 3.435648 -0.554349 -0.668526
C 2.351331 -1.352477 0.078269
C 1.734214 -0.544294 1.231425
C 0.671362 -1.32064 2.019516
H 3.488306 -0.684472 2.134587
H 2.000375 2.839596 -0.5797
H 5.321397 -1.050178 -0.359331
H 0.673021 -2.215669 -0.472117
H 3.988836 1.452859 -1.346121
H 3.566543 -1.030106 -1.645845
H 2.846916 -2.248888 0.487438
H 1.259652 0.345107 0.80864
H 1.124611 -2.18515 2.514578
H 0.238175 -0.666123 2.784901
H 4.304645 1.811858 0.666501
Na 0.046287 -0.074761 -1.777612

162. Transition States of 4th Iso-L1 C=O Migration

46

O -0.34036 -1.841351 1.088605
O -1.389024 -1.886641 -1.322332
O -4.281212 -0.698611 0.492576
O -2.124878 0.699536 -2.116594
O -0.991253 0.262843 0.472075
O -3.998975 2.215616 1.083197
C -1.446885 -1.032553 0.877444
C -2.279081 -1.658472 -0.237586
C -3.420816 -0.704794 -0.624629
C -2.909325 0.713547 -0.917473
C -2.023465 1.224756 0.220975
C -2.775183 1.628504 1.499579
H -1.829359 -2.411096 -2.001789
H -4.867514 0.071287 0.469597
H -2.460921 1.358787 -2.733397
H -4.385889 2.723402 1.805436
H -2.042531 -0.946595 1.793756
H -2.705942 -2.599518 0.127463
H -3.923772 -1.090934 -1.523732
H -3.764157 1.385053 -1.036568
H -1.492097 2.103312 -0.15918
H -2.971513 0.762313 2.136628
H -2.158559 2.341979 2.056482
O 2.81687 3.019496 0.09482
O 1.914371 1.200357 -1.472928
O 4.66088 -0.534831 0.118873
O 1.432315 -1.697936 -0.932509
O 2.719673 -0.110899 2.140691
C 3.465159 1.899954 0.100239
C 3.013443 0.912814 -0.842049
C 3.470299 -0.535847 -0.648772
C 2.372335 -1.344771 0.065852
C 1.738203 -0.556201 1.22385
C 0.664532 -1.347675 1.979592
H 3.478897 -0.709863 2.149876
H 2.048797 2.859166 -0.514379
H 5.347028 -1.051348 -0.317578
H 0.702528 -2.19712 -0.527559
H 4.039599 1.481832 -1.281254
H 3.6141 -0.992214 -1.633479
H 2.859526 -2.249272 0.467037
H 1.2669 0.339644 0.80979
H 1.107096 -2.227528 2.457302
H 0.223114 -0.711942 2.755693
H 4.334337 1.802062 0.740749
Na 0.092525 -0.023419 -1.763165

163. Reactant of 4th Iso-L1 C=O Migration

46

O -0.34036 -1.841351 1.088605
O -1.389024 -1.886641 -1.322332
O -4.281212 -0.698611 0.492576
O -2.124878 0.699536 -2.116594
O -0.991253 0.262843 0.472075
O -3.998975 2.215616 1.083197
C -1.446885 -1.032553 0.877444
C -2.279081 -1.658472 -0.237586
C -3.420816 -0.704794 -0.624629
C -2.909325 0.713547 -0.917473
C -2.023465 1.224756 0.220975
C -2.775183 1.628504 1.499579
H -1.829359 -2.411096 -2.001789
H -4.867514 0.071287 0.469597
H -2.460921 1.358787 -2.733397
H -4.385889 2.723402 1.805436
H -2.042531 -0.946595 1.793756
H -2.705942 -2.599518 0.127463
H -3.923772 -1.090934 -1.523732
H -3.764157 1.385053 -1.036568
H -1.492097 2.103312 -0.15918
H -2.971513 0.762313 2.136628
H -2.158559 2.341979 2.056482
O 2.81687 3.019496 0.09482
O 1.914371 1.200357 -1.472928
O 4.66088 -0.534831 0.118873
O 1.432315 -1.697936 -0.932509
O 2.719673 -0.110899 2.140691
C 3.465159 1.899954 0.100239
C 3.013443 0.912814 -0.842049
C 3.470299 -0.535847 -0.648772
C 2.372335 -1.344771 0.065852
C 1.738203 -0.556201 1.22385
C 0.664532 -1.347675 1.979592
H 3.478897 -0.709863 2.149876
H 2.048797 2.859166 -0.514379
H 5.347028 -1.051348 -0.317578
H 0.702528 -2.19712 -0.527559
H 4.039599 1.481832 -1.281254
H 3.6141 -0.992214 -1.633479
H 2.859526 -2.249272 0.467037
H 1.2669 0.339644 0.80979
H 1.107096 -2.227528 2.457302
H 0.223114 -0.711942 2.755693
H 4.334337 1.802062 0.740749
Na 0.092525 -0.023419 -1.763165

164. Transition States of 1st Iso-L2 C-C cleavage

46

O -0.425513 -0.745514 -0.623289
O 0.544088 -0.137389 1.829416
O 3.488992 -1.811568 0.667609
O 2.603187 1.713433 1.239764
O 1.420036 0.396722 -0.944836
O 4.96635 -0.286022 -1.305271
C 0.963003 -0.850205 -0.442805
C 1.325653 -1.013765 1.043294
C 2.814302 -0.725491 1.264326
C 3.245942 0.597947 0.618052
C 2.828435 0.655662 -0.848241
C 3.634906 -0.247295 -1.794462
H -0.350913 -0.51846 1.902646
H 4.396595 -1.56478 0.435207
H 2.951415 1.83292 2.131958
H 5.567231 -0.585923 -1.996804
H 1.365754 -1.684566 -1.033609
H 1.162485 -2.056574 1.341689
H 2.994586 -0.682379 2.348366
H 4.33293 0.711675 0.681078
H 2.934449 1.695815 -1.169276
H 3.211986 -1.255873 -1.820292
H 3.593482 0.177265 -2.80263
O -0.747369 2.091258 -1.289635
O -1.947643 1.608928 1.075821
O -4.616605 0.587227 -1.014981
O -2.218598 -0.651569 1.721093
O -3.344458 -2.887916 -0.479639
C -2.123856 1.804237 -1.346885
C -2.686975 1.439865 0.00711
C -3.925668 0.84056 0.138882
C -3.051955 -0.952855 0.792377
C -2.615665 -1.678361 -0.487644
C -1.122798 -1.978827 -0.491899
H -3.478636 -3.212274 -1.377493
H -0.247426 1.321272 -1.601344
H -5.544867 0.39949 -0.835382
H -2.0245 0.654979 1.608315
H -2.655994 2.69833 -1.695223
H -4.475394 0.941873 1.069257
H -4.061727 -1.2598 1.092491
H -2.878184 -1.073927 -1.366331
H -0.863167 -2.490131 0.443001
H -0.88019 -2.640402 -1.333728
H -2.3494 1.008569 -2.065796
Na 0.346163 1.928486 0.756623

165. Reactant of 1st Iso-L2 C-C cleavage

46

O -0.425513 -0.745514 -0.623289
O 0.544088 -0.137389 1.829416
O 3.488992 -1.811568 0.667609
O 2.603187 1.713433 1.239764
O 1.420036 0.396722 -0.944836
O 4.96635 -0.286022 -1.305271
C 0.963003 -0.850205 -0.442805
C 1.325653 -1.013765 1.043294
C 2.814302 -0.725491 1.264326
C 3.245942 0.597947 0.618052
C 2.828435 0.655662 -0.848241
C 3.634906 -0.247295 -1.794462
H -0.350913 -0.51846 1.902646
H 4.396595 -1.56478 0.435207
H 2.951415 1.83292 2.131958
H 5.567231 -0.585923 -1.996804
H 1.365754 -1.684566 -1.033609
H 1.162485 -2.056574 1.341689
H 2.994586 -0.682379 2.348366
H 4.33293 0.711675 0.681078
H 2.934449 1.695815 -1.169276
H 3.211986 -1.255873 -1.820292
H 3.593482 0.177265 -2.80263
O -0.747369 2.091258 -1.289635
O -1.947643 1.608928 1.075821
O -4.616605 0.587227 -1.014981
O -2.218598 -0.651569 1.721093
O -3.344458 -2.887916 -0.479639
C -2.123856 1.804237 -1.346885
C -2.686975 1.439865 0.00711
C -3.925668 0.84056 0.138882
C -3.051955 -0.952855 0.792377
C -2.615665 -1.678361 -0.487644
C -1.122798 -1.978827 -0.491899
H -3.478636 -3.212274 -1.377493
H -0.247426 1.321272 -1.601344
H -5.544867 0.39949 -0.835382
H -2.0245 0.654979 1.608315
H -2.655994 2.69833 -1.695223
H -4.475394 0.941873 1.069257
H -4.061727 -1.2598 1.092491
H -2.878184 -1.073927 -1.366331
H -0.863167 -2.490131 0.443001
H -0.88019 -2.640402 -1.333728
H -2.3494 1.008569 -2.065796
Na 0.346163 1.928486 0.756623

166. Transition States of 2nd Iso-L2 C-C cleavage

46

O 0.325627 1.778587 -0.93919
O 0.991792 -0.523318 -2.011671
O 4.393092 0.24415 -1.035831
O 2.109926 -2.364028 0.09385
O 1.065918 0.299881 0.655985
O 3.844446 1.394692 1.419815
C 1.456642 1.179185 -0.411644
C 2.029964 0.332736 -1.54419
C 3.216625 -0.515157 -1.061499
C 2.940836 -1.210623 0.291907
C 2.161315 -0.355069 1.304068
C 2.993625 0.55924 2.193027
H 0.270621 0.050951 -2.326087
H 4.406818 0.80768 -0.245585
H 2.584892 -3.016109 -0.436808
H 4.42136 1.91296 1.993916
H 2.163885 1.922483 -0.022725
H 2.389408 0.982916 -2.348689
H 3.373272 -1.300938 -1.80995
H 3.90177 -1.512486 0.726384
H 1.681294 -1.058557 1.994586
H 2.304962 1.163321 2.795503
H 3.58703 -0.076077 2.862136
O -1.435614 -1.911166 1.50836
O -2.331089 -1.671656 -0.941709
O -4.755018 0.370296 0.675363
O -1.894734 0.411328 -1.909919
O -2.568279 2.187029 1.165955
C -2.786201 -1.47973 1.425482
C -3.095892 -1.164371 -0.019254
C -4.048236 -0.216759 -0.354714
C -2.486815 1.092884 -0.995814
C -1.744766 1.478175 0.278247
C -0.576333 2.417172 -0.056723
H -3.409276 1.725671 1.295424
H -1.264786 -2.252386 2.393496
H -5.643618 0.619082 0.394234
H -2.067711 -0.76944 -1.62257
H -3.475503 -2.269327 1.752813
H -4.483604 -0.207351 -1.34875
H -3.261868 1.808294 -1.305541
H -1.307706 0.579569 0.738862
H -0.948971 3.315004 -0.557344
H -0.093933 2.709683 0.883044
H -2.959566 -0.596751 2.051116
Na -0.045624 -1.724606 -0.327173

167. Reactant of 2nd Iso-L2 C-C cleavage

46

O 0.325627 1.778587 -0.93919
O 0.991792 -0.523318 -2.011671
O 4.393092 0.24415 -1.035831
O 2.109926 -2.364028 0.09385
O 1.065918 0.299881 0.655985
O 3.844446 1.394692 1.419815
C 1.456642 1.179185 -0.411644
C 2.029964 0.332736 -1.54419
C 3.216625 -0.515157 -1.061499
C 2.940836 -1.210623 0.291907
C 2.161315 -0.355069 1.304068
C 2.993625 0.55924 2.193027
H 0.270621 0.050951 -2.326087
H 4.406818 0.80768 -0.245585
H 2.584892 -3.016109 -0.436808
H 4.42136 1.91296 1.993916
H 2.163885 1.922483 -0.022725
H 2.389408 0.982916 -2.348689
H 3.373272 -1.300938 -1.80995
H 3.90177 -1.512486 0.726384
H 1.681294 -1.058557 1.994586
H 2.304962 1.163321 2.795503
H 3.58703 -0.076077 2.862136
O -1.435614 -1.911166 1.50836
O -2.331089 -1.671656 -0.941709
O -4.755018 0.370296 0.675363
O -1.894734 0.411328 -1.909919
O -2.568279 2.187029 1.165955
C -2.786201 -1.47973 1.425482
C -3.095892 -1.164371 -0.019254
C -4.048236 -0.216759 -0.354714
C -2.486815 1.092884 -0.995814
C -1.744766 1.478175 0.278247
C -0.576333 2.417172 -0.056723
H -3.409276 1.725671 1.295424
H -1.264786 -2.252386 2.393496
H -5.643618 0.619082 0.394234
H -2.067711 -0.76944 -1.62257
H -3.475503 -2.269327 1.752813
H -4.483604 -0.207351 -1.34875
H -3.261868 1.808294 -1.305541
H -1.307706 0.579569 0.738862
H -0.948971 3.315004 -0.557344
H -0.093933 2.709683 0.883044
H -2.959566 -0.596751 2.051116
Na -0.045624 -1.724606 -0.327173

168. Transition States of 3rd Iso-L2 C-C cleavage

46

O -0.506412 -1.484334 -1.514835
O -1.337812 0.981991 -2.029028
O -4.433628 -0.156296 -0.510784
O -1.975712 2.275406 0.556598
O -1.019613 -0.381371 0.410218
O -1.901946 -2.088469 2.288263
C -1.557169 -1.022868 -0.739703
C -2.282582 0.023748 -1.582801
C -3.356013 0.73015 -0.739875
C -2.851867 1.151499 0.655505
C -2.021136 0.058007 1.330312
C -2.836855 -1.094562 1.913729
H -0.650932 0.503182 -2.524198
H -5.019234 -0.186255 -1.276412
H -2.469714 3.057538 0.281718
H -2.356483 -2.784549 2.775813
H -2.213723 -1.847259 -0.427488
H -2.769721 -0.466043 -2.43619
H -3.679729 1.631458 -1.278192
H -3.725035 1.396063 1.272129
H -1.451726 0.514219 2.146282
H -3.383308 -0.69979 2.780363
H -3.573012 -1.479665 1.201511
O 1.28936 1.600645 1.699613
O 2.589323 2.075052 -0.604754
O 4.44713 -0.63557 0.73703
O 1.589766 0.316199 -1.729276
O 2.572711 -2.531406 0.17223
C 2.705916 1.421635 1.695298
C 3.124304 1.266682 0.257917
C 3.878243 0.18587 -0.197778
C 2.272646 -0.654392 -1.210498
C 1.593354 -1.572576 -0.206204
C 0.426612 -2.331784 -0.873492
H 2.229949 -3.101579 0.871813
H 1.001037 1.866412 2.580134
H 4.186018 -1.555349 0.565841
H 2.207767 1.40556 -1.408146
H 3.213385 2.304194 2.1047
H 4.424595 0.325151 -1.13113
H 2.974518 -1.187149 -1.866774
H 1.228189 -1.001246 0.655053
H 0.840111 -2.985814 -1.646048
H -0.073164 -2.945871 -0.110786
H 3.00303 0.537294 2.263417
Na 0.089012 1.632388 -0.273643

169. Reactant of 3rd Iso-L2 C-C cleavage

46

O -0.506412 -1.484334 -1.514835
O -1.337812 0.981991 -2.029028
O -4.433628 -0.156296 -0.510784
O -1.975712 2.275406 0.556598
O -1.019613 -0.381371 0.410218
O -1.901946 -2.088469 2.288263
C -1.557169 -1.022868 -0.739703
C -2.282582 0.023748 -1.582801
C -3.356013 0.73015 -0.739875
C -2.851867 1.151499 0.655505
C -2.021136 0.058007 1.330312
C -2.836855 -1.094562 1.913729
H -0.650932 0.503182 -2.524198
H -5.019234 -0.186255 -1.276412
H -2.469714 3.057538 0.281718
H -2.356483 -2.784549 2.775813
H -2.213723 -1.847259 -0.427488
H -2.769721 -0.466043 -2.43619
H -3.679729 1.631458 -1.278192
H -3.725035 1.396063 1.272129
H -1.451726 0.514219 2.146282
H -3.383308 -0.69979 2.780363
H -3.573012 -1.479665 1.201511
O 1.28936 1.600645 1.699613
O 2.589323 2.075052 -0.604754
O 4.44713 -0.63557 0.73703
O 1.589766 0.316199 -1.729276
O 2.572711 -2.531406 0.17223
C 2.705916 1.421635 1.695298
C 3.124304 1.266682 0.257917
C 3.878243 0.18587 -0.197778
C 2.272646 -0.654392 -1.210498
C 1.593354 -1.572576 -0.206204
C 0.426612 -2.331784 -0.873492
H 2.229949 -3.101579 0.871813
H 1.001037 1.866412 2.580134
H 4.186018 -1.555349 0.565841
H 2.207767 1.40556 -1.408146
H 3.213385 2.304194 2.1047
H 4.424595 0.325151 -1.13113
H 2.974518 -1.187149 -1.866774
H 1.228189 -1.001246 0.655053
H 0.840111 -2.985814 -1.646048
H -0.073164 -2.945871 -0.110786
H 3.00303 0.537294 2.263417
Na 0.089012 1.632388 -0.273643

170. Transition States of 4th Iso-L2 C-C cleavage

46

O 0.260406 2.118639 -0.419913
O 1.112175 0.408825 -2.230508
O 4.254429 0.902027 -0.582787
O 2.034693 -2.008855 -0.884743
O 1.015032 0.219864 0.598354
O 3.845253 -0.984928 2.518063
C 1.403657 1.416816 -0.079995
C 2.071499 1.050658 -1.404801
C 3.247639 0.099798 -1.168458
C 2.891301 -1.06859 -0.228161
C 2.113697 -0.604073 1.002826
C 2.968748 0.041062 2.093666
H 0.384947 1.039569 -2.365473
H 5.114819 0.471826 -0.649569
H 2.520208 -2.476237 -1.575054
H 4.414894 -0.662935 3.225091
H 2.061968 2.011465 0.569223
H 2.457006 1.95837 -1.88204
H 3.559853 -0.29728 -2.144514
H 3.808765 -1.563676 0.111573
H 1.654624 -1.49359 1.447009
H 3.52846 0.902829 1.717736
H 2.29473 0.364412 2.898002
O -1.227378 -2.186669 0.733917
O -2.559508 -1.487059 -1.471288
O -4.578233 0.02508 1.033682
O -1.811442 0.695498 -1.574049
O -2.62098 1.911696 1.735343
C -2.641809 -2.086693 0.854526
C -3.136937 -1.29225 -0.328909
C -4.020267 -0.225751 -0.202203
C -2.479281 1.170301 -0.569315
C -1.766565 1.36548 0.764641
C -0.654374 2.421709 0.615842
H -3.342232 1.295961 1.921259
H -0.889797 -2.818765 1.379007
H -5.488282 0.333725 0.946939
H -2.248323 -0.42385 -1.810284
H -3.111095 -3.078241 0.813818
H -4.576439 0.092108 -1.079389
H -3.250107 1.924657 -0.778489
H -1.311077 0.417558 1.084385
H -1.11455 3.38161 0.367001
H -0.149735 2.515216 1.584759
H -2.931311 -1.596832 1.788795
Na -0.106066 -1.176513 -1.028758

171. Reactant of 4th Iso-L2 C-C cleavage

46

O 0.260406 2.118639 -0.419913
O 1.112175 0.408825 -2.230508
O 4.254429 0.902027 -0.582787
O 2.034693 -2.008855 -0.884743
O 1.015032 0.219864 0.598354
O 3.845253 -0.984928 2.518063
C 1.403657 1.416816 -0.079995
C 2.071499 1.050658 -1.404801
C 3.247639 0.099798 -1.168458
C 2.891301 -1.06859 -0.228161
C 2.113697 -0.604073 1.002826
C 2.968748 0.041062 2.093666
H 0.384947 1.039569 -2.365473
H 5.114819 0.471826 -0.649569
H 2.520208 -2.476237 -1.575054
H 4.414894 -0.662935 3.225091
H 2.061968 2.011465 0.569223
H 2.457006 1.95837 -1.88204
H 3.559853 -0.29728 -2.144514
H 3.808765 -1.563676 0.111573
H 1.654624 -1.49359 1.447009
H 3.52846 0.902829 1.717736
H 2.29473 0.364412 2.898002
O -1.227378 -2.186669 0.733917
O -2.559508 -1.487059 -1.471288
O -4.578233 0.02508 1.033682
O -1.811442 0.695498 -1.574049
O -2.62098 1.911696 1.735343
C -2.641809 -2.086693 0.854526
C -3.136937 -1.29225 -0.328909
C -4.020267 -0.225751 -0.202203
C -2.479281 1.170301 -0.569315
C -1.766565 1.36548 0.764641
C -0.654374 2.421709 0.615842
H -3.342232 1.295961 1.921259
H -0.889797 -2.818765 1.379007
H -5.488282 0.333725 0.946939
H -2.248323 -0.42385 -1.810284
H -3.111095 -3.078241 0.813818
H -4.576439 0.092108 -1.079389
H -3.250107 1.924657 -0.778489
H -1.311077 0.417558 1.084385
H -1.11455 3.38161 0.367001
H -0.149735 2.515216 1.584759
H -2.931311 -1.596832 1.788795
Na -0.106066 -1.176513 -1.028758

172. Transition States of 1st Iso-L2 C=O Migration

46

O -0.481397 0.646388 -0.759486
O 0.843509 -1.751253 -1.067196
O 3.597423 0.449297 -1.928842
O 3.13152 -1.758859 0.895624
O 1.225169 0.385962 0.724577
O 2.12641 2.845677 1.198076
C 0.90052 0.620633 -0.640736
C 1.447612 -0.537079 -1.469109
C 2.971492 -0.631764 -1.264396
C 3.3987 -0.528369 0.216017
C 2.624486 0.548927 0.982019
C 3.120023 1.971379 0.70411
H -0.121006 -1.704231 -1.224377
H 3.703645 0.256201 -2.867516
H 3.790609 -2.420602 0.654488
H 2.474627 3.743768 1.226266
H 1.33362 1.575452 -0.967767
H 1.25454 -0.341346 -2.532885
H 3.314222 -1.600135 -1.653334
H 4.47167 -0.304263 0.241921
H 2.727048 0.353627 2.05377
H 4.073997 2.089439 1.234546
H 3.309247 2.137749 -0.362077
O -2.012383 -1.851523 -1.306125
O -1.174038 -1.743221 1.573469
O -1.505626 0.771454 1.980235
O -4.50958 0.64934 0.180178
O -2.890598 0.872958 -1.897918
C -2.865175 -1.960838 -0.155167
C -2.18861 -1.205858 0.978031
C -2.331474 0.216269 1.139092
C -3.261291 1.200269 0.472098
C -2.580186 1.733463 -0.829049
C -1.075629 1.947096 -0.712112
H -2.323411 0.082271 -1.852845
H -2.309001 -2.485301 -1.97206
H -0.807713 0.100717 2.167243
H -4.580679 0.573986 -0.785188
H -3.856403 -1.559854 -0.375907
H -3.166929 -0.908205 1.741303
H -3.377709 2.032104 1.17762
H -3.057047 2.69242 -1.055423
H -0.730356 2.530678 -1.572878
H -0.801867 2.458097 0.218586
H -2.939399 -3.004809 0.160634
Na 0.895656 -2.061117 1.189123

173. Reactant of 1st Iso-L2 C=O Migration

46

O -0.481397 0.646388 -0.759486
O 0.843509 -1.751253 -1.067196
O 3.597423 0.449297 -1.928842
O 3.13152 -1.758859 0.895624
O 1.225169 0.385962 0.724577
O 2.12641 2.845677 1.198076
C 0.90052 0.620633 -0.640736
C 1.447612 -0.537079 -1.469109
C 2.971492 -0.631764 -1.264396
C 3.3987 -0.528369 0.216017
C 2.624486 0.548927 0.982019
C 3.120023 1.971379 0.70411
H -0.121006 -1.704231 -1.224377
H 3.703645 0.256201 -2.867516
H 3.790609 -2.420602 0.654488
H 2.474627 3.743768 1.226266
H 1.33362 1.575452 -0.967767
H 1.25454 -0.341346 -2.532885
H 3.314222 -1.600135 -1.653334
H 4.47167 -0.304263 0.241921
H 2.727048 0.353627 2.05377
H 4.073997 2.089439 1.234546
H 3.309247 2.137749 -0.362077
O -2.012383 -1.851523 -1.306125
O -1.174038 -1.743221 1.573469
O -1.505626 0.771454 1.980235
O -4.50958 0.64934 0.180178
O -2.890598 0.872958 -1.897918
C -2.865175 -1.960838 -0.155167
C -2.18861 -1.205858 0.978031
C -2.331474 0.216269 1.139092
C -3.261291 1.200269 0.472098
C -2.580186 1.733463 -0.829049
C -1.075629 1.947096 -0.712112
H -2.323411 0.082271 -1.852845
H -2.309001 -2.485301 -1.97206
H -0.807713 0.100717 2.167243
H -4.580679 0.573986 -0.785188
H -3.856403 -1.559854 -0.375907
H -3.166929 -0.908205 1.741303
H -3.377709 2.032104 1.17762
H -3.057047 2.69242 -1.055423
H -0.730356 2.530678 -1.572878
H -0.801867 2.458097 0.218586
H -2.939399 -3.004809 0.160634
Na 0.895656 -2.061117 1.189123

174. Transition States of 2nd Iso-L2 C=O Migration

46

O 0.514019 0.663581 0.772596
O -0.897524 -1.745562 1.092366
O -3.563899 0.569004 1.907151
O -3.159454 -1.704301 -0.878245
O -1.17948 0.394479 -0.727236
O -2.007377 2.869263 -1.239418
C -0.868624 0.635919 0.636724
C -1.442792 -0.503607 1.475157
C -2.969386 -0.543102 1.263759
C -3.382659 -0.452261 -0.222325
C -2.569367 0.58442 -1.003606
C -3.034241 2.024226 -0.759445
H 0.066192 -1.777383 1.296154
H -3.692767 0.386775 2.845058
H -3.852503 -2.330022 -0.635946
H -2.340453 3.771235 -1.303351
H -1.301253 1.596125 0.952413
H -1.248101 -0.301215 2.537962
H -3.346206 -1.492907 1.665983
H -4.447025 -0.190864 -0.258328
H -2.665707 0.3671 -2.071498
H -3.971053 2.159656 -1.315436
H -3.244916 2.211133 0.299218
O 1.83899 -1.759155 1.478739
O 1.119529 -1.874463 -1.493769
O 1.509266 0.579723 -2.047263
O 4.521828 0.557217 -0.279017
O 2.958014 0.900537 1.783609
C 2.69465 -1.986215 0.370361
C 2.128662 -1.328965 -0.888013
C 2.305704 0.072396 -1.152132
C 3.264468 1.086681 -0.564278
C 2.624852 1.747905 0.687
C 1.112148 1.943954 0.601447
H 3.015042 1.402812 2.605427
H 1.981216 -0.8436 1.772537
H 0.816922 -0.109841 -2.203
H 4.585379 0.408002 0.675821
H 3.710905 -1.644438 0.58601
H 3.138422 -1.130811 -1.629068
H 3.374454 1.861219 -1.332211
H 3.107828 2.724154 0.809012
H 0.784452 2.608616 1.411822
H 0.819746 2.384681 -0.360212
H 2.711855 -3.061845 0.19004
Na -0.946747 -2.208116 -1.105674

175. Reactant of 2nd Iso-L2 C=O Migration

46

O 0.514019 0.663581 0.772596
O -0.897524 -1.745562 1.092366
O -3.563899 0.569004 1.907151
O -3.159454 -1.704301 -0.878245
O -1.17948 0.394479 -0.727236
O -2.007377 2.869263 -1.239418
C -0.868624 0.635919 0.636724
C -1.442792 -0.503607 1.475157
C -2.969386 -0.543102 1.263759
C -3.382659 -0.452261 -0.222325
C -2.569367 0.58442 -1.003606
C -3.034241 2.024226 -0.759445
H 0.066192 -1.777383 1.296154
H -3.692767 0.386775 2.845058
H -3.852503 -2.330022 -0.635946
H -2.340453 3.771235 -1.303351
H -1.301253 1.596125 0.952413
H -1.248101 -0.301215 2.537962
H -3.346206 -1.492907 1.665983
H -4.447025 -0.190864 -0.258328
H -2.665707 0.3671 -2.071498
H -3.971053 2.159656 -1.315436
H -3.244916 2.211133 0.299218
O 1.83899 -1.759155 1.478739
O 1.119529 -1.874463 -1.493769
O 1.509266 0.579723 -2.047263
O 4.521828 0.557217 -0.279017
O 2.958014 0.900537 1.783609
C 2.69465 -1.986215 0.370361
C 2.128662 -1.328965 -0.888013
C 2.305704 0.072396 -1.152132
C 3.264468 1.086681 -0.564278
C 2.624852 1.747905 0.687
C 1.112148 1.943954 0.601447
H 3.015042 1.402812 2.605427
H 1.981216 -0.8436 1.772537
H 0.816922 -0.109841 -2.203
H 4.585379 0.408002 0.675821
H 3.710905 -1.644438 0.58601
H 3.138422 -1.130811 -1.629068
H 3.374454 1.861219 -1.332211
H 3.107828 2.724154 0.809012
H 0.784452 2.608616 1.411822
H 0.819746 2.384681 -0.360212
H 2.711855 -3.061845 0.19004
Na -0.946747 -2.208116 -1.105674

176. Transition States of 3rd Iso-L2 C=O Migration

46

O -0.493011 0.160209 -1.475494
O 1.58961 -1.558287 -1.530093
O 3.605647 1.429861 -1.171218
O 2.961301 -1.316277 1.095785
O 0.669474 0.258419 0.450299
O 0.598435 2.754906 1.476243
C 0.709546 0.592815 -0.927729
C 1.852059 -0.171368 -1.593816
C 3.178641 0.114363 -0.871892
C 3.059854 0.041942 0.664513
C 1.798614 0.735392 1.185956
C 1.898096 2.260928 1.201142
H 0.707012 -1.723588 -1.906852
H 4.055191 1.455125 -2.023794
H 3.827383 -1.73911 1.056647
H 0.664706 3.675907 1.75229
H 0.834779 1.678234 -1.052741
H 1.943303 0.15995 -2.637791
H 3.914193 -0.633952 -1.1967
H 3.94986 0.516913 1.094406
H 1.616324 0.394794 2.210408
H 2.608887 2.529795 1.992925
H 2.28323 2.65908 0.257044
O -0.828894 -2.804825 -1.023449
O -0.994446 -2.191244 1.64426
O -1.614756 0.17624 2.216055
O -3.860901 0.080212 -0.473639
O -2.975815 2.747789 -0.917964
C -2.082315 -2.312013 -0.542883
C -1.825693 -1.640673 0.813884
C -2.105428 -0.252322 1.090785
C -2.949747 0.74854 0.359073
C -2.085343 1.754651 -0.434943
C -1.464643 1.174268 -1.706614
H -3.028585 3.489708 -0.305147
H -0.951602 -3.703677 -1.346683
H -1.014956 -0.562816 2.509839
H -4.422672 0.74785 -0.892882
H -2.511349 -1.61164 -1.25887
H -2.997768 -1.486354 1.269849
H -3.471238 1.327905 1.135086
H -1.308979 2.166395 0.224856
H -2.242235 0.714365 -2.320214
H -1.02384 2.002184 -2.273589
H -2.769842 -3.147431 -0.38938
Na 0.925746 -2.280438 0.577288

177. Reactant of 3rd Iso-L2 C=O Migration

46

O -0.493011 0.160209 -1.475494
O 1.58961 -1.558287 -1.530093
O 3.605647 1.429861 -1.171218
O 2.961301 -1.316277 1.095785
O 0.669474 0.258419 0.450299
O 0.598435 2.754906 1.476243
C 0.709546 0.592815 -0.927729
C 1.852059 -0.171368 -1.593816
C 3.178641 0.114363 -0.871892
C 3.059854 0.041942 0.664513
C 1.798614 0.735392 1.185956
C 1.898096 2.260928 1.201142
H 0.707012 -1.723588 -1.906852
H 4.055191 1.455125 -2.023794
H 3.827383 -1.73911 1.056647
H 0.664706 3.675907 1.75229
H 0.834779 1.678234 -1.052741
H 1.943303 0.15995 -2.637791
H 3.914193 -0.633952 -1.1967
H 3.94986 0.516913 1.094406
H 1.616324 0.394794 2.210408
H 2.608887 2.529795 1.992925
H 2.28323 2.65908 0.257044
O -0.828894 -2.804825 -1.023449
O -0.994446 -2.191244 1.64426
O -1.614756 0.17624 2.216055
O -3.860901 0.080212 -0.473639
O -2.975815 2.747789 -0.917964
C -2.082315 -2.312013 -0.542883
C -1.825693 -1.640673 0.813884
C -2.105428 -0.252322 1.090785
C -2.949747 0.74854 0.359073
C -2.085343 1.754651 -0.434943
C -1.464643 1.174268 -1.706614
H -3.028585 3.489708 -0.305147
H -0.951602 -3.703677 -1.346683
H -1.014956 -0.562816 2.509839
H -4.422672 0.74785 -0.892882
H -2.511349 -1.61164 -1.25887
H -2.997768 -1.486354 1.269849
H -3.471238 1.327905 1.135086
H -1.308979 2.166395 0.224856
H -2.242235 0.714365 -2.320214
H -1.02384 2.002184 -2.273589
H -2.769842 -3.147431 -0.38938
Na 0.925746 -2.280438 0.577288

178. Transition States of 4th Iso-L2 C=O Migration

46

O	-0.37118	0.3751	-0.34013
O	-1.064817	0.944993	2.020026
O	-4.509111	0.727685	0.982889
O	-1.91616	-1.857921	0.962482
O	-2.486397	0.620474	-1.232992
O	-5.051197	-0.508686	-1.466686
C	-1.546799	1.147003	-0.338063
C	-2.13197	1.158539	1.092475
C	-3.273473	0.131887	1.260092
C	-3.031601	-1.127966	0.420064
C	-2.733563	-0.77428	-1.057527
C	-3.880887	-1.116177	-1.986955
H	-1.355077	1.236788	2.892902
H	-4.771252	0.572204	0.059457
H	-2.254082	-2.594854	1.483052
H	-5.781194	-0.562177	-2.092522
H	-1.329579	2.15933	-0.687094
H	-2.582656	2.142057	1.258805
H	-3.287837	-0.171505	2.315911
H	-3.930822	-1.75156	0.467695
H	-1.850869	-1.349324	-1.364562
H	-3.647054	-0.734954	-2.986933
H	-3.99388	-2.207243	-2.031597
O	5.217047	0.912502	-0.902277
O	4.372273	-2.381124	-0.015968
O	2.018337	-1.555595	0.195852
O	1.766041	0.807298	1.47954
O	1.123347	2.686774	-0.393537
C	5.52617	-0.328168	-0.311818
C	4.253141	-1.090538	-0.090869
C	2.888739	-0.620007	-0.017019
C	2.513514	0.844389	0.281689
C	1.706161	1.482386	-0.869533
C	0.551671	0.637064	-1.390797
H	1.776436	3.396444	-0.370817
H	6.032382	1.374234	-1.130297
H	3.452516	-2.696669	0.211831
H	1.102132	1.517037	1.442649
H	6.178636	-0.945356	-0.939709
H	3.332028	-0.592025	-1.212864
H	3.421821	1.438476	0.427334
H	2.398553	1.670736	-1.702382
H	0.051112	1.169075	-2.20696
H	0.909712	-0.327294	-1.762318
H	6.000346	-0.211961	0.673799
Na	0.208775	-0.978931	1.362781

179. Reactant of 4th Iso-L2 C=O Migration

46

O -0.37118 0.3751 -0.34013
O -1.064817 0.944993 2.020026
O -4.509111 0.727685 0.982889
O -1.91616 -1.857921 0.962482
O -2.486397 0.620474 -1.232992
O -5.051197 -0.508686 -1.466686
C -1.546799 1.147003 -0.338063
C -2.13197 1.158539 1.092475
C -3.273473 0.131887 1.260092
C -3.031601 -1.127966 0.420064
C -2.733563 -0.77428 -1.057527
C -3.880887 -1.116177 -1.986955
H -1.355077 1.236788 2.892902
H -4.771252 0.572204 0.059457
H -2.254082 -2.594854 1.483052
H -5.781194 -0.562177 -2.092522
H -1.329579 2.15933 -0.687094
H -2.582656 2.142057 1.258805
H -3.287837 -0.171505 2.315911
H -3.930822 -1.75156 0.467695
H -1.850869 -1.349324 -1.364562
H -3.647054 -0.734954 -2.986933
H -3.99388 -2.207243 -2.031597
O 5.217047 0.912502 -0.902277
O 4.372273 -2.381124 -0.015968
O 2.018337 -1.555595 0.195852
O 1.766041 0.807298 1.47954
O 1.123347 2.686774 -0.393537
C 5.52617 -0.328168 -0.311818
C 4.253141 -1.090538 -0.090869
C 2.888739 -0.620007 -0.017019
C 2.513514 0.844389 0.281689
C 1.706161 1.482386 -0.869533
C 0.551671 0.637064 -1.390797
H 1.776436 3.396444 -0.370817
H 6.032382 1.374234 -1.130297
H 3.452516 -2.696669 0.211831
H 1.102132 1.517037 1.442649
H 6.178636 -0.945356 -0.939709
H 3.332028 -0.592025 -1.212864
H 3.421821 1.438476 0.427334
H 2.398553 1.670736 -1.702382
H 0.051112 1.169075 -2.20696
H 0.909712 -0.327294 -1.762318
H 6.000346 -0.211961 0.673799
Na 0.208775 -0.978931 1.362781

180. Transition States of 1st Iso-L3a C-C cleavage

46

O 0.209264 -1.303943 -0.535946
O -0.454548 0.95771 -1.561945
O -3.822643 -0.346594 -1.738458
O -2.364346 2.164958 0.363487
O -1.412672 -0.567154 0.821641
O -3.212622 -2.216628 2.035653
C -1.157043 -1.088535 -0.46608
C -1.439419 -0.05908 -1.570677
C -2.818327 0.585523 -1.38636
C -3.113058 0.977927 0.068547
C -2.749444 -0.132618 1.061201
C -3.73317 -1.300218 1.096525
H 0.42677 0.540227 -1.50127
H -3.956299 -0.354592 -2.693386
H -2.794225 2.621873 1.096716
H -3.846583 -2.927519 2.180896
H -1.728079 -2.016279 -0.620332
H -1.425735 -0.584985 -2.534866
H -2.867374 1.493141 -2.000347
H -4.185798 1.194163 0.126567
H -2.71155 0.290643 2.071097
H -4.709512 -0.902462 1.40646
H -3.858237 -1.756407 0.109264
O 2.166562 2.905459 -0.083151
O 0.685852 1.096177 1.214693
O 2.555844 0.265791 -1.019367
O 4.710915 -1.348851 -0.066871
O 2.497779 -2.063054 -1.420153
C 2.660121 2.318316 1.115746
C 2.077651 0.905177 1.302803
C 2.735544 0.022965 0.252247
C 3.685624 -0.899414 0.679437
C 2.241886 -2.36895 -0.198884
C 0.796259 -2.319562 0.244041
H 2.395872 -0.931149 -1.496059
H 2.548706 2.422928 -0.833359
H 0.156545 0.281309 1.244484
H 4.584342 -1.11218 -0.998309
H 3.754555 2.296375 1.122982
H 2.363786 0.539142 2.298111
H 3.810598 -1.112057 1.735907
H 2.894085 -3.09787 0.287802
H 0.34438 -3.297856 0.013023
H 0.690678 -2.135137 1.319481
H 2.31513 2.952133 1.936372
Na -0.163352 2.6724 -0.139926

181. Reactant of 1st Iso-L3a C-C cleavage

46

O 0.209264 -1.303943 -0.535946
O -0.454548 0.95771 -1.561945
O -3.822643 -0.346594 -1.738458
O -2.364346 2.164958 0.363487
O -1.412672 -0.567154 0.821641
O -3.212622 -2.216628 2.035653
C -1.157043 -1.088535 -0.46608
C -1.439419 -0.05908 -1.570677
C -2.818327 0.585523 -1.38636
C -3.113058 0.977927 0.068547
C -2.749444 -0.132618 1.061201
C -3.73317 -1.300218 1.096525
H 0.42677 0.540227 -1.50127
H -3.956299 -0.354592 -2.693386
H -2.794225 2.621873 1.096716
H -3.846583 -2.927519 2.180896
H -1.728079 -2.016279 -0.620332
H -1.425735 -0.584985 -2.534866
H -2.867374 1.493141 -2.000347
H -4.185798 1.194163 0.126567
H -2.71155 0.290643 2.071097
H -4.709512 -0.902462 1.40646
H -3.858237 -1.756407 0.109264
O 2.166562 2.905459 -0.083151
O 0.685852 1.096177 1.214693
O 2.555844 0.265791 -1.019367
O 4.710915 -1.348851 -0.066871
O 2.497779 -2.063054 -1.420153
C 2.660121 2.318316 1.115746
C 2.077651 0.905177 1.302803
C 2.735544 0.022965 0.252247
C 3.685624 -0.899414 0.679437
C 2.241886 -2.36895 -0.198884
C 0.796259 -2.319562 0.244041
H 2.395872 -0.931149 -1.496059
H 2.548706 2.422928 -0.833359
H 0.156545 0.281309 1.244484
H 4.584342 -1.11218 -0.998309
H 3.754555 2.296375 1.122982
H 2.363786 0.539142 2.298111
H 3.810598 -1.112057 1.735907
H 2.894085 -3.09787 0.287802
H 0.34438 -3.297856 0.013023
H 0.690678 -2.135137 1.319481
H 2.31513 2.952133 1.936372
Na -0.163352 2.6724 -0.139926

182. Transition States of 2nd Iso-L3a C-C cleavage

46

O 0.714907 -1.172058 -1.236341
O 2.592148 -2.109696 0.451993
O 4.468604 0.616 -1.144801
O 3.435211 -0.177671 2.17981
O 1.32455 0.422303 0.24612
O 1.025867 3.003005 -0.4176
C 1.744612 -0.301332 -0.895148
C 2.959508 -1.15294 -0.5368
C 4.08775 -0.217005 -0.06811
C 3.599046 0.694723 1.067035
C 2.265519 1.378921 0.731643
C 2.366499 2.581648 -0.202351
H 2.764691 -1.720046 1.328094
H 5.235472 0.254091 -1.602641
H 3.300545 0.314898 2.998234
H 1.004224 3.846658 -0.883774
H 1.970583 0.382551 -1.725279
H 3.288884 -1.703582 -1.423758
H 4.924871 -0.811906 0.313782
H 4.372637 1.446812 1.263783
H 1.816857 1.735402 1.666928
H 2.951871 3.361734 0.299189
H 2.859444 2.334054 -1.146679
O -4.659932 -0.031014 -1.40517
O -5.252657 0.297529 1.282939
O -3.03643 -1.085663 1.467162
O -1.487184 1.726691 -0.091611
O -1.583905 -2.111703 -0.228308
C -4.431497 1.19294 -0.740411
C -4.124842 0.93871 0.740995
C -2.875543 0.104909 0.923562
C -1.624565 0.501271 0.488745
C -1.678553 -1.038414 -0.938688
C -0.479795 -0.595964 -1.757668
H -2.448924 -1.75082 0.922726
H -5.4261 -0.449404 -0.989112
H -4.97678 -0.467513 1.806221
H -0.556609 2.018782 -0.084361
H -3.591191 1.686996 -1.231263
H -3.94799 1.906805 1.234363
H -0.738235 0.04893 0.919494
H -2.638977 -0.811614 -1.418692
H -0.622314 -0.987319 -2.772716
H -0.412607 0.495655 -1.812622
H -5.31057 1.845842 -0.79224
Na 0.454723 -2.956691 0.129086

183. Reactant of 2nd Iso-L3a C-C cleavage

46

O 0.714907 -1.172058 -1.236341
O 2.592148 -2.109696 0.451993
O 4.468604 0.616 -1.144801
O 3.435211 -0.177671 2.17981
O 1.32455 0.422303 0.24612
O 1.025867 3.003005 -0.4176
C 1.744612 -0.301332 -0.895148
C 2.959508 -1.15294 -0.5368
C 4.08775 -0.217005 -0.06811
C 3.599046 0.694723 1.067035
C 2.265519 1.378921 0.731643
C 2.366499 2.581648 -0.202351
H 2.764691 -1.720046 1.328094
H 5.235472 0.254091 -1.602641
H 3.300545 0.314898 2.998234
H 1.004224 3.846658 -0.883774
H 1.970583 0.382551 -1.725279
H 3.288884 -1.703582 -1.423758
H 4.924871 -0.811906 0.313782
H 4.372637 1.446812 1.263783
H 1.816857 1.735402 1.666928
H 2.951871 3.361734 0.299189
H 2.859444 2.334054 -1.146679
O -4.659932 -0.031014 -1.40517
O -5.252657 0.297529 1.282939
O -3.03643 -1.085663 1.467162
O -1.487184 1.726691 -0.091611
O -1.583905 -2.111703 -0.228308
C -4.431497 1.19294 -0.740411
C -4.124842 0.93871 0.740995
C -2.875543 0.104909 0.923562
C -1.624565 0.501271 0.488745
C -1.678553 -1.038414 -0.938688
C -0.479795 -0.595964 -1.757668
H -2.448924 -1.75082 0.922726
H -5.4261 -0.449404 -0.989112
H -4.97678 -0.467513 1.806221
H -0.556609 2.018782 -0.084361
H -3.591191 1.686996 -1.231263
H -3.94799 1.906805 1.234363
H -0.738235 0.04893 0.919494
H -2.638977 -0.811614 -1.418692
H -0.622314 -0.987319 -2.772716
H -0.412607 0.495655 -1.812622
H -5.31057 1.845842 -0.79224
Na 0.454723 -2.956691 0.129086

184. Transition States of 3rd Iso-L3a C-C cleavage

46

O 1.243193 1.54393 1.341454
O 3.271915 1.892275 -0.209743
O 4.389524 -1.431281 0.700488
O 2.773499 -0.065035 -2.245826
O 1.166851 -0.200318 -0.077443
O 0.354862 -2.60945 0.806276
C 1.873696 0.349693 1.024589
C 3.307823 0.731875 0.616391
C 4.004598 -0.389819 -0.17303
C 3.079817 -1.020605 -1.22674
C 1.723515 -1.393508 -0.636863
C 1.692153 -2.557925 0.347384
H 3.13306 2.656373 0.366007
H 5.259951 -1.258145 1.076928
H 3.543411 0.074026 -2.810391
H 0.228877 -3.369815 1.384656
H 1.856764 -0.357771 1.866494
H 3.891228 0.931827 1.522993
H 4.870389 0.054202 -0.681542
H 3.572539 -1.903789 -1.648681
H 1.046457 -1.639057 -1.4615
H 1.976546 -3.467279 -0.198267
H 2.396159 -2.426349 1.173243
O -5.095708 -1.524947 -1.07819
O -4.740826 -0.046824 1.086543
O -2.227504 0.339975 -1.47457
O -2.269769 -0.494419 2.024999
O -0.764026 1.951701 -0.471363
C -5.039195 -0.12566 -1.304193
C -4.216588 0.421378 -0.141275
C -2.748714 0.073657 -0.301003
C -1.889484 -0.268156 0.738652
C -1.144916 1.626721 0.721765
C -0.087112 1.47061 1.830244
H -1.494 1.097509 -1.262057
H -5.698961 -1.946014 -1.699763
H -5.11845 -0.925457 0.91277
H -3.225438 -0.322623 2.110045
H -4.550998 0.119579 -2.251733
H -4.290972 1.519048 -0.144894
H -0.942182 -0.734867 0.481685
H -2.054715 2.106888 1.113526
H -0.198096 2.336552 2.489794
H -0.250309 0.567333 2.427685
H -6.038898 0.321852 -1.268018
Na 1.26709 1.655597 -1.426863

185. Reactant of 3rd Iso-L3a C-C cleavage

46

O 1.243193 1.54393 1.341454
O 3.271915 1.892275 -0.209743
O 4.389524 -1.431281 0.700488
O 2.773499 -0.065035 -2.245826
O 1.166851 -0.200318 -0.077443
O 0.354862 -2.60945 0.806276
C 1.873696 0.349693 1.024589
C 3.307823 0.731875 0.616391
C 4.004598 -0.389819 -0.17303
C 3.079817 -1.020605 -1.22674
C 1.723515 -1.393508 -0.636863
C 1.692153 -2.557925 0.347384
H 3.13306 2.656373 0.366007
H 5.259951 -1.258145 1.076928
H 3.543411 0.074026 -2.810391
H 0.228877 -3.369815 1.384656
H 1.856764 -0.357771 1.866494
H 3.891228 0.931827 1.522993
H 4.870389 0.054202 -0.681542
H 3.572539 -1.903789 -1.648681
H 1.046457 -1.639057 -1.4615
H 1.976546 -3.467279 -0.198267
H 2.396159 -2.426349 1.173243
O -5.095708 -1.524947 -1.07819
O -4.740826 -0.046824 1.086543
O -2.227504 0.339975 -1.47457
O -2.269769 -0.494419 2.024999
O -0.764026 1.951701 -0.471363
C -5.039195 -0.12566 -1.304193
C -4.216588 0.421378 -0.141275
C -2.748714 0.073657 -0.301003
C -1.889484 -0.268156 0.738652
C -1.144916 1.626721 0.721765
C -0.087112 1.47061 1.830244
H -1.494 1.097509 -1.262057
H -5.698961 -1.946014 -1.699763
H -5.11845 -0.925457 0.91277
H -3.225438 -0.322623 2.110045
H -4.550998 0.119579 -2.251733
H -4.290972 1.519048 -0.144894
H -0.942182 -0.734867 0.481685
H -2.054715 2.106888 1.113526
H -0.198096 2.336552 2.489794
H -0.250309 0.567333 2.427685
H -6.038898 0.321852 -1.268018
Na 1.26709 1.655597 -1.426863

186. Transition States of 4th Iso-L3a C-C cleavage

46

O -0.243948 -1.848731 0.910998
O -1.644748 -1.936639 -1.339066
O -4.271894 -0.472969 0.704972
O -2.300365 0.824394 -2.040851
O -0.852903 0.220355 0.290302
O -1.145397 1.814308 2.389567
C -1.327845 -0.990831 0.844874
C -2.323933 -1.611142 -0.132732
C -3.448733 -0.607198 -0.437145
C -2.923867 0.80662 -0.755852
C -1.83353 1.254392 0.219278
C -2.316734 1.684932 1.602684
H -1.086839 -2.706397 -1.155215
H -4.9325 -1.174683 0.735547
H -2.965475 0.72475 -2.732669
H -1.36504 2.242158 3.224647
H -1.774741 -0.809959 1.832747
H -2.7687 -2.510813 0.310406
H -4.013597 -0.973816 -1.304707
H -3.770866 1.502268 -0.724848
H -1.297811 2.095781 -0.230181
H -2.8402 2.642585 1.487665
H -3.017272 0.967219 2.040784
O 2.144194 3.640889 -0.031761
O 1.004036 1.641114 -1.257859
O 1.976738 -0.798168 -1.335558
O 4.427317 -1.386163 -0.008889
O 2.054666 -2.652222 0.119569
C 2.871506 2.425932 0.011878
C 1.811221 1.330804 -0.131298
C 2.452399 -0.031333 -0.39251
C 3.542151 -0.439881 0.364989
C 2.106471 -1.934275 1.185496
C 0.794006 -1.478602 1.801002
H 1.845447 -1.924659 -0.733055
H 2.743437 4.393962 -0.077973
H 0.935248 2.608538 -1.285471
H 4.087947 -1.890601 -0.76318
H 3.58657 2.358883 -0.818002
H 1.181295 1.323402 0.770543
H 3.910828 0.170492 1.181928
H 2.939006 -2.11527 1.869191
H 0.672533 -2.017985 2.751534
H 0.773446 -0.402815 2.012338
H 3.406375 2.320003 0.962179
Na -0.168121 -0.149899 -1.968145

187. Reactant of 4th Iso-L3a C-C cleavage

46

O -0.243948 -1.848731 0.910998
O -1.644748 -1.936639 -1.339066
O -4.271894 -0.472969 0.704972
O -2.300365 0.824394 -2.040851
O -0.852903 0.220355 0.290302
O -1.145397 1.814308 2.389567
C -1.327845 -0.990831 0.844874
C -2.323933 -1.611142 -0.132732
C -3.448733 -0.607198 -0.437145
C -2.923867 0.80662 -0.755852
C -1.83353 1.254392 0.219278
C -2.316734 1.684932 1.602684
H -1.086839 -2.706397 -1.155215
H -4.9325 -1.174683 0.735547
H -2.965475 0.72475 -2.732669
H -1.36504 2.242158 3.224647
H -1.774741 -0.809959 1.832747
H -2.7687 -2.510813 0.310406
H -4.013597 -0.973816 -1.304707
H -3.770866 1.502268 -0.724848
H -1.297811 2.095781 -0.230181
H -2.8402 2.642585 1.487665
H -3.017272 0.967219 2.040784
O 2.144194 3.640889 -0.031761
O 1.004036 1.641114 -1.257859
O 1.976738 -0.798168 -1.335558
O 4.427317 -1.386163 -0.008889
O 2.054666 -2.652222 0.119569
C 2.871506 2.425932 0.011878
C 1.811221 1.330804 -0.131298
C 2.452399 -0.031333 -0.39251
C 3.542151 -0.439881 0.364989
C 2.106471 -1.934275 1.185496
C 0.794006 -1.478602 1.801002
H 1.845447 -1.924659 -0.733055
H 2.743437 4.393962 -0.077973
H 0.935248 2.608538 -1.285471
H 4.087947 -1.890601 -0.76318
H 3.58657 2.358883 -0.818002
H 1.181295 1.323402 0.770543
H 3.910828 0.170492 1.181928
H 2.939006 -2.11527 1.869191
H 0.672533 -2.017985 2.751534
H 0.773446 -0.402815 2.012338
H 3.406375 2.320003 0.962179
Na -0.168121 -0.149899 -1.968145

188. Transition States of 1st Iso-L3a C=O Migration

46

O -0.201477 0.126005 1.45919
O -1.581475 -2.070033 0.900288
O -4.471478 0.037075 1.506893
O -3.12647 -1.495501 -1.492249
O -1.515112 0.525469 -0.355002
O -4.520912 2.283179 -1.339333
C -1.511596 0.329958 1.057613
C -2.279694 -0.943825 1.410736
C -3.690444 -0.909331 0.802734
C -3.690741 -0.46618 -0.670692
C -2.817078 0.762063 -0.90991
C -3.431634 2.087738 -0.461546
H -0.749334 -2.140848 1.390201
H -4.875465 -0.365565 2.28406
H -3.772184 -2.196412 -1.639196
H -5.059549 3.024462 -1.0427
H -1.947072 1.211343 1.551176
H -2.370734 -1.012178 2.502129
H -4.116135 -1.919253 0.866652
H -4.715899 -0.235901 -0.976765
H -2.657418 0.842579 -1.989818
H -3.765407 2.055531 0.57982
H -2.665996 2.867317 -0.578498
O 4.818588 1.163763 -1.267006
O 4.140852 0.715028 1.608571
O 3.644241 -2.045268 -0.586147
O 1.279093 -1.30988 -0.90107
O 2.479783 1.98382 -0.071712
C 5.52482 0.297071 -0.384645
C 4.541813 -0.227465 0.664375
C 3.381708 -0.880903 -0.062658
C 2.004684 -0.470535 -0.216495
C 1.490648 0.981348 -0.077378
C 0.701863 1.196237 1.216704
H 3.054527 1.912685 -0.851963
H 5.429999 1.556905 -1.900474
H 3.727529 1.472683 1.155585
H 2.817816 -2.284968 -1.086655
H 5.949716 -0.548994 -0.936121
H 5.052735 -1.029329 1.209252
H 2.25584 -0.884615 0.957683
H 0.825601 1.091933 -0.94046
H 1.403527 1.21698 2.056443
H 0.17293 2.154427 1.166911
H 6.318628 0.831642 0.146979
Na -0.83862 -1.587885 -1.257878

189. Reactant of 1st Iso-L3a C=O Migration

46

O -0.201477 0.126005 1.45919
O -1.581475 -2.070033 0.900288
O -4.471478 0.037075 1.506893
O -3.12647 -1.495501 -1.492249
O -1.515112 0.525469 -0.355002
O -4.520912 2.283179 -1.339333
C -1.511596 0.329958 1.057613
C -2.279694 -0.943825 1.410736
C -3.690444 -0.909331 0.802734
C -3.690741 -0.46618 -0.670692
C -2.817078 0.762063 -0.90991
C -3.431634 2.087738 -0.461546
H -0.749334 -2.140848 1.390201
H -4.875465 -0.365565 2.28406
H -3.772184 -2.196412 -1.639196
H -5.059549 3.024462 -1.0427
H -1.947072 1.211343 1.551176
H -2.370734 -1.012178 2.502129
H -4.116135 -1.919253 0.866652
H -4.715899 -0.235901 -0.976765
H -2.657418 0.842579 -1.989818
H -3.765407 2.055531 0.57982
H -2.665996 2.867317 -0.578498
O 4.818588 1.163763 -1.267006
O 4.140852 0.715028 1.608571
O 3.644241 -2.045268 -0.586147
O 1.279093 -1.30988 -0.90107
O 2.479783 1.98382 -0.071712
C 5.52482 0.297071 -0.384645
C 4.541813 -0.227465 0.664375
C 3.381708 -0.880903 -0.062658
C 2.004684 -0.470535 -0.216495
C 1.490648 0.981348 -0.077378
C 0.701863 1.196237 1.216704
H 3.054527 1.912685 -0.851963
H 5.429999 1.556905 -1.900474
H 3.727529 1.472683 1.155585
H 2.817816 -2.284968 -1.086655
H 5.949716 -0.548994 -0.936121
H 5.052735 -1.029329 1.209252
H 2.25584 -0.884615 0.957683
H 0.825601 1.091933 -0.94046
H 1.403527 1.21698 2.056443
H 0.17293 2.154427 1.166911
H 6.318628 0.831642 0.146979
Na -0.83862 -1.587885 -1.257878

190. Transition States of 2nd Iso-L3a C=O Migration

46

O 0.219062 0.184664 -1.358233
O 1.716214 -1.98296 -1.141438
O 4.412479 0.359316 -1.422851
O 3.060887 -1.682419 1.321952
O 1.531846 0.496835 0.465617
O 4.845145 1.891334 1.007676
C 1.526351 0.422 -0.966768
C 2.363724 -0.758595 -1.463265
C 3.755999 -0.736462 -0.824909
C 3.686765 -0.558008 0.698399
C 2.82168 0.64175 1.0776
C 3.44813 2.015096 0.787919
H 1.000529 -2.112421 -1.777551
H 5.122864 0.685938 -0.850538
H 3.635458 -2.455003 1.256763
H 5.243407 2.761979 1.119934
H 1.900169 1.363433 -1.388088
H 2.49277 -0.662221 -2.546942
H 4.256738 -1.686475 -1.06253
H 4.69275 -0.416381 1.106234
H 2.628445 0.56302 2.151623
H 3.255467 2.324438 -0.243145
H 3.002351 2.751429 1.464508
O -4.874693 1.130806 1.296324
O -4.143046 0.821315 -1.581339
O -3.729658 -2.056725 0.476442
O -1.36325 -1.375228 0.871944
O -2.495964 1.973919 0.19998
C -5.574533 0.319444 0.357956
C -4.577477 -0.164002 -0.697234
C -3.441759 -0.871837 0.016868
C -2.061873 -0.491456 0.216364
C -1.522724 0.956681 0.164282
C -0.709485 1.230919 -1.102019
H -3.091986 1.861355 0.959287
H -5.49283 1.498725 1.938319
H -3.731547 1.549034 -1.080547
H -2.914905 -2.331692 0.979312
H -6.020353 -0.547867 0.857438
H -5.088207 -0.927701 -1.294589
H -2.297242 -0.841573 -0.981871
H -0.869155 1.006723 1.042186
H -1.39101 1.273693 -1.957629
H -0.198729 2.194948 -1.002778
H -6.351224 0.890918 -0.160382
Na 0.772297 -1.714939 0.98102

191. Reactant of 2nd Iso-L3a C=O Migration

46

O	0.219062	0.184664	-1.358233
O	1.716214	-1.98296	-1.141438
O	4.412479	0.359316	-1.422851
O	3.060887	-1.682419	1.321952
O	1.531846	0.496835	0.465617
O	4.845145	1.891334	1.007676
C	1.526351	0.422	-0.966768
C	2.363724	-0.758595	-1.463265
C	3.755999	-0.736462	-0.824909
C	3.686765	-0.558008	0.698399
C	2.82168	0.64175	1.0776
C	3.44813	2.015096	0.787919
H	1.000529	-2.112421	-1.777551
H	5.122864	0.685938	-0.850538
H	3.635458	-2.455003	1.256763
H	5.243407	2.761979	1.119934
H	1.900169	1.363433	-1.388088
H	2.49277	-0.662221	-2.546942
H	4.256738	-1.686475	-1.06253
H	4.69275	-0.416381	1.106234
H	2.628445	0.56302	2.151623
H	3.255467	2.324438	-0.243145
H	3.002351	2.751429	1.464508
O	-4.874693	1.130806	1.296324
O	-4.143046	0.821315	-1.581339
O	-3.729658	-2.056725	0.476442
O	-1.36325	-1.375228	0.871944
O	-2.495964	1.973919	0.19998
C	-5.574533	0.319444	0.357956
C	-4.577477	-0.164002	-0.697234
C	-3.441759	-0.871837	0.016868
C	-2.061873	-0.491456	0.216364
C	-1.522724	0.956681	0.164282
C	-0.709485	1.230919	-1.102019
H	-3.091986	1.861355	0.959287
H	-5.49283	1.498725	1.938319
H	-3.731547	1.549034	-1.080547
H	-2.914905	-2.331692	0.979312
H	-6.020353	-0.547867	0.857438
H	-5.088207	-0.927701	-1.294589
H	-2.297242	-0.841573	-0.981871
H	-0.869155	1.006723	1.042186
H	-1.39101	1.273693	-1.957629
H	-0.198729	2.194948	-1.002778
H	-6.351224	0.890918	-0.160382
Na	0.772297	-1.714939	0.98102

192. Transition States of 3rd Iso-L3a C=O Migration

46

O -0.333796 0.222222 1.406208
O -1.765347 -1.994672 1.008834
O -4.635447 0.201451 1.312841
O -3.226927 -1.564024 -1.518445
O -1.577045 0.427432 -0.484352
O -2.29402 2.977941 -0.80854
C -1.624099 0.404692 0.93726
C -2.44346 -0.803946 1.386022
C -3.829199 -0.795881 0.718191
C -3.774345 -0.463266 -0.786942
C -2.842872 0.711025 -1.087864
C -3.386777 2.087234 -0.703816
H -0.945355 -2.035406 1.522645
H -5.036528 -0.124283 2.126944
H -3.867236 -2.284068 -1.564325
H -2.608272 3.885297 -0.72731
H -2.048586 1.350347 1.302488
H -2.580127 -0.76953 2.474421
H -4.268137 -1.795831 0.836396
H -4.792442 -0.239959 -1.12681
H -2.633363 0.718802 -2.16193
H -4.186462 2.330444 -1.416021
H -3.819749 2.095424 0.301338
O 4.768342 0.90967 -1.283542
O 4.017618 0.770509 1.604587
O 3.515369 -2.188204 -0.314811
O 1.178776 -1.454301 -0.765493
O 2.415975 1.887458 -0.223897
C 5.43569 0.124065 -0.301058
C 4.418571 -0.271219 0.771676
C 3.262957 -0.972875 0.084117
C 1.898532 -0.558201 -0.148481
C 1.405039 0.90711 -0.167089
C 0.582045 1.261015 1.073797
H 2.99575 1.745647 -0.989788
H 5.40137 1.2163 -1.942884
H 3.615397 1.477756 1.06668
H 2.693199 -2.460328 -0.807314
H 5.853784 -0.78259 -0.753054
H 4.899272 -1.023645 1.407405
H 2.111641 -0.857515 1.067661
H 0.767654 0.950537 -1.056576
H 1.260724 1.370324 1.925777
H 0.05338 2.204249 0.898378
H 6.229535 0.693904 0.192852
Na -0.928719 -1.728022 -1.15445

193. Reactant of 3rd Iso-L3a C=O Migration

46

O -0.333796 0.222222 1.406208
O -1.765347 -1.994672 1.008834
O -4.635447 0.201451 1.312841
O -3.226927 -1.564024 -1.518445
O -1.577045 0.427432 -0.484352
O -2.29402 2.977941 -0.80854
C -1.624099 0.404692 0.93726
C -2.44346 -0.803946 1.386022
C -3.829199 -0.795881 0.718191
C -3.774345 -0.463266 -0.786942
C -2.842872 0.711025 -1.087864
C -3.386777 2.087234 -0.703816
H -0.945355 -2.035406 1.522645
H -5.036528 -0.124283 2.126944
H -3.867236 -2.284068 -1.564325
H -2.608272 3.885297 -0.72731
H -2.048586 1.350347 1.302488
H -2.580127 -0.76953 2.474421
H -4.268137 -1.795831 0.836396
H -4.792442 -0.239959 -1.12681
H -2.633363 0.718802 -2.16193
H -4.186462 2.330444 -1.416021
H -3.819749 2.095424 0.301338
O 4.768342 0.90967 -1.283542
O 4.017618 0.770509 1.604587
O 3.515369 -2.188204 -0.314811
O 1.178776 -1.454301 -0.765493
O 2.415975 1.887458 -0.223897
C 5.43569 0.124065 -0.301058
C 4.418571 -0.271219 0.771676
C 3.262957 -0.972875 0.084117
C 1.898532 -0.558201 -0.148481
C 1.405039 0.90711 -0.167089
C 0.582045 1.261015 1.073797
H 2.99575 1.745647 -0.989788
H 5.40137 1.2163 -1.942884
H 3.615397 1.477756 1.06668
H 2.693199 -2.460328 -0.807314
H 5.853784 -0.78259 -0.753054
H 4.899272 -1.023645 1.407405
H 2.111641 -0.857515 1.067661
H 0.767654 0.950537 -1.056576
H 1.260724 1.370324 1.925777
H 0.05338 2.204249 0.898378
H 6.229535 0.693904 0.192852
Na -0.928719 -1.728022 -1.15445

194. Transition States of 4th Iso-L3a C=O Migration

46

O 0.82197 -1.196759 -0.869022
O 3.148259 -1.795358 -0.044511
O 3.779673 1.805189 -0.170915
O 1.470876 -0.126363 1.731154
O 1.367638 0.968709 -1.423974
O -0.206496 3.307655 0.684223
C 1.794871 -0.356988 -1.435704
C 3.111256 -0.498781 -0.633385
C 3.26024 0.630908 0.414673
C 1.904436 1.008259 1.00053
C 0.944726 1.4218 -0.1406
C 0.778517 2.927195 -0.280324
H 4.064182 -2.059456 0.098267
H 4.741522 1.772004 -0.213408
H 0.503526 -0.134039 1.823284
H -0.127675 4.246866 0.886369
H 1.940217 -0.615795 -2.490718
H 3.933561 -0.389434 -1.349952
H 3.89641 0.260782 1.229927
H 2.029288 1.861643 1.678857
H -0.053127 1.004481 0.076363
H 1.730226 3.437737 -0.105656
H 0.430713 3.146118 -1.295665
O -2.238903 2.011595 -0.747615
O -3.622719 -0.297371 -1.439865
O -2.65257 0.287325 1.952164
O -0.906213 -1.449739 1.620563
O -0.719201 -3.256817 -0.413393
C -3.535229 1.800087 -0.2216
C -3.740301 0.284511 -0.167997
C -2.693165 -0.2678 0.77583
C -1.738281 -1.347553 0.620656
C -1.38525 -2.040556 -0.715274
C -0.416938 -1.220929 -1.557924
H -1.359842 -3.940657 -0.183571
H -1.72683 2.619816 -0.184029
H -3.083493 0.290102 -1.990445
H -1.882953 -0.154563 2.408764
H -3.657215 2.232198 0.774841
H -4.735893 0.056249 0.229747
H -2.889354 -1.794652 0.881773
H -2.2897 -2.207448 -1.303168
H -0.287673 -1.70683 -2.533568
H -0.798056 -0.20166 -1.70051
H -4.291606 2.221936 -0.892916
Na 1.115088 -2.368258 1.015605

195. Reactant of 4th Iso-L3a C=O Migration

46

O 0.82197 -1.196759 -0.869022
O 3.148259 -1.795358 -0.044511
O 3.779673 1.805189 -0.170915
O 1.470876 -0.126363 1.731154
O 1.367638 0.968709 -1.423974
O -0.206496 3.307655 0.684223
C 1.794871 -0.356988 -1.435704
C 3.111256 -0.498781 -0.633385
C 3.26024 0.630908 0.414673
C 1.904436 1.008259 1.00053
C 0.944726 1.4218 -0.1406
C 0.778517 2.927195 -0.280324
H 4.064182 -2.059456 0.098267
H 4.741522 1.772004 -0.213408
H 0.503526 -0.134039 1.823284
H -0.127675 4.246866 0.886369
H 1.940217 -0.615795 -2.490718
H 3.933561 -0.389434 -1.349952
H 3.89641 0.260782 1.229927
H 2.029288 1.861643 1.678857
H -0.053127 1.004481 0.076363
H 1.730226 3.437737 -0.105656
H 0.430713 3.146118 -1.295665
O -2.238903 2.011595 -0.747615
O -3.622719 -0.297371 -1.439865
O -2.65257 0.287325 1.952164
O -0.906213 -1.449739 1.620563
O -0.719201 -3.256817 -0.413393
C -3.535229 1.800087 -0.2216
C -3.740301 0.284511 -0.167997
C -2.693165 -0.2678 0.77583
C -1.738281 -1.347553 0.620656
C -1.38525 -2.040556 -0.715274
C -0.416938 -1.220929 -1.557924
H -1.359842 -3.940657 -0.183571
H -1.72683 2.619816 -0.184029
H -3.083493 0.290102 -1.990445
H -1.882953 -0.154563 2.408764
H -3.657215 2.232198 0.774841
H -4.735893 0.056249 0.229747
H -2.889354 -1.794652 0.881773
H -2.2897 -2.207448 -1.303168
H -0.287673 -1.70683 -2.533568
H -0.798056 -0.20166 -1.70051
H -4.291606 2.221936 -0.892916
Na 1.115088 -2.368258 1.015605
